# Multicomponent synthesis of 2*H*-chromene-fused-thiazolo-triazole derivatives *via* cascade Michael addition/cyclization reaction: anticancer, antibacterial and computational evaluations

**DOI:** 10.1039/d6ra03687b

**Published:** 2026-05-21

**Authors:** Barsha Samanta, Tapaswini Pati, Ananya Dash, Bhabani Shankar Panda, Eeshara Naik, Seetaram Mohapatra, Chita Ranjan Sahoo, Pradeep Kumar Naik

**Affiliations:** a Organic Synthesis Laboratory, Department of Chemistry, Ravenshaw University Cuttack-753003 Odisha India seetaram.mohapatra@gmail.com; b Centre of Excellence in Natural Products and Therapeutic, Department of Biotechnology and Bioinformatics, Sambalpur University Jyoti Vihar, Burla Sambalpur 768019 Odisha India; c ICMR-Regional Medical Research Centre, Department of Health Research, Ministry of Health & Family Welfare, Govt. of India Bhubaneswar-751023 Odisha India

## Abstract

We report an efficient approach for the synthesis of a series of 2*H*-chromene-fused-thiazolo-triazole derivatives. This method enables the synthesis of the desired compounds through a multicomponent cascade of C–N and C–S bond formation. The synthesized compounds were thoroughly characterized by ^1^H NMR, ^13^C NMR, HRMS, and single-crystal XRD. The anticancer results showed that in the MCF-7 cell line, all tested compounds exhibited better activity than the other two cancer cell lines (MDA-MB-231 and A549). Compounds 4f, 4h and 4i showed significant cytotoxicity against the MCF-7 cell line compared to Doxorubicin. Additionally, *in vitro* antibacterial activities were evaluated against *E. coli* (PDB ID: 3G7E) and *S. aureus* (PDB ID: 3G7B), where compounds 4a, 4e, and 4i showed the highest potency compared to Gentamicin. Molecular docking studies further supported these findings, indicating the strong binding affinities of the active compounds towards the selected protein targets. FMO analysis based on global reactivity parameters indicated that compounds 4a, 4e, 4f, 4h and 4i exhibited high stability. Additionally, MEP plots revealed that these compounds exhibited a strong electrophilic reaction potential. In addition, ADMET predictions indicated favorable physicochemical and pharmacokinetic properties of these potent compounds. Overall, compounds 4a, 4e, 4f, 4h and 4i were identified as promising dual anticancer and antibacterial inhibitors for future drug discovery.

## Abstract 2

An efficient synthesis of 2*H*-chromene-fused thiazolo-triazoles *via* a multicomponent cascade C–N/C–S bond-forming strategy is reported. Biological evaluation and computational studies identify potent dual anticancer and antibacterial candidates.

## Graphical abstract

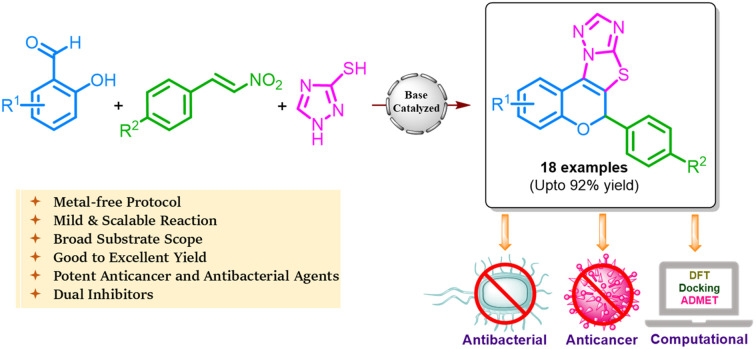

## 1. Introduction

Cancer and infectious diseases are a major global health burden.^1^ According to estimates, cancer-related mortality is expected to nearly double in the coming decades.^2^ Although chemotherapy remains the cornerstone of cancer treatment, it suffers from limited selectivity, severe side effects, and emergence of multidrug resistance.^3,4^ Concurrently, infectious diseases continue to pose a serious challenge owing to the rising resistance of pathogens to existing antimicrobial agents.^5,6^ Cancer patients undergoing chemotherapy are particularly susceptible to microbial infections because of compromised immune function.^7^ Therefore, the development of dual-acting agents with anticancer and antimicrobial activities may offer a cost-effective therapeutic strategy by reducing drug administration frequency, minimizing side effects, and limiting the development of antimicrobial resistance.

Heterocyclic frameworks are vital for the design of biologically active molecules due to their presence in a vast array of natural products, pharmaceuticals, and agrochemicals.^8,9^ Among the, fused polyheterocyclic compounds, characterized by multiple rings and heteroatoms, have gained considerable prominence due to their unique physicochemical properties, conformational rigidity, and potential for enhanced target specificity in therapeutic applications.^10^ These scaffolds often exhibit improved pharmacokinetic profiles, membrane permeability, and binding affinity, making them highly attractive candidates for drug discovery and development.^11,12^ Consequently, the construction of these complex frameworks using concise, step-efficient, and eco-friendly methods remains a key research focus.

In this regard, multicomponent reactions (MCRs) have emerged as powerful one-pot synthetic strategies for the rapid construction of structurally complex heterocycles in an atom-economic manner.^13^ By integrating three or more reactants into a single operation, MCRs minimize waste generation, reduce the reaction steps and energy consumption, and eliminate intermediate purification processes.^14^ Owing to these advantages, MCRs have gained considerable attention for the efficient synthesis of diverse natural product-like heterocyclic frameworks.

Among the privileged heterocycles, thiazoles and 1,2,4-triazoles stand out as two privileged nitrogen- and sulfur-containing heterocyclic cores widely recognized for their broad pharmacological utility.^15–18^ These heterocycles are found in a variety of FDA-approved drugs and bioactive agents with antibacterial, antifungal, anti-inflammatory, and anticancer properties.^19–23^ Their fusion into thiazolo[3,2-*b*][1,2,4]triazole scaffolds yields a rigid, conjugated framework featuring multiple hydrogen bond donors and acceptors, aromatic π-systems, and reactive heteroatoms, the attributes of which contribute to high affinity and selectivity for biological targets.^24–27^ However, their synthetic accessibility remains a challenge, often requiring multistep protocols, harsh reagents, or transition metal catalysts, which can reduce efficiency, increase cost, and complicate scalability.

In parallel, our research on 2*H*-chromene derivatives recognized them as biologically significant scaffolds with both antibacterial and anticancer activities. Our studies have shown that 2*H*-chromenes act as effective inhibitors of bacterial DNA gyrase, a validated target essential for DNA replication.^28–30^ In addition to their antibacterial potential, these derivatives are well documented for their anticancer properties, including their ability to modulate cell proliferation, induce apoptosis, and interfere with key tumor-related signaling pathways. Their structural modularity further supports their use in scaffold-fusion strategies to enhance biological performance.^31,32^

Based on these considerations, we planned a synthetic approach for the construction of 2*H*-chromene-fused-thiazolo-triazole derivatives *via* a multicomponent cascade *aza*-Michael addition and cyclization reaction, under base-catalyzed conditions. Following the synthesis, the compounds were assessed for *in vitro* anticancer and antibacterial activities, together with DFT, molecular docking and ADMET evaluation.

## 2. Results and discussion

### 2.1. Chemistry

#### 2.1.1. Synthesis

To achieve our objective, we began the study by devising a practical strategy and optimizing the model reaction between salicylaldehyde (1a), *trans-β*-nitrostyrene (2a), and 1,2,4-triazole-3-thiol (3). Initially, a series of organic and inorganic bases was screened under identical conditions employing equimolar amounts of each substrate (Table 1), based on previously reported literature protocols.^28^ Among the bases examined, DABCO emerged as the most efficient, affording the desired product in 65% yield in 1 hour (entry 2). However, the other bases failed to deliver the product in comparison to this outcome.

**Table 1:** Screening of base for the synthesis of 2*H*-chromene-fused-thiazolo-triazole derivatives^a^

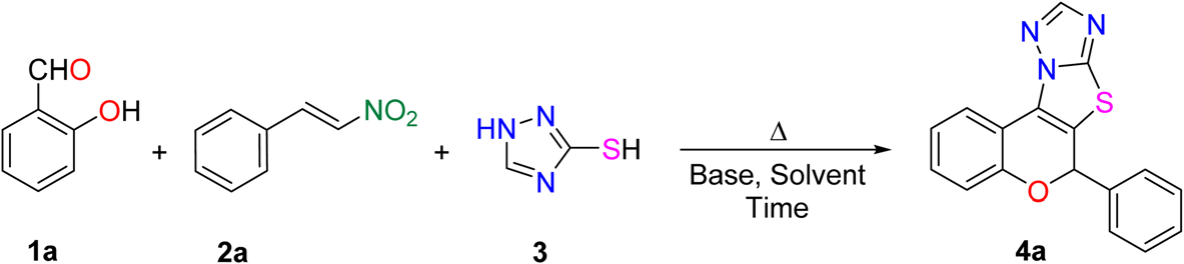				
Entry	Base	Equiv.	Time (h)	Yield^b^ (%)
1	NEt_3_	1	1	53
**2**	**DABCO**	**1**	**1.5**	**65**
3	Cs_2_CO_3_	1	2.5	12
4	l-Proline	1	2	n.r.
5	K_2_CO_3_	1	2.5	21
6	Na_2_CO_3_	1	3	20

a Reaction condition: salicylaldehyde (1a, 1 equiv.), *trans*-β-nitrostyrene (2a, 1 equiv.), 1,2,4-triazole-3-thiol (3, 1 equiv.), EtOH (3 mL), 90 °C.

b Isolated yield; n.r.: no reaction.

After screening the base, we attempted to improve the methodology by screening the solvent, temperature, time, and substrate equivalents (Table 2). Initially solvents such as toluene, methanol, and water were ineffective, even under elevated temperatures or microwave irradiation, affording either no reaction or only a moderate yield (entries 1–6). Using ethanol as the solvent under microwave heating at 90 °C and 30 W yielded 73%, while conventional heating resulted in a diminished yield with unreacted starting materials (entries 7 and 8). Upon switching the solvent to THF, the reaction did not proceed under ambient conditions (entry 9), whereas heating rendered THF optimal, affording a 65% yield (entry 10). Reducing the base to 0.5 equivalents at 100 °C improved the yield to 77% (entry 11). Further increasing the thiol to 1.2 equivalents significantly enhanced the efficiency, providing an excellent isolated yield of 89% of 4a in 1 h at 90 °C (entry 12). In contrast, increasing the temperature to 110 °C proved detrimental, leading to a significant decline in yield (entry 13). Then using the same equivalent of substrates under microwave irradiation at 80 °C and 30 W also furnished an unsatisfactory yield (entry 14).

**Table 2:** Optimization of reaction conditions for the synthesis of 2*H*-chromene-fused-thiazolo-triazole derivatives

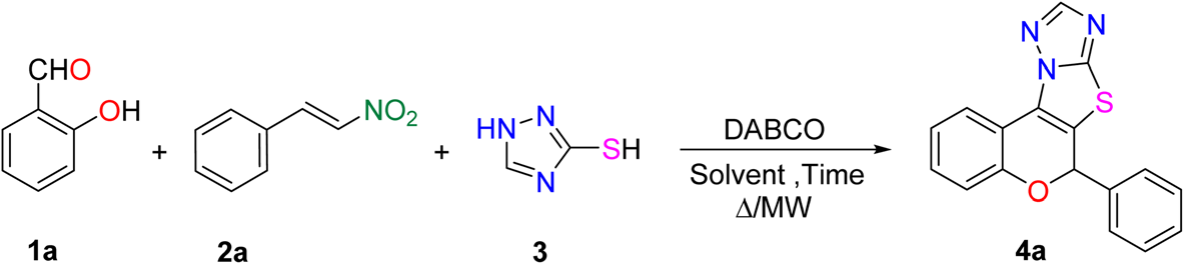						
Entry	Solvent	Equiv. of thiol (3)	Equiv. of DABCO	Temperature (in °C) and power (in W)	Time (h/min)	Yield^b^ (%)
1	Toluene	1.0	1.0	rt	24 h	n.r.
2	Toluene	1.0	1.0	110 °C, 50 W	30 min	n.r.
3	MeOH	1.0	1.0	rt	5 h	n.r.
4	MeOH	1.0	1.0	60 °C	3 h	54%
5	H_2_O	1.0	1.0	100 °C	9 h	n.r.
6	H_2_O	1.0	1.0	90 °C, 30 W	30 min	n.r.
7	EtOH	1.0	1.0	90 °C, 30 W	30 min	73%
8	EtOH	1.0	1.0	90 °C	1.5 h	65%
9	THF	1.0	1.0	rt	48 h	n.r.
10	THF	1.0	1.0	80 °C	9 h	65%
11	THF	1.0	0.5	100 °C	1 h	77%
**12** a	**THF**	**1.2**	**0.5**	**90 °C**	**1 h**	**89%**
13	THF	1.2	0.5	110 °C	1 h	47%
14	THF	1.2	0.5	80 °C, 30 W	30 min	71%

a Standard condition: salicylaldehyde (1a, 1 equiv.), *trans*-β-nitrostyrene (2a, 1 equiv.), 1,2,4-triazole-3-thiol (3, 1.2 equiv.), DABCO (0.5 equiv.), time (1 h), THF (3 mL), 90 °C.

b Isolated yield; n.r.: no reaction.

**Scheme 1:**
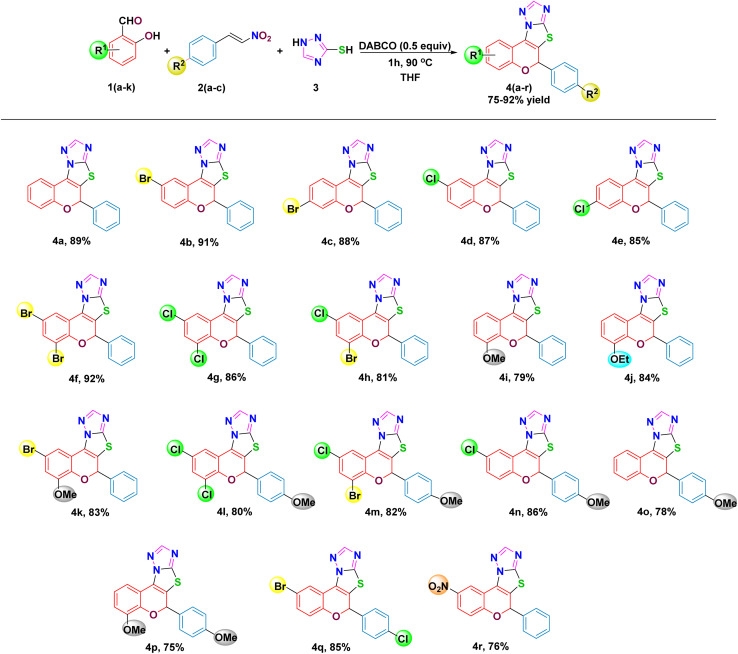
Substrate scope of multicomponent synthesis of 2*H*-chromene-fused-thiazolo-triazole derivatives 4(a–r).

With the optimized conditions in hand, the generality of this one-pot cascade protocol was systematically explored using a wide range of substituted aromatic aldehydes 1 and nitroolefin 2, while maintaining 1,2,4-triazole-3-thiol 3 as the Michael donor (Scheme 1). The reaction proceeded smoothly in almost all the cases and the results are summarized in Scheme 1. The product yields were dependent on the substituents and their positions on the aromatic ring of salicylaldehyde and nitroolefin.

At first, salicylaldehyde without any substitution 1a furnished fused heterocycle 4a in an excellent yield of 89%, thereby establishing the intrinsic efficiency of the protocol. Motivated by this result, aldehydes bearing electron-withdrawing halogen substituents were examined. The –Br substitution at the 2- and 3-positions of the aldehyde ring was well accommodated, delivering the corresponding products (4b and 4c) in 91% and 88% yields, respectively. Likewise, –Cl substituted derivatives at the 2- and 3-positions underwent the cascade sequence efficiently delivering 4d and 4e, although with a marginal decrease in yield relative to their bromo counterparts.

The di-substituted systems incorporating 2,4-dibromo and 2,4-dichloro patterns on the benzopyran ring were also compatible with the reaction conditions, providing target compounds 4f and 4g in 92% and 86% yields, respectively. In contrast, the presence of a –Br substituent at C4 and with a –Cl group at the C2 position resulted in a moderate reduction in the isolated yield to 81%, reflecting the influence of steric and electronic effects (4h).

Further introduction of aldehydes with electron-rich groups was less compatible. 4-OMe and 4-OEt substituted aldehydes afforded the corresponding products (4i, 4j) in reduced yields of 79% and 84% respectively. Moreover, the introduction of 2-Br-4-OMe substitution led to a slightly diminished yield of the desired Michael adduct (4k).

**Fig. 1:**
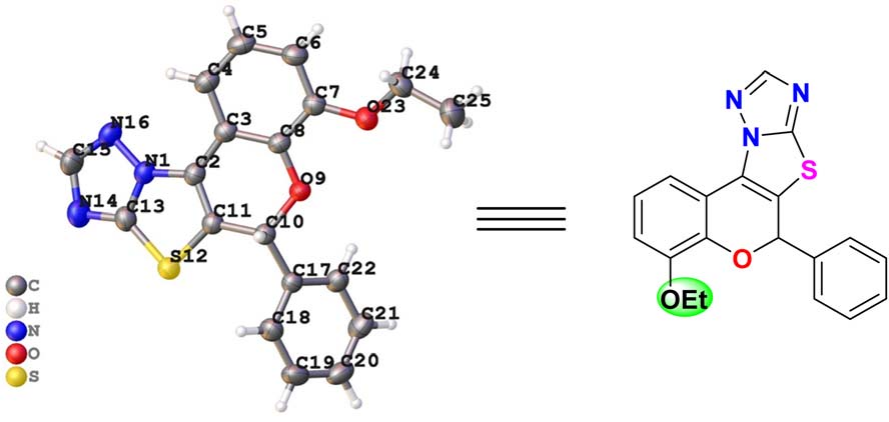
The ORTEP diagram of the compound 4j (CCDC: 2309815).

Subsequently, with the extension of the scope to substituted nitroolefins revealed that –OMe substituted nitroolefins combined with electron-deficient aldehydes afforded products (4l–4o) in consistently good yields ranging from 78% to 86%, whereas the presence of -OMe groups on both reaction partners led to diminished yield (4p). Notably, introduction of an electron-withdrawing –Cl substituent on the nitroolefin restored reactivity, providing the corresponding product (4q) in 85% yield. Further, with 2-NO_2_ substitution the yield was found to be moderate with 76% (4r). Overall, these results highlight the broad applicability of the protocol, which tolerates diverse electronic environments while delivering the desired product in good to high yields. To confirm the structures of the synthesized Michael adducts, all compounds were characterized and confirmed by ^1^H NMR, ^13^C NMR, and HRMS spectral data. Additionally, the structure of compound 4j was determined by single-crystal X-ray diffraction (CCDC deposition no. 2309815), as shown in Fig. 1.

**Fig. 2:**
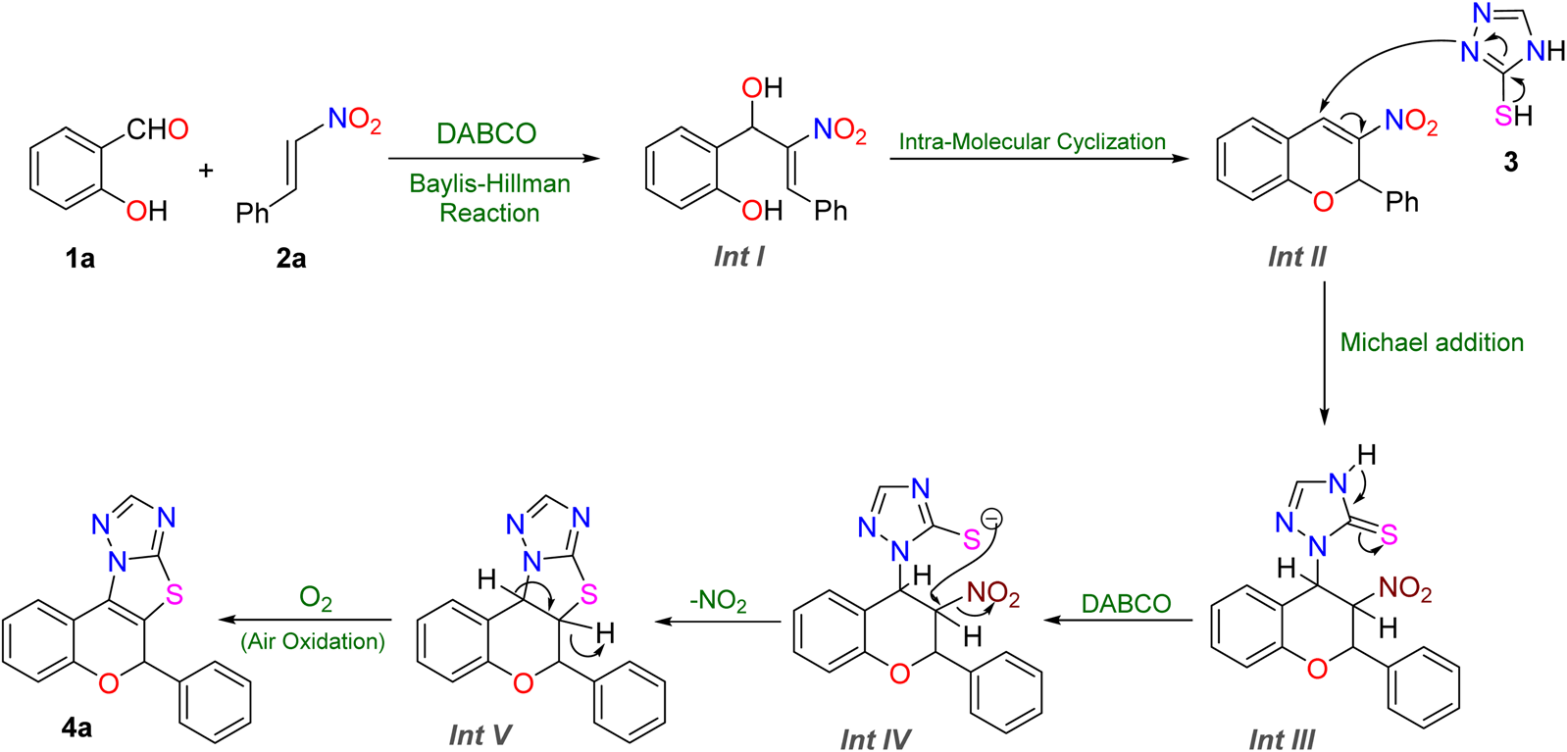
Plausible mechanism of the reaction.

Based on the above results, we propose a possible mechanism for this method. First, the reaction is initiated by a DABCO-catalyzed Baylis–Hillman reaction between salicylaldehyde 1a and nitroolefin 2a, generating the *β*-hydroxy nitroalkene intermediate int I. This intermediate subsequently underwent intramolecular cyclization *via* nucleophilic attack of the phenoxide oxygen on the activated alkene moiety, leading to the formation of chromene int II. Under basic conditions, intermediate II undergoes Michael addition with the thiol-containing heterocycle 3 to generate intermediate int III. This intermediate undergoes tautomerization to produce intermediate IV. Following this, the nucleophilic thiol component attacks the C3 position, resulting in the elimination of –NO_2_ and subsequent cyclization *via* a C–S bond, furnishing intermediate V. Finally, int V is then oxidized, delivering the final Michael adduct 4a (Fig. 2).

### 2.2. Biological study

#### 2.2.1. Anticancer evaluation

##### 2.2.1.1. *In vitro* cytotoxicity study

All newly synthesized compounds 4(a–q) were evaluated for *in vitro* cytotoxic activity against three human cancer cell lines, MCF-7 (breast cancer),^33^ MDA-MB-231 (breast cancer),^34^ A549 (lung cancer),^30^ and a noncancerous human embryonic kidney cell line (HEK-293),^35^ using the MTT colorimetric assay.^36^ The IC_50_ values (in mean ± SD) for all tested compounds against three cancer cell lines and one noncancer cell line are presented in Table 3. The MTT assay showed that cell viability was reduced in the MCF-7, MDA-MB-231, and A549 cancer cell lines in a dose-dependent manner. However, the HEK-293 cells displayed higher IC_50_ values (>200 µM). Conversely, the synthesized compounds showed strong cytotoxic effects on breast cancer cells (particularly MCF-7) than on healthy cells. Among all synthesized compounds, 4f and 4h exhibited the lowest IC_50_ values compared to the others. Compound 4f showed highest antiproliferative potency with IC_50_ values of 1.73 ± 0.24 µM, 12.36 ± 0.85 µM, and 20.51 ± 1.03 µM in MCF-7, MDA-MB-231, and A549 cells, respectively. Similarly, compound 4h demonstrated effective antiproliferative potency against MCF-7, MDA-MB-231, and A549 cells, with IC_50_ values of 5.47 ± 0.72 µM, 14.43 ± 1.24 µM, and 35.78 ± 1.45 µM respectively. Also, compound 4i and 4k displayed favorable antiproliferative activity with IC_50_ values of 8.12 ± 0.84 µM and 12.15 ± 0.98 µM towards MCF-7 cell line. However, the remaining compounds exhibited limited inhibitory activities against the tested cell lines.

**Table 3:** IC_50_ values^a^ (in µM) of all synthesized compounds 4(a–q) on four different cell lines

Sl. no.	Compounds	IC_50_ (mean ± SD) µM			
		HEK-293^b^	MCF-7^c^	MDA-MB-231^d^	A549^e^
1	4a	289.37 ± 2.46	36.95 ± 1.56	58.43 ± 1.87	71.27 ± 2.15
2	4b	303.93 ± 2.48	21.89 ± 1.34	42.93 ± 1.76	53.27 ± 1.75
3	4c	507.62 ± 2.85	15.20 ± 1.28	33.21 ± 1.92	61.45 ± 2.16
4	4d	218.10 ± 2.34	28.04 ± 1.44	56.73 ± 1.74	65.62 ± 2.11
5	4e	573.45 ± 2.76	139.50 ± 2.17	162.90 ± 2.05	155.12 ± 2.00
6	4f	240.80 ± 2.38	1.73 ± 0.24	12.36 ± 0.85	20.51 ± 1.03
7	4g	227.00 ± 2.36	22.66 ± 1.35	52.81 ± 1.38	64.45 ± 1.92
8	4h	244.91 ± 2.64	5.47 ± 0.72	14.43 ± 1.24	35.78 ± 1.45
9	4i	244.86 ± 2.16	8.12 ± 0.84	25.68 ± 1.37	36.52 ± 1.33
10	4j	523.33 ± 2.92	42.08 ± 1.85	57.18 ± 1.11	83.60 ± 1.74
11	4k	246.29 ± 1.67	12.15 ± 0.98	33.40 ± 1.69	41.31 ± 1.06
12	4l	293.84 ± 2.47	138.81 ± 2.13	146.54 ± 2.39	172.08 ± 1.83
13	4m	354.47 ± 2.55	107.73 ± 2.03	141.33 ± 1.57	188.24 ± 2.01
14	4n	549.90 ± 2.74	119.25 ± 2.07	140.60 ± 2.35	147.66 ± 1.86
15	4o	634.78 ± 3.17	176.82 ± 2.36	185.52 ± 1.75	193.41 ± 2.92
16	4p	476.11 ± 2.56	161.16 ± 2.38	188.73 ± 2.44	197.04 ± 2.18
17	4q	250.62 ± 2.38	120.71 ± 2.08	73.22 ± 2.02	79.35 ± 1.83
18	Doxorubicin	88.32 ± 2.63	1.87 ± 0.18	1.95 ± 0.16	2.38 ± 0.34

a Half maximal inhibitory concentration (IC_50_) value: the data were presented as mean ± SD values from three different experiments performed in triplicates.

b Human embryonic kidney cell line.

c Estrogen receptor-positive breast cancer cell line.

d Triple-negative breast cancer cell line.

e Lung cancer cell line.

##### 2.2.1.2. Hoechst 33342 and acridine orange staining for apoptosis

**Fig. 3:**
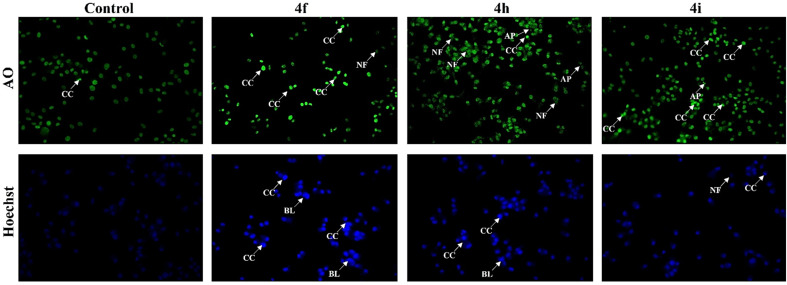
Induction of apoptosis in the MCF-7 cells determined by acridine orange and Hoechst staining. MCF-7 cells were treated with (IC_50_) concentration of 4f, 4h and 4i. The different apoptotic characteristics were determined by chromatin condensation (CC), blebbing (BL), and nuclear fragmentation.

Apoptotic cells are characterized by the formation of apoptotic bodies, chromatin condensation, cellular shrinkage, and membrane blebbing, which are essential morphological alterations associated with programmed cell death.^37^ As depicted in Fig. 3, treatment with the potent compounds 4f, 4h and 4i at their respective IC_50_ concentrations induced notable apoptotic changes in the MCF-7 breast cancer cells. Specifically, these treatments induced chromatin condensation (CC), nuclear fragmentation (NF), and membrane blebbing (BL), which are well-established hallmarks of apoptotic cell death.^38^ The Hoechst 33342 (HO) and Acridine Orange (AO) staining confirmed the presence these characteristic features, with treated cells displaying markedly enhanced fluorescence intensity compared to control cells. However, untreated cells retained their normal morphology.

#### 2.2.2. Molecular docking analysis of anticancer proteins

To rationalize the observed anticancer potential and to gain insight into the binding modes of the synthesized compounds, molecular docking studies were performed for the 2*H*-chromene-thiazolo-triazole derivatives 4(a–q) against two cancer-related protein targets, with PDB ID 1ZXN (for breast cancer) and 2W3L (for lung cancer) along with standard drug Doxorubicin. The docking visualizations revealed various types of interactions such as C–H bonds, van der Waals, conventional-H bonds, alkyl, π–σ, π–cation, π–alkyl, π–sulfur, *etc.*, of different categories (hydrogen bonds, electrostatic bonds, and hydrophobic bonds) (Fig. S55–S82). The calculated docking scores and key amino acid interactions are summarized in Table 4.

**Table 4:** Ligand–protein binding energies scores and various amino acid residues binding interactions of 2*H*-chromene-thiazolo-triazole derivatives 4(a–q)

Compounds	Breast cancer (PDB ID: 1ZXN)		Lung cancer (PDB ID: 2W3L)	
	Docking score (kcal mol^−1^)	Residue interactions	Docking score (kcal mol^−1^)	Residue interactions
4a	−9.4	ARG70, ILE97, ASN63, ASP66	−8.4	ALA84, VAL66, LEU72, PHE47, TYR43
4b	−9.8	ILE97, ILE113, ALA136, ARG70, ASN67, ASP66, ASN122	−8.7	GLU71, ARG81, LEU72, ALA84
4c	−9.9	ARG70, ASP66, ASN122, ALA64, ASN63, ALA136	−8.6	PHE39, PHE47, TYR43, MET50, ALA84, LEU72, GLU71
4d	−9.6	ILE186, ILE60, PHE114, ASN63, ALA136, ILE113, TYR6, ARG70	−8.5	ALA84, ARG81, GLU71, LEU72, PHE39
4e	−8.7	ARG70, ASP66, ASN122, ASN63, ILE113, ILE186	−8.1	ARG81, LEU72, ALA84
4f	**−10.8**	**TYR6, ALA136, ILE113, ASN63, ILE97, ARG70**	**−9.3**	**ARG81, ALA84, PHE39**
4g	−9.7	ARG70, SER121, TYR6, ILE113	−8.5	PHE39, TYR43, ARG81, LEU72, VAL68, ALA84
4h	−**10.3**	**ASN122, ALA136, ARG70, PHE114**	**−9.1**	**PHE47, ASP46, MET50, TYR43, PHE39, ALA84, ARG81**
4i	**−10.2**	**LYS137, ALA136, ILE113, PHE114, ILE97, ARG70**	**−8.9**	**TYR43, PHE39, PHE47, ALA84, ARG81**
4j	−9.3	ASN67, ILE97, ARG70, GLY130, ASN122	−8.3	TYR43, ARG81, ALA84, PHE39
4k	−10.1	ASN63, ILE97, ARG70, SER121, ILE113, ALA136	−8.8	TYR43, PHE39, ALA84
4l	−8.8	ASN122, ASP66, ARG70, ASN63, ILE90, ALA64, ILE186, ILE60	−7.9	ASP75, ARG81, LEU72, PHE88, ALA84, PHE47, GLU71
4m	−9.2	ASN122, ASP66, ARG70, ILE113, ALA136, PHE114, ALA64, ILE186, ASN63, ILE90, ILE60	−8.1	TYR43, ARG81, ALA84, PHE88, LEU72, MET50, VAL66
4n	−9.0	ASN122, ARG70, ASN63, ILE90, ILE60, ILE186, ALA64, ILE113, PHE114, ALA136	−8.2	ARG81, LEU72, PHE39, ALA84, TYR43, PHE47
4o	−8.3	ASN122, ARG70, ILE113, ASN63, ILE90, ILE60, ILE186, ALA64	−7.6	ARG81, LEU72, ALA84, PHE39
4p	−8.5	ASN122, ARG70, ASP66, ILE113, ALA136, ASN63, ILE90, ILE186, ALA64, ILE60	−7.5	TYR43, ALA84, LEU72, ARG81
4q	−8.9	ALA136, ASN122, ARG70, ASP66, ILE90, ILE186, ALA64, ILE60	−8.3	LEU72, ARG81, PHE39, ALA84, PHE88, PHE47
Doxorubicin	−9.5	TYR6, ARG70, GLN69, VAL127, LYS126, ILE97	−7.7	GLU71, ARG81, PHE39, MET50

**Fig. 4:**
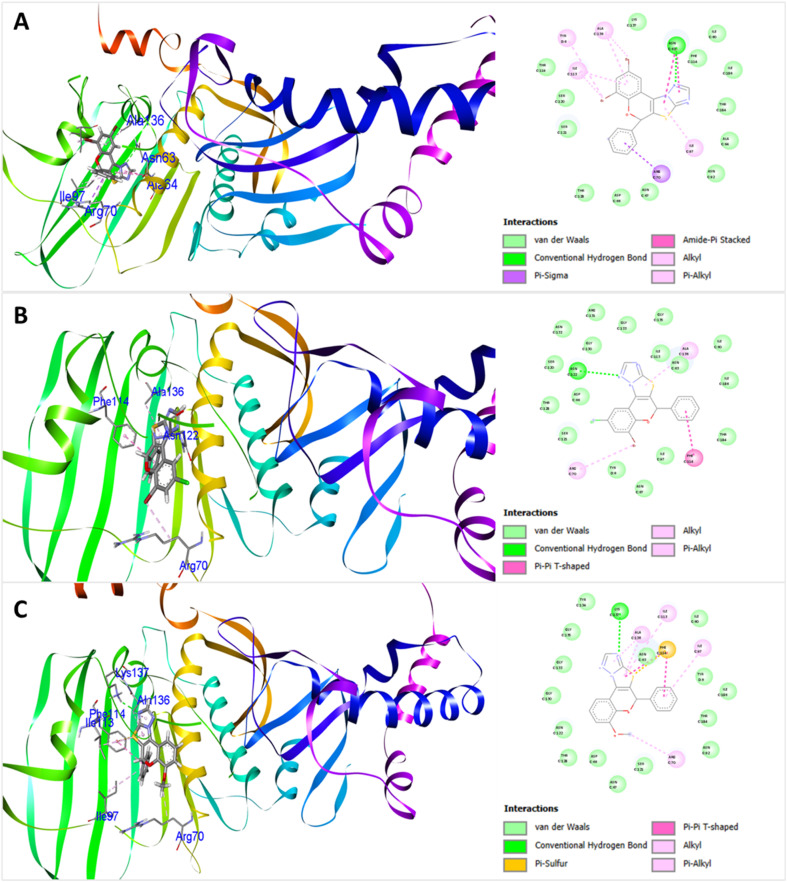
3D and 2D representation for the docking interaction of compounds 4f (A), 4h (B), and 4i (C) with DNA topoisomerase IIA (PDB ID: 1ZXN). In the docked complex, protein represents as flat ribbon and the compounds as stick model with amino acid residue interactions.

**Fig. 5:**
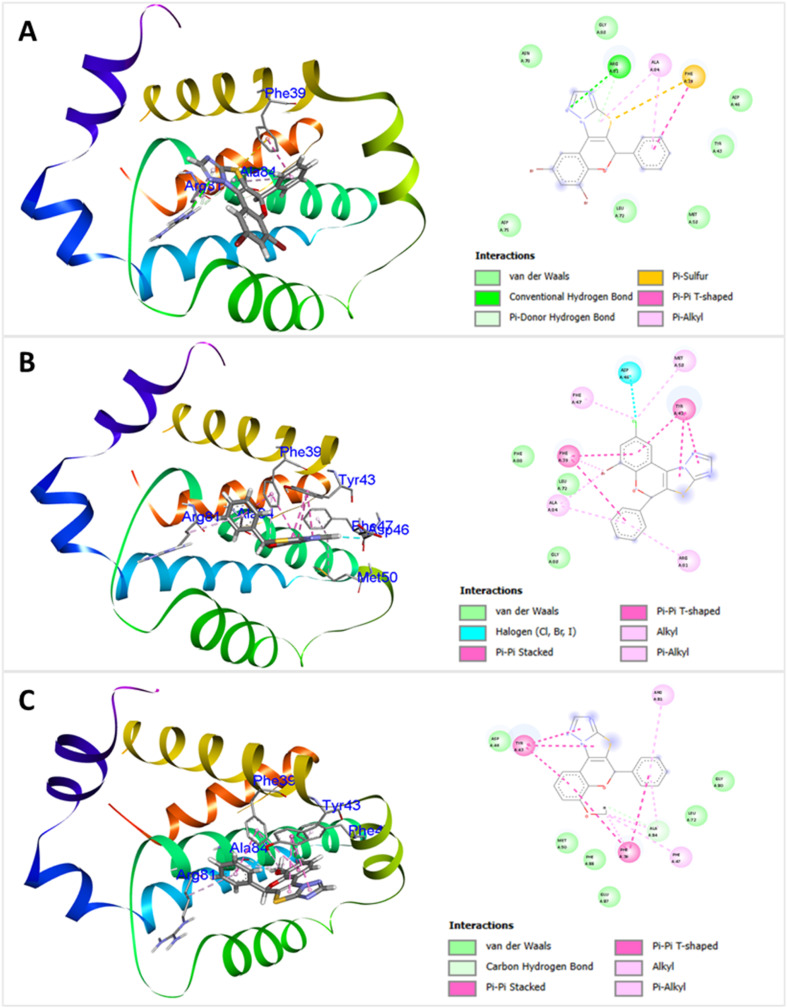
3D and 2D representation for the docking interaction of compounds 4f (A), 4h (B), and 4i (C) with apoptosis regulator BCL-2 (PDB ID: 2W3L). In the docked complex, protein represents as flat ribbon and the compounds as stick model with amino acid residue interactions.

Against the 1ZXN protein, most of the compounds exhibited favorable binding energies ranging from −8.3 kcal mol^−1^ to −10.8 kcal mol^−1^, indicating strong ligand–protein affinity. Notably, several derivatives displayed binding interactions comparable to or better than that of reference drug Doxorubicin (−9.4 kcal mol^−1^). Among the tested derivatives, compound 4f emerged as the most potent binder with a docking score of −10.8 kcal mol^−1^. This enhanced affinity can be attributed to its extensive interactions with crucial active-site residues including TYR6, ALA136, ILE113, ASN63, ILE97, and ARG70 *via* van der Waals, hydrogen bonds, π–σ, π–alkyl, alkyl, and amide–π stacking interactions. Compound 4h was the second most potent with docking score of −10.3 kcal mol^−1^. Additionally, compounds 4i each with −10.2 kcal mol^−1^ and 4k with −10.1 kcal mol^−1^ demonstrated excellent binding interactions, respectively. While 4d with −9.6 kcal mol^−1^ and 4g with −9.7 kcal mol^−1^ showed moderate binding affinities. In contrast, 4o exhibited the lowest docking scores within this series, suggesting a weaker accommodation within the 1ZXN active site. In comparison, docking against the 2W3L protein resulted in relatively lower binding energies overall, with docking scores ranging from −7.5 to −9.3 kcal mol^−1^. Nevertheless, several derivatives exhibited binding affinity compared to Doxorubicin (−7.7 kcal mol^−1^). Consistent with the 1ZXN results, compound 4f again showed the highest affinities toward the protein targets with a docking score of −9.3 kcal mol^−1^. Compounds 4h with −9.1 kcal mol^−1^ and 4k with −8.8 kcal mol^−1^ also displayed significant binding, often through π–π stacking and hydrophobic interactions. The docking scores for derivatives 4c, 4d, and 4i were moderate, ranging from −8.5 to −8.9 kcal mol^−1^. Lower docking scores were observed for compounds 4o and 4p, which had binding energies of −7.6 kcal mol^−1^ and −7.5 kcal mol^−1^, indicating a decreased binding affinity. The docking conformations and three-dimensional interaction profiles of compounds 4f, 4h, and 4i within the active sites of the two cancer-related protein targets (PDB IDs: 1ZXN and 2W3L) are shown in Fig. 4 and 5, respectively.

**Fig. 6:**
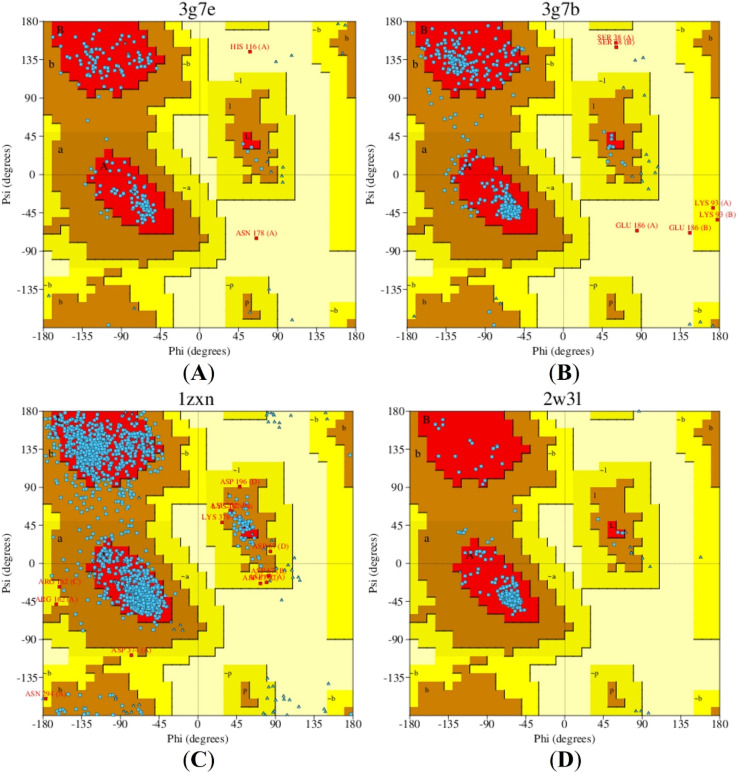
Ramachandran plots for targeted antibacterial proteins (A) 3G7E and (B) 3G7B, as well as anticancer proteins (C) 1ZXN and (D) 2W3L.

The Ramachandran plot is an essential tool in structural biology for evaluating the stability and activity of proteins, particularly in molecular docking studies that examine interactions between proteins and ligands.^39^ This plot provides a two-dimensional representation of the allowed and disallowed regions of the phi (*ϕ*) and psi (*ψ*) torsion angles of amino acid residues within proteins. The investigation of the Ramachandran plots for the 3G7B, 3G7E, 1ZXN, and 2W3L proteins revealed that 85.5%, 90.2%, 83.2%, and 96.0% of residues, respectively, were located in the most favoured regions (Fig. 6). A higher proportion of residues in these regions reflects greater structural stability, which is fundamental for maintaining proper biological function.

#### 2.2.3. Anticancer structure–activity relationships (SARs)

**Fig. 7:**
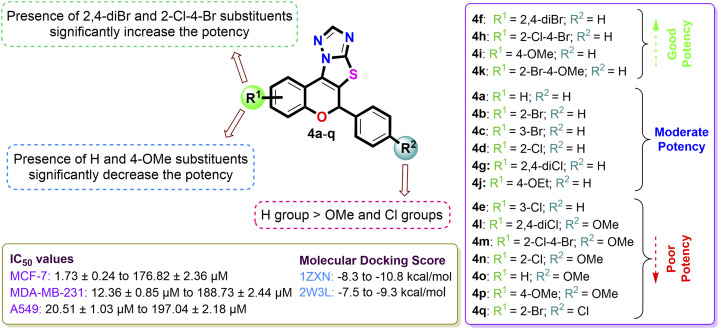
Combined structure activity relationship (SAR) for antiproliferative activity based on IC_50_ values and molecular docking study.

The structure–activity relationships (SAR) were delineated (Fig. 7) based on the *in vitro* anticancer evaluation against the MCF-7 cell line and *in silico* molecular docking studies of compounds 4(a–q). Among all, the compound 4f with R^1^ = 2,4-diBr, R^2^ = H and compound 4h with R^1^ = 2-Cl–4-Br, R^2^ = H exhibited significant anticancer effects against MCF-7 cell line with IC_50_ value of 1.73 ± 0.24 µM and 5.47 ± 0.72 µM and molecular docking score of −10.8 kcal mol^−1^ and −10.3 kcal mol^−1^ against 1ZXN protein respectively. Compounds 4k and 4i also demonstrated appreciable activity, with IC_50_ values of 8.12 ± 0.84 µM and 12.15 ± 0.98 µM. In contrast, compounds 4a, 4b, 4c, 4d, 4g and 4j, bearing bromo- and chloro-substituents, showed moderate potency, supported by their relative binding energy scores across multiple cancer-related targets. Conversely, compounds 4e, 4l, 4m, 4n, 4o, 4p, and 4q were the least active, displaying higher IC_50_ values, diminished potency, and low docking scores.

#### 2.2.4. Antibacterial evaluation

**Fig. 8:**
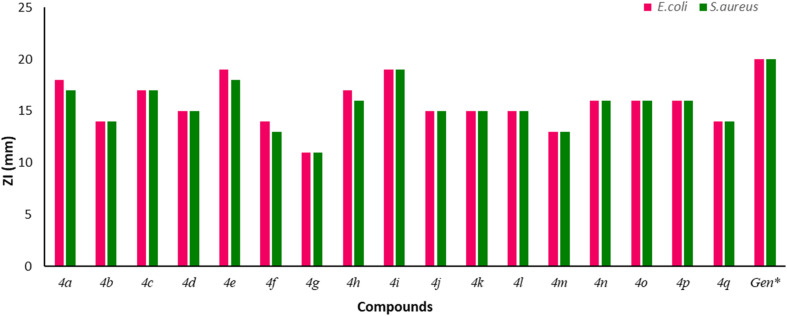
The inhibitory antimicrobial action by growth inhibition zone (ZI) assay with the synthesized 2*H*-chromeno-thiazolo-triazole derivatives investigated with Gentamicin, as positive control.

**Fig. 9:**
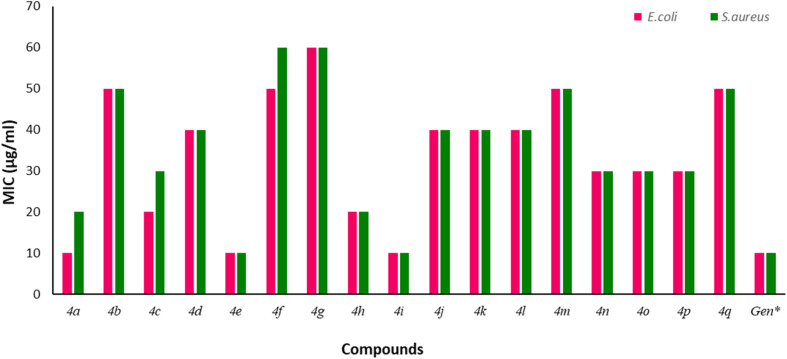
Graphical representation of the *in vitro* antimicrobial (MIC) assay of the synthesized 2*H*-chromeno-thiazolo-triazole derivatives in comparison to the standard drug, Gentamicin. The green color represented the inhibitory action of *S. aureus*, while the pink color represented the inhibitory action of *E. coli*.

The synthesized compounds were assessed for their *in vitro* antimicrobial properties against two bacterial strains, including Gram-positive *Staphylococcus aureus* and Gram-negative *Escherichia coli*. These strains were chosen due to their clinical significance and diverse resistant mechanisms.^40^ Additionally, *E. coli* is a non-pathogenic strain, making it a safe option for laboratory experiments, whereas *S. aureus* is a human pathogen, making it a pertinent target for antibacterial research.^41,42^ The minimum inhibitory concentrations (MIC) and zone of inhibition (ZI) of the compounds were determined by the broth microdilution method and agar well diffusion method with Gentamicin as the positive control. All the tested strains showed susceptibility to the antibacterial properties of the compounds. Among them, compound 4i, with a methoxy (–OCH_3_) substitution at the C4 position, demonstrated the highest bacterial inhibitory activity against both bacterial strains, with an MIC value of 10 µg mL^−1^ and a ZI value of 19 mm, respectively. Likewise, compound 4a exhibited significant antibacterial activity with an MIC of 10 µg mL^−1^ and ZI of 18 mm against *E. coli*, and an MIC of 20 µg mL^−1^ and ZI of 17 mm against *S. aureus*. Additionally, compound 4e, with a chloro (–Cl) substitution at the C3 position, exhibited notable antibacterial properties, achieving MIC values of 10 µg mL^−1^ against both tested strains. Compounds 4c and 4h displayed moderate antibacterial activities, each with an MIC of 20 µg mL^−1^ against *E. coli*. In contrast, compound 4g featuring a dichloro substitution on the benzopyran ring exhibited the lowest bacterial inhibitory potency with a ZI of 11 mm and MIC of 60 µg mL^−1^ for each Gram-positive (*S. aureus*) and Gram-negative (*E. coli*) bacterial strain. Consequently, these findings indicate that compounds 4a, 4e, and 4i are the most promising antibacterial agents. The ZI values and MIC values of all tested compounds are graphically depicted in Fig. 8 and 9, respectively.

#### 2.2.5. Molecular docking analysis of antibacterial proteins

To better understand the inhibitory mechanism of the synthesized compounds on bacterial DNA gyrase, a molecular docking analysis was performed to examine potential binding interactions. This analysis targeted the active sites of DNA gyrase receptors of *E. coli* (PDB ID: 3G7E) and *S. aureus* (PDB ID: 3G7B) within DNA gyrase subunit A, using AutoDock 4.2.0 software. DNA gyrase is a vital bacterial enzyme involved in DNA replication and transcription. DNA gyrase inhibitors such as fluoroquinolones, novobiocin, *etc.*, bind to its ATP-binding site, preventing the enzyme from breaking and rejoining DNA strands during replication, leading to bacterial cell death.^43^ However, excessive use of these inhibitors can exert a discerning burden on bacteria, promoting the development of multidrug resistance in several pathogens. This resistance can arise through various mechanisms, including mutations in the gyrA and gyrB genes, overexpression of efflux pumps, plasmid-mediated resistance genes like qnr, and reduced drug permeability.^44^ To address this issue, identifying a different binding pocket is a viable strategy. With the advent of molecular docking and drug design, new molecules that interact with alternative sites outside the conventional gyrase inhibitor binding pocket may offer an optimistic solution to tackle resistance challenges. Therefore, we selected the gyrA subunit as an alternative binding site for our *in silico* docking studies.

**Fig. 10:**
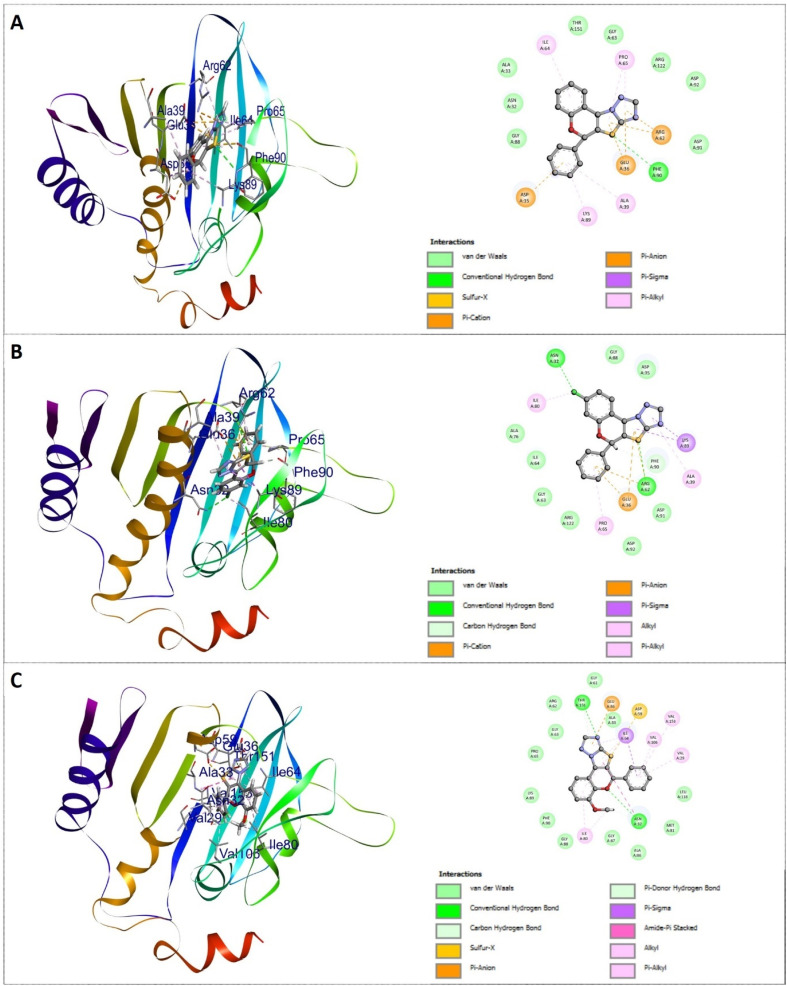
Docking illustrations and 3D interaction of compounds 4a (A), 4e (B), and 4i (C) against *E. Coli* DNA gyrase (PDB ID: 3G7E). In 3D-interaction, the proteins of *E. coli* DNA gyrase are depicted in simplified graphical cartoons containing helical and *β* strands, while the compounds 4a, 4e, and 4i are shown as stick models. The amino acids involved in the interaction are labelled.

**Fig. 11:**
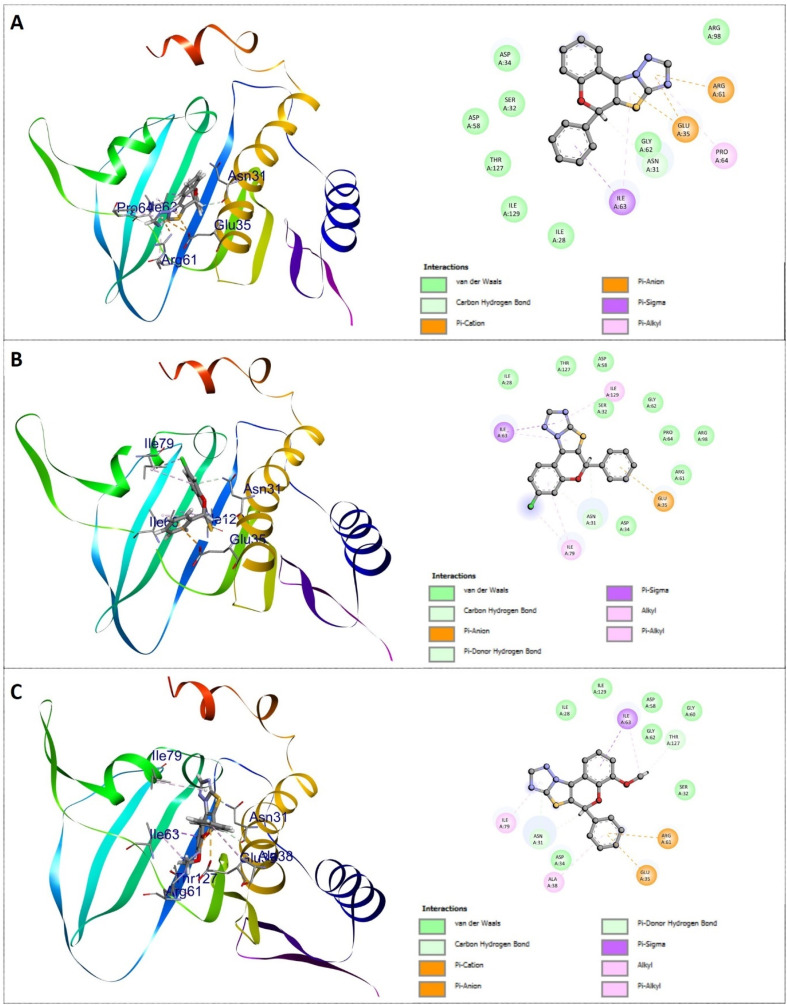
Docking illustrations and 3D interaction of compounds 4a (A), 4e (B), and 4i (C) against *S. aureus* DNA gyrase (PDB ID: 3G7B). In 3D-interaction, the proteins of *S. aureus* DNA gyrase are depicted in simplified graphical cartoons containing helical and *β* strands, while the compounds 4a, 4e, and 4i are shown as stick models. The amino acids involved in the interaction are labelled.

The results of the study demonstrated that the synthesized 2*H*-chromene-thiazole-triazole adducts interact favourably with bacterial proteins. The ligand–protein binding energies range from −6.8 kcal mol^−1^ to −9.6 kcal mol^−1^ for *E. coli* DNA gyrase and from −6.6 kcal mol^−1^ to −9.3 kcal mol^−1^ for *S. aureus* DNA gyrase. The docking study revealed that all the synthesized compounds exhibited stronger binding interactions with the DNA gyrase receptors of *E. coli* than those of *S. aureus*. Also, the docked complexes often interact with DNA gyrase amino residues *via* van der Waals forces, conventional H-bonds, π-donor hydrogen bonds, π–alkyl contacts, alkyl interactions, π–anion interactions, sulphur–X interactions, amide–π stacking, and π–σ interactions. Compound 4i demonstrated the strongest binding affinity, with a ligand–protein binding energy of −9.6 kcal mol^−1^ against *E. coli* DNA gyrase and −9.3 kcal mol^−1^ against *S. aureus* DNA gyrase. This highly potent compound binds securely into the active pockets of DNA gyrase receptors in both *E. coli* and *S. aureus* through a series of van der Waals and hydrophobic interactions with specific amino acid residues, including ILE 80, ASN 32, VAL 29, VAL 106, VAL 153, ILE 64, ASP 59, GLU 36, THR 151 of *E. coli*, and with ILE 79, ASN 31, ALA 38, GLU 35, ARG 61, THR 127, ILE 63 of *S. aureus*, respectively. The DNA gyrase inhibitor 4e was the second most potent, showing a binding affinity of −9.3 kcal mol^−1^ against *E. coli* DNA gyrase and −9.1 kcal mol^−1^ against *S. aureus* both DNA gyrase receptors. Additionally, compound 4a demonstrated active interactions with *E. coli* and *S. aureus*, with docking scores of −9.1 kcal mol^−1^ and −8.8 kcal mol^−1^, respectively. In contrast, compound 4g exhibited the lowest binding affinity, with docking scores of −6.8 kcal mol^−1^ and −6.6 kcal mol^−1^ for the DNA gyrase of *E. coli* and *S. aureus*. The corresponding docking illustrations and 3D-interaction of compounds 4i, 4e, and 4a against *E. coli* bacterial DNA gyrase (PDB ID: 3G7E) and *S. aureus* DNA gyrase (PDB ID: 3G7B) are depicted in Fig. 10 and 11 respectively. The docking scores and interactions with amino acid residues of all compounds are shown in Table 5.

**Table 5:** Docking score and binding interactions of synthesized 2*H*-chromene-fused-thiazolo-triazole derivatives 4(a–q) with amino acid residues of DNA gyrase

Compounds	*E. coli* (PDB ID: 3G7E)		*S. aureus* (PDB ID: 3G7B)	
	Docking score (kcal mol^−1^)	Residue interactions	Docking score (kcal mol^−1^)	Residue interactions
4a	**−9.1**	ILE 64, PRO 65, ARG 62, PHE 90, GLU 36, ALA 39, LYS 89, ASP 35	**−8.8**	ARG 61, GLU 35, PRO 64, ASN 31, ILE 63
4b	−7.4	ASP 59, ASN 32, ALA 33, ILE 64, PRO 65, GLU 36, ARG 62, ALA 39, LYS 89, PHE 90, ILE 80	−7.3	ARG 61, PRO 64, ILE 63, THR 127, GLU 35, ASP 34, ALA 38
4c	−8.8	ILE 64, ALA 33, ILE 80, PHE 90, GLU 36, PRO 65, ARG 62	−8.5	THR 127, GLU 35, ILE 63, PRO 64
4d	−7.7	ALA 33, ILE 64, PRO 65, GLU 36, ARG 62, ALA 39, LYS 89, PHE 90, ILE 80	−7.6	ARG 98, PRO 64, ARG 61, THR 127, ILE 63, GLU 35, ALA 38, ASP 34
4e	**−9.3**	ASN 32, ILE 80, PRO 65, GLU 36, ARG 62, PHE 90, LYS 89, ALA 39	**−9.1**	ILE 63, ILE 129, GLU 35, ASN 31, ILE 79
4f	−6.9	PRO 65, GLU 36, ARG 62, ALA 39, LYS 89, ASP 35	−6.7	ILE 79, LEU 80, ASN 31, ILE 63, ILE 129, GLU 35
4g	−6.8	LEU 38, GLU 36, ARG 62, LYS 89, ALA 39, VAL 97	−6.6	ARG 61, PRO 64, GLU 35, ILE 79, ILE 63, ILE 129, ILE 28, GLY 62, ASP 58, THR 127
4h	−8.4	PHE 90, PRO 65, ARG 62, LYS 89, ALA 39	−8.2	ASN 31, ILE 28, GLY 62, ILE 129, ILE 63, ASP 58, THR 127
4i	**−9.6**	ILE 80, ASN 32, VAL 29, VAL 106, VAL 153, ILE 64, ASP 59, GLU 36, THR 151	**−9.3**	ILE 79, ASN 31, ALA 38, GLU 35, ARG 61, THR 127, ILE 63
4j	−7.6	PRO 65, GLU 36, ASP 35, LYS 89, ALA 39, ARG 62	−7.5	ARG 61, GLU 35, PRO 64, ILE 63, ASN 31
4k	−7.8	LYS 89, ALA 39, ILE 80, GLU 36, PRO 65, ARG 62	−7.6	THR 127, GLU 35, GLY 62, ILE 63, LEU 80, ILE 28, ILE 129, ASN 31, ILE 179
4l	−7.6	LEU 38, ASP 35, HIS 102, GLU 36, ALA 39, LYS 89, ARG 62, SER 98	−7.6	ILE 63, THR 127, GLU 35, PRO 64
4m	−7.1	GLY 87, PRO 65, ILE 80, ALA 76, PHE 90, ILE 64, GLU 36, ALA 33, ASP 59, ARG 62, LYS 89	−6.7	ILE 79, PRO 64, GLU 35, ILE 63, ASN 31
4n	−8.1	ILE 80, GLU 36, ILE 64, THR 151, ASP 59, ARG 62, LYS 89	−8	GLY 62, ASN 31, ILE 79, PRO 64, ILE 63, THR 127, ASP 58
4o	−8.3	ILE 64, PRO 65, ARG 62, GLU 36, PHE 90, LYS 89, ALA 39, ASP 35	−8.2	ILE 63, GLU 35, PRO 64, ARG 61, ILE 79
4p	−8.0	ALA 39, LYS 89, ARG 62, GLU 36, ASP 59, THR 151, ILE 64, PRO 65, ILE 80, ALA 76, PHE 90	−7.9	THR 127, ILE 63, GLU 35, PRO 64, ALA 38
4q	−6.9	ILE 80, ILE 64, THR 151, PRO 65, GLY 63, GLU 36, ARG 62, LYS 89, ALA 39, PHE 90	−6.7	ILE 79, ILE 63, ASN 31, PRO 64, GLU 35
Gentamicin	−7.6	SER98, LYS89, GLU36, ASP35, ASN32, ILE64, PRO65, PHE90	−6.8	ILE28, ASP58, ILE129, ASP34, ILE63

#### 2.2.6. Antibacterial structure–activity relationships (SARs)

**Fig. 12:**
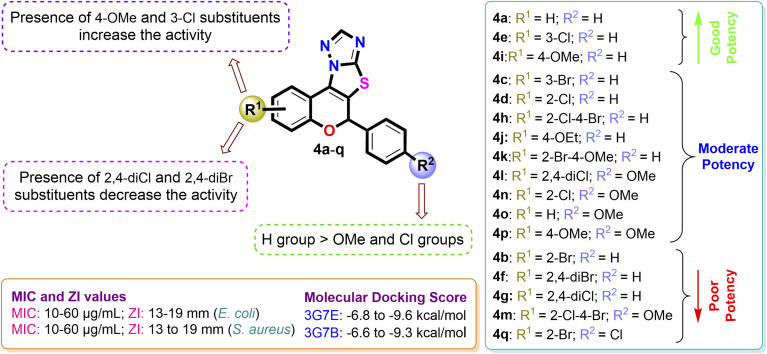
Pictorial illustration of SARs of 2*H*-chromene-fused-thiazolo-triazole derivatives 4(a–q) from antibacterial evaluation and molecular docking study.

The structure–activity relationships (SARs) were summarized by examining the screening results of the antibacterial studies of the synthesized compounds. Notably, compound 4i, with an appended 4-OMe group on the benzopyran ring, exhibited the strongest antibacterial activity against both *E. coli* and *S. aureus*, with a minimum inhibitory concentration (MIC) of 10 µg mL^−1^ and a zone of inhibition (ZI) of 19 mm. Compound 4e, containing 3-chloro substitution, also showed substantial antibacterial efficacy with MIC of 10 µg mL^−1^ against *E. coli* and *S. aureus*. Similarly, the non-substituted compound 4a exhibited notable bacterial inhibition with a ZI of 18 mm and a MIC of 10 µg mL^−1^ for *E. coli*, which was further validated by favorable binding interactions with DNA gyrase in both *E. coli* and *S. aureus* bacteria, with binding energies of −9.1 kcal mol^−1^ and −8.8 kcal mol^−1^, respectively. Compounds 4h (bearing 2-Cl–4-Br groups), 4c (containing a 3-Br substitution), and 4o (with a 4-OMe group attached to the phenyl moiety) displayed moderate interactions with bacterial proteins of both *E. coli* and *S. aureus*. However, when the substitution was altered to 4-Br on the benzopyran ring with a 4-Cl group attached to the phenyl moiety in 4g, the ZI and MIC values decreased to 14 mm and 50 µg mL^−1^ for the tested bacterial strains. Regrettably, in compound 4q with a 2,4-dichloro substitution, the antibacterial inhibition potency was the lowest, with a ZI value of 11 mm, a MIC value of 60 µg mL^−1^, and reduced binding energies *in silico* (Fig. 12).

## 3. Computational studies

### 3.1. Density functional theory (DFT) calculations

#### 3.1.1. Geometry optimization

**Fig. 13:**
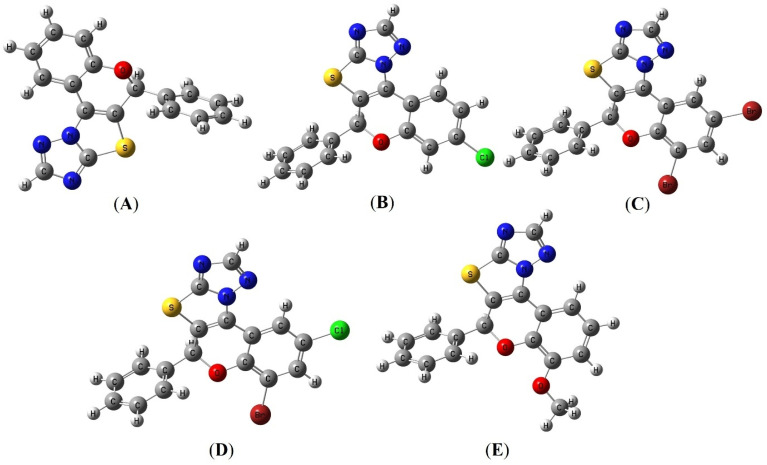
The ball-and-stick representation of the optimized structures for compounds 4a (A), 4e (B), 4f (C), 4h (D), and 4i (E).

The synthesized 2*H*-chromene-fused-thiazolo-triazole derivatives (4a–q) were structurally optimized using a hybrid correlation functional (B3LYP) combined with the 6-311G++(d,p) basis set.^45,46^ The optimized structures of these compounds are shown in Fig. 13 and S111.

#### 3.1.2. Frontier molecular orbital (FMO) analysis

The DFT calculations provide valuable insights into the electronic properties of these compounds, particularly in terms of their stability, reactivity, and charge transfer characteristics, which are critical for understanding their interactions with biological targets. According to frontier molecular orbital (FMO) theory, the energy difference between the highest molecular orbital (HOMO) and lowest molecular orbital (LUMO) is an important indicator of molecular behavior. A large HOMO–LUMO energy gap (Δ*E*_g_) results in higher kinetic stability and lower chemical reactivity, whereas a small HOMO–LUMO gap results in lower kinetic stability and higher chemical reactivity for a molecule. In general, the molecules with small Δ*E*_g_ values tend to be softer, more chemically reactive, more polarizable and less kinetically stable. Conversely, molecules with large Δ*E*_g_ values are classified as harder and exhibit high kinetic stability.^30,34^

**Fig. 14:**
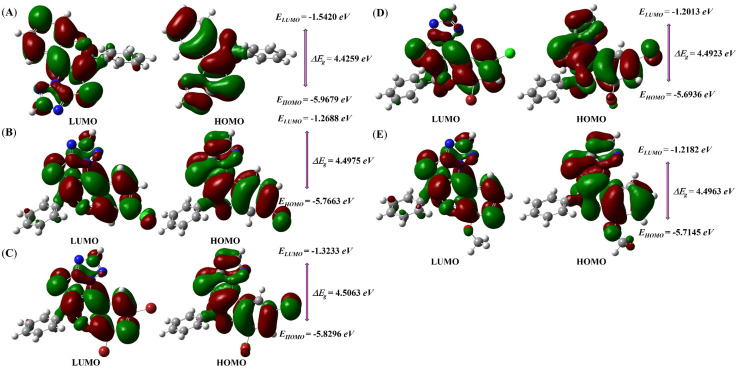
HOMO–LUMO surface maps of optimized molecular structure of synthesized 2*H*-chromene-fused-thiazolo-triazole derivatives 4a (A), 4e (B), 4f (C), 4h (D), and 4i (E).

Table 6 presents the chemical reactivity descriptors for all the synthesized compounds 4a–q. Herein, the synthesized 2*H*-chromene-fused-thiazolo-triazole derivatives 4a–q exhibited HOMO and LUMO energies (energy of LUMO = *E*_LUMO_ and energy of HOMO = *E*_HOMO_) values ranging from −6.1750 eV to −5.6462 eV and −1.7749 eV to −1.1684 eV respectively. The calculated HOMO–LUMO energy gap (Δ*E*_g_) for all synthesized compounds was in the range of 4.3724 eV to 4.5063 eV. Among all the studied compounds, five compounds 4f, 4e, 4i, 4h, and 4a exhibited comparatively higher energy gaps of 4.5063 eV, 4.4975 eV, 4.4963 eV, 4.4923 eV, and 4.4259 eV respectively, reinforcing their enhanced stability. As shown in Fig. 14(A–E) and S111, molecular orbital analysis of the synthesized compounds 4a–q reveals that the HOMO cloud is primarily localized over the benzopyran and thiazolo-triazole rings, densely covering all ring atoms. In contrast, the LUMO cloud is more dispersed and less concentrated over the substituted 2*H*-chromene and thiazolo-triazole rings, leaving the nitrogen atoms of the triazole ring partially covered. Correlation of the FMO results with the biological data revealed that compounds such as 4f and 4h, which exhibited superior anticancer activity, also possess favorable orbital distributions. In these compounds, the HOMO is predominantly localized over the conjugated benzopyran and thiazolo-triazole framework, promoting electron-donating ability, whereas the LUMO is distributed over electron-deficient regions, facilitating electron acceptance. This distinct spatial separation of frontier orbitals enhances their capacity for charge-transfer interactions with amino acid residues within the protein active site, thereby contributing to their improved biological activity. Moreover, global reactivity descriptors such as chemical hardness (*η*), softness (*S*) and electrophilicity index (*ω*) further support this interpretation. As presented in Table 6, the relationship between hardness and softness plays a crucial role in governing the molecular adaptability of the synthesized compounds. Among the synthesized compounds, compound 4f possesses the highest hardness (*η* = 2.2532 eV), indicating strong resistance to electronic deformation, whereas compound 4m, with the highest softness (*S* = 0.2287 eV^−1^), suggesting greater flexibility in charge transfer interactions. Notably, despite its relatively higher HOMO–LUMO energy gap, compound 4f maintains an optimal balance between stability and electronic adaptability, enabling effective participation in stabilizing non-covalent interactions, as supported by its strong docking score (−10.8 kcal mol^−1^). These findings highlight that an appropriate balance between molecular stability and reactivity is essential for achieving enhanced biological performance. Chemical potential (*µ*) shows the ability of an electron to escape a chemical system,^47^ whereas electronegativity (*χ*) defines the propensity to attract electrons of a molecule.^48^ The synthesized compounds unveiled chemical potentials between −3.9750 eV and −3.4073 eV, suggesting more resistant to electron loss but more susceptible to gain electron. The electrophilicity index, chemical potential, and softness play a significant role in modulating the interaction of synthesized compounds with amino acid residues, thereby facilitating hydrogen bonding, π–π stacking, and other stabilizing non-covalent interactions observed in the docking studies. Among the investigated compounds, 4b shows the highest electronegativity (*χ* = 3.9750 eV), indicating that it has the strongest tendency to accept electrons. Moreover, compound 4b (*ω* = 3.5909 eV and Δ*N* = 1.8068) is more electronegative and electrophilic; as compound having high electrophilicity index (*ω*) and extra electronic charge (Δ*N*) values indicate the strongest tendency to accept electrons.^49^ Conversely, a good nucleophile will have a lower value of electrophilicity index (*ω*) and a higher value of nucleophilicity (*ε*).^50^ Therefore, the most nucleophilic character in 4c is exhibited with the largest value of *ε* = 0.3857 eV^−1^.

**Table 6:** Calculated global electronic properties of synthesized 2*H*-chromene-fused-thiazolo-triazole derivatives 4(a–q)

Compounds	*E*_LUMO_ (eV)	*E*_HOMO_ (eV)	Δ*E*_g_ (eV)	*I* (eV)	*A* (eV)	*χ* (eV)	*µ* (eV)	*η* (eV)	*S* (eV^−1^)	*ω* (eV)	*ε* (eV^−1^)	Δ*N*
4a	−1.5420	−5.9679	4.4259	5.9679	1.5420	3.7550	−3.7550	2.2130	0.2259	3.1857	0.3139	1.6968
4b	−1.7749	−6.1750	4.4001	6.1750	1.7749	3.9750	−3.9750	2.2001	0.2273	3.5909	0.2785	1.8068
4c	−1.1684	−5.6462	4.4778	5.6462	1.1684	3.4073	−3.4073	2.2389	0.2233	2.5927	0.3857	1.5219
4d	−1.7191	−6.1099	4.3908	6.1099	1.7191	3.9145	−3.9145	2.1954	0.2277	3.4899	0.2865	1.7830
4e	−1.2688	−5.7663	4.4975	5.7663	1.2688	3.5176	−3.5176	2.2488	0.2223	2.7511	0.3635	1.5642
4f	−1.3233	−5.8296	4.5063	5.8296	1.3233	3.5765	−3.5765	2.2532	0.2219	2.8385	0.3523	1.5873
4g	−1.7107	−6.0985	4.3878	6.0985	1.7107	3.9046	−3.9046	2.1939	0.2279	3.4746	0.2878	1.7798
4h	−1.2013	−5.6936	4.4923	5.6936	1.2013	3.4475	−3.4475	2.2462	0.2226	2.6456	0.3780	1.5348
4i	−1.2182	−5.7145	4.4963	5.7145	1.2182	3.4664	−3.4664	2.2482	0.2224	2.6723	0.3742	1.5419
4j	−1.7659	−6.1648	4.3989	6.1648	1.7659	3.9654	−3.9654	2.1995	0.2273	3.5745	0.2798	1.8029
4k	−1.5750	−5.9809	4.4059	5.9809	1.5750	3.7780	−3.7780	2.2030	0.2270	3.2395	0.3087	1.7150
4l	−1.7592	−6.1412	4.3820	6.1412	1.7592	3.9502	−3.9502	2.1910	0.2282	3.5609	0.2808	1.8029
4m	−1.4452	−5.8176	4.3724	5.8176	1.4452	3.6314	−3.6314	2.1862	0.2287	3.0160	0.3316	1.6611
4n	−1.5191	−5.9178	4.3987	5.9178	1.5191	3.7185	−3.7185	2.1994	0.2273	3.1434	0.3181	1.6907
4o	−1.6857	−6.0693	4.3836	6.0693	1.6857	3.8775	−3.8775	2.1918	0.2281	3.4298	0.2916	1.7691
4p	−1.5494	−5.9502	4.4008	5.9502	1.5494	3.7498	−3.7498	2.2004	0.2272	3.1951	0.3130	1.7041
4q	−1.5692	−5.9597	4.3905	5.9597	1.5692	3.7645	−3.7645	2.1953	0.2278	3.2277	0.3098	1.7148

#### 3.1.3. Molecular electrostatic potential (MEP) analysis

**Fig. 15:**
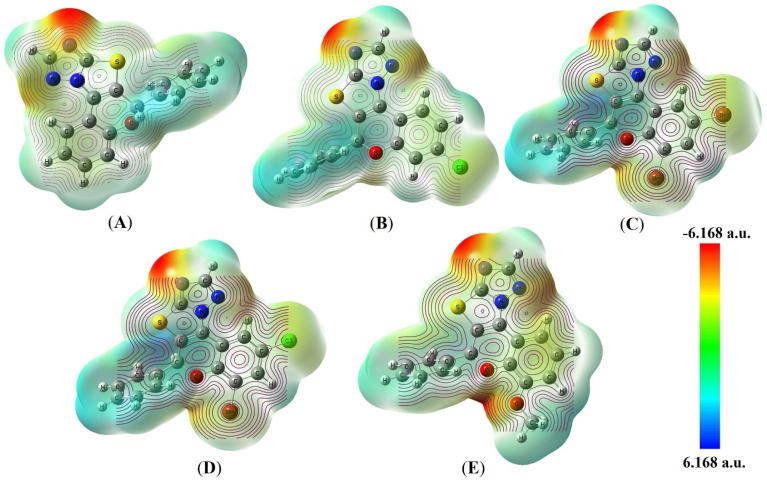
Molecular electrostatic potential (MEP) maps and electrostatic contour plots of the synthesized compounds 4a (A), 4e (B), 4f (C), 4h (D), and 4i (E) generated using the B3LYP/6-311G++(d,p) basis set.

The molecular electrostatic potential (MEP) analysis was conducted to provide deeper insight into the electronic features governing ligand–protein interactions and to elucidate their correlation with the observed biological activities. The MEP surface provides a three-dimensional representation of charge distribution, where red regions (electrophilic) interact with electron-deficient sites on biomolecules, blue color regions (nucleophilic) engage with electron-rich biological sites, and green regions represent neutral sites.^30,34^ This distribution of charge plays a pivotal role in determining molecular reactivity and binding affinity, as these regions directly influence the ability of the molecules to participate in key non-covalent interactions, including hydrogen bonding, electrostatic interactions, and π-type interactions within the active sites of target proteins.^51^ According to the studied results, the MEP maps ranging from −6.168 a.u. to 6.168 a.u. on an electron density iso-surface of 0.0004 a.u. As shown in Fig. 15 and S112, the MEP maps of the synthesized compounds exhibit distinct charge separation, with pronounced negative potential regions localized around heteroatoms such as oxygen and nitrogen, while positive potential regions are primarily distributed over hydrogen atoms and aromatic frameworks. These electrostatic features are directly relevant to intermolecular interactions, as electron-rich regions favor hydrogen bonding and electrostatic interactions with electron-deficient residues of proteins, whereas electron-deficient regions facilitate complementary interactions with nucleophilic amino acid residues.

Importantly, when analyzed in conjunction with molecular docking results, a clear correlation emerges between the MEP profiles and the binding behavior of the compounds. The biologically active derivatives (4a, 4e, 4f, 4h and 4i) exhibit well-defined and spatially accessible negative potential regions that coincide with the formation of strong hydrogen bonds and electrostatic interactions with key residues in both anticancer (1ZXN and 2W3L) and antibacterial (3G7E and 3G7B) protein targets. This is consistent with their higher docking scores and enhanced biological activities observed in cytotoxicity and antimicrobial assays. Furthermore, the balanced distribution of positive and negative electrostatic regions in these active compounds facilitates multiple non-covalent interactions, including π–π stacking and van der Waals interactions, thereby contributing to stable ligand–protein complex formation. Furthermore, the intramolecular charge distribution of the compounds is illustrated through contour mapping, where yellow contours denote regions of positive charge and red contours correspond to negative charge accumulation.^52^ The electrostatic contour lines illustrate charge variations, reinforcing their potential as antibacterial and anticancer agents. The charge distributions support hydrogen bonding as well as electrostatic interactions with target proteins. This electronic feature further supports their suitability as promising bioactive candidates. Conversely, the compounds with weaker biological activity exhibit less pronounced or poorly oriented electrostatic regions, which may limit their ability to establish strong interactions within the active sites.

### 3.2. Drug likeness prediction

#### 3.2.1. Physicochemical and medicinal chemistry properties

The physicochemical and medicinal chemistry properties of five potent compounds (4a, 4e, 4f, 4h and 4i) were evaluated using the SwissADME web tool and compared with the standard drugs Gentamicin and Doxorubicin to obtain preliminary insights into their drug-likeness characteristics (Table 7). These descriptors are commonly employed in early-stage of drug discovery to predict whether compounds fall within the general property space of orally active molecules.^34,53^ Lipinski's rule, Veber's rule, and Egan's rule are three of the most common drug-likeness rules used to predict whether drugs will be orally active prior to clinical trials or *in vivo* studies.^30,54,55^

**Table 7:** Physiochemical properties and medicinal chemistry properties with drug likeness rules of potent compounds 4a, 4e, 4f, 4h, 4i and standard drugs Gentamicin, Doxorubicin calculated using SwissADME web tool^a^

Properties	Compounds					Gentamicin	Doxorubicin
	4a	4e	4f	4h	4i		
**Physicochemical**							
MW	305.35	339.80	463.15	418.69	335.38	477.60	543.52
Csp^3^	0.06	0.06	0.06	0.06	0.11	1.00	0.44
^*n*^RB	1	1	1	1	2	7	5
^*n*^HA	3	3	3	3	4	12	12
^*n*^HD	0	0	0	0	0	8	6
MR	85.32	90.33	100.72	98.03	91.82	118.31	132.66
TPSA	67.66	67.66	67.66	67.66	76.89	199.73	206.07
log *P*	3.62	4.27	5.14	5.03	3.62	−3.33	−0.32
% ABS	85.66%	85.66%	85.66%	85.66%	82.47%	40.09%	37.91%

**Medicinal chemistry**							
SA score	3.82	3.82	3.87	3.85	3.95	6.51	5.81
BA score	0.55	0.55	0.55	0.55	0.55	0.17	0.17
PAINS alerts	0	0	0	0	0	0	1

**Druglikeness rules**							
Lipinski's rule (^*n*^Vio)	Yes, 0	Yes, 0	Yes, 0	Yes, 0	Yes, 0	No, 2	No, 3
Veber's rule (^*n*^Vio)	Yes, 0	Yes, 0	Yes, 0	Yes, 0	Yes, 0	No, 1	No, 1
Egan's rule (^*n*^Vio)	Yes, 0	Yes, 0	Yes, 0	Yes, 0	Yes, 0	No, 1	No, 1

a MW = molecular weight (in g mol^−1^), ^*n*^RB = number of rotatable bonds, ^*n*^HA = number of hydrogen bond acceptors, ^*n*^HD = number of hydrogen bond donors, log *P* = the logarithm of the *n*-octanol/water distribution coefficients at 7.4 pH, TPSA = topological polar surface area (in Å^2^), Csp^3^ = sp^3^ hybridized fraction of carbon atoms in a molecule, MR = molar refractivity (m^3^ mol^−1^), % ABS = percentage of absorption rate, SA score = synthetic accessibility score, BA score = bioavailability score, PAINS = pan assay interference structures, ^*n*^Vio = the number of violations.

The synthesized potent compounds showed molecular weights ranging from 305.35 to 463.15 g mol^−1^, which are within the optimal range for small-molecule drug candidates and also lower than the reference drugs. All evaluated compounds displayed low Csp3 fractions, consistent with aromatic-rich scaffolds and exhibited limited molecular flexibility with 1–2 rotatable bonds, which is valuable for the target binding. In contrast, both standard drugs Gentamicin and Doxorubicin possessed significantly higher flexibility. The number of hydrogen bond acceptor (^*n*^HA = 3–4) and donor (^*n*^HD = 0) for evaluated compounds were fully complied with Lipinski's rule, indicating a favorable balance between polarity and permeability. The topological polar surface area (TPSA) values of the compounds ranged between 67.66–76.89 Å^2^, which were below the threshold associated with good oral absorption, whereas the reference drugs exhibited excessively high TPSA values (>190 Å^2^). The absorption rate of each tested potent molecule was calculated using the formula (% ABS = 109 − (0.345 × TPSA)),^30,34^ and the oral absorption percentages found between 82.47% and 85.66% as compare to standard drugs 40.09% and 37.91%. The medicinal chemistry property prediction results revealed that the synthetic accessibility scores (3.82–3.95) indicate that the potent synthesized compounds are synthetically feasible and less complex than the standard drugs. The potent compounds demonstrated a bioavailability score of 0.55, indicating favorable oral bioavailability, while the standard drugs Gentamicin and Doxorubicin showed poor scores (0.17). Notably, none of the synthesized compounds triggered PAINS alerts and all complied with drug-likeness rules (Lipinski, Veber, and Egan), showing zero violations, which confirms their suitability as reliable biological candidates.

**Fig. 16:**
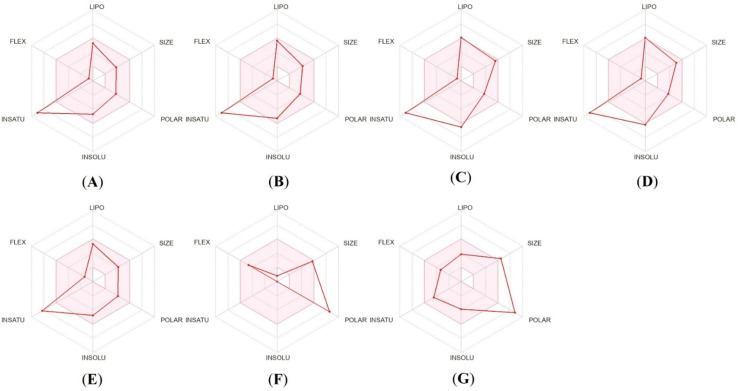
Bioavailability radar plots of most potent compounds 4a (A), 4e (B), 4f (C), 4h (D), 4i (E), Gentamicin (F), and Doxorubicin (G).

The radar chart is intended to illustrate the physiochemical properties that can lead to good oral bioavailability. The optimal range (pink area) of the oral active drug consist of six major variables, namely lipophilicity (LIPO): 0.7 < log *P* < 5.0, SIZE: 150 g mol^−1^ < MW < 500 g mol^−1^, POLAR (polarity): 20 Å^2^ < TPSA < 130 Å^2^, INSOLU (insolubility): 0 < log *S* < 6, INSATU (instauration): 0.25 < fraction Csp^3^ < 1, FLEX (flexibility): 0 < number of rotatable bonds < 9.^30,34,55^ All five potent compounds tested fell slightly beyond the ideal insaturation range and the standard drugs were found to be slightly outside the ideal ranges for polarity (Fig. 16(A–G)).

Overall, the physicochemical evaluation suggests that the synthesized compounds occupy a favorable chemical space for small-molecule design, but these findings should be considered exploratory and require further experimental validation.

### 3.3. ADMET properties

The *in silico* ADMET (Absorption, Distribution, Metabolism, Excretion, and Toxicity) profiles of the selected compounds were predicted using pkCSM and ProTox 3.0 platforms to provide preliminary insights into their efficacy and safety characteristics.^56,57^ Such computational approaches are widely used in early-stage screening; however, their predictive nature necessitates cautious interpretation.

As presented in Table 8, the water solubility was evaluated using log *S* values, and the log *S* < 0 is said to be highly soluble.^58,59^ All potent compounds as well as standard drugs were classified as moderately soluble (−3.386, −3.485, −4.353, −3.806, −3.45), whereas the standard drugs exhibited significantly higher solubility with log *S* values −2.843 and −2.915 respectively, indicating a more favorable solubility profile. The Caco-2 monolayer permeability model simulates intestinal transport.^60,61^ For this Caco-2 permeability, values above 0.90 indicate high permeability of the compound.^61^ Here, all the potent compounds demonstrated high intestinal permeability. All potent compounds exhibited high skin permeability (log *K*_p_ between −2.734 and −2.666), similar to Gentamicin (−2.735), and Doxorubicin (−2.735). All compounds exhibited excellent predicted intestinal absorption (>96%), indicating strong oral bioavailability, whereas none were identified as *P*-glycoprotein substrates. The compounds showed moderate volume of distribution and acceptable plasma protein binding, with positive BBB permeability values suggesting potential CNS accessibility, unlike the reference drugs. Metabolic predictions indicated CYP3A4 substrate behavior and inhibition of selected CYP isoforms, highlighting possible drug–drug interaction risks that indicate the need of further optimization. The toxicity profiling demonstrated non-inhibition of hERG I for all compounds, acceptable maximum tolerated dose values, and low acute and chronic toxicity. The compounds 4a and 4f exhibited LD_50_ values of 1000 mg kg^−1^ and 4e, 4h, and 4i at 600 mg kg^−1^, all falling under toxicity class 4 (Fig. S113(A–G)). In comparison, Gentamicin displayed lower toxicity (LD_50_ = 5000 mg kg^−1^, class 5), whereas Doxorubicin showed higher toxicity (LD_50_ = 205 mg kg^−1^, class 3), consistent with their known safety profiles (Fig. S112(A–G)). Overall, the synthesized compounds demonstrate acceptable acute toxicity and are less toxic than Doxorubicin, supporting their suitability for further biological investigation. Notably, compounds 4e and 4f were non-mutagenic and non-hepatotoxic, and were identified them as the most promising leads. Collectively, the ADMET predictions indicate favorable drug-likeness for the synthesized compounds (particularly 4e and 4f) which are promising candidates for further experimental studies.

**Table 8:** *In silico* ADMET parameters of potent compounds 4a, 4e, 4f, 4h, 4i and standard drugs Gentamicin, Doxorubicin obtained using pkCSM and ProTox 3.0 platforms^a^

ADMET parameters		Compounds					Gentamicin	Doxorubicin	Unit
		4a	4e	4f	4h	4i			
Absorption	Water solubility	−3.386	−3.485	−4.353	−3.806	−3.45	−2.843	−2.915	Numeric (log mol L^−1^)
	Caco-2 permeability	1.42	1.548	1.075	1.095	1.403	0.979	0.457	Numeric (log *P*_app_ in 10^−6^ cm s^−1^)
	Intestinal absorption	99.057	96.397	96.568	96.376	100	19.161	62.372	Numeric (% absorbed)
	Skin permeability	−2.719	−2.734	−2.666	−2.724	−2.734	−2.735	−2.735	Numeric (log *K*_p_)
	*P*-glycoprotein substrate	No	No	No	No	No	Yes	Yes	Categorical (yes/no)
	*P*-glycoprotein I inhibitor	No	No	Yes	No	No	No	No	Categorical (yes/no)
	*P*-glycoprotein II inhibitor	Yes	Yes	Yes	Yes	Yes	No	No	Categorical (yes/no)
Distribution	VDss	0.305	0.2	0.568	0.516	0.242	−1.313	1.647	Numeric (log L kg^−1^)
	Fraction unbound	0.28	0.254	0.182	0.197	0.235	0.744	0.215	Numeric (Fu)
	BBB permeability	0.631	0.411	0.406	0.387	0.322	−0.851	−1.379	Numeric (log BB)
	CNS permeability	−1.46	−1.438	−1.431	−1.446	−1.543	−4.093	−4.307	Numeric (log PS)
Metabolism	CYP2D6 substrate	No	No	No	No	No	No	No	Categorical (yes/no)
	CYP3A4 substrate	Yes	Yes	Yes	Yes	Yes	No	No	Categorical (yes/no)
	CYP1A2 inhibitor	Yes	Yes	Yes	Yes	Yes	No	No	Categorical (yes/no)
	CYP2C19 inhibitor	Yes	Yes	Yes	Yes	Yes	No	No	Categorical (yes/no)
	CYP2C9 inhibitor	Yes	Yes	Yes	Yes	Yes	No	No	Categorical (yes/no)
	CYP2D6 inhibitor	No	No	No	No	No	No	No	Categorical (yes/no)
	CYP3A4 inhibitor	No	Yes	Yes	Yes	Yes	No	No	Categorical (yes/no)
Excretion	Total clearance	0.227	0.134	0.039	0.068	0.336	0.708	0.987	Numeric (log mL min^−1^ kg^−1^)
	Renal OCT2 substrate	Yes	No	Yes	No	No	No	No	Categorical (yes/no)
Toxicity	AMES toxicity	Yes	No	No	Yes	Yes	No	No	Categorical (yes/no)
	Max. tolerated dose (human)	0.288	0.559	−0.317	0.251	0.45	0.188	0.081	Numeric (log mg kg^−1^ day^−1^)
	hERG I inhibitor	No	No	No	No	No	No	No	Categorical (yes/no)
	hERG II inhibitor	Yes	Yes	No	Yes	Yes	No	Yes	Categorical (yes/no)
	Oral rat acute toxicity	2.301	2.475	2.634	2.54	2.643	2.559	2.408	Numeric (mol kg^−1^)
	Oral rat chronic toxicity	0.752	0.351	0.713	0.557	0.728	2.763	3.339	Numeric (log mg kg_bw_^−1^ day^−1^)
	Hepatotoxicity	Yes	Yes	No	No	Yes	No	Yes	Categorical (yes/no)
	Skin sensitisation	No	No	No	No	No	No	No	Categorical (yes/no)
	*T. pyriformis* toxicity	0.296	0.287	0.298	0.29	0.287	0.285	0.285	Numeric (log µg L^−1^)
	Minnow toxicity	0.538	−0.364	1.547	1.022	−0.086	6.242	4.412	Numeric (log mM)

a *P*_app_: apparent permeability, *P*gp: *P*-glycoprotein, BBB: blood–brain barrier, CYP: cytochrome P450, OCT2: organic cation transporter 2, hERG: human ether-a-go-go related gene.

In summary, the predicted ADMET profiles suggest that comprehensive experimental validation is essential to confirm these findings and to establish the true therapeutic potential of these compounds.

## 4. Conclusion

In summary, we have successfully developed a method for the synthesis of 2*H*-chromene-fused-thiazolo-triazole derivatives 4(a–r) with good to excellent yields, following a multicomponent protocol. The synthesized compounds were characterized by ^1^H NMR, ^13^C NMR, HRMS and single-crystal X-ray diffraction. The synthesized derivatives were evaluated against MCF-7, MDA-MB-231, and A549 cancer cell lines, wherein superior activity was observed in MCF 7 cells. Notably, compounds 4f, 4h and 4i showed most potent inhibitory activity in comparison to Doxorubicin with IC_50_ values of 1.73 ± 0.24 µM, 5.47 ± 0.72 µM and 8.12 ± 0.84 µM, respectively. The *in vitro* antibacterial activity of synthesized compounds was evaluated against *S. aureus* and *E. coli*. Among these, compounds 4a, 4e, and 4i exhibited the highest potency against both bacterial strains compared to the standard drug, Gentamicin. Compound 4i exhibited the highest bacterial inhibition against both bacterial strains, with an MIC of 10 µg mL^−1^ and a ZI of 19 mm. DFT calculations provided insights into the electronic properties, with *E*_LUMO_ values ranging from −1.7749 eV to −1.1684 eV, *E*_HOMO_ values from −6.1750 eV to −5.6462 eV, and Δ*E*_g_ values from 4.3724 eV to 4.5063 eV. ADMET profiling supported the drug likeness of the most active molecules. Overall, compound 4i emerged as a promising dual inhibitor with both anticancer and antimicrobial potential, whereas compounds 4a and 4e may serve as potent antibacterial leads and compounds 4f and 4h as selective breast cancer agents. Moreover, we anticipate that further research will lead to the development of more potent druggable agents.

## Author contributions

Barsha Samanta: conceptualization, investigation, methodology, writing-original draft, writing-review & editing, formal analysis, data curation, software. Tapaswini Pati: formal analysis, validation. Ananya Dash: formal analysis, validation. Bhabani Shankar Panda: software, writing-review & editing, software, formal analysis, validation. Eeshara Naik: methodology, formal analysis. Seetaram Mohapatra: conceptualization, supervision, project administration, funding acquisition, resources, writing-review & editing, validation. Chita Ranjan Sahoo: resources, methodology, validation. Pradeep Kumar Naik: validation, resources, methodology.

## Conflicts of interest

The authors declare no conflict of interest.

## Supplementary Material

RA-016-D6RA03687B-s001

RA-016-D6RA03687B-s002

## Data Availability

The X-ray crystallographic data of compound 4j can be obtained from Cambridge Structural Database (CCDC deposition number: 2309815, DOI: 10.5517/ccdc.csd.cc2hjk5s).^62^ All the findings or data analysed during this study are included in this article and the Supplementary Information (SI). Supplementary information: the ^1^H NMR spectra, ^13^C NMR spectra, HRMS data and *in silico* studies data of all synthesized compounds 4(a–r). See DOI: https://doi.org/10.1039/d6ra03687b.
