# Supplementary material for: Multicomponent synthesis of 2H-chromene-fused-thiazolo-triazole derivatives via cascade Michael addition/cyclization reaction: anticancer, antibacterial and computational evaluations
Source: RSC Adv. 2026 May 21;16(30):27374–96. doi: 10.1039/d6ra03687b (PMC13195423; doi:10.1039/d6ra03687b)
Supplement: RA-016-D6RA03687B-s001 [file RA-016-D6RA03687B-s001.pdf]

Supplementary Information (SI) for RSC Advances.
This journal is © The Royal Society of Chemistry 2026

SUPPORTING INFORMATION

Multicomponent Synthesis of 2*H*-chromene-fused-thiazolo-triazole derivatives *via* Cascade Michael addition/Cyclization Reaction: Anticancer, Antibacterial and Computational Evaluations

Barsha Samanta[1], Tapaswini Pati[1], Ananya Dash[1], Bhabani Shankar Panda[1], Eeshara Naik[2], Seetaram Mohapatra[1]*, Chita Ranjan Sahoo[3], Pradeep Kumar Naik[2]

[1]*Organic Synthesis Laboratory, Department of Chemistry, Ravenshaw University, Cuttack-753003, Odisha, India*
[2]*Centre of Excellence in Natural Products and Therapeutic, Department of Biotechnology and Bioinformatics, Sambalpur University, Jyoti Vihar, Burla, Sambalpur 768019, Odisha, India*
[3]*ICMR-Regional Medical Research Centre, Department of Health Research, Ministry of Health & Family Welfare, Govt. of India, Bhubaneswar-751023, Odisha, India*

**Corresponding Author*
Email: seetaram.mohapatra@gmail.com

Sl. No.	Contents	Page No.
1	General information	1-1
2	Synthetic procedure of compounds	1-7
3	Experimental: ^{1}H NMR, ^{13}C NMR and HRMS Spectra of 2*H*-chromene-fused-thiazolo-triazole derivatives **4(a-r)**	7-62
4	Biological Activity Evaluations	63-64
5	Computational Studies	64-101

1. General information

All chemicals and solvents were procured from commercial suppliers. DABCO, salicylaldehyde, trans-*β*-nitrostyrene, and 1,2,4-triazole-3-thiol were purchased from Sigma-Aldrich and used without further purification. The progress of the reaction was monitored by Thin Layer Chromatography (TLC) performed on silica gel aluminium plates, and visualization was done by UV light. ^{1}H NMR and ^{13}C NMR spectra were recorded at 400 MHz and 100 MHz, respectively, with TMS as an internal standard. Chemical shifts (δ) are reported in parts per million (ppm), downfield from the internal standard (TMS, δ = 0.00 ppm) relative to residual $CHCl_3$ (^{1}H: δ = 7.26 ppm, ^{13}C: δ = 77.00 ppm) as an internal reference. Coupling constants (*J*) are reported in Hertz (Hz). The abbreviations used in reporting ^{1}H NMR data were denoted as follows: s, singlet; dd, double doublet; d, doublet; t, triplet; m, multiplet. The coupling constant values (*J*) were recorded in hertz (Hz). The melting points were measured with an electrothermal (Stuart SPM10) apparatus. High-resolution mass Spectra (HRMS) were recorded using the Bruker microTOF-QII mass spectrometer model at the laboratory of IISER, Berhampur.

2. Synthetic procedure for the synthesis of 2*H*-chromene-fused-thiazolo-triazole derivatives 4(a-r)

A mixture of substituted salicylaldehyde (1a-l) and trans-*β*-nitrostyrene (2a-c) was placed in a sterile, oven-dried sealed tube containing THF. The reaction mixture was heated at 90 °C for 15 min. Subsequently, 1,2,4-triazole-3-thiol (3) was added, and the mixture was stirred for 1 h. The progress of the reaction was monitored by TLC. The reaction mixture was diluted with water upon completion, and the product was extracted with ethyl acetate. The organic layer was separated, dried over anhydrous Na_2SO_4, and evaporated under reduced pressure. The impure product was then refined via standard silica gel (100-200 mesh) column chromatography, employing a mixture of ethyl acetate and hexane, generating the desired 2*H*-chromene-fused-thiazolo-triazole derivatives **4(a-r)** with a good to excellent yield of 75%-92%.

2.1. *6-phenyl-6H-chromeno[4',3':4,5]thiazolo[3,2-b][1,2,4]triazole* [**4a**]

White Solid (89%, 220 mg); Melting point: 217-218 °C; ^{1}H NMR (400 MHz, $CDCl_3$): δ_H (ppm) 8.37(dd, J_{12}=2 Hz, J_{13}=8 Hz, 1H), 8.24(s, 1H), 7.55-7.45(m, 5H), 7.35-7.32(m, 1H), 7.18-7.14(m, 1H), 7.05(d, *J*=8 Hz, 1H), 6.54(s, 1H); ^{13}C NMR (100 MHz, $CDCl_3$): δ_C (ppm) 160.0, 155.7, 152.4, 137.7, 131.0, 129.9, 129.1, 127.4, 124.9, 123.0, 122.5, 120.0, 117.0, 114.2, 77.3; HRMS (ESI) calculated for $C_{17}H_{11}N_3OS$ $[M+H]^+$ 306.0701, found 306.0710

2.2. *2-bromo-6-phenyl-6H-chromeno[4',3':4,5]thiazolo[3,2-b][1,2,4]triazole* [**4b**]

White Solid (91%, 174 mg); Melting point: 200-201 °C; ^{1}H NMR (400 MHz, $CDCl_3$): δ_H (ppm) 8.50(d, *J*= 2 Hz, 1H), 8.25(s, 1H), 7.52-7.40(m, 6H), 6.92(d, *J*= 8.8 Hz, 1H), 6.54(s, 1H); ^{13}C NMR (100 MHz, $CDCl_3$): δ_C (ppm) 155.8, 151.3, 137.3, 133.6, 130.1, 129.2, 127.4, 125.6, 123.7, 121.1, 118.7, 115.6, 114.8, 77.5; HRMS (ESI) calculated for $C_{17}H_{10}BrN_3OS$ $[M+H]^+$ 383.9806, found 383.9816

2.3. *3-bromo-6-phenyl-6H-chromeno[4',3':4,5]thiazolo[3,2-b][1,2,4]triazole* [**4c**]

Yellow Solid (88%, 166 mg); Melting point: 221-222 °C; ^{1}H NMR (400 MHz, $CDCl_3$): δ_H (ppm) 8.24(d, *J*= 8.8 Hz, 2H), 7.51-7.45(m, 5H), 7.29(dd, J_{12}= 1.6 Hz, J_{13}= 8 Hz, 1H) 7.21(d, *J*= 2 Hz, 1H), 6.54(s, 1H); ^{13}C NMR (100 MHz, $CDCl_3$): δ_C (ppm) 156.9, 155.8, 152.9, 137.3, 130.1, 129.2, 127.3, 125.6, 124.2, 124.0, 124.0, 120.4, 120.0, 113.0, 77.6; HRMS (ESI) calculated for $C_{17}H_{10}BrN_3OS$ $[M+H]^+$ 383.9806, found 383.9803

2.4. *2-chloro-6-phenyl-6H-chromeno[4',3':4,5]thiazolo[3,2-b][1,2,4]triazole* [**4d**]

White solid (87%, 189 mg); Melting point: 214-215 °C; ^{1}H NMR (400 MHz, $CDCl_3$): δ_H (ppm) 8.37(d, *J*= 2 Hz, 1H), 8.24(s, 1H), 7.52-7.50(m, 3H), 7.49-7.45(m, 3H), 6.97(d, *J*= 8.4 Hz, 1H), 6.53(s, 1H); ^{13}C NMR (100 MHz, $CDCl_3$): δ_C (ppm) 156.8, 155.9, 150.8, 137.3, 130.6, 130.1, 129.2, 127.6, 127.4, 123.9, 122.8, 121.2, 118.3, 115.2, 77.5; HRMS (ESI) calculated for $C_{17}H_{10}ClN_3OS$ $[M+H]^+$ 340.0311, found 340.0314

2.5. *3-chloro-6-phenyl-6H-chromeno[4',3':4,5]thiazolo[3,2-b][1,2,4]triazole* [**4e**]

Yellow Solid (85%, 184 mg); Melting point: 213-214 °C; ^{1}H NMR (400 MHz, $CDCl_3$): δ_H (ppm) 8.32(d, *J*= 8.4 Hz, 1H), 8.23(s, 1H), 7.51-7.44(m, 5H), 7.23(dd, J_{12}= 2 Hz, J_{13}= 8.4 Hz, 1H), 7.05(d, *J*= 2 Hz, 1H), 6.55(s, 1H); ^{13}C NMR (100 MHz, $CDCl_3$): δ_C (ppm) 156.9, 155.8, 153.0, 137.4, 136.2, 130.1, 129.2, 127.4, 124.2, 123.8, 122.7, 119.8, 117.5, 112.7, 77.6; HRMS (ESI) calculated for $C_{17}H_{10}ClN_3OS$ $[M+H]^+$ 340.0311, found 340.0309

2.6. *2,4-dibromo-6-phenyl-6H-chromeno[4',3':4,5]thiazolo[3,2-b][1,2,4]triazole* [**4f**]

White Solid (92%, 152 mg); Melting point: 238-239 °C; ^{1}H NMR (400 MHz, $CDCl_3$): δ_H (ppm) 8.48(d, *J*= 2.4 Hz, 1H), 8.24(s, 1H), 7.67(d, *J*= 2.4 Hz, 1H), 7.51-7.43(m, 5H), 6.65(s, 1H); ^{13}C NMR (100 MHz, $CDCl_3$): δ_C (ppm) 156.8, 156.0, 148.3, 137.0, 136.3, 130.0, 129.2, 127.1, 124.7, 123.1, 121.7, 116.4, 114.8, 111.8, 77.7; HRMS (ESI) calculated for $C_{17}H_9Br_2N_3OS$ $[M+H]^+$ 463.8889, found 463.8888

2.7. *2,4-dichloro-6-phenyl-6H-chromeno[4',3':4,5]thiazolo[3,2-b][1,2,4]triazole* [**4g**]

White solid (86%, 168 mg); Melting point: 218-219 °C; ^{1}H NMR (400 MHz, $CDCl_3$): δ_H (ppm) 8.31(d, J= 2.4 Hz, 1H), 8.25(s, 1H), 7.52-7.49(m, 2H), 7.48-7.45(m, 3H), 7.38(d, J= 2.4 Hz, 1H), 6.65(s, 1H); ^{13}C NMR (100 MHz, $CDCl_3$): δ_C (ppm) 156.0, 136.9, 130.9, 130.1, 129.2, 127.6, 127.1, 123.3, 123.0, 121.8, 121.3, 116.1, 77.7; HRMS (ESI) calculated for $C_{17}H_9Cl_2N_3OS$ [M +H]$^+$ 373.9922, found 373.9933

2.8. *4-bromo-2-chloro-6-phenyl-6H-chromeno[4',3':4,5]thiazolo[3,2-b][1,2,4]triazole* [**4h**]

White Solid (81%, 142 mg); Melting point: 206-207 °C; ^{1}H NMR (400 MHz, $CDCl_3$): δ_H (ppm) 8.35(d, J_{12}= 2.4 Hz, 1H), 8.25(s, 1H), 7.54(d, J= 2.4 Hz, 1H), 7.52-7.45(m, 5H), 6.65(s, 1H); ^{13}C NMR (100 MHz, $CDCl_3$): δ_C (ppm) 156.0, 147.8, 136.9, 133.6, 130.0, 129.2, 128.0, 127.1, 123.3, 121.9, 121.8, 116.0, 111.5, 77.7; HRMS (ESI) calculated for $C_{17}H_9BrClN_3OS$ [M +H]$^+$ 419.9396, found 419.9388

2.9. *4-methoxy-6-phenyl-6H-chromeno[4',3':4,5]thiazolo[3,2-b][1,2,4]triazole* [**4i**]

Yellow Solid (79%, 172 mg); Melting point: 193-194 °C; ^{1}H NMR (400 MHz, $CDCl_3$): δ_H (ppm) 8.23(s, 1H), 8.01(d, J= 7.2 Hz, 1H), 7.55-7.73(m, 2H), 7.43(t, J= 4 Hz, 3H), 7.12(t, J= 8 Hz, 1H), 7.00-6.98(m, 1H), 6.57(s, 1H), 3.88(s, 3H); ^{13}C NMR (100 MHz, $CDCl_3$): δ_C (ppm) 155.6, 148.5, 141.5, 137.7, 129.8, 129.1, 127.4, 124.9, 124.7, 122,4, 120.2, 115.1, 115.0, 114.1, 77.3, 56.2; HRMS (ESI) calculated for $C_{18}H_{13}N_3O_2S$ [M +H]$^+$ 336.0807, found 336.0808

2.10. *4-ethoxy-6-phenyl-6H-chromeno[4',3':4,5]thiazolo[3,2-b][1,2,4]triazole* [**4j**]

Orangish-yellow solid (84%, 176 mg); Melting point: 170-171 °C; ^{1}H NMR (400 MHz, $CDCl_3$): δ_H (ppm) 8.22(s, 1H), 8.00(dd, J_{12}= 1.6 Hz, J_{13}= 7.6 Hz, 1H), 7.56-7.51(m, 2H), 7.44-7.39(m, 3H), 7.08(t, J= 8.4 Hz, 1H), 6.98(dd, J_{12}= 1.6 Hz, J_{13}= 8.4 Hz, 1H), 6.56(s, 1H), 4.15-4.07(m, 2H), 1.39(t, J= 7.2 Hz, 3H); ^{13}C NMR (100 MHz, $CDCl_3$): δ_C (ppm) 155.7, 147.9, 142.0, 137.9, 129.7, 129.1, 127.3, 125.1, 122.5, 120.1, 116.4, 115.4, 115.3, 77.1, 65.2, 14.9; HRMS (ESI) calculated for $C_{19}H_{15}N_3O_2S$ [M +H]$^+$ 350.0963, found 350.0978

2.11. *2-bromo-4-methoxy-6-phenyl-6H-chromeno[4',3':4,5]thiazolo[3,2-b][1,2,4]triazole* [**4k**]

Light yellow Solid (83%, 148 mg); Melting point: 226-227 °C; ^{1}H NMR (400 MHz, $CDCl_3$): δ_H (ppm) 8.23(s, 1H), 8.17(d, J= 2.4 Hz, 1H), 7.53(m, 2H), 7.48(m, 3H), 7.09(d, J= 2 Hz, 1H), 6.56(s, 1H), 3.87(s, 3H); ^{13}C NMR (100 MHz, $CDCl_3$): δ_C (ppm) 156.7, 155.8, 149.2, 140.6, 137.3, 130.0, 129.2, 127.4, 121.2, 117.6, 117.2, 116.0, 114.6, 77.5, 56.5; HRMS (ESI) calculated for $C_{18}H_{12}BrN_3O_2S$ [M +H]$^+$ 413.9912, found 413.9912

2.12. *2,4-dichloro-6-(4-methoxyphenyl)-6H-chromeno[4',3':4,5]thiazolo[3,2-b][1,2,4]triazole* [**4l**]

Yellow Solid (80%, 168 mg); Melting point: 203-204 °C; ^{1}H NMR (400 MHz, $CDCl_3$): δ_H (ppm) 8.29(d, *J*= 2.4 Hz, 1H), 8.25(s, 1H), 7.44-7.40(m, 2H), 7.36 (d, *J*=2.8 Hz, 1H), 6.97-6.94(m, 2H), 6.59(s, 1H), 3.84(s, 3H); ^{13}C NMR (100 MHz, $CDCl_3$): δ_C (ppm) 160.9, 156.7, 155.9, 146.8, 130.8, 129.0, 128.8, 127.4, 123.4, 123.0, 122.2, 121.2, 116.1, 114.5, 77.5, 55.4; HRMS (ESI) calculated for $C_{18}H_{11}Cl_2N_3O_2S$ $[M+H]^+$ 404.0027, found 404.0029

2.13. *4-bromo-2-chloro-6-(4-methoxyphenyl)-6H-chromeno[4',3':4,5]thiazolo[3,2-b][1,2,4]triazole* [**4m**]

Yellow Solid (82%, 154 mg); Melting point: 196-197 °C; ^{1}H NMR (400 MHz, $CDCl_3$): δ_H (ppm) 8.34(d, *J*=2 Hz, 1H), 8.25(s, 1H), 7.52(d, *J*=2.4 Hz, 1H), 7.44-7.40(m, 2H), 6.97-6.94(m, 2H), 6.60(s, 1H), 3.84(s, 3H); ^{13}C NMR (100 MHz, $CDCl_3$): δ_C (ppm) 160.9, 156.8, 155.9, 147.8, 133.5, 129.0, 128.8, 127.8, 123.4, 122.1, 121.8, 116.0, 114.5, 111.4, 77.5, 55.4; HRMS (ESI) calculated for $C_{18}H_{11}BrClN_3O_2S$ $[M+H]^+$ 447.9522, found 447.9518

2.14. *2-chloro-6-(4-methoxyphenyl)-6H-chromeno[4',3':4,5]thiazolo[3,2-b][1,2,4]triazole* [**4n**]

Yellow Solid (86%, 200 mg); Melting point: 205-206 °C; ^{1}H NMR (400 MHz, $CDCl_3$): δ_H (ppm) 8.36(d, *J*=2.4 Hz, 1H), 8.25(s, 1H), 7.45-7.41(m, 2H), 7.27-7.24(m, 1H), 6.98-6.93(m, 3H), 6.48(s, 1H), 3.84(s, 3H); ^{13}C NMR (100 MHz, $CDCl_3$): δ_C (ppm) 160.9, 156.8, 155.8, 150.9, 130.6, 129.2, 129.1, 127.5, 124.0, 122.7, 121.5 118.3, 115.2, 114.5, 77.3, 55.4; HRMS (ESI) calculated for $C_{18}H_{12}ClN_3O_2S$ $[M+H]^+$ 370.0417, found 370.0413

2.15. *6-(4-methoxyphenyl)-6H-chromeno[4',3':4,5]thiazolo[3,2-b][1,2,4]triazole* [**4o**]

Orangish-yellow solid (78%, 212 mg); Melting point: 186-187 °C; ^{1}H NMR (400 MHz, $CDCl_3$): δ_H (ppm) 8.36(dd, J_{12}=2 Hz, J_{13}=8 Hz, 1H), 8.24(s, 1H), 7.48-7.44(m, 2H), 7.33-7.29(m, 1H), 7.17-7.13(m, 1H), 7.02(dd, J_{12}=1.2 Hz, J_{13}=8.4 Hz, 1H), 6.98-6.95(m, 2H), 6.48(s, 1H), 3.84(s, 3H); ^{13}C NMR (100 MHz, $CDCl_3$): δ_C (ppm) 160.8, 156.9, 155.6, 152.5, 130.9, 129.7, 129.2, 125.0, 122.9, 122.4, 120.3, 117.0, 114.4, 114.2, 77.1, 55.4; HRMS (ESI) calculated for $C_{18}H_{13}N_3O_2S$ $[M+H]^+$ 336.0807, found 336.0823

2.16. *4-methoxy-6-(4-methoxyphenyl)-6H-chromeno[4',3':4,5]thiazolo[3,2-b][1,2,4]triazole* [**4p**]

Yellow Solid (75%, 178 mg); Melting point: 199-200 °C; ^{1}H NMR (400 MHz, $CDCl_3$): δ_H (ppm) 8.23(s, 1H), 8.00(dd, J_{12}= 1.2 Hz, J_{13}= 7.6 Hz, 1H), 7.48-7.44(m, 2H), 7.11(t, *J*=8 Hz, 1H), 6.99-6.92(m, 3H), 6.52(s, 1H), 3.86(s, 3H), 3.83(s, 3H); ^{13}C NMR (100 MHz, $CDCl_3$): δ_C (ppm) 160.7, 156.9, 155.5, 153.8, 148.5, 141.5, 129.6, 129.2, 125.0, 122.3, 120.6, 115.0, 114.4, 114.0, 77.3, 56.2, 55.3; HRMS (ESI) calculated for $C_{19}H_{15}N_3O_3S$ $[M+H]^+$ 366.0912, found 366.0922

2.17. *2-bromo-6-(4-chlorophenyl)-6H-chromeno[4',3':4,5]thiazolo[3,2-b][1,2,4]triazole* [**4q**]

White Solid (85%, 174 mg); Melting point: 211-212 °C; ^{1}H NMR (400 MHz, $CDCl_3$): δ_H (ppm) 8.49(d, *J*= 2.4 Hz, 1H), 8.25(s, 1H), 7.46-7.40(m, 5H), 6.90(d, *J*=8.8 Hz, 1H), 6.51(s, 1H); ^{13}C NMR (100 MHz, $CDCl_3$): δ_C (ppm) 156.7, 155.9, 151.0, 136.1, 135.7, 133.7, 129.5, 128.8, 125.6, 123.8, 120.3, 118.7, 115.5, 115.0, 76.7; HRMS (ESI) calculated for $C_{17}H_9BrClN_3OS$ $[M +H]^+$ 417.9416, found 417.9409

2.18. *2-nitro-6-phenyl-6H-chromeno[4',3':4,5]thiazolo[2,3-c][1,2,4]triazole* [**4r**]

Yellow solid (76%, 159 mg); Melting point: 208-209 °C; ^{1}H NMR (400 MHz, $CDCl_3$): δ_H (ppm) 8.13(s, 1H), 7.42-7.40(m, 5H), 7.21-7.19(m, 3H), 6.69(s, 1H); ^{13}C NMR (100 MHz, $CDCl_3$): δ_C (ppm) 157.5, 156.1, 152.9, 152.7, 151.8, 144.7, 144.5, 136.8, 129.4, 129.2, 129.1, 127.4, 127.1, 62.6; HRMS (ESI) calculated for $C_{17}H_{10}N_4O_3S$ $[M +H]^+$ 351.0507, found 351.0548

2.19. *Structure Elucidation*

The final Michael addition/cyclization product **4a** was characterized using ^{1}H NMR, ^{13}C NMR, and HRMS spectral data. In the ^{1}H NMR spectra, product **4a** showed a sharp singlet at δ 8.24 ppm, corresponding to the H_b proton of the 1,2,4-triazole-3-thiol moiety **3**. The characteristic singlet for the H_a proton appeared at δ 6.54 ppm, indicating the presence of a double bond in the 2-phenyl-3-nitro-2*H*-chromene scaffold and the formation of the thiazole moiety by cyclization. Additionally, a double doublet for the H_c proton was observed at δ 8.37 ppm with J_{12} = 2 Hz and J_{13} = 8 Hz. The remaining eight protons had chemical shifts between δ 7.55-7.05 ppm. In the ^{13}C NMR spectra, a notable peak at δ 152.4 ppm for the C_4 carbon and δ 120.0 ppm for the C_3 carbon indicated the formation of C-N and C-S bonds between the 1,2,4-triazole and 2*H*-chromene rings *via* cascade Michael addition/cyclization. Similarly, the characteristic C_2 carbon peak of 2-phenyl-3-nitro-2*H*-chromene appeared at δ 77.3 ppm. Additionally, the 1,2,4-triazole and thiazole rings, containing two carbon atoms (C_{13} and C_{15}), exhibited peaks at δ 155.7 ppm and δ 137.7 ppm, respectively. The signals at δ 114.2 ppm, 160.0 ppm, and 124.9 ppm corresponded to the quaternary carbons C_9, C_{10}, and C_{17}, respectively. All the protons and carbons of the synthesized compound **4a** were identified in their respective positions (**Fig. S1**(**A**)). Additionally, the formation structure of compound **4a** was validated by HRMS spectral data, which showed an experimental $[M + H]^+$ value of 306.0710, matching the calculated value of 306.0701.

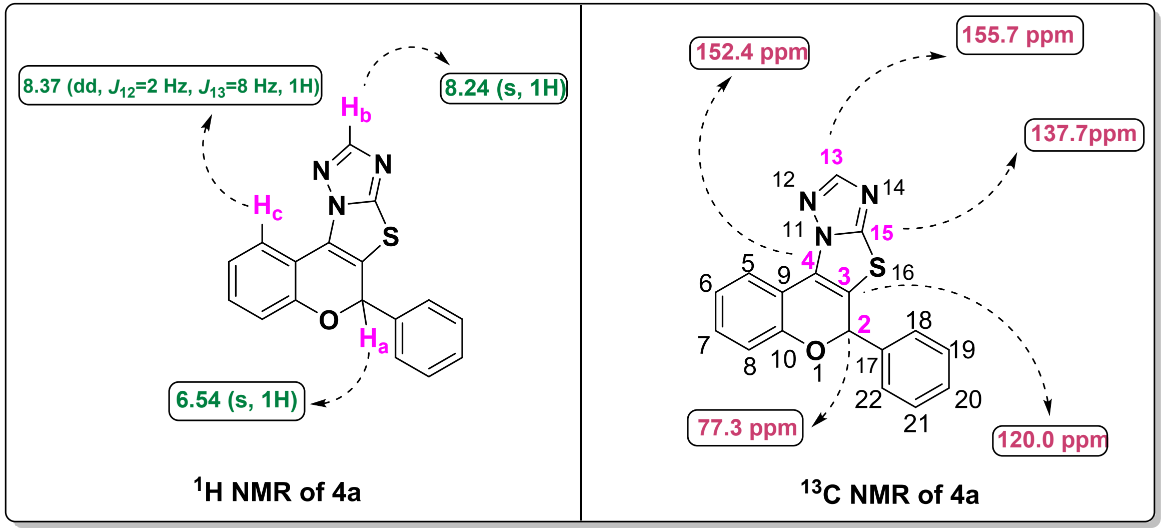

Figure S1(A). ^{1}H and ^{13}C NMR data of synthesized compound **4a**.

2.20. X-ray crystallographic studies

2.20.1. Crystal growth development

The pure single crystal of compound **4j** was grown at room temperature using the slow evaporation or vapor diffusion method from a solvent mixture of acetone and hexane in a 1:2 ratio.

2.20.2. Single crystal data collection, data reduction, structure solution, and structure refinement

A high-quality light red single crystal was selected using a thin glass fiber loop with parabar oil (adhesive) and mounted on a Rigaku XtaLab-II Super-Nova single-crystal X-ray diffractometer with a misfocused copper K*α* X-ray source and a graphite monochromator. The intensity data were recorded at 300.15 K using graphite-monochromatized copper K*α* radiation (λ = 1.54184 Å). Data collection was performed at room temperature in *ω* scan mode to account for Lorentz-polarization and absorption effects. A total of 14,156 reflections were measured experimentally, of which 3397 were treated as independent reflections. Data collection, reduction, and absorption correction were performed using the CrysAlisPro software.[1] The structure of the compound was solved via SHELXT in the intrinsic phase method using the OLEX2 software.[2] Empirical absorption corrections were

done using spherical harmonics, implemented in the SCALE3 ABSPACK scaling algorithm.[4] All the assigned atoms are refined with SHELXL.[2] Again, the SHELXTL-PLUS package of programs is used for Full-matrix least-squares structure refinement against $|F^2|$.[5] The hydrogen atoms are added to the non-hydrogen atoms and held in a riding mode. Finally, anisotropic thermal parameters for non-hydrogen atoms and isotropic thermal parameters for hydrogen atoms are employed. The crystallographic data and refinement parameters of compound **4j** are listed in Table S1.

Table S1. Crystallographic data and refinement parameters of compound **4j**.

**Parameter**	**Compound 4j**
CCDC No.	2309815
Empirical formula	$C_{19}H_{15}N_3O_2S$
Formula weight [g/mol]	349.40
Temperature [K]	300.15
Crystal system	Monoclinic
Space group	$P2_1/c$
Cell lengths (a, b, c) [Å]	17.4745 (5), 4.70452 (12), 20.9252(7)
Cell angle (α, β, γ) [°]	90, 105.964 (4), 90
Cell volume (V) [Å^3]	1653.90 (9)
T_{min}, T_{max}	0.762, 0.828
Z	4
ρ_{calc} [g/cm^3]	1.403
μ [mm^{-1}]	1.890
Scan method	*ω*
F (000)	728.0
Crystal size [mm^3]	0.15 × 0.12 × 0.1
Radiation type [mm]	Cu K*α* (λ = 1.54184)

2Θ range for data collection [°]	8.79 to 155.596
Index ranges (h, k, l)	$-22 \leq h \leq 22$, $-5 \leq k \leq 3$, $-22 \leq l \leq 26$
Reflections collected	14156
Independent reflections	3397 [R_{int} = 0.0399, R_{sigma} = 0.0347]
Data/restraints/parameters	3397/0/227
Goodness-of-fit on F^2	1.147
Final R indexes [I>=2σ (I)]	R_1 = 0.0440, wR_2 = 0.1169
Final R indexes [all data]	R_1 = 0.0486, wR_2 = 0.1230
Largest diff. peak/hole [e $Å^{-3}$]	0.29/-0.53

3. Experimental

3.1. ^{1}H NMR, ^{13}C NMR and HRMS Spectra of 2*H*-chromene-fused-thiazolo-triazole derivatives 4(a-r)

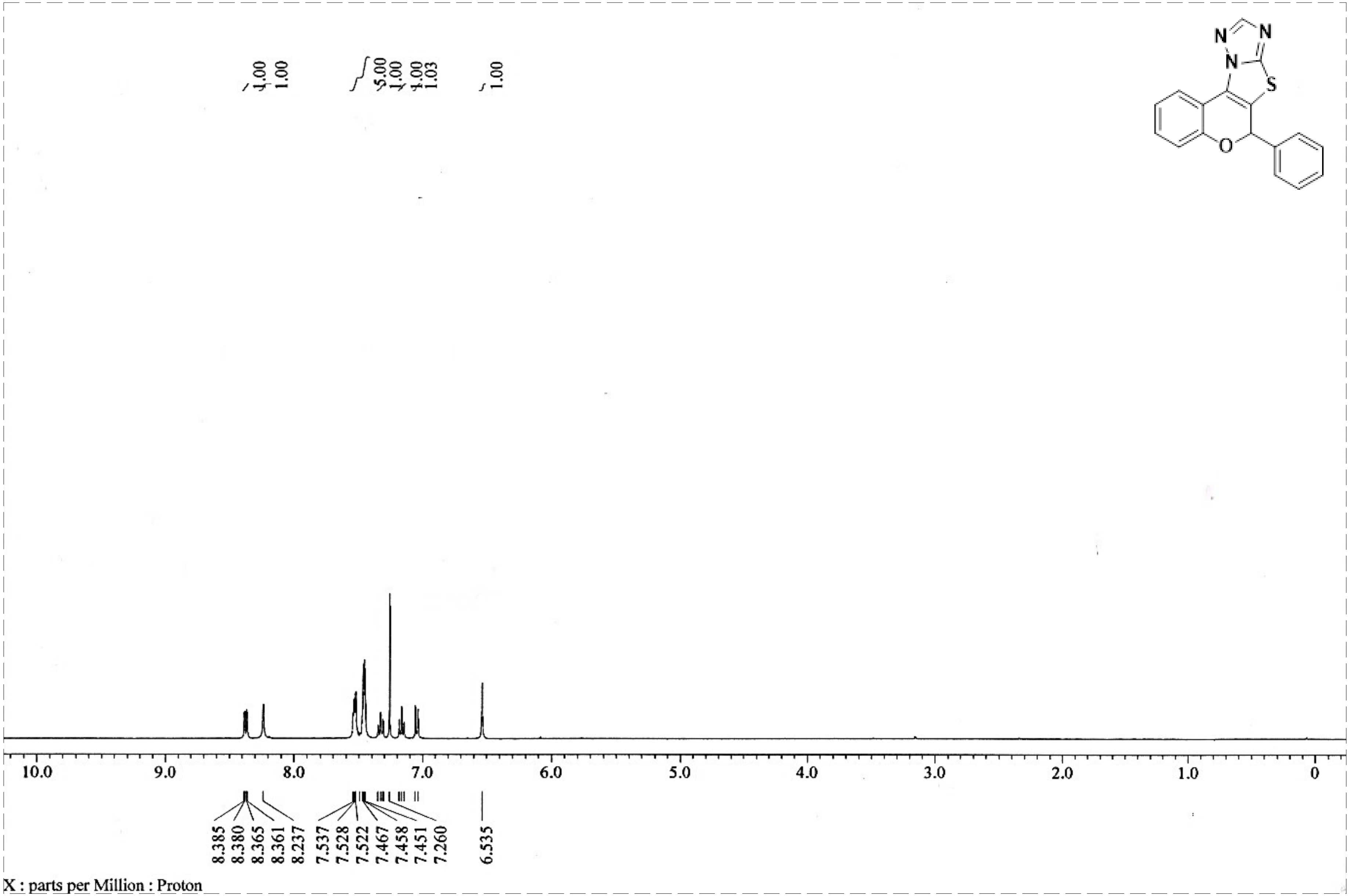
1.00
1.00
5.00
1.00
1.00
1.03
1.00
N
N
N
S
O
10.0
9.0
8.0
7.0
6.0
5.0
4.0
3.0
2.0
1.0
0
8.385
8.380
8.365
8.361
8.237
7.537
7.528
7.522
7.467
7.458
7.451
7.260
6.535
X : parts per Million : Proton

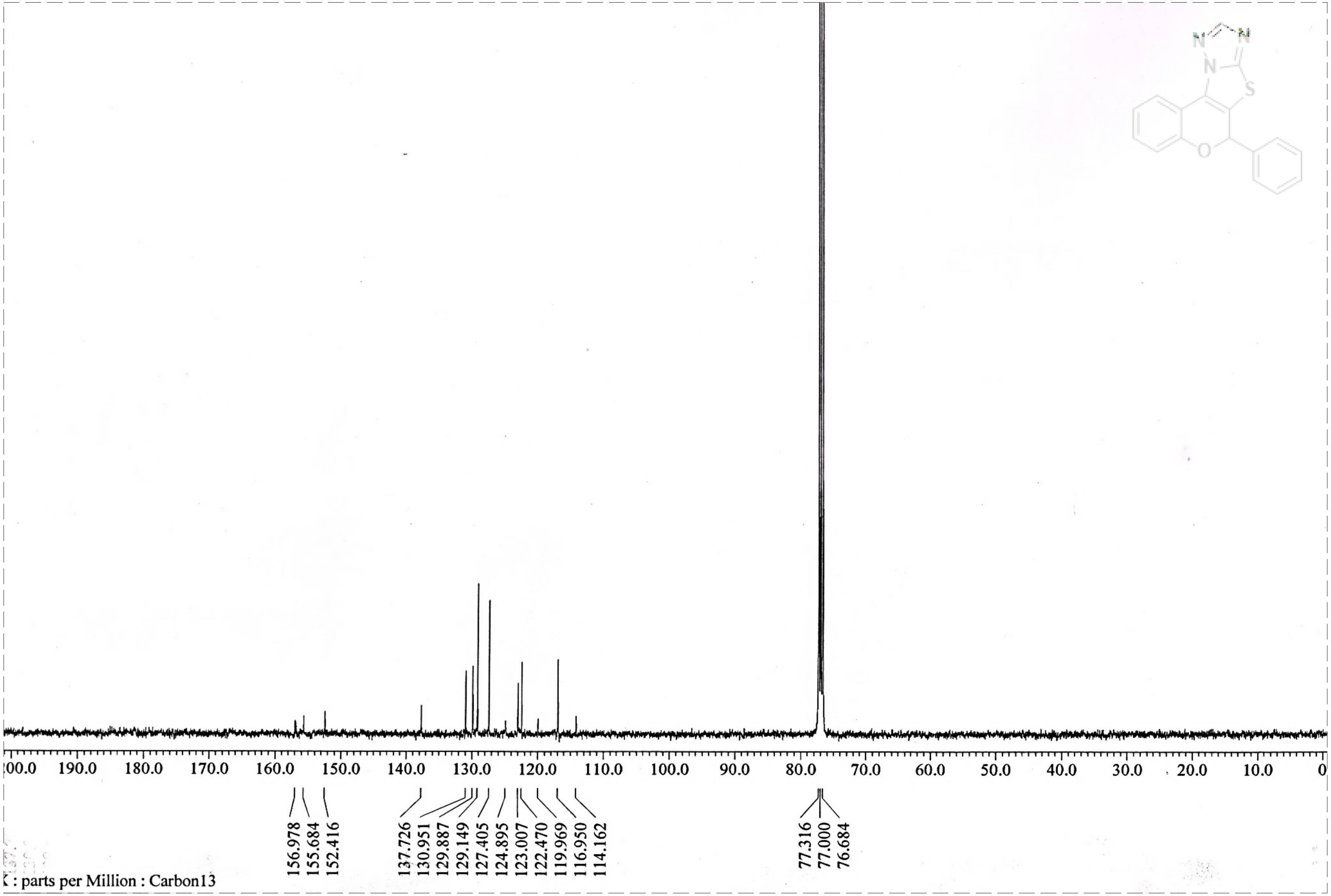

Fig S2. ^{13}C NMR Spectrum of compound **4a**

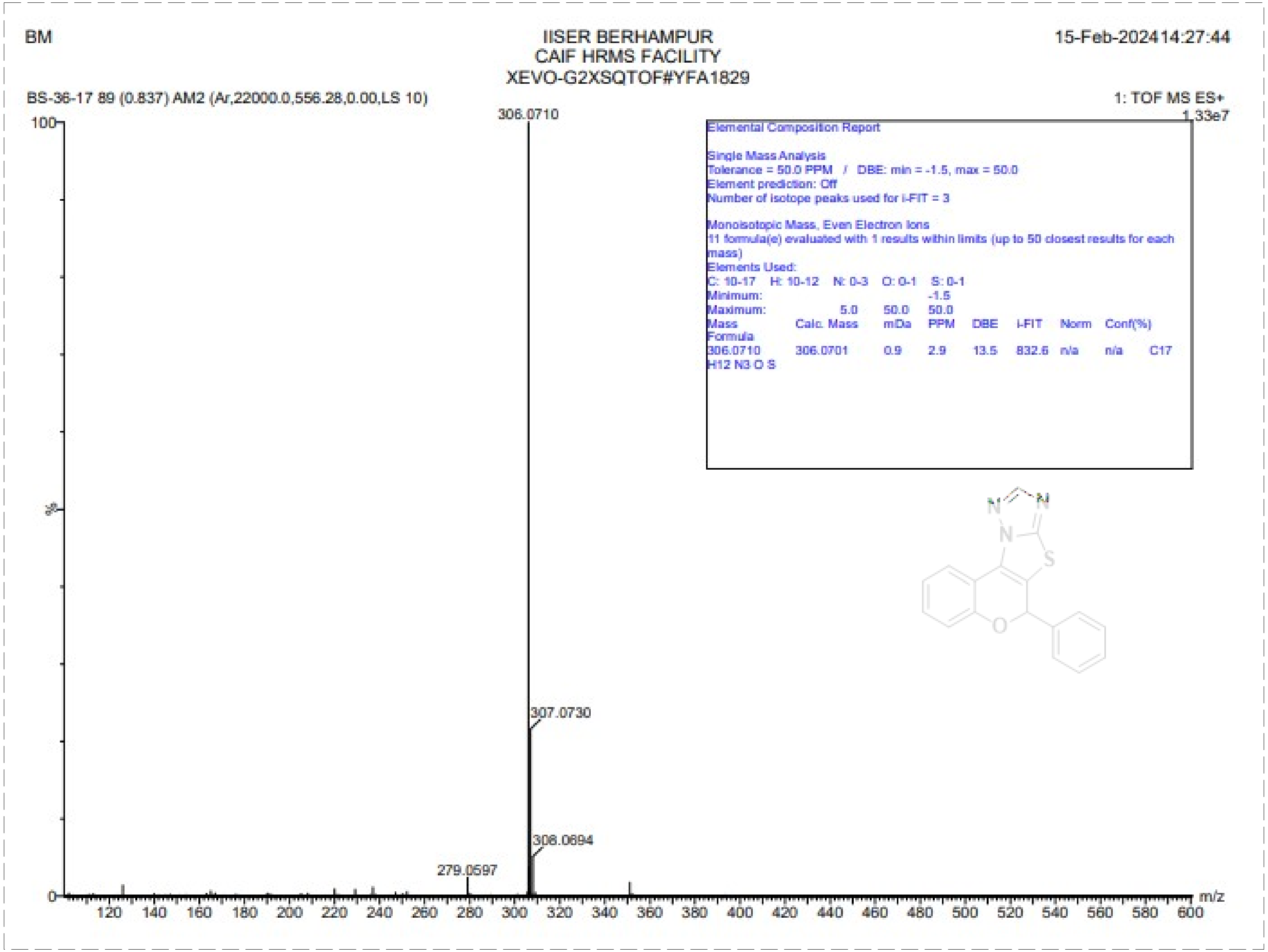

Fig S3. HRMS Spectrum of compound **4a**

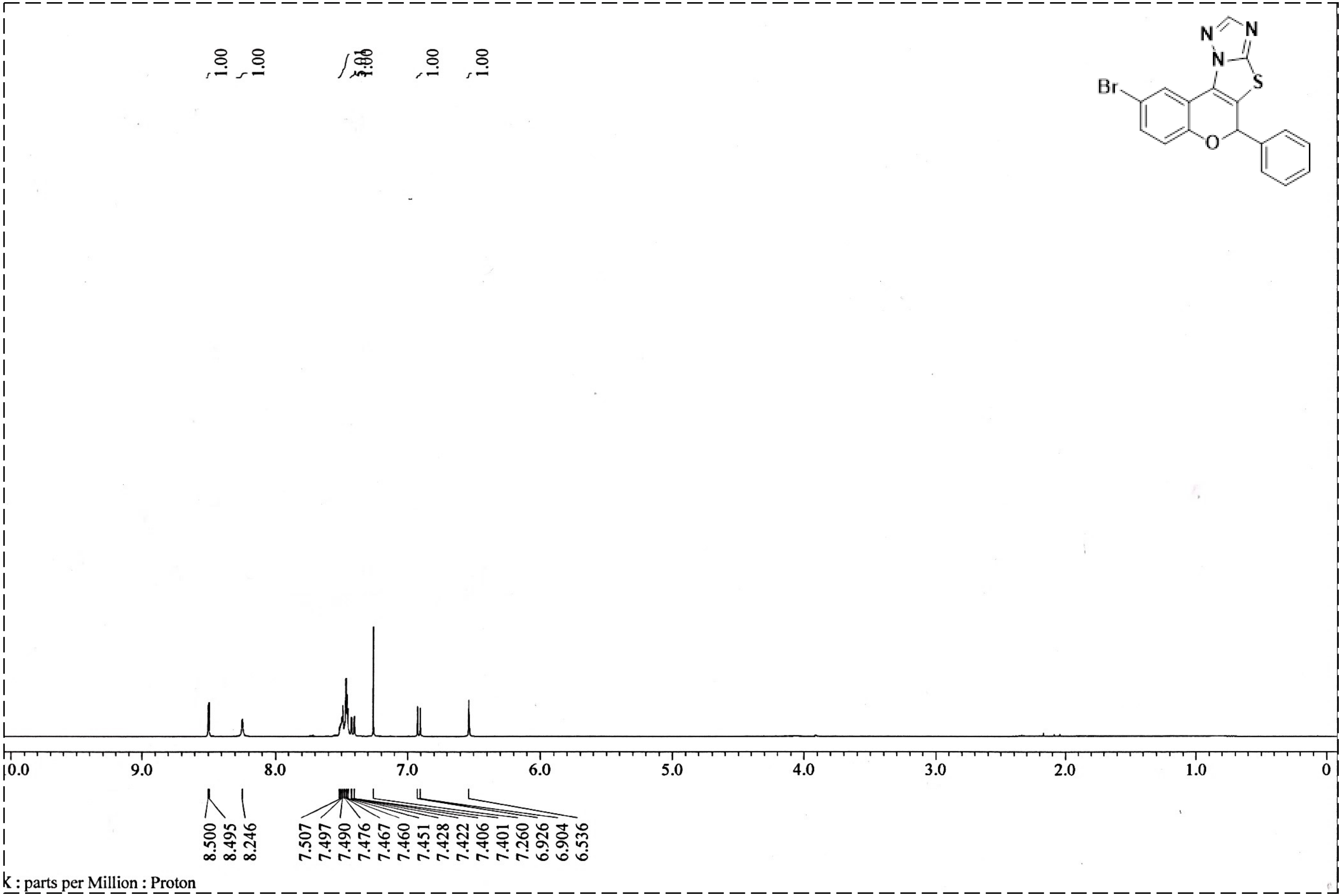

Fig S4. ^{1}H NMR Spectrum of compound **4b**

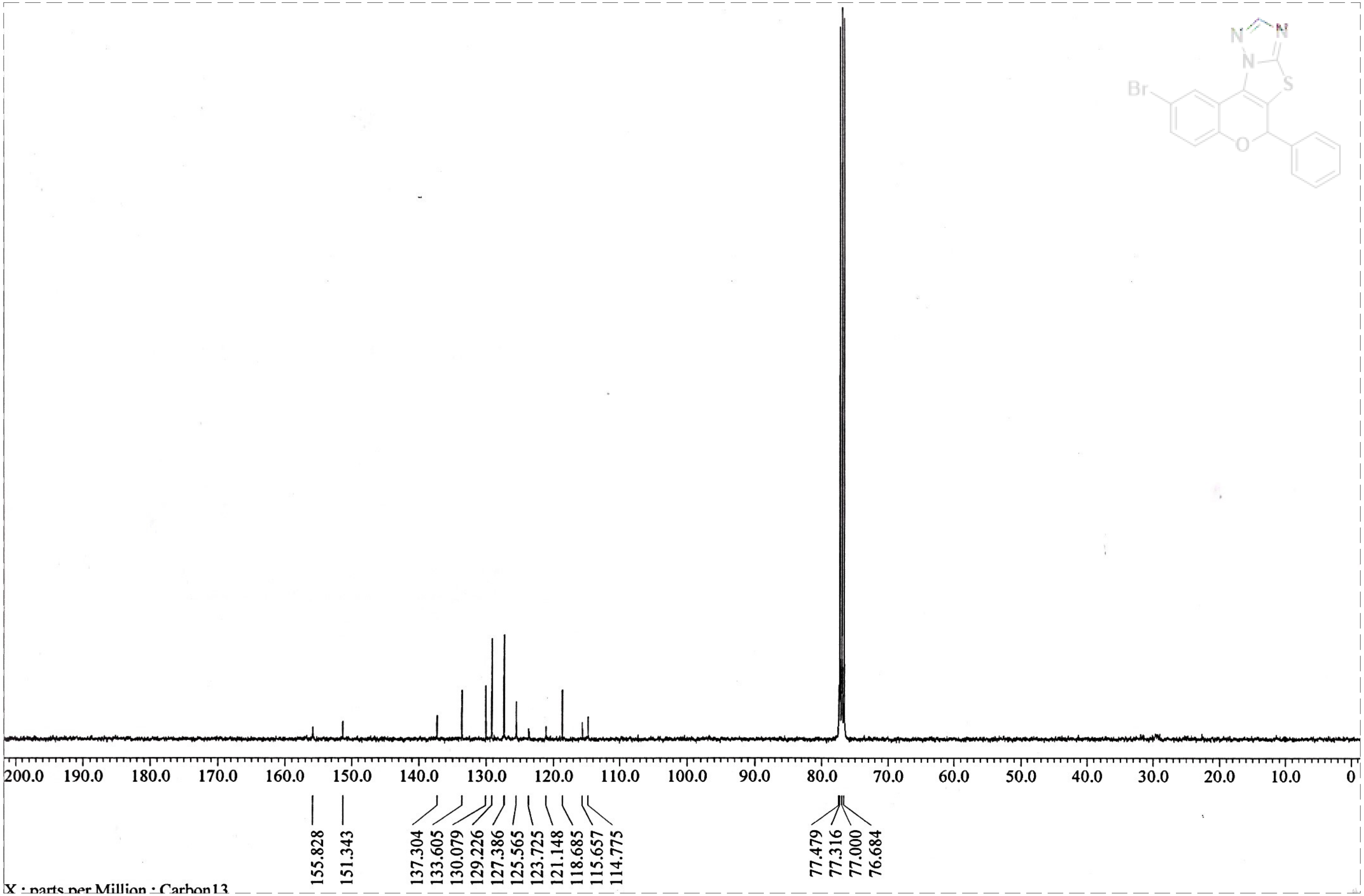

Fig S5. ^{13}C NMR Spectrum of compound **4b**

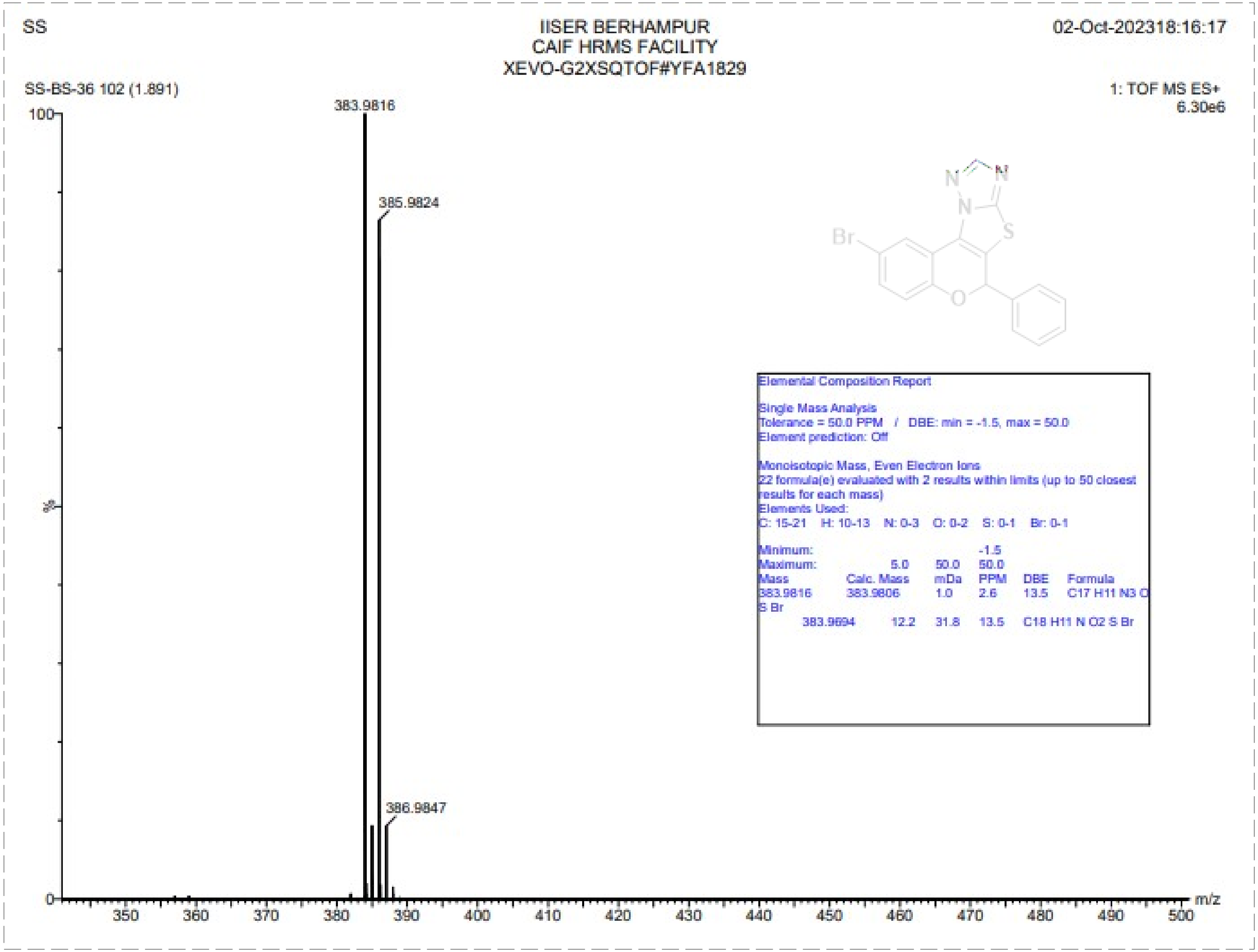

Fig S6. HRMS Spectrum of compound **4b**

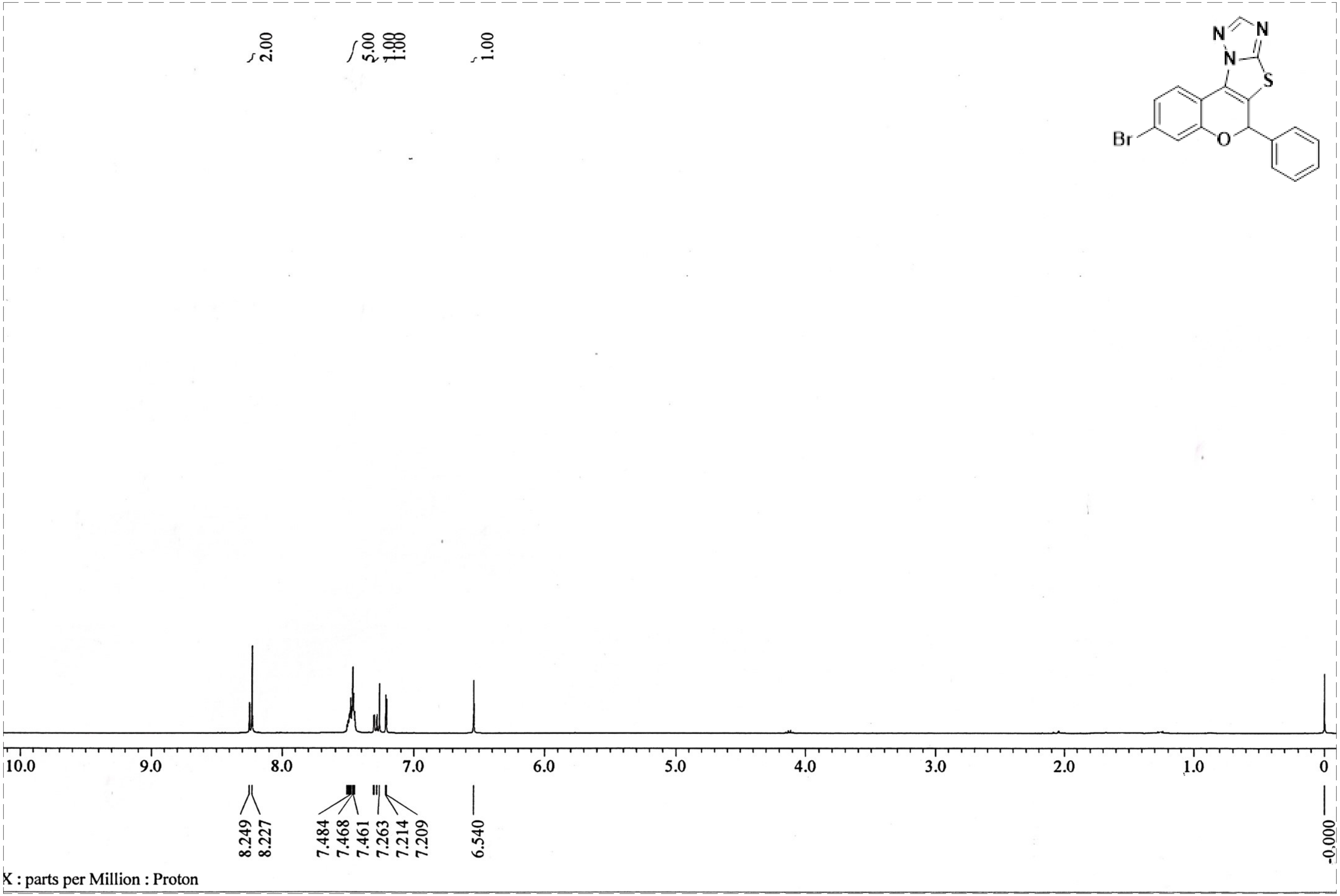

Fig S7. ^{1}H NMR Spectrum of compound **4c**

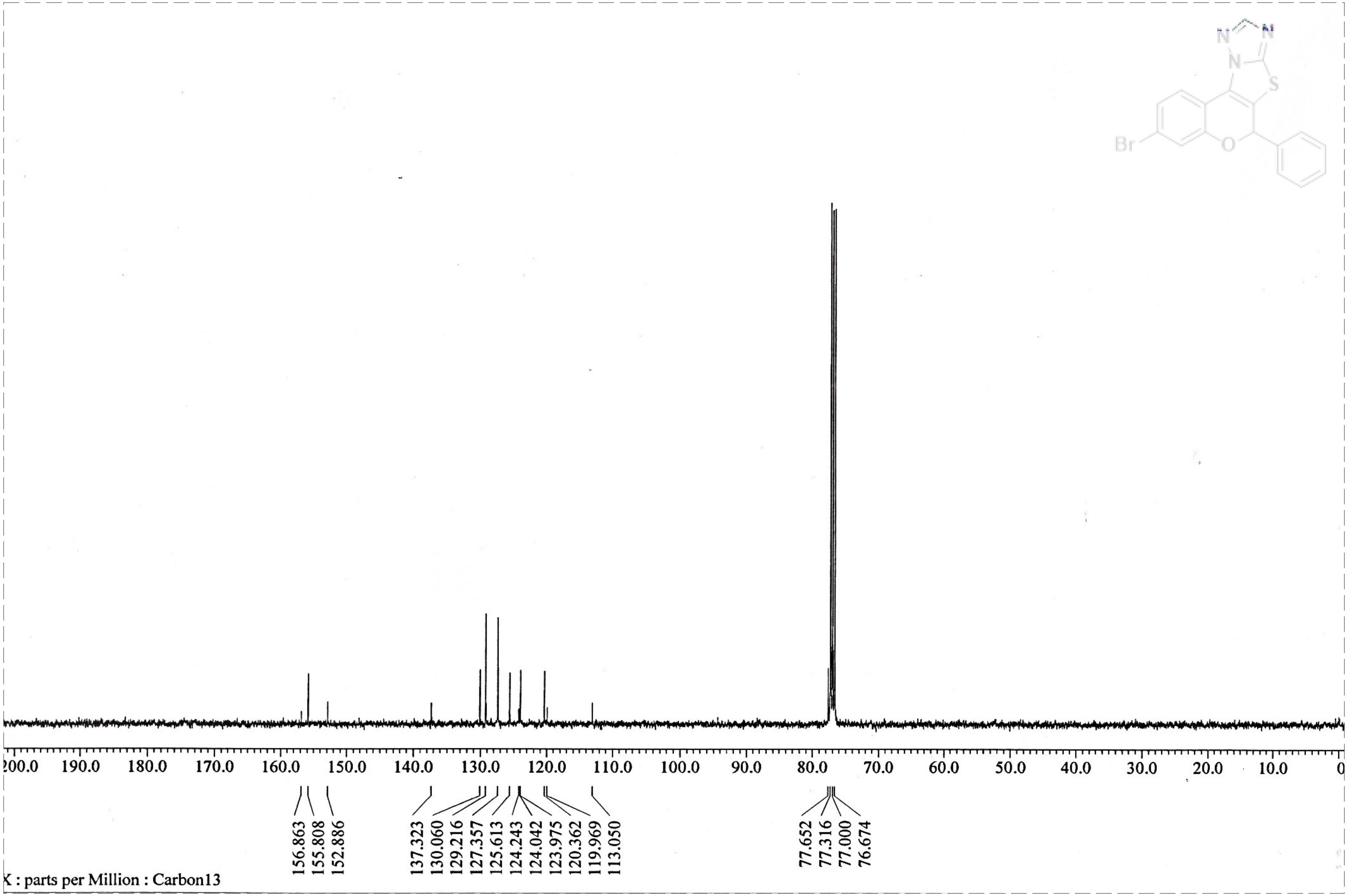

Fig S8. ^{13}C NMR Spectrum of compound **4c**

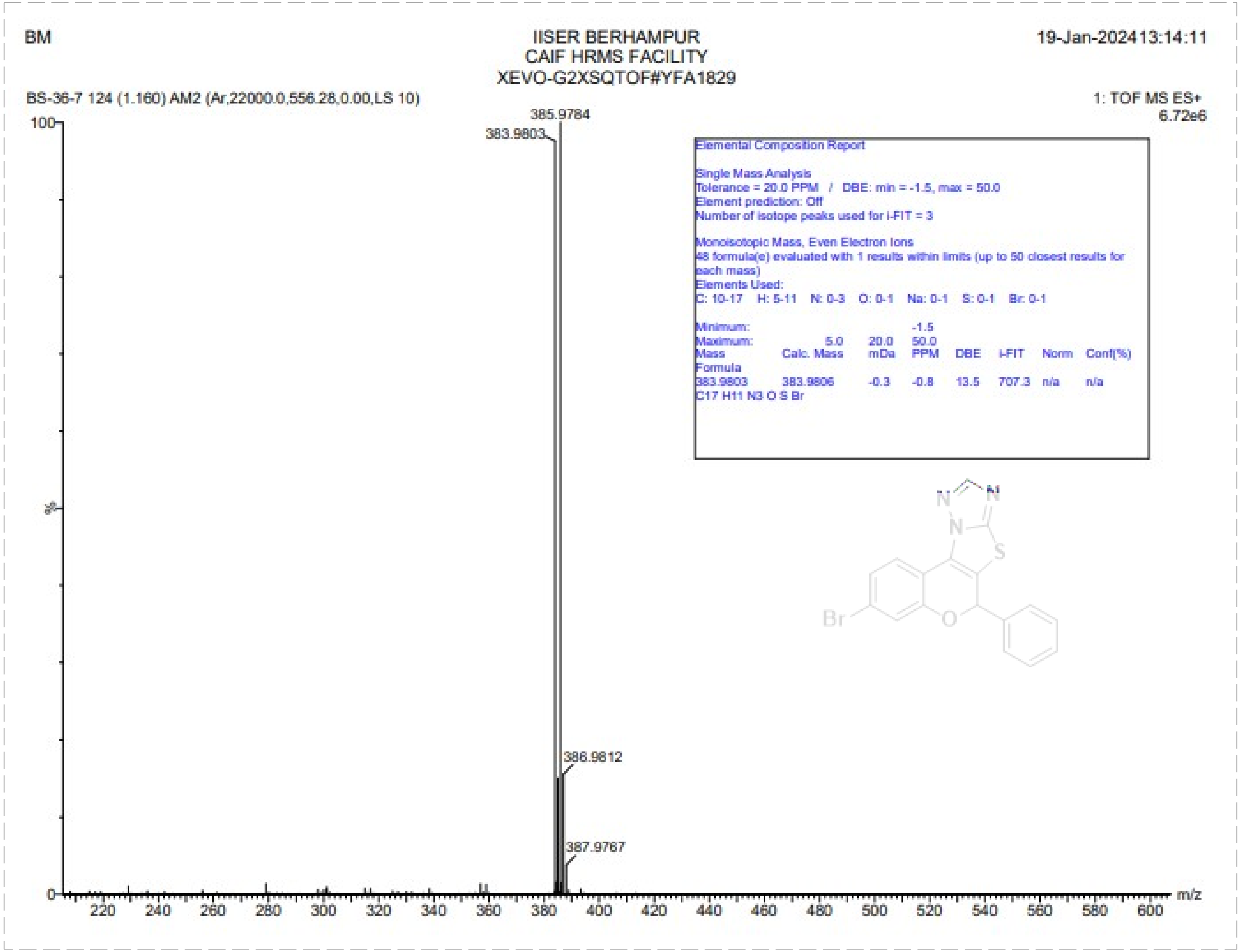

Fig S9. HRMS Spectrum of compound **4c**

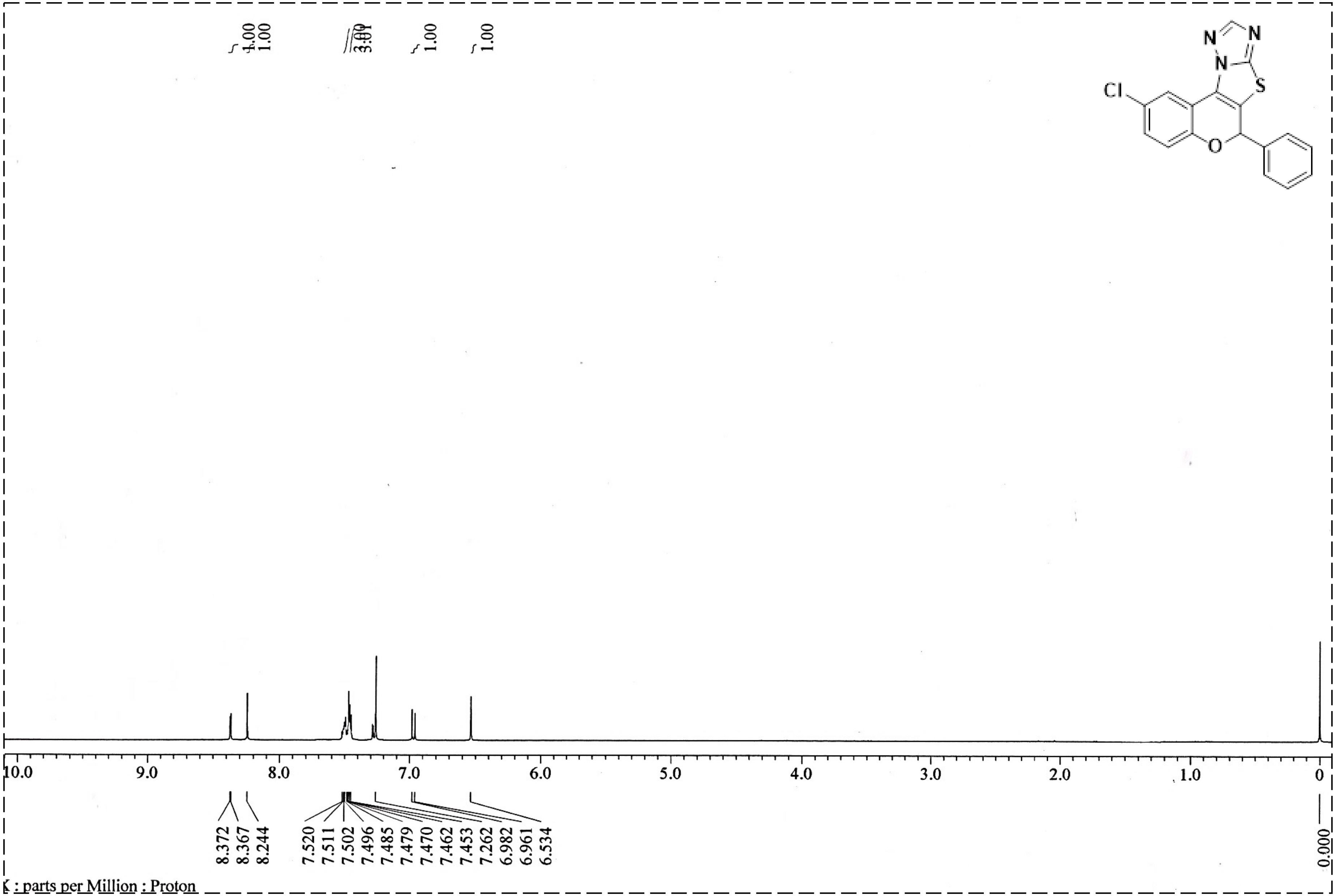

Fig S10. ¹H NMR Spectrum of compound **4d**

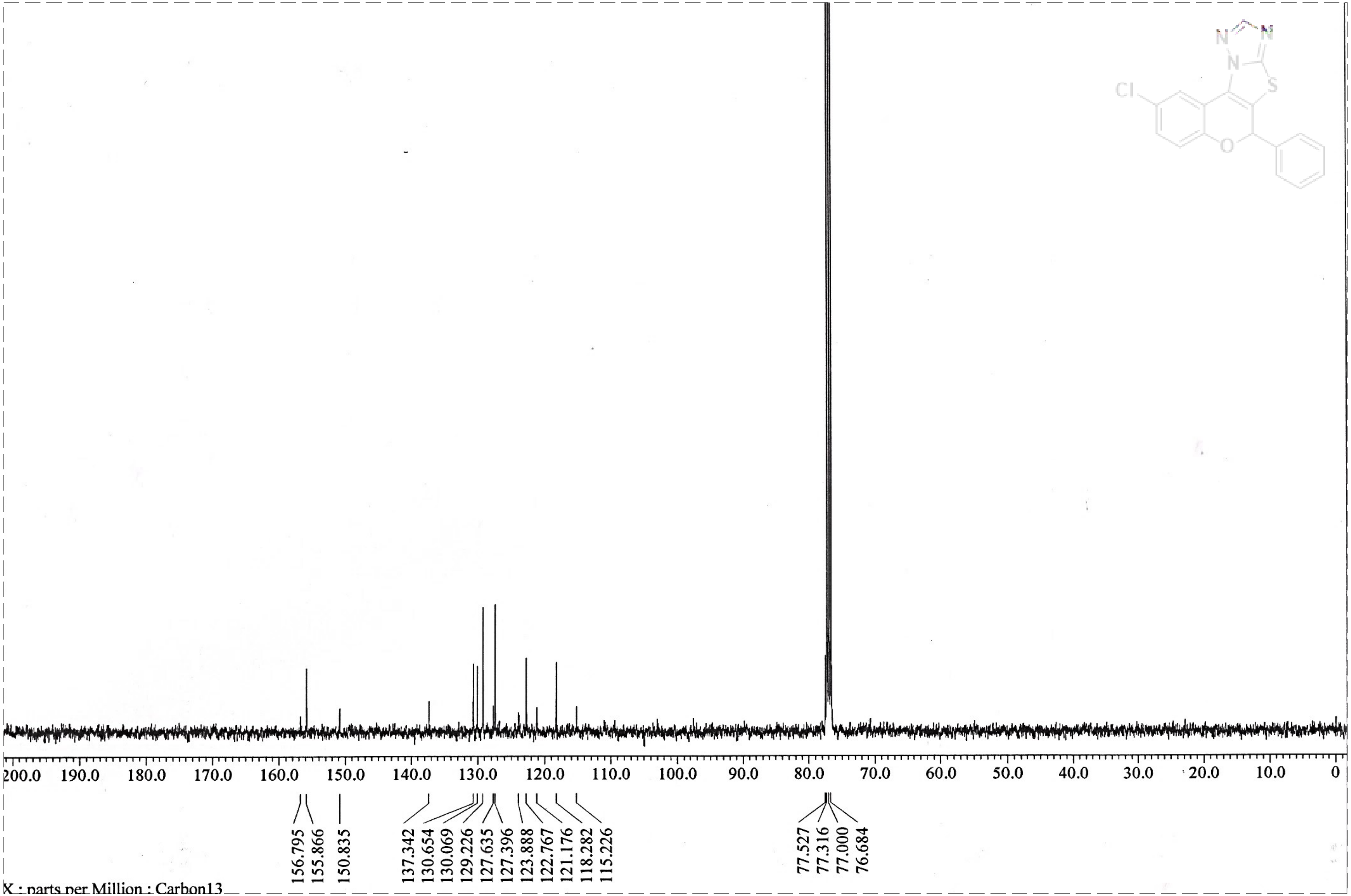

Fig S11. ^{13}C NMR Spectrum of compound **4d**

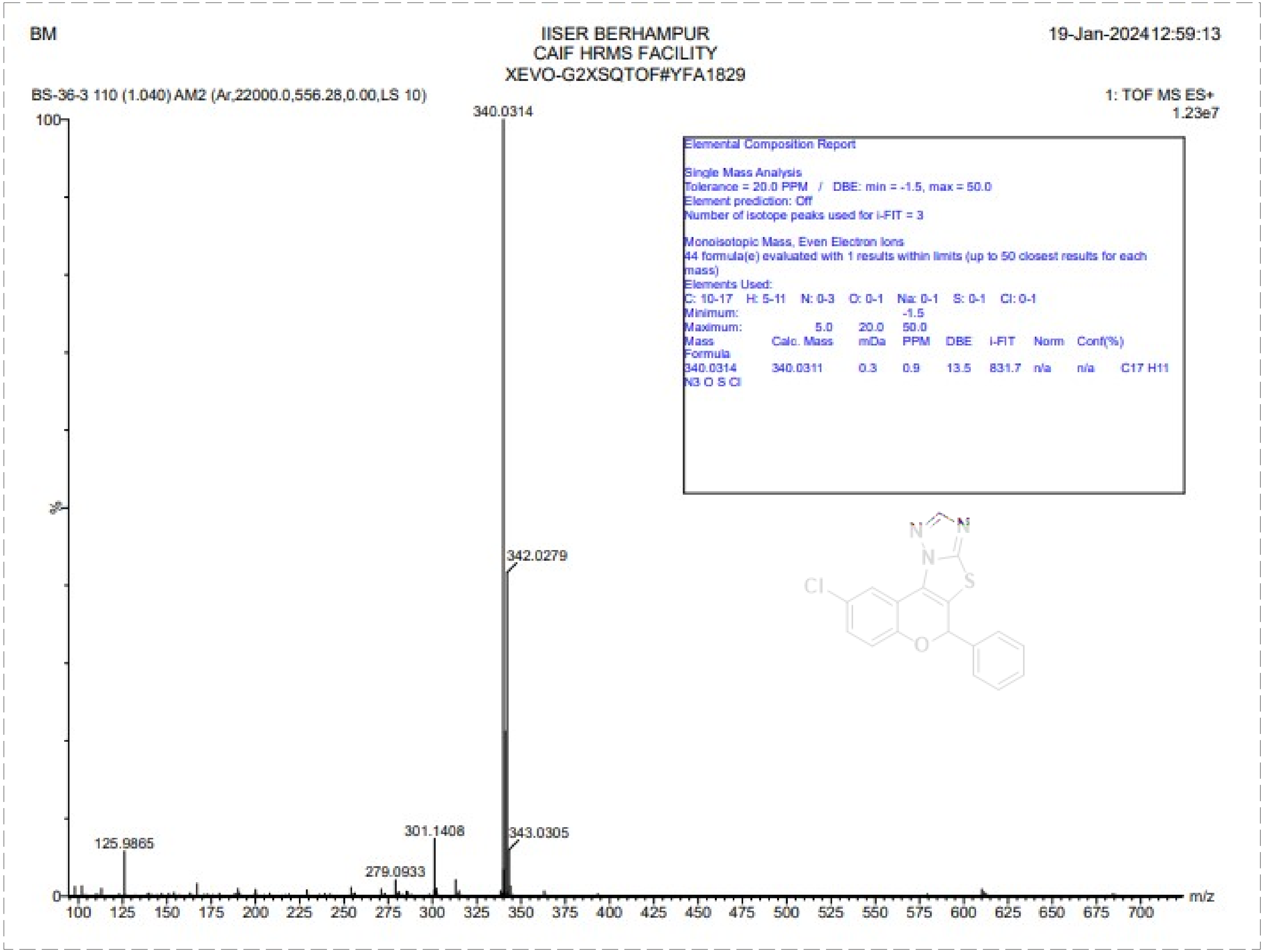

Fig S12. HRMS Spectrum of compound **4d**

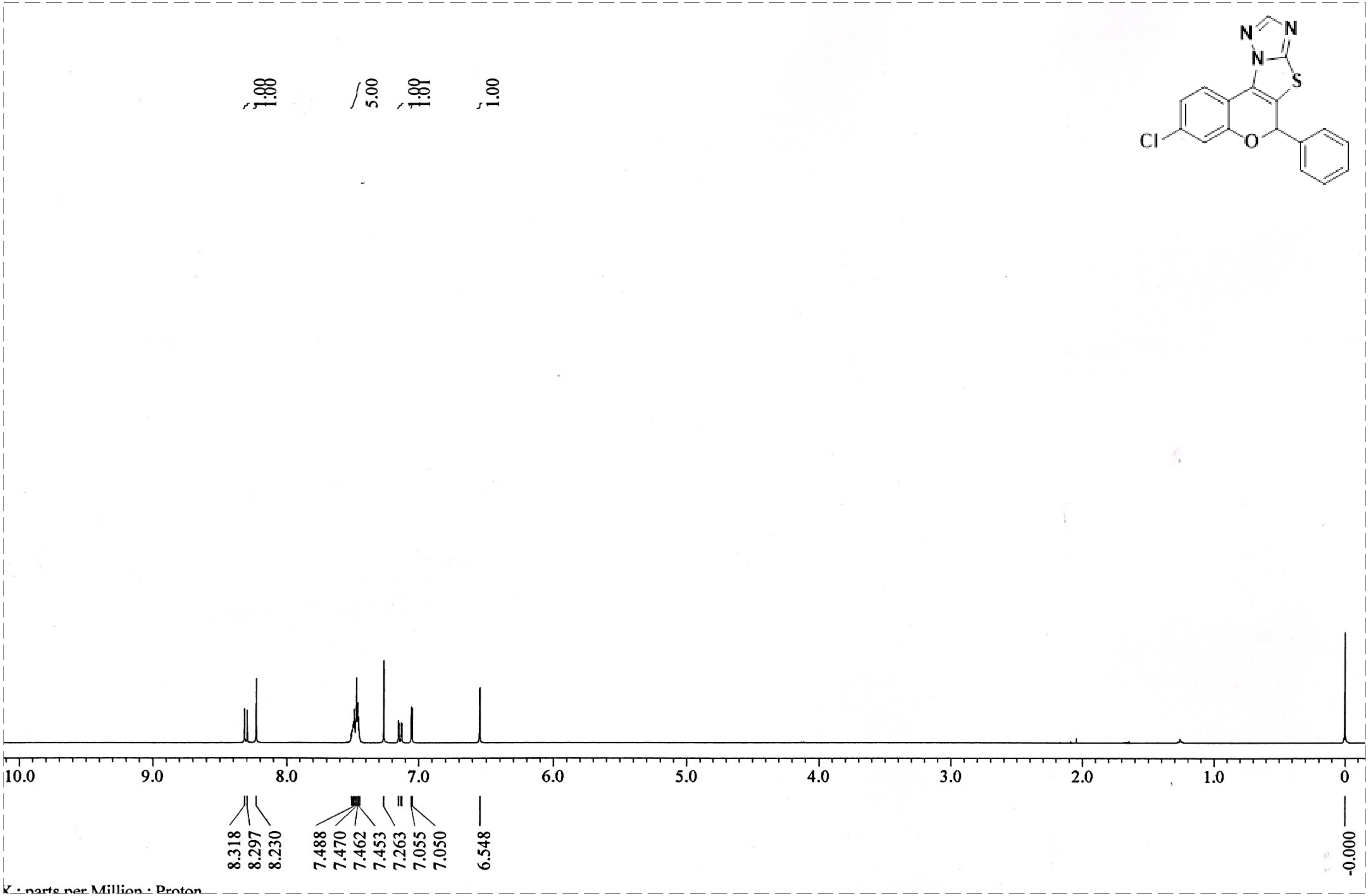

Fig S13. ^{1}H NMR Spectrum of compound **4e**

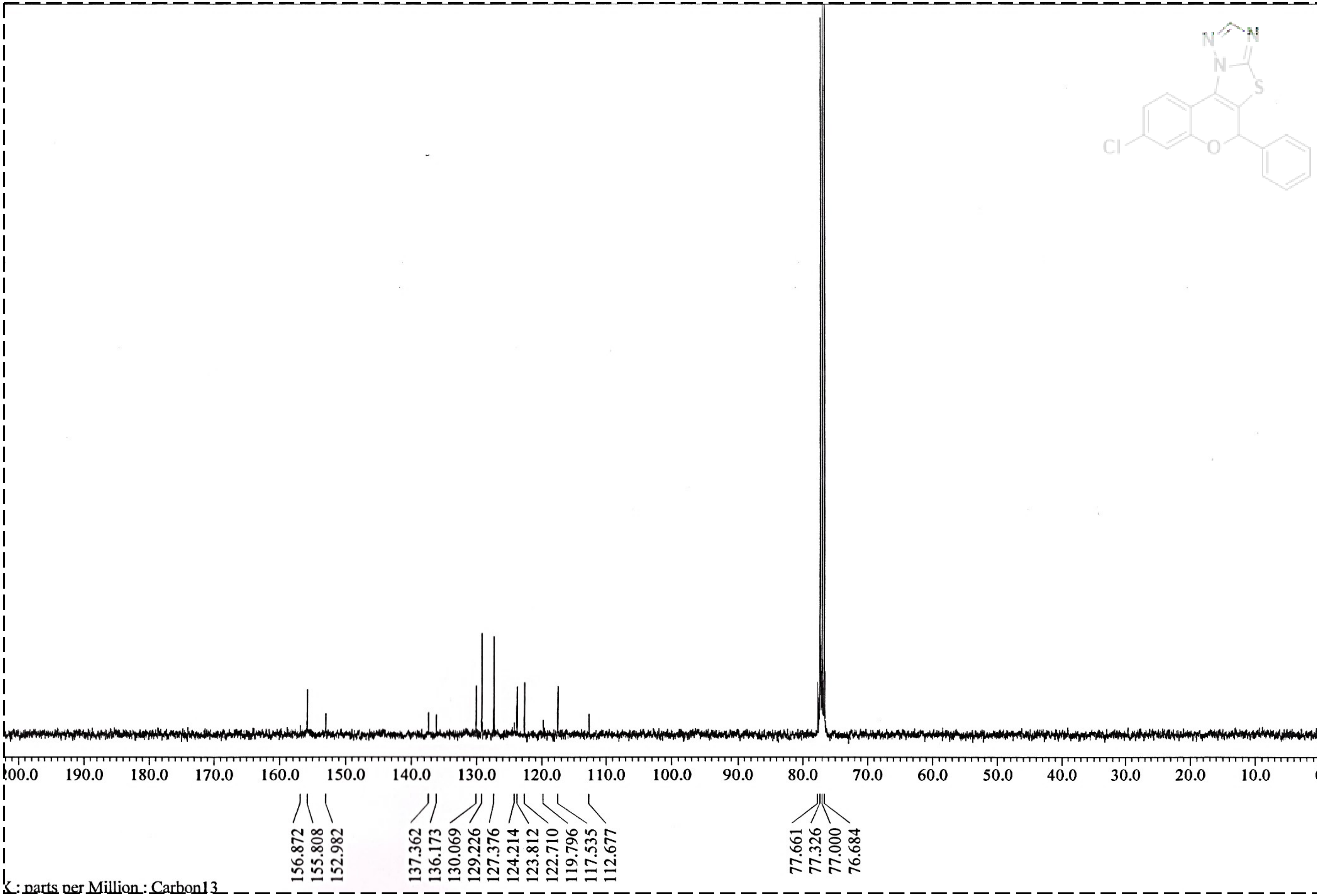

Fig S14. ^{13}C NMR Spectrum of compound **4e**

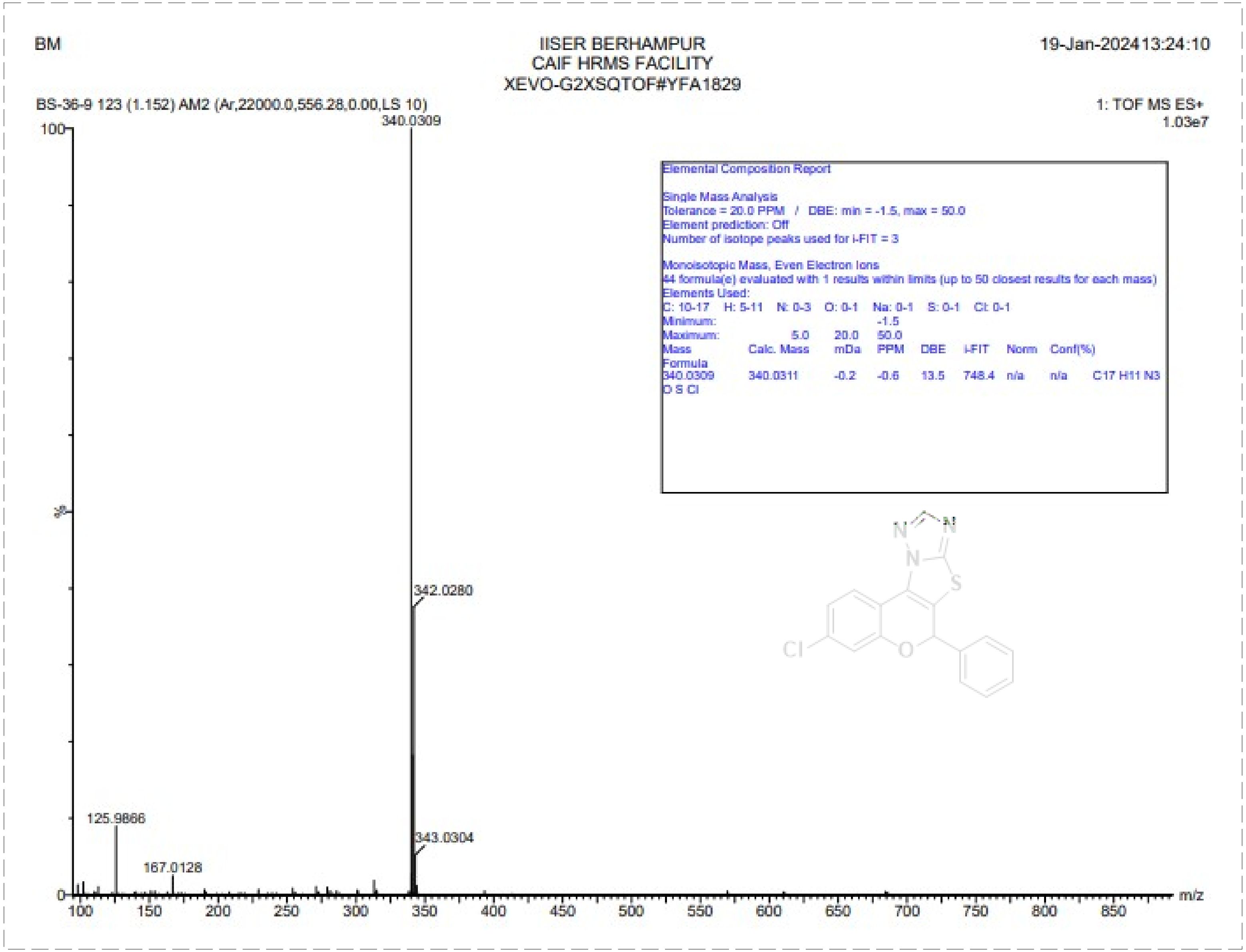

Fig S15. HRMS Spectrum of compound **4e**

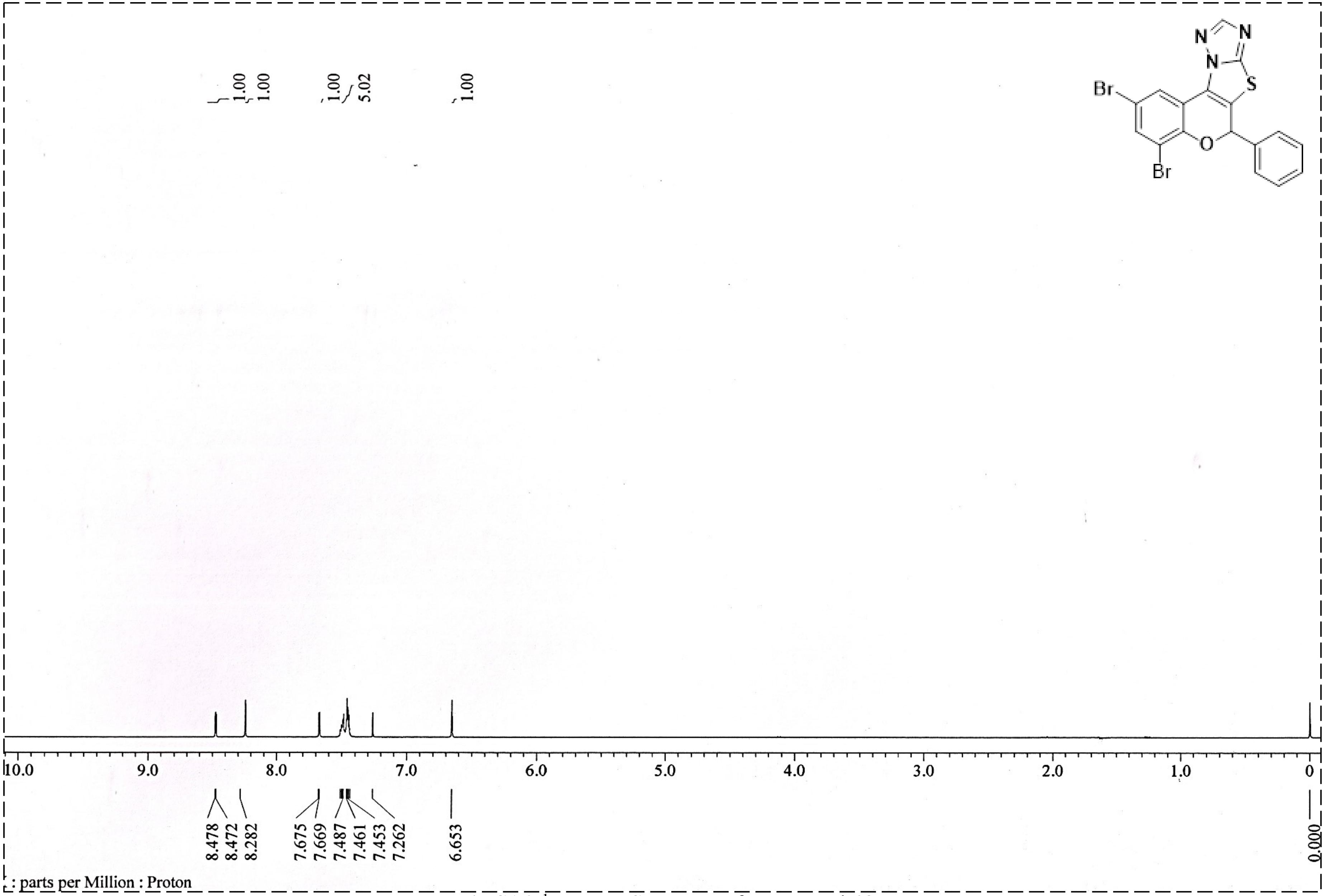

Fig S16. ^{1}H NMR Spectrum of compound **4f**

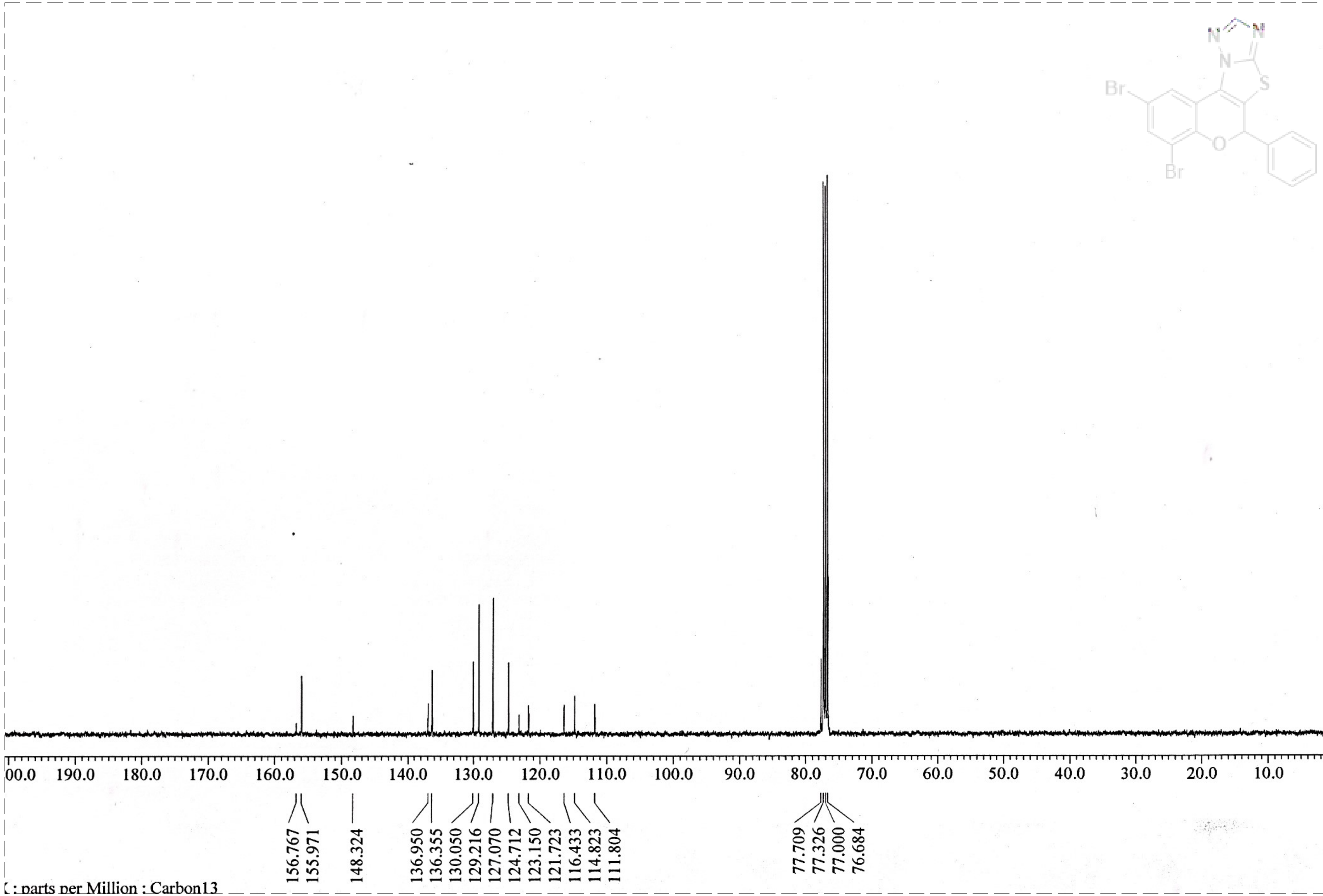

Fig S17. ^{13}C NMR Spectrum of compound **4f**

Compound Details

Cpd. 1: C17 H9 Br2 N3 O S

Formula	m/z	Observed M/Z	Difference_Da	Difference_PPM	Score
C17 H9 Br2 N3 O S	463.8889	463.888851871904	0.370472451379555	0.803831348581856	99.37

Compound Spectra (Zoomed)

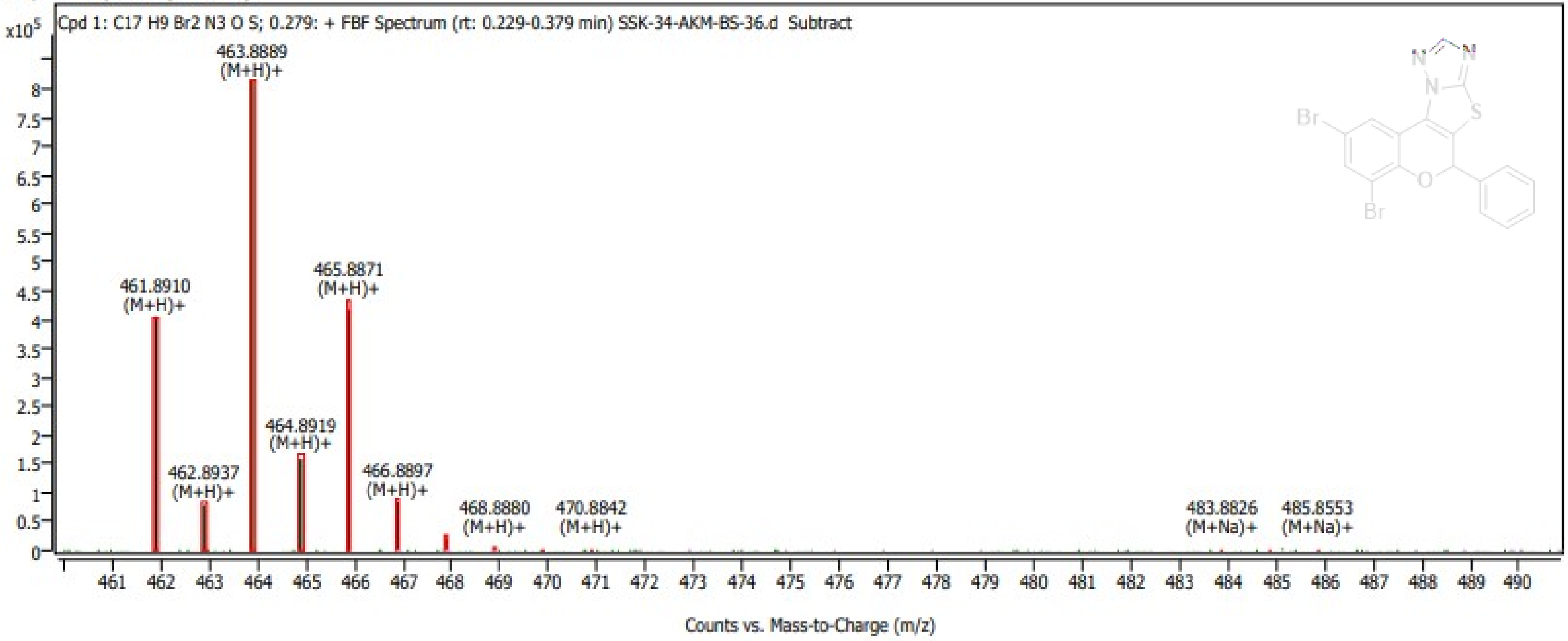

Fig S18. HRMS Spectrum of compound **4f**

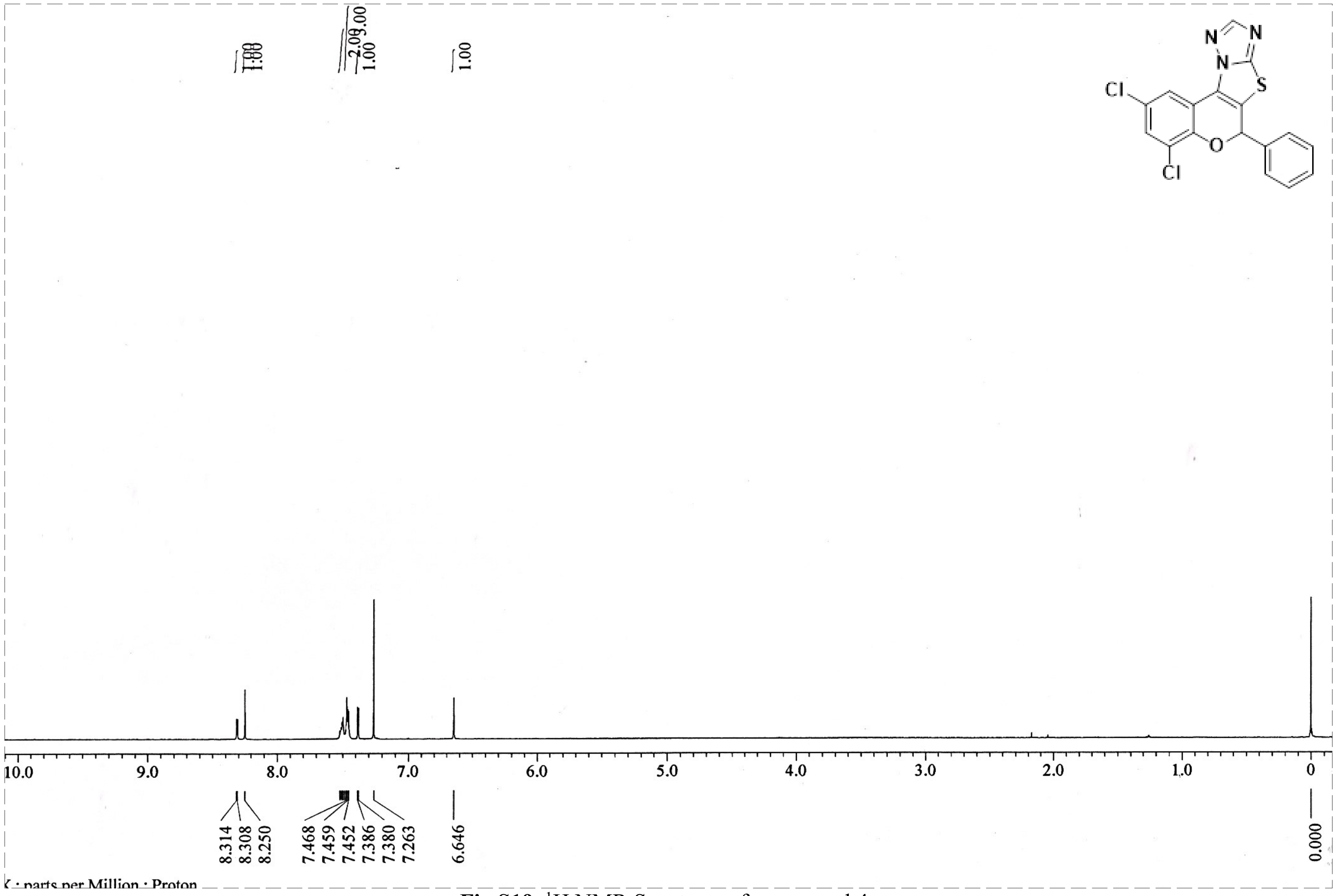

Fig S19. ^{1}H NMR Spectrum of compound **4g**

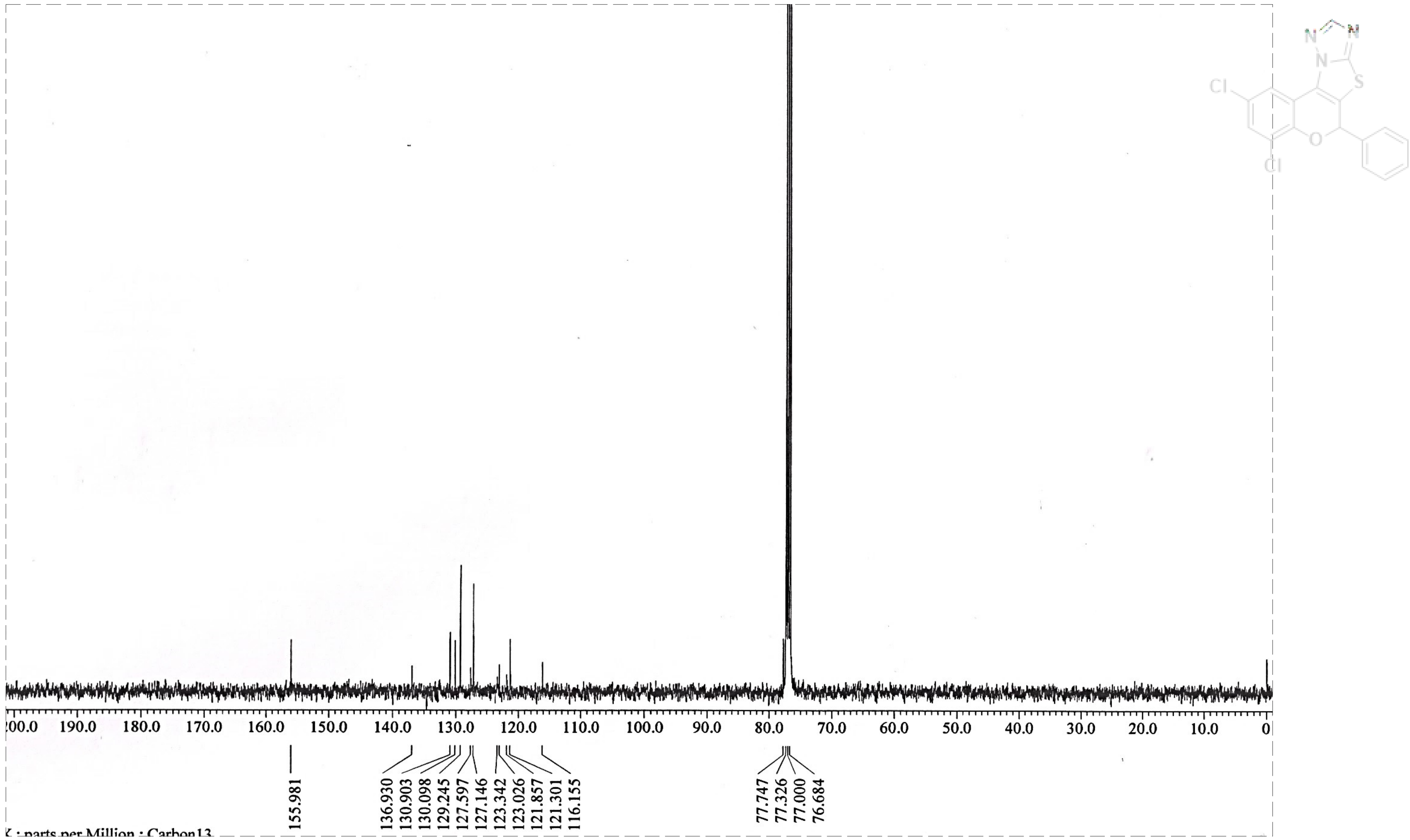

Fig S20. ^{13}C NMR Spectrum of compound **4g**

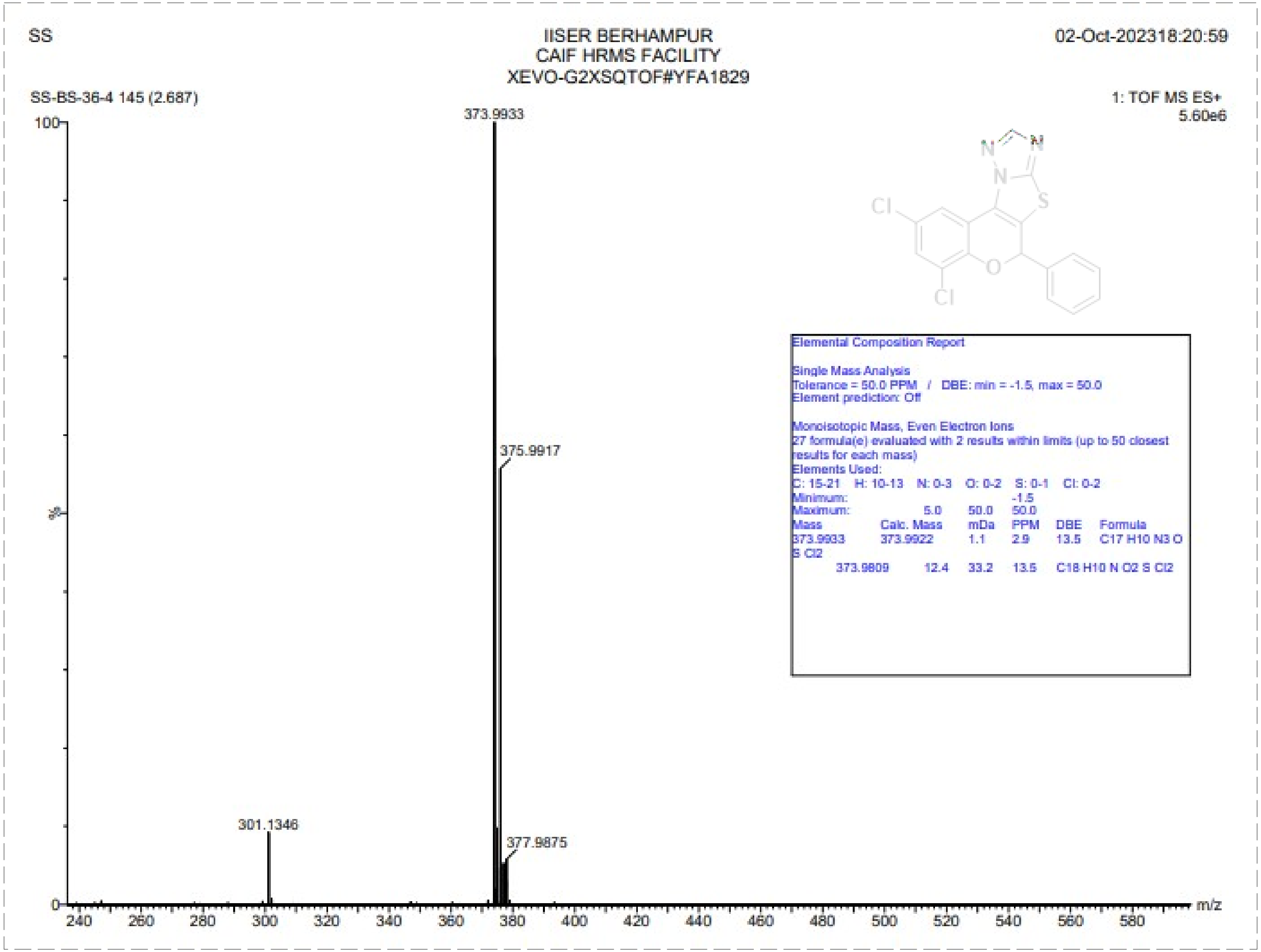

Fig S21. HRMS Spectrum of compound **4g**

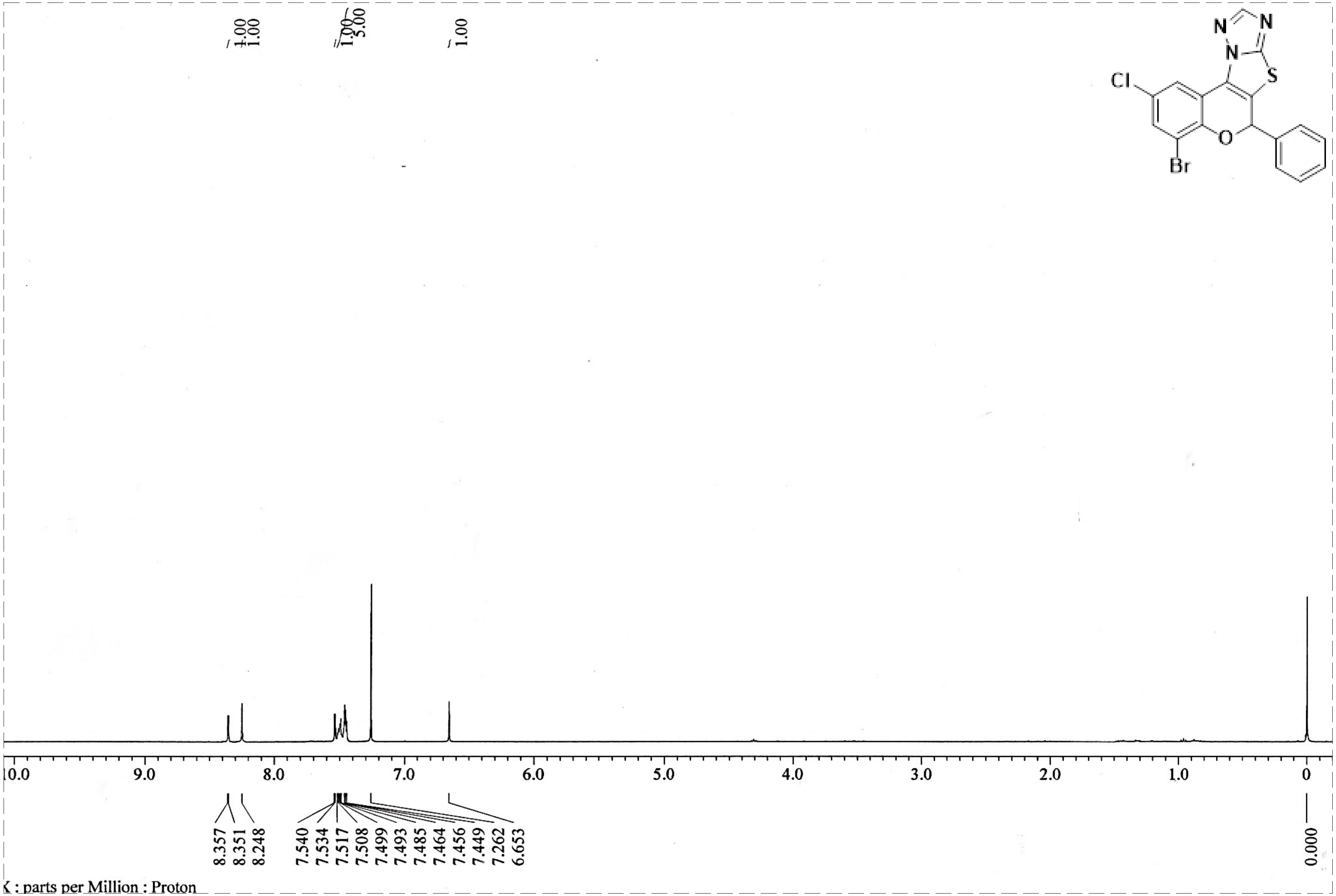

Fig S22. ^{1}H NMR Spectrum of compound **4h**

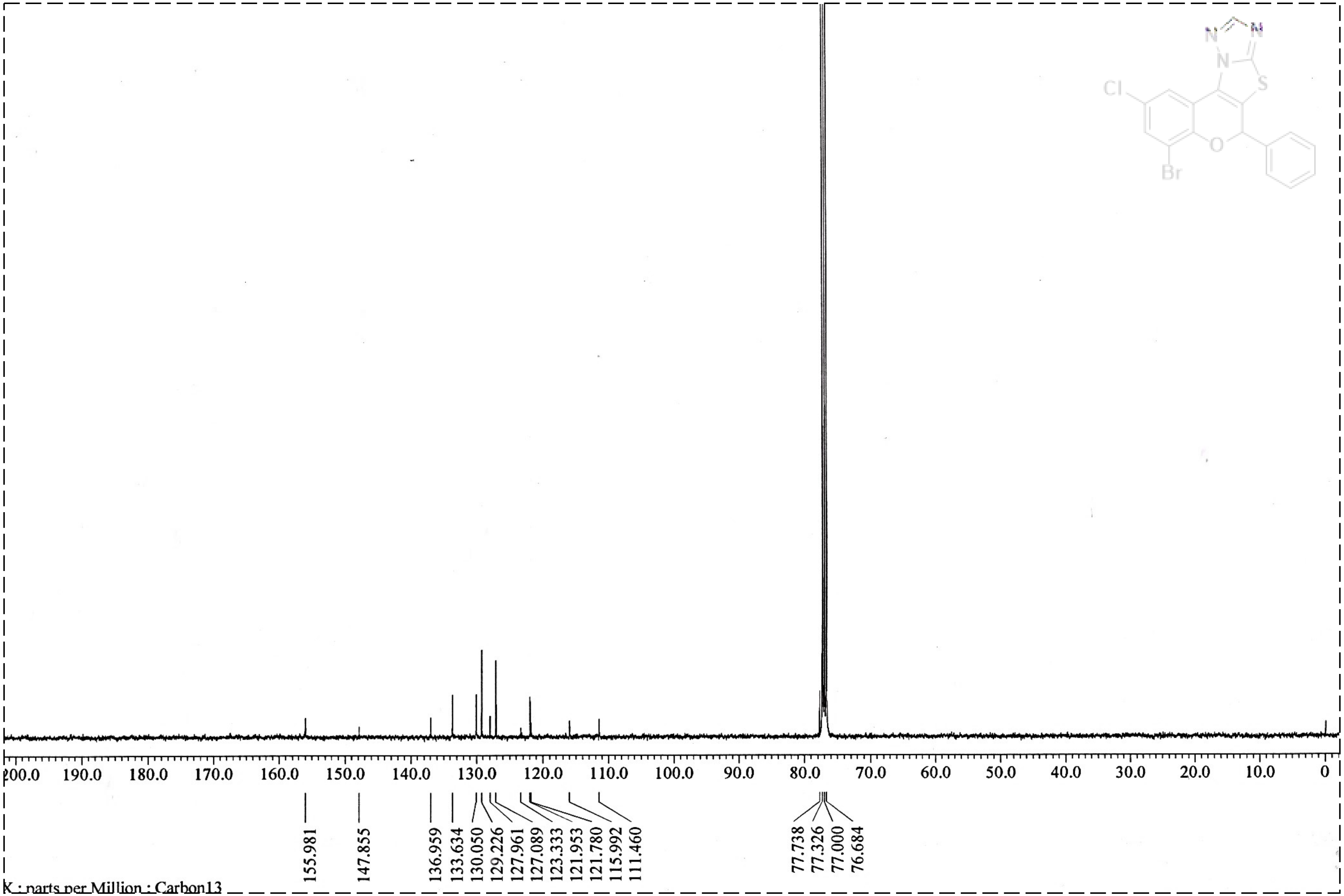

Fig S23. ^{13}C NMR Spectrum of compound **4h**

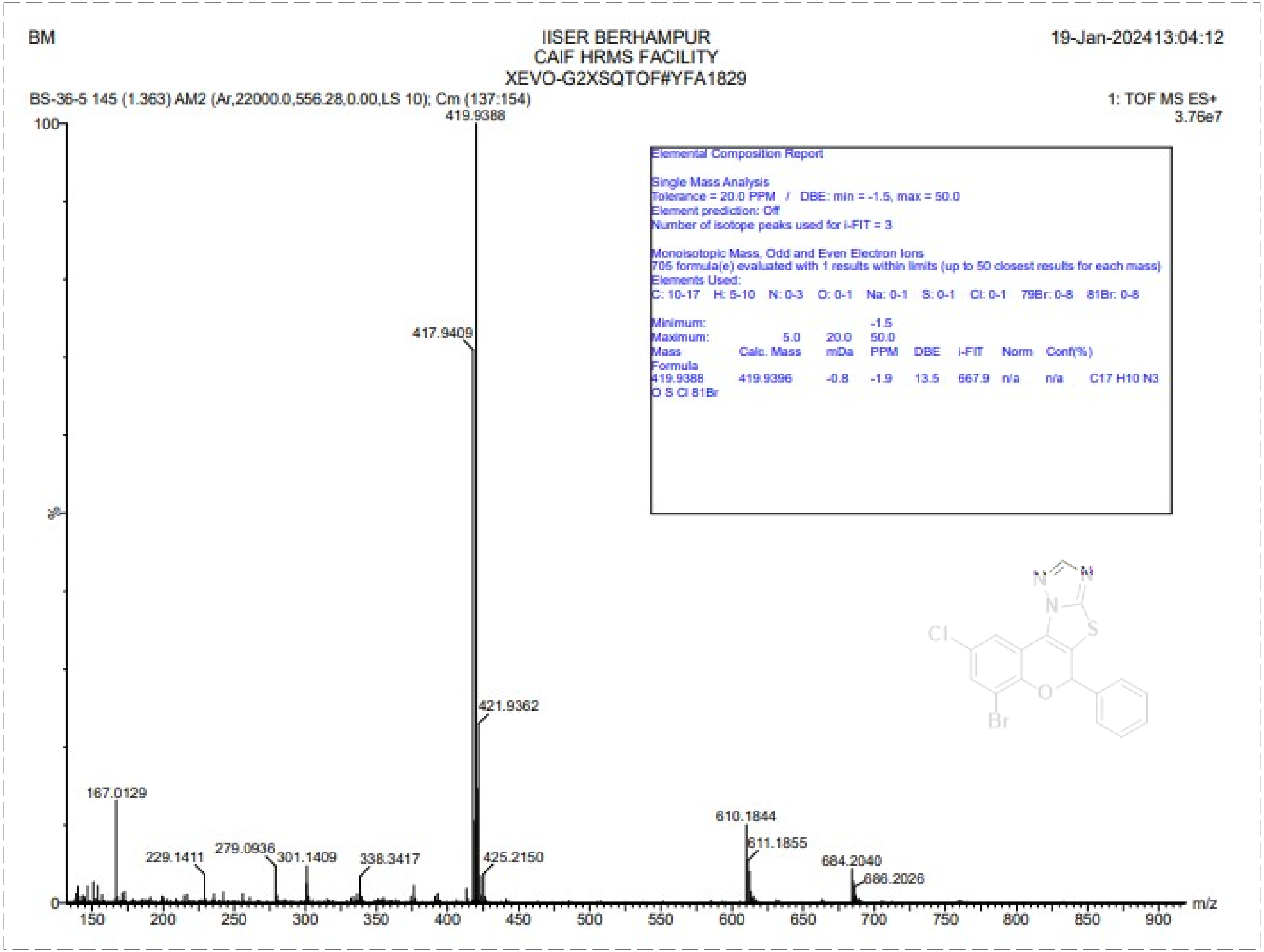

Fig S24. HRMS Spectrum of compound **4h**

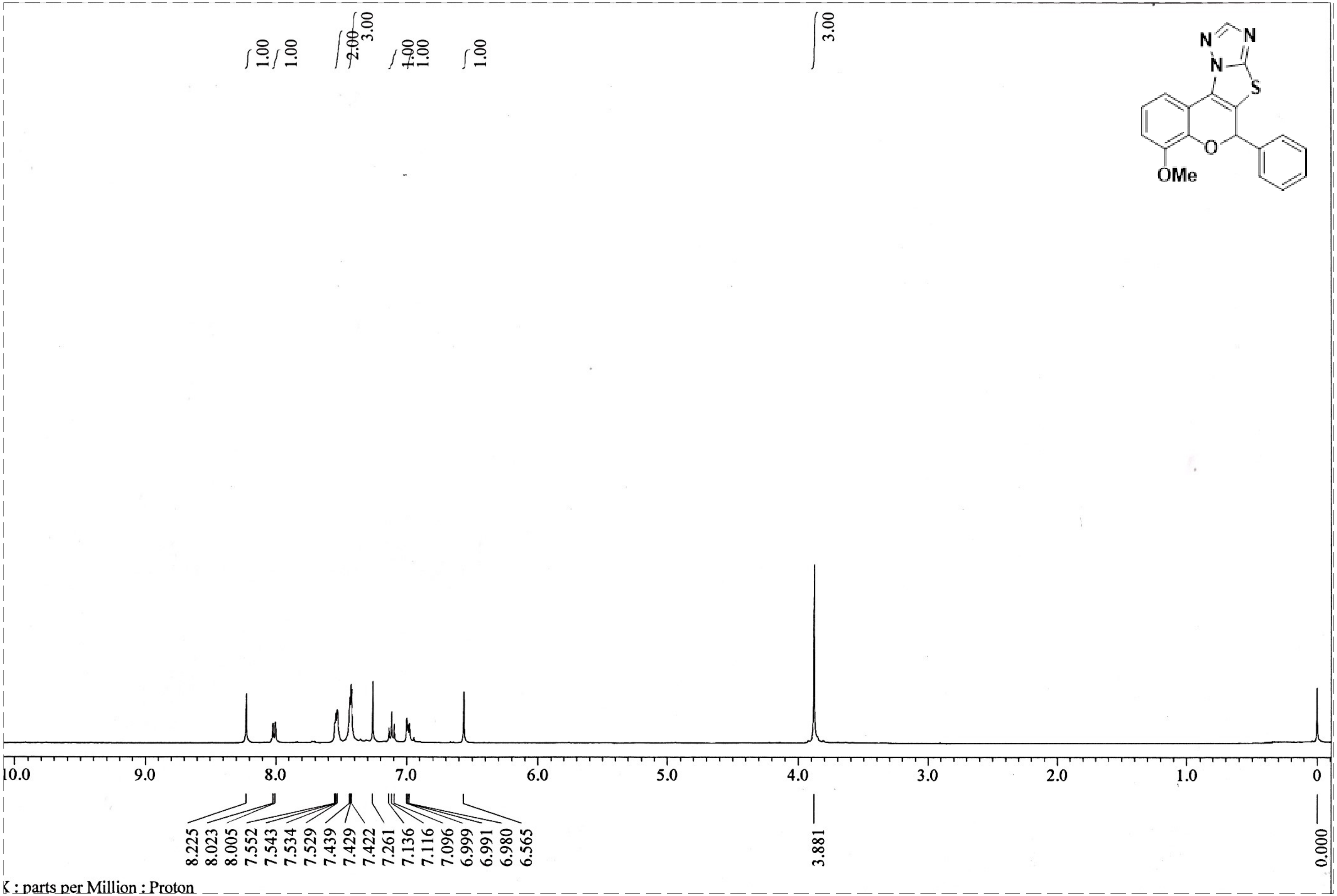

Fig S25. ^{1}H NMR Spectrum of compound **4i**

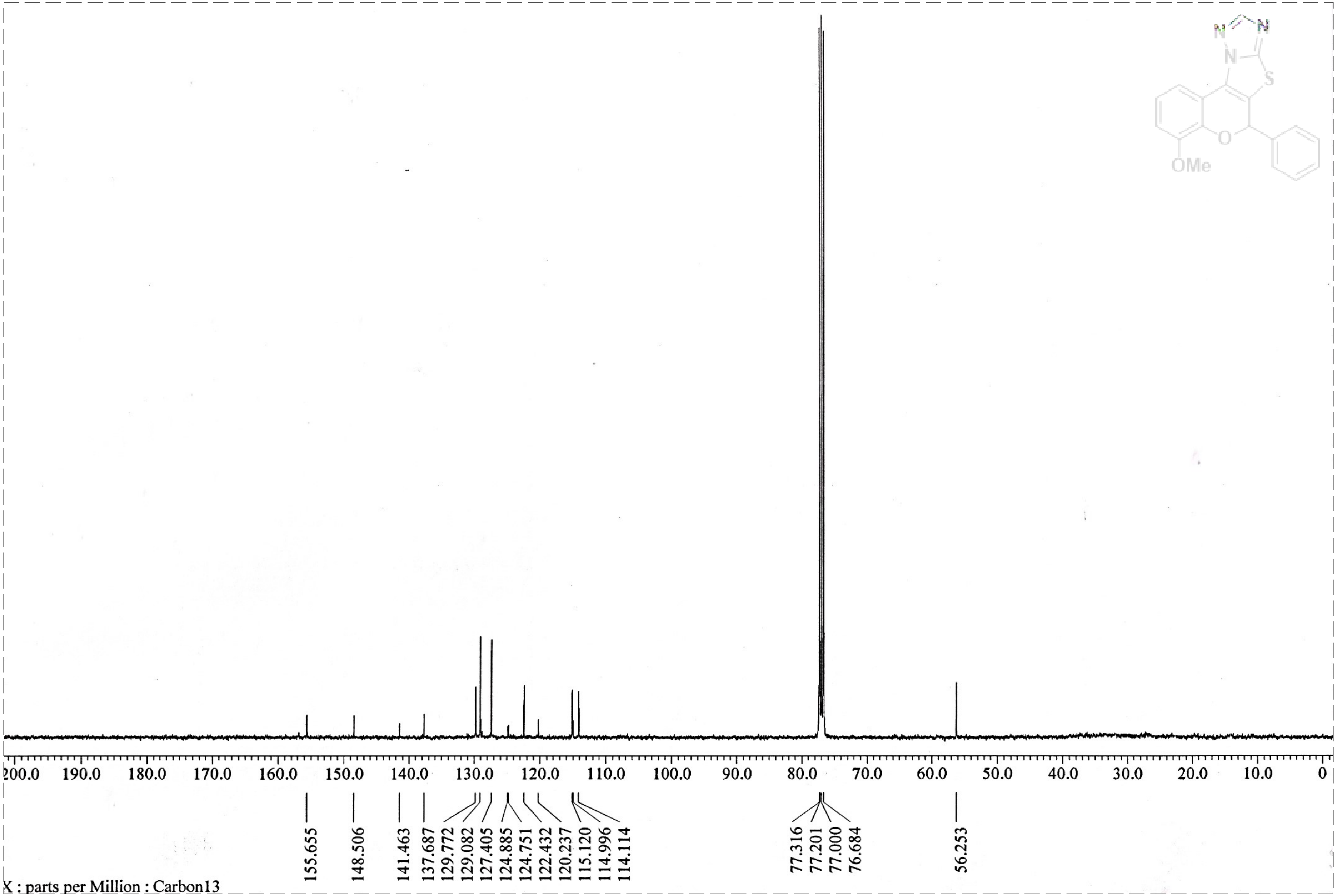

Fig S26. ^{13}C NMR Spectrum of compound **4i**

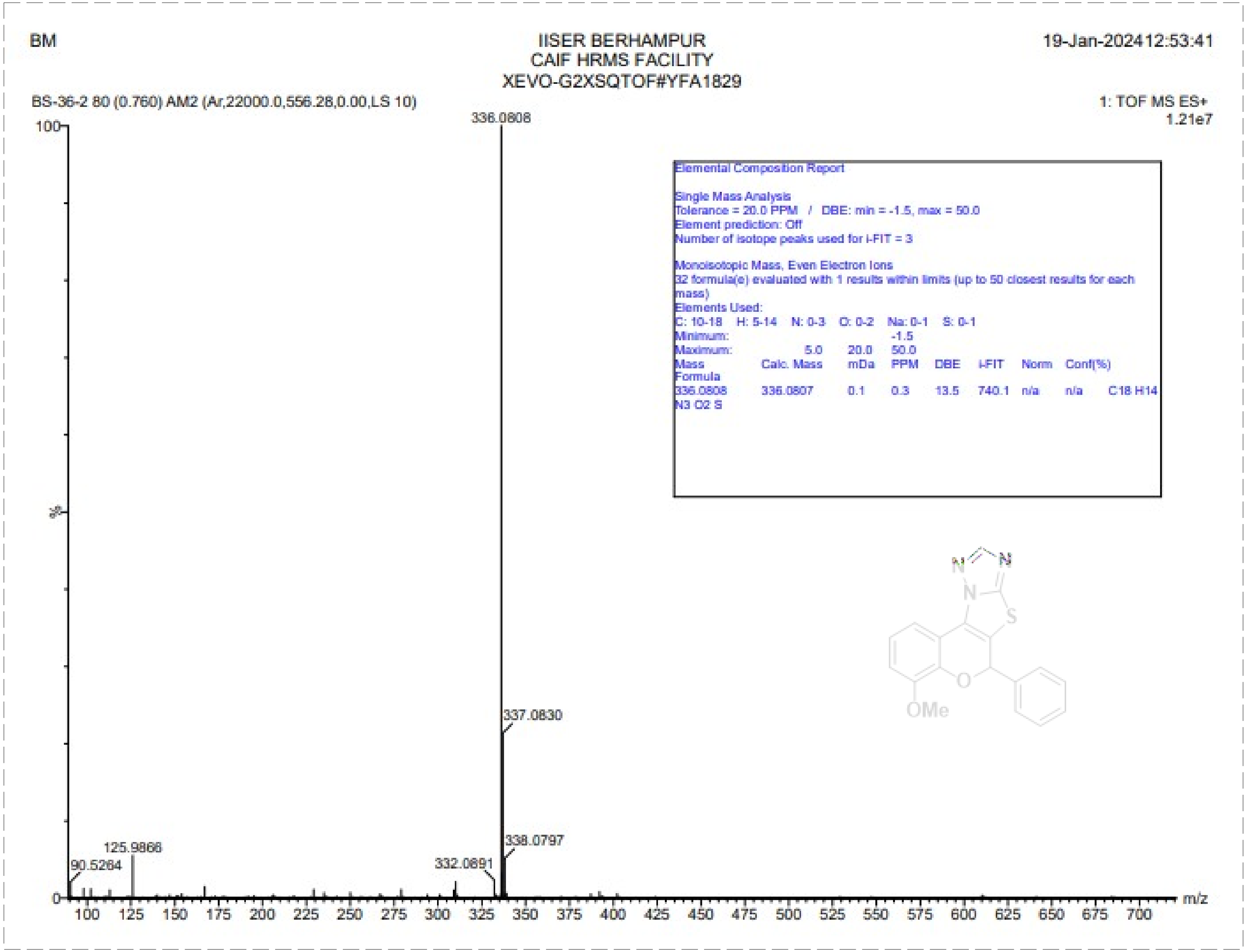

Fig S27. HRMS Spectrum of compound **4i**

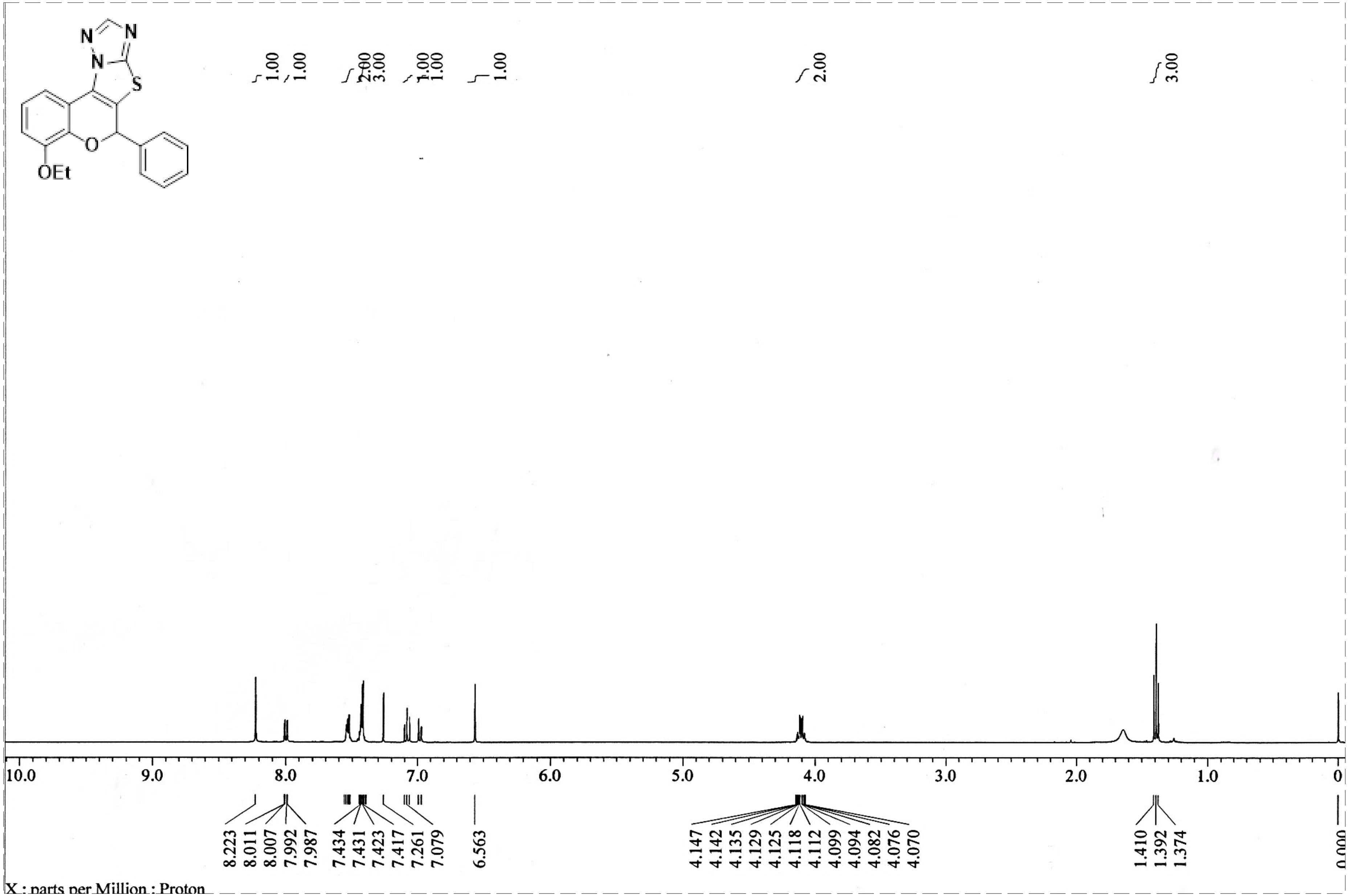

Fig S28. ^{1}H NMR Spectrum of compound **4j**

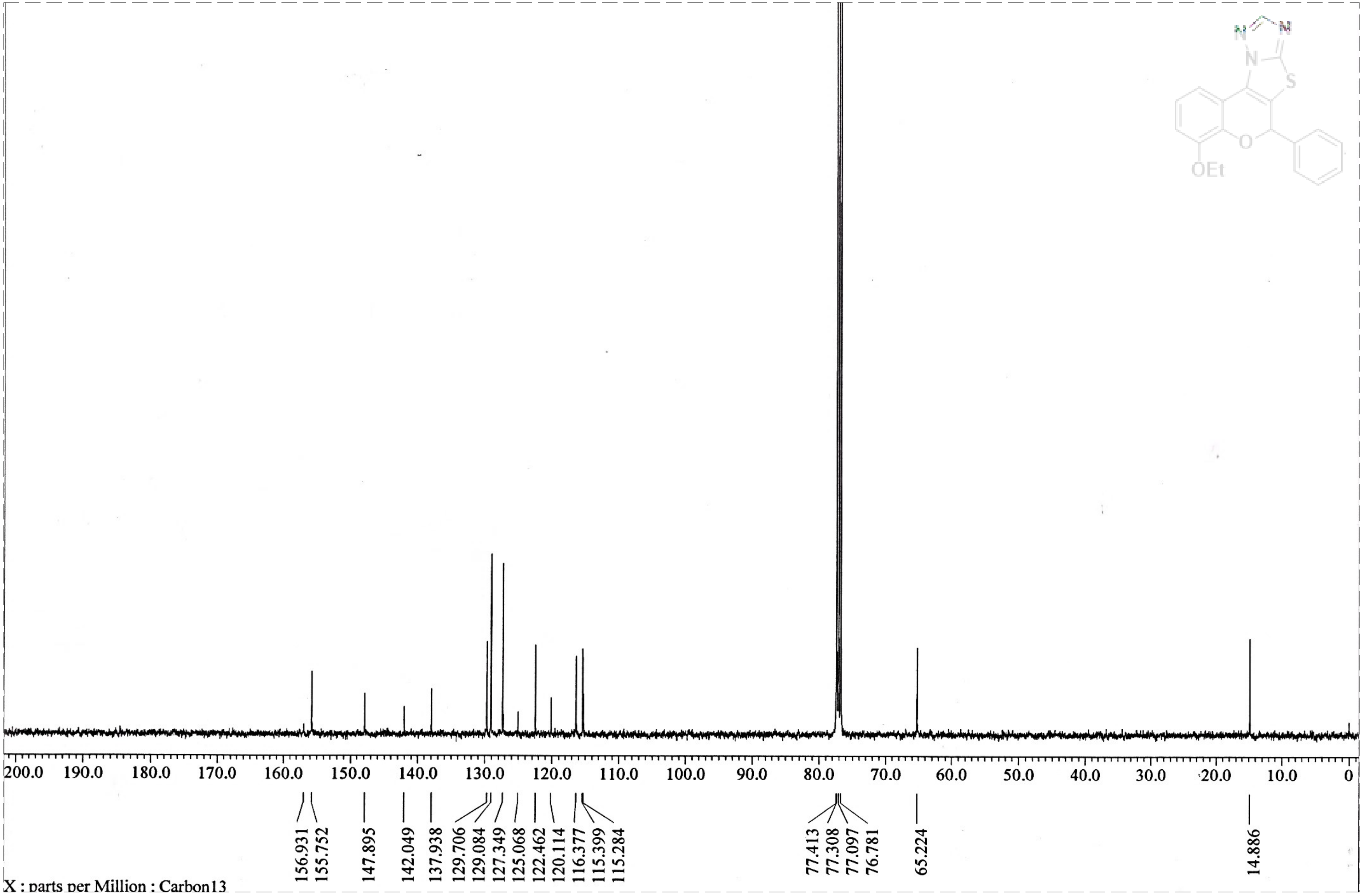

Fig S29. ^{13}C NMR Spectrum of compound **4j**

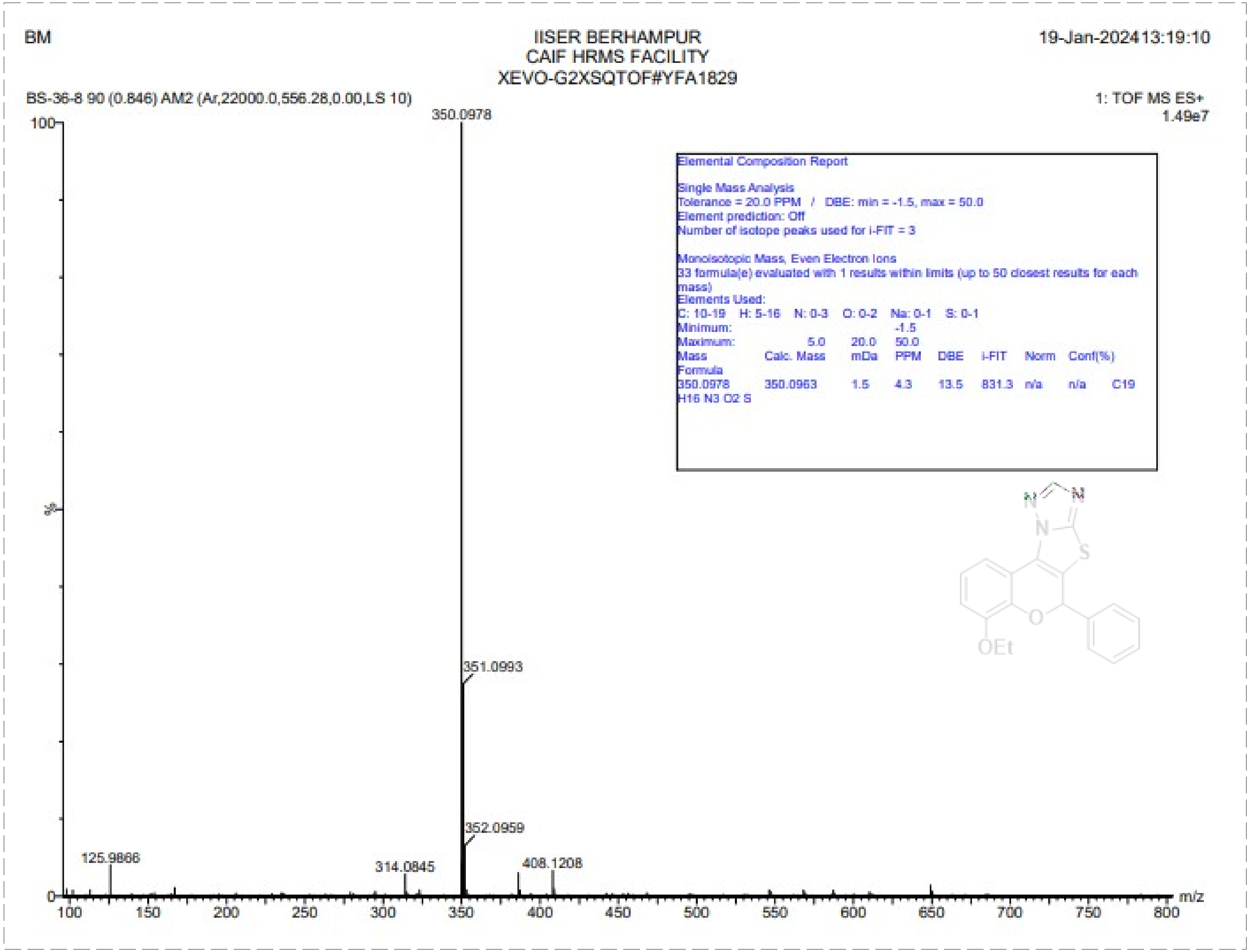

Fig S30. HRMS Spectrum of compound **4j**

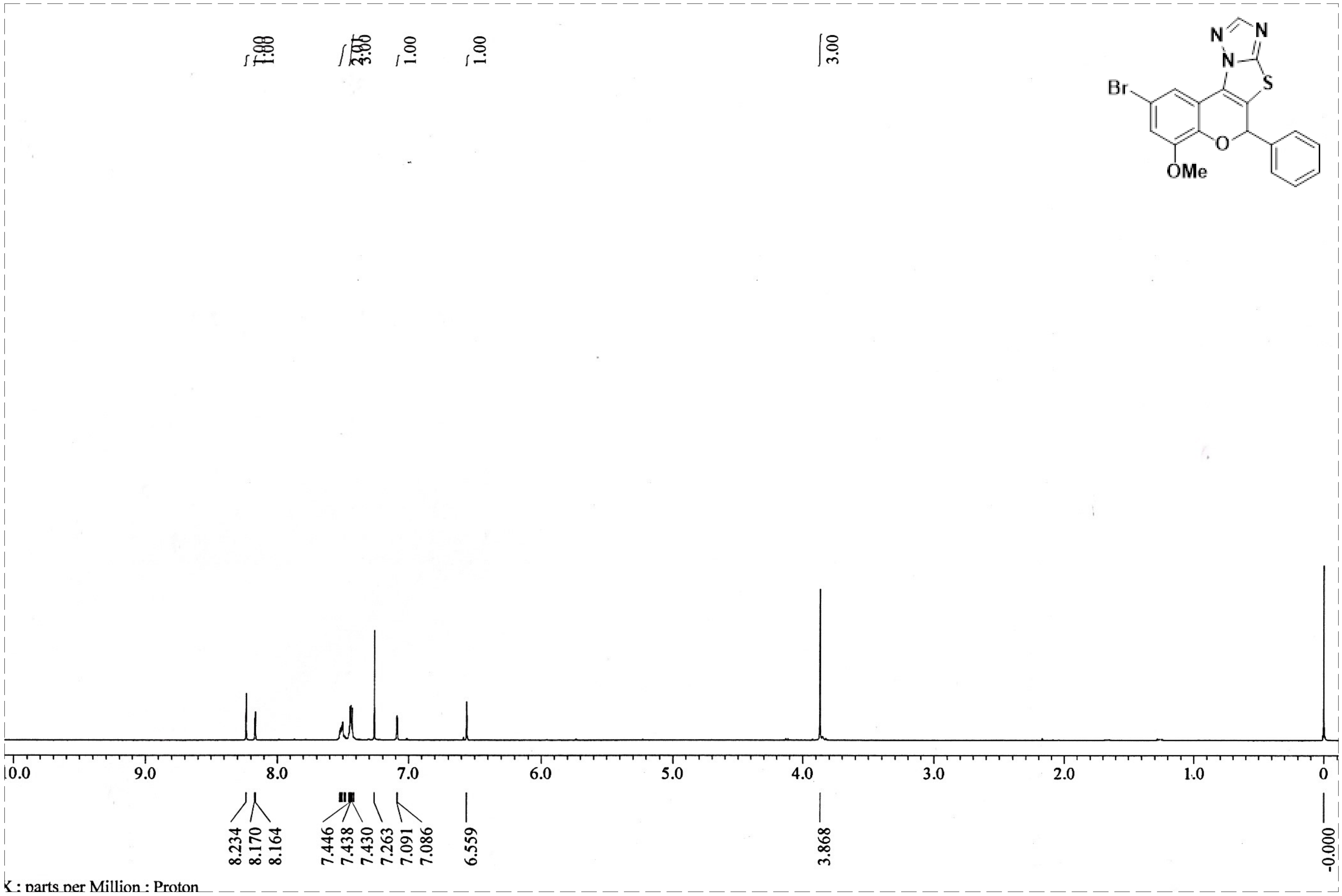

Fig S31. ^{1}H NMR Spectrum of compound **4k**

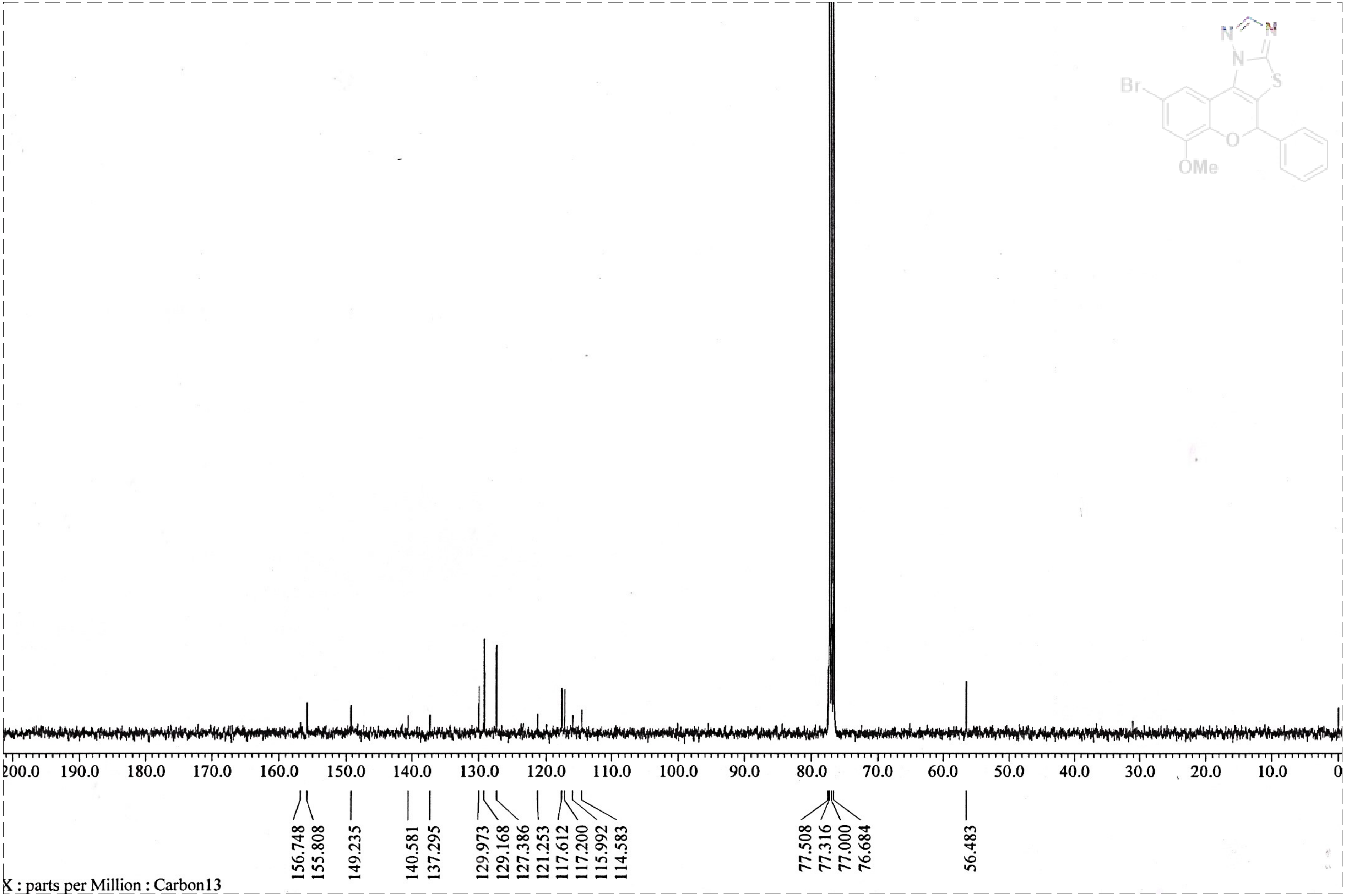

Fig S32. ^{13}C NMR Spectrum of compound **4k**

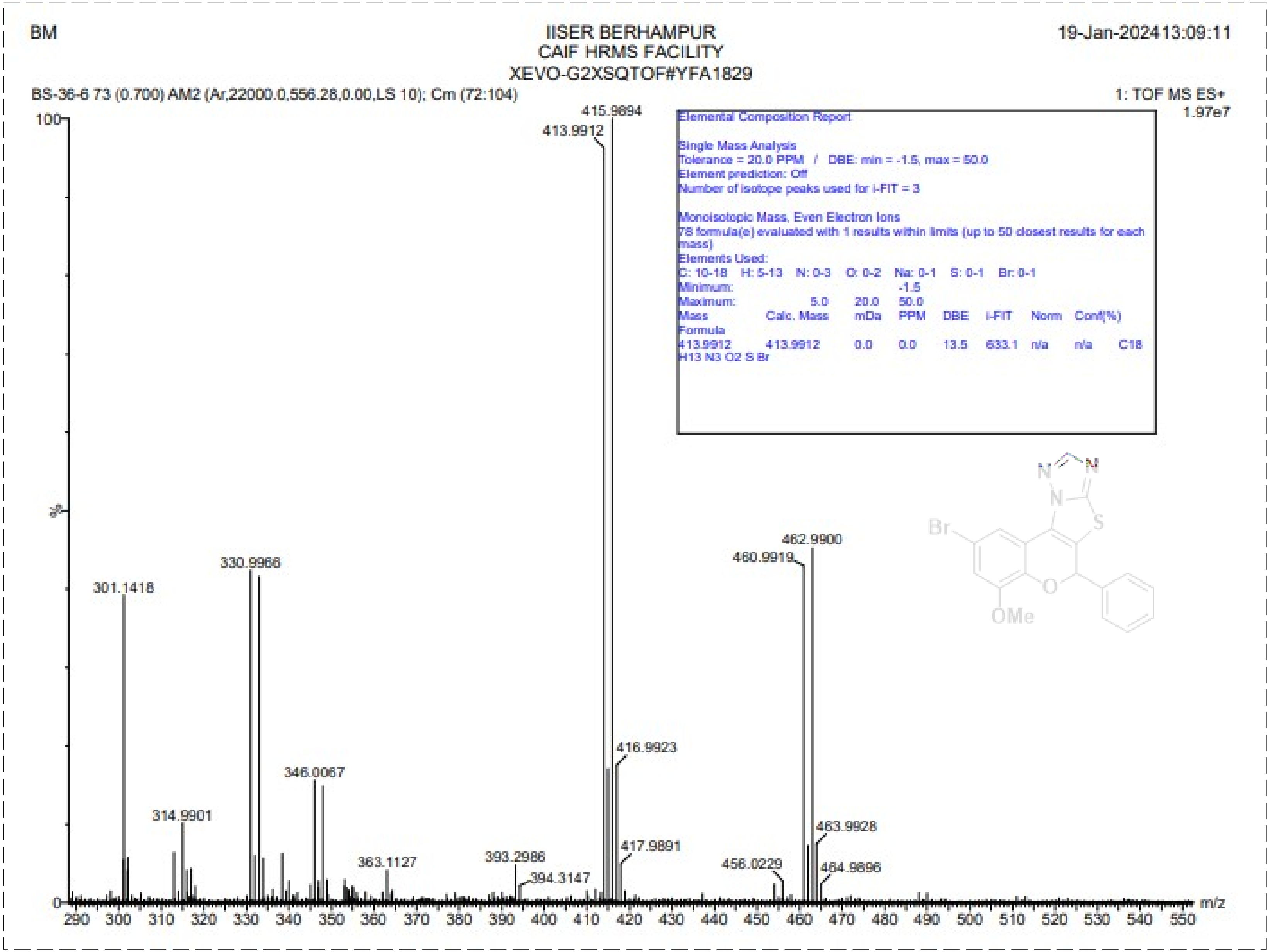

Fig S33. HRMS Spectrum of compound **4k**

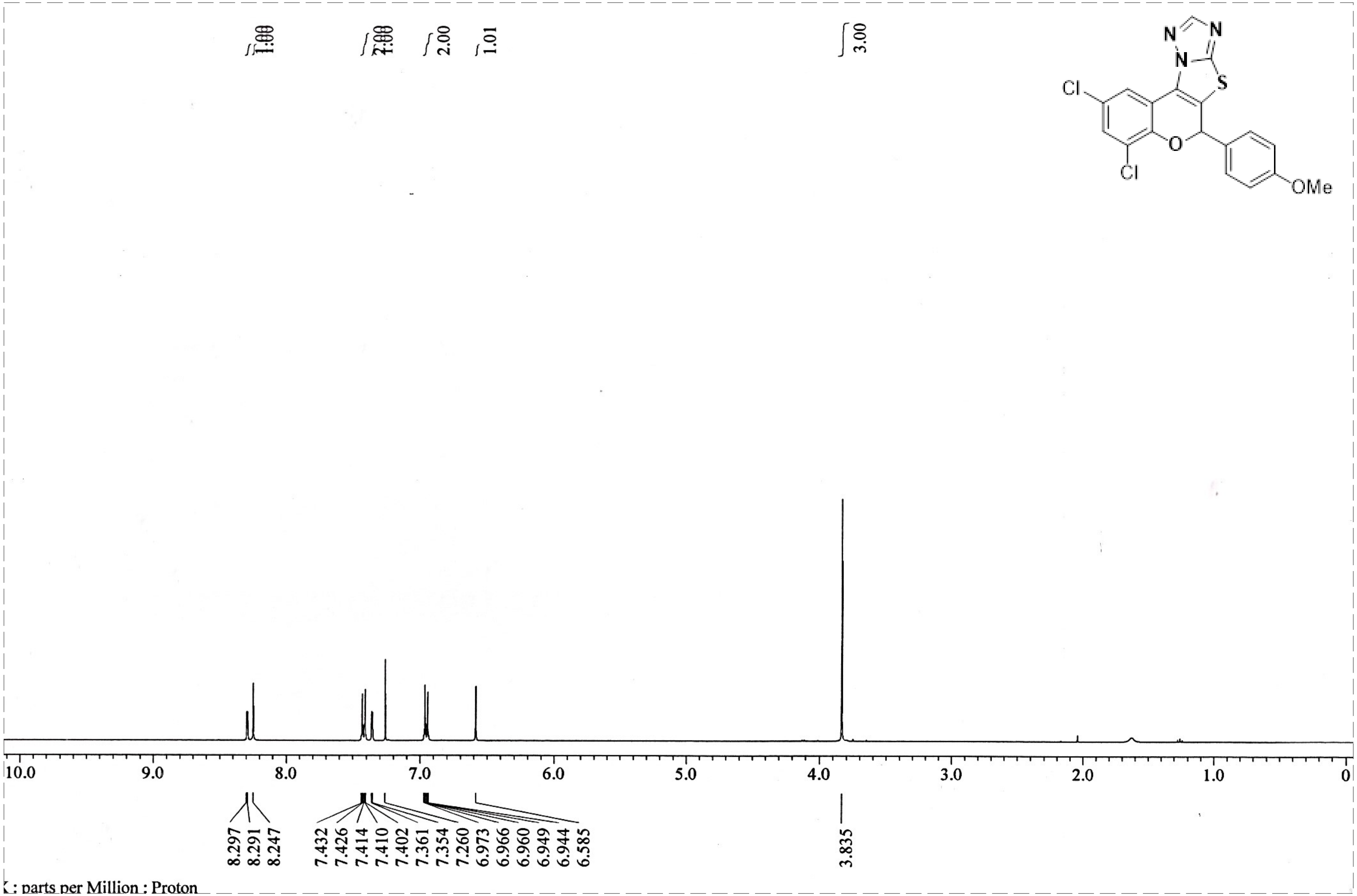

Fig S34. ^{1}H NMR Spectrum of compound **4l**

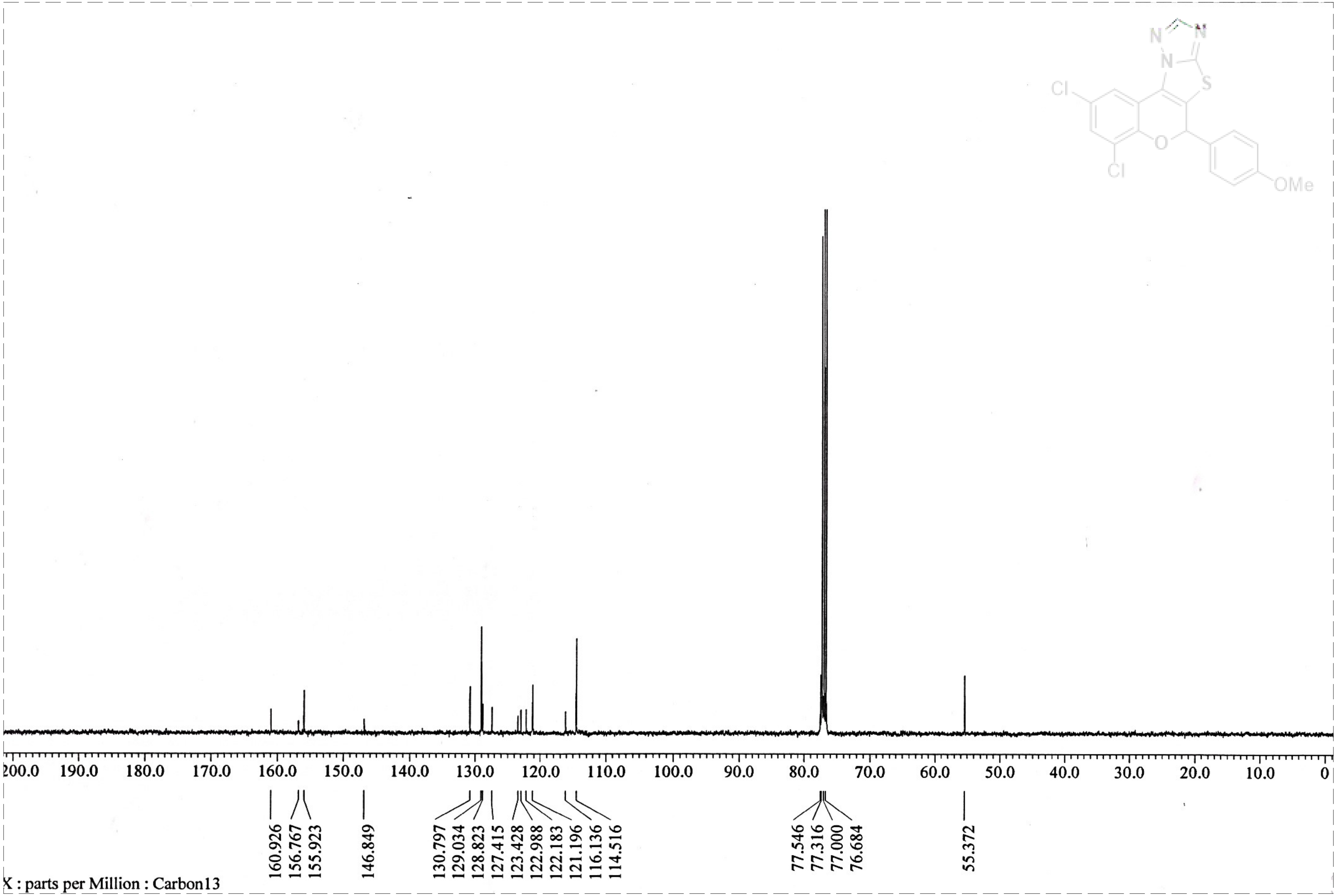

Fig S35. ^{13}C NMR Spectrum of compound **4l**

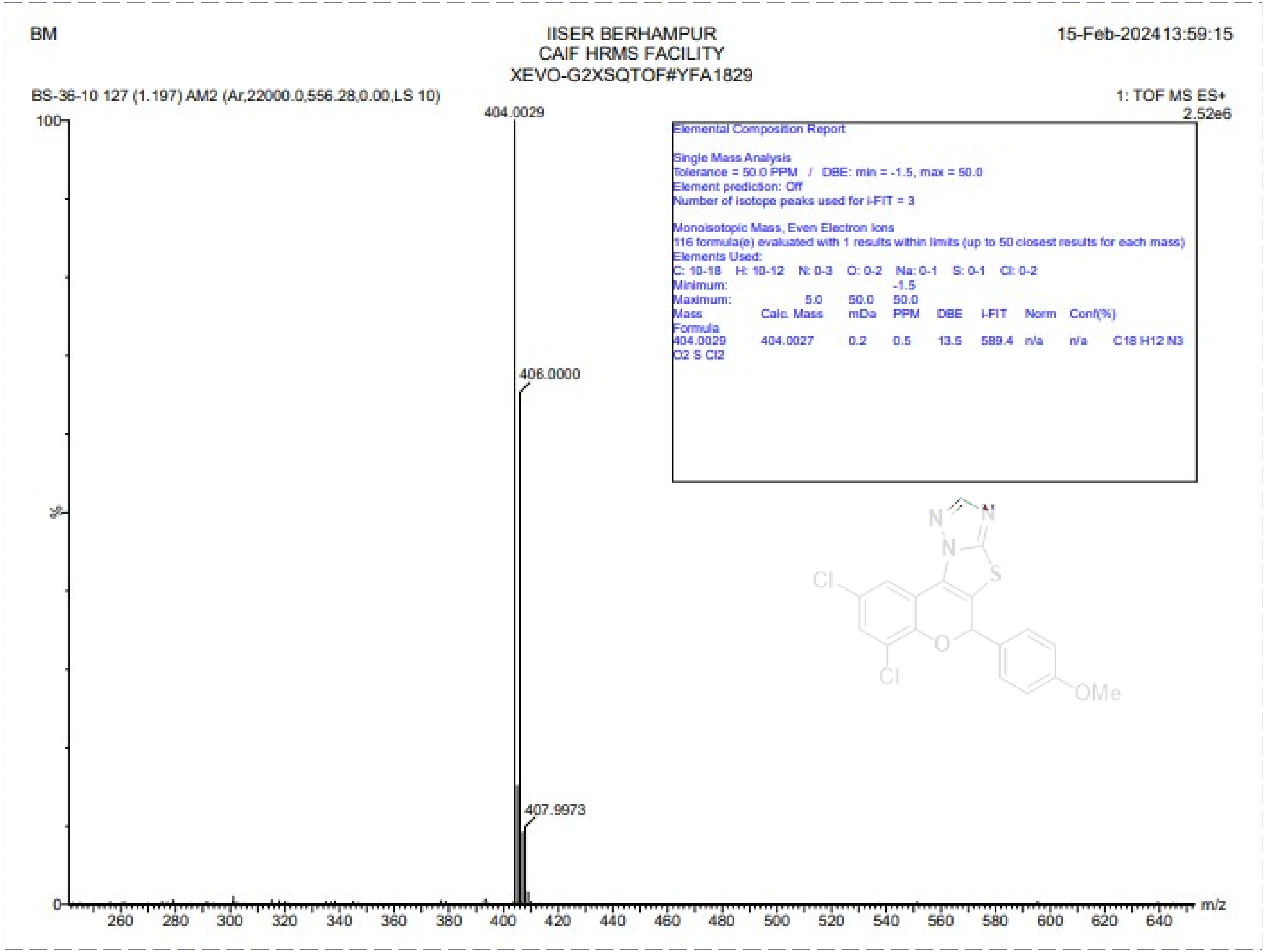

Fig S36. HRMS Spectrum of compound **4l**

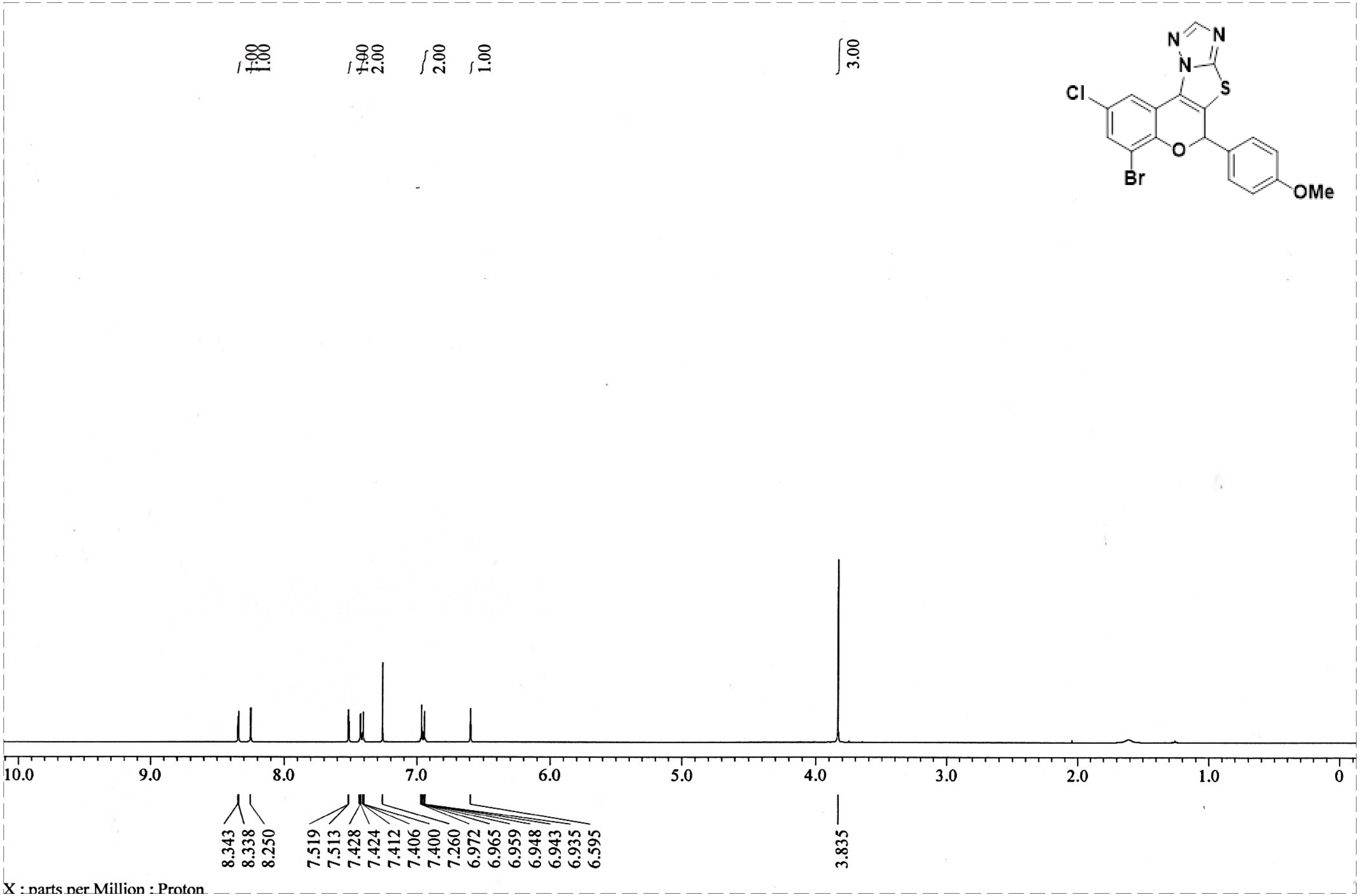

Fig S37. ^{1}H NMR Spectrum of compound **4m**

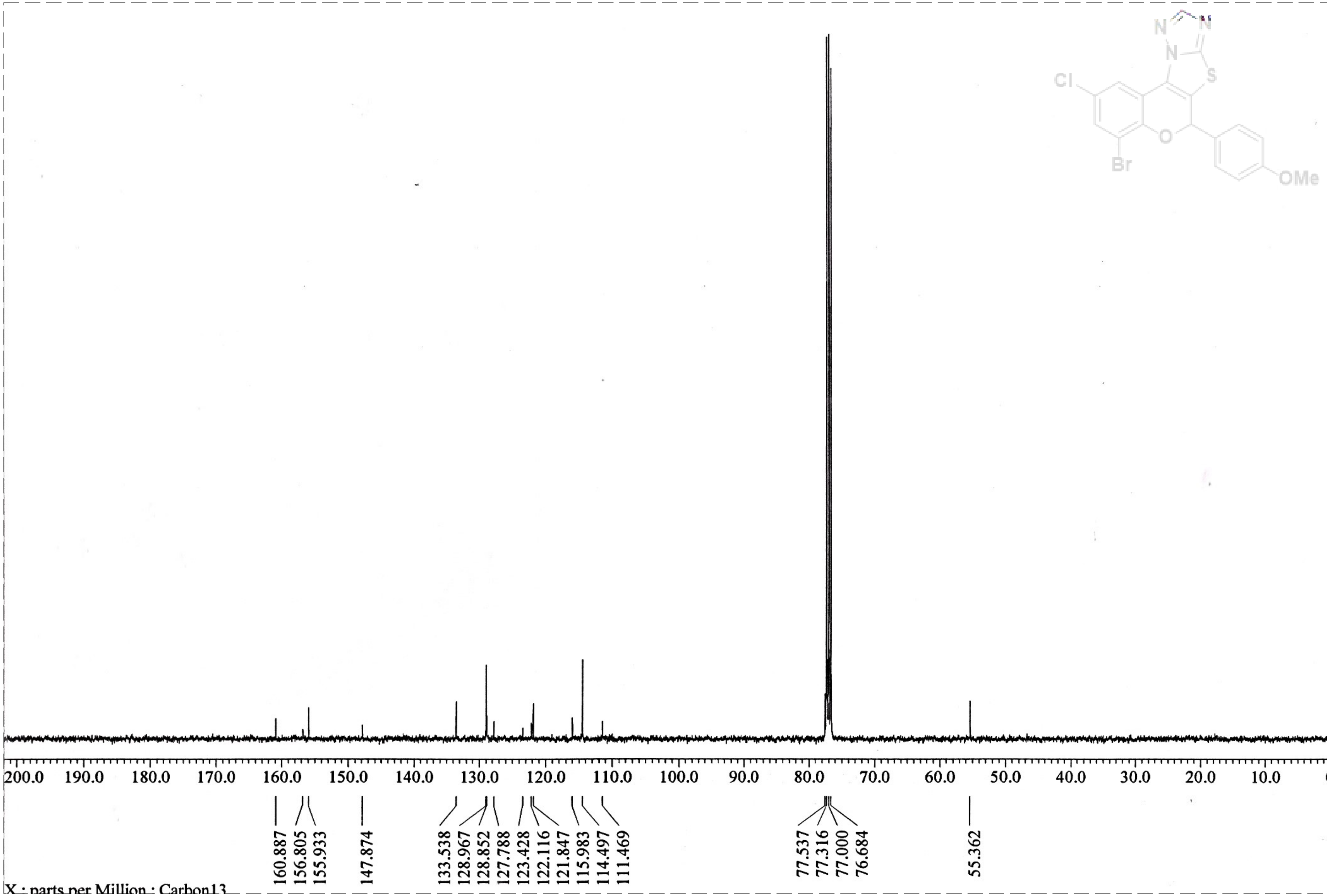

Fig S38. ^{13}C NMR Spectrum of compound **4m**

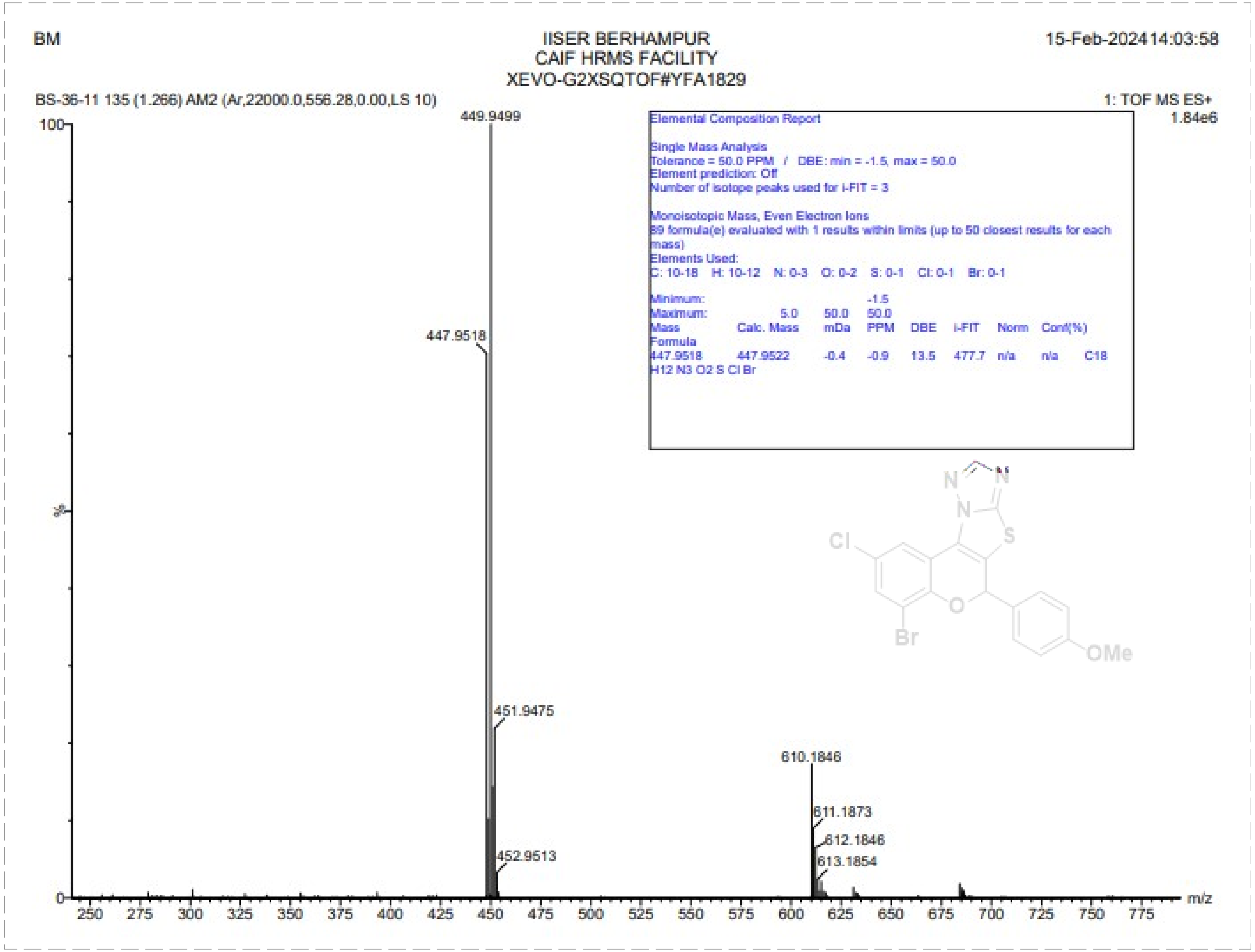

Fig S39. HRMS Spectrum of compound **4m**

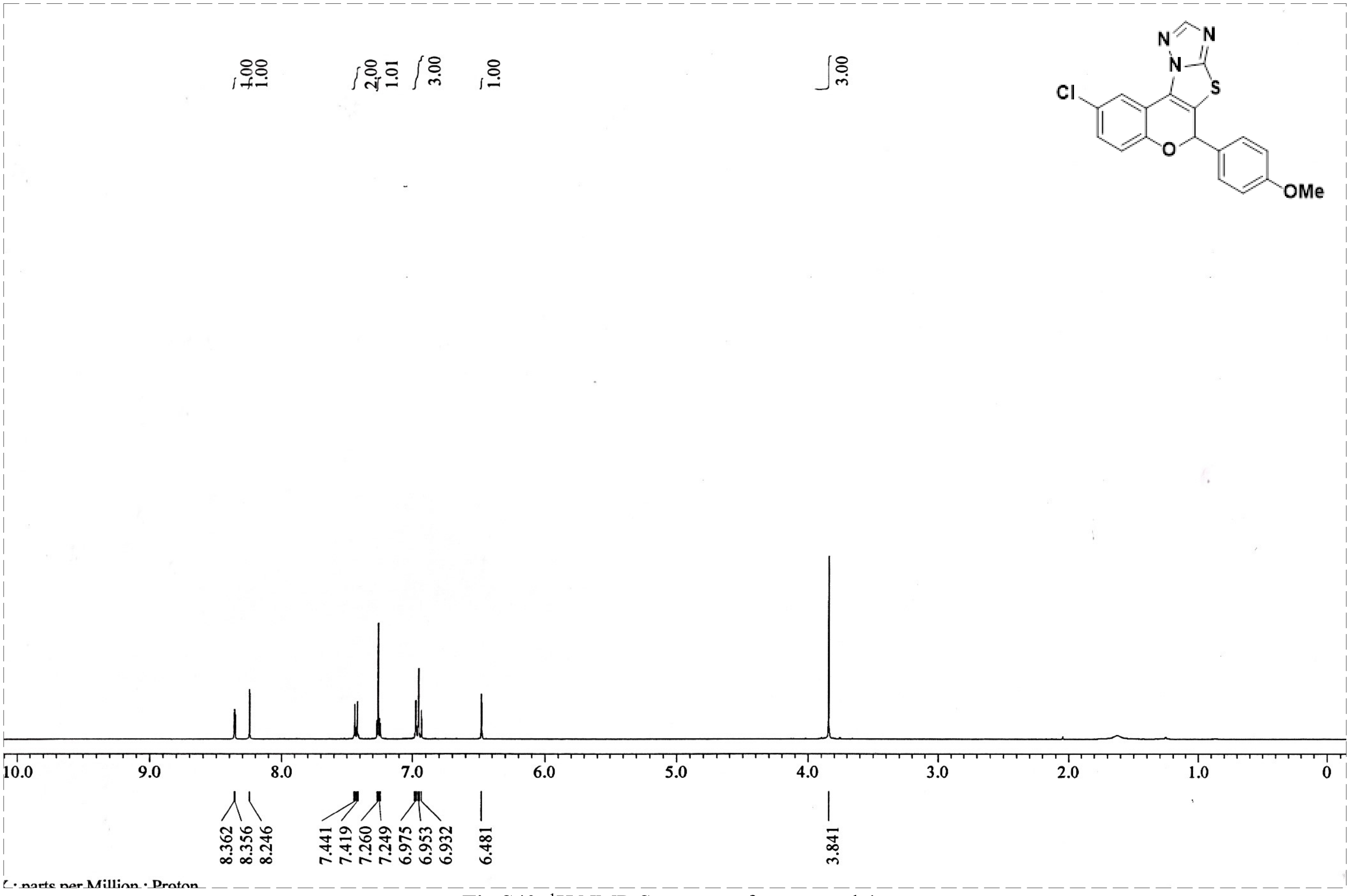

Fig S40. ^{1}H NMR Spectrum of compound **4n**

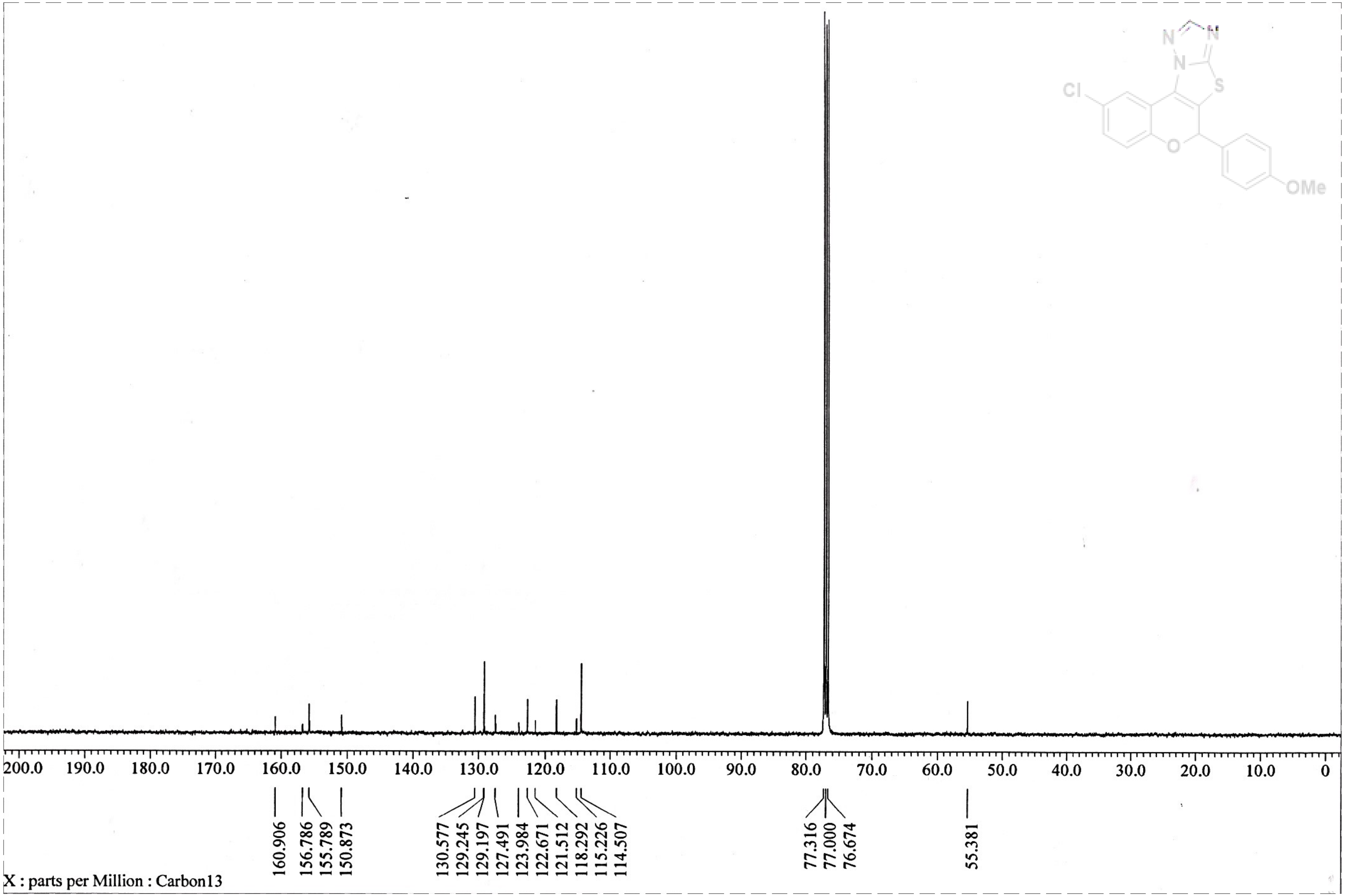

Fig S41. ^{13}C NMR Spectrum of compound **4n**

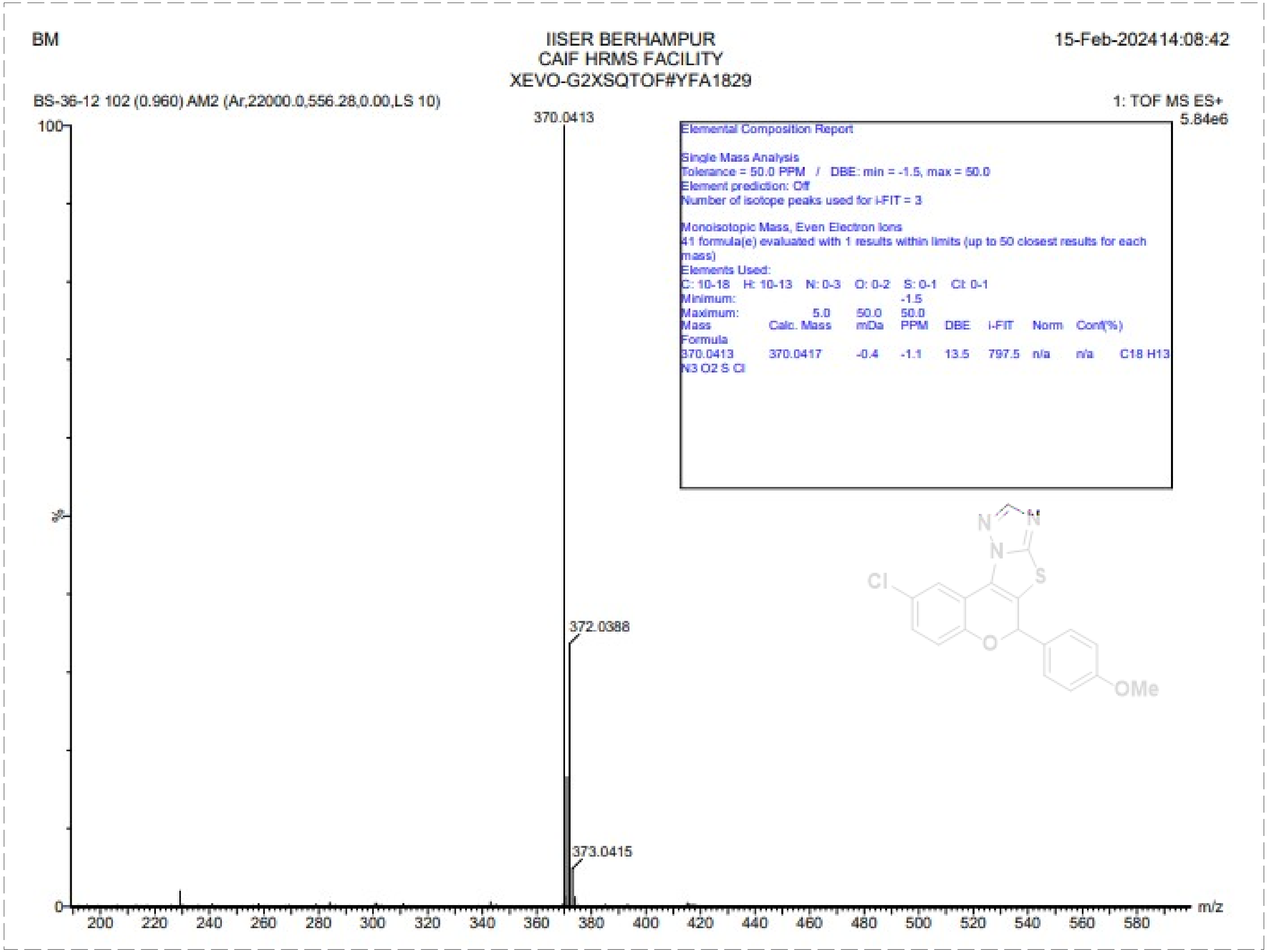

Fig S42. HRMS Spectrum of compound **4n**

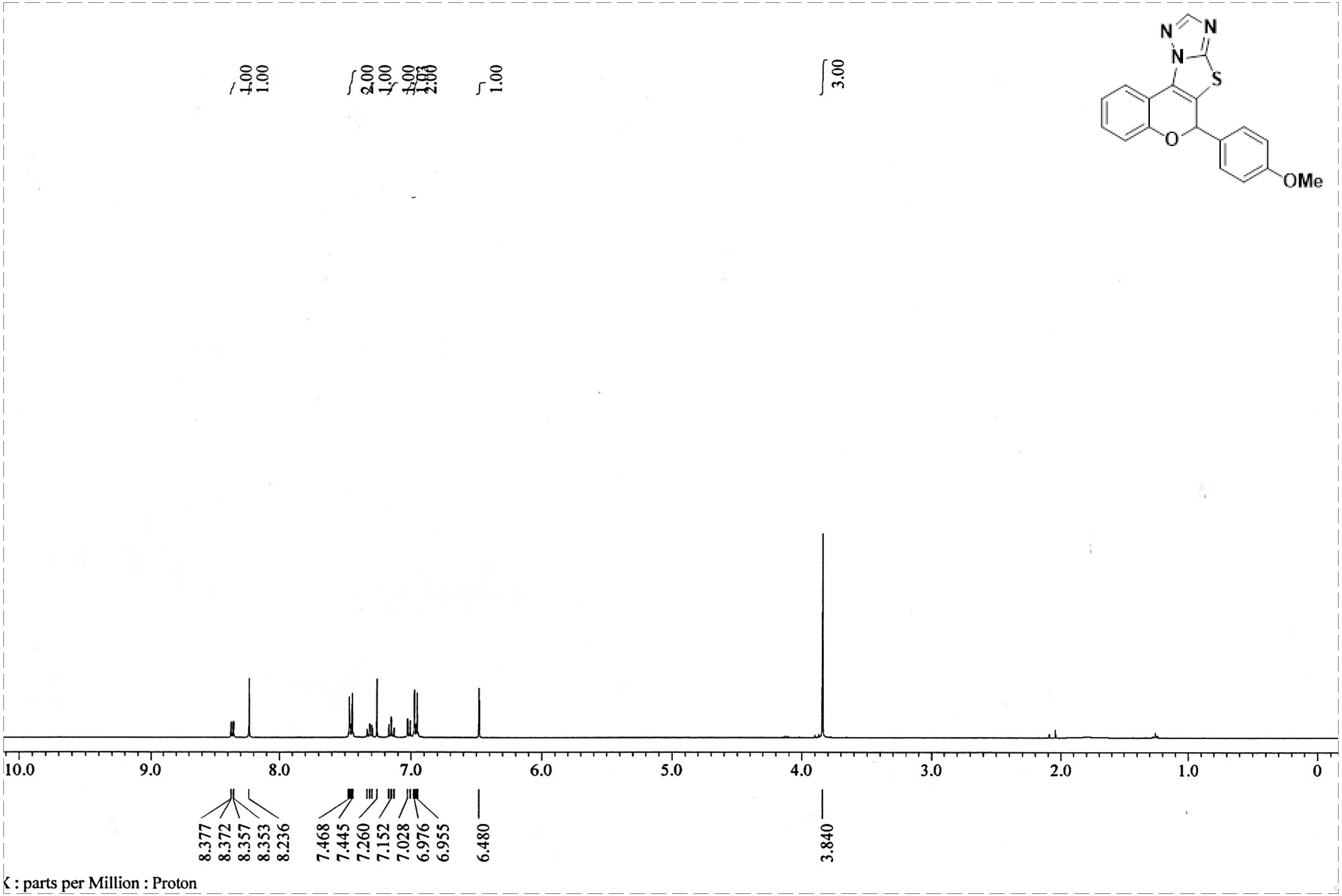

Fig S43. ^{1}H NMR Spectrum of compound **4o**

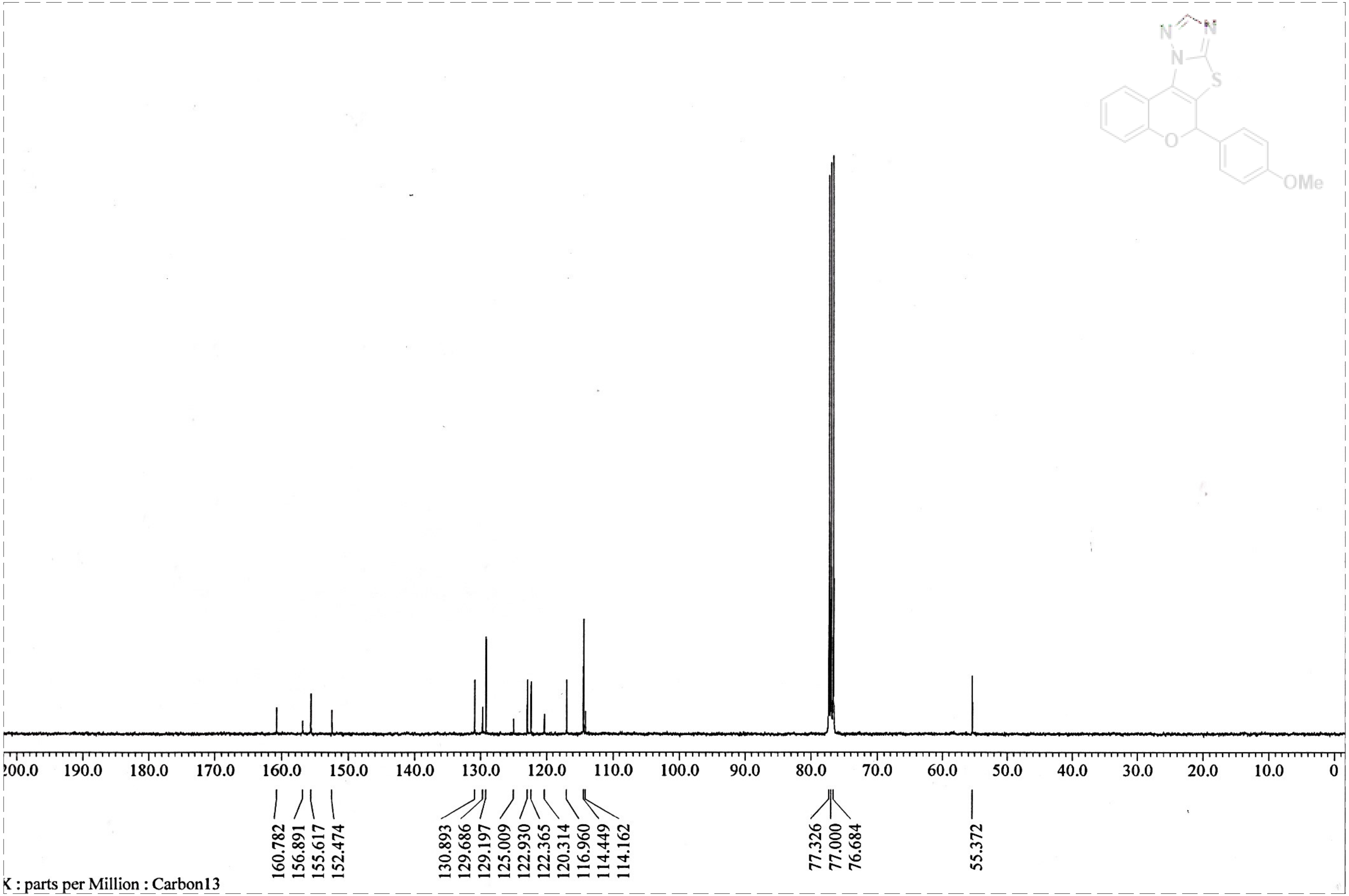

Fig S44. ^{13}C NMR Spectrum of compound **4o**

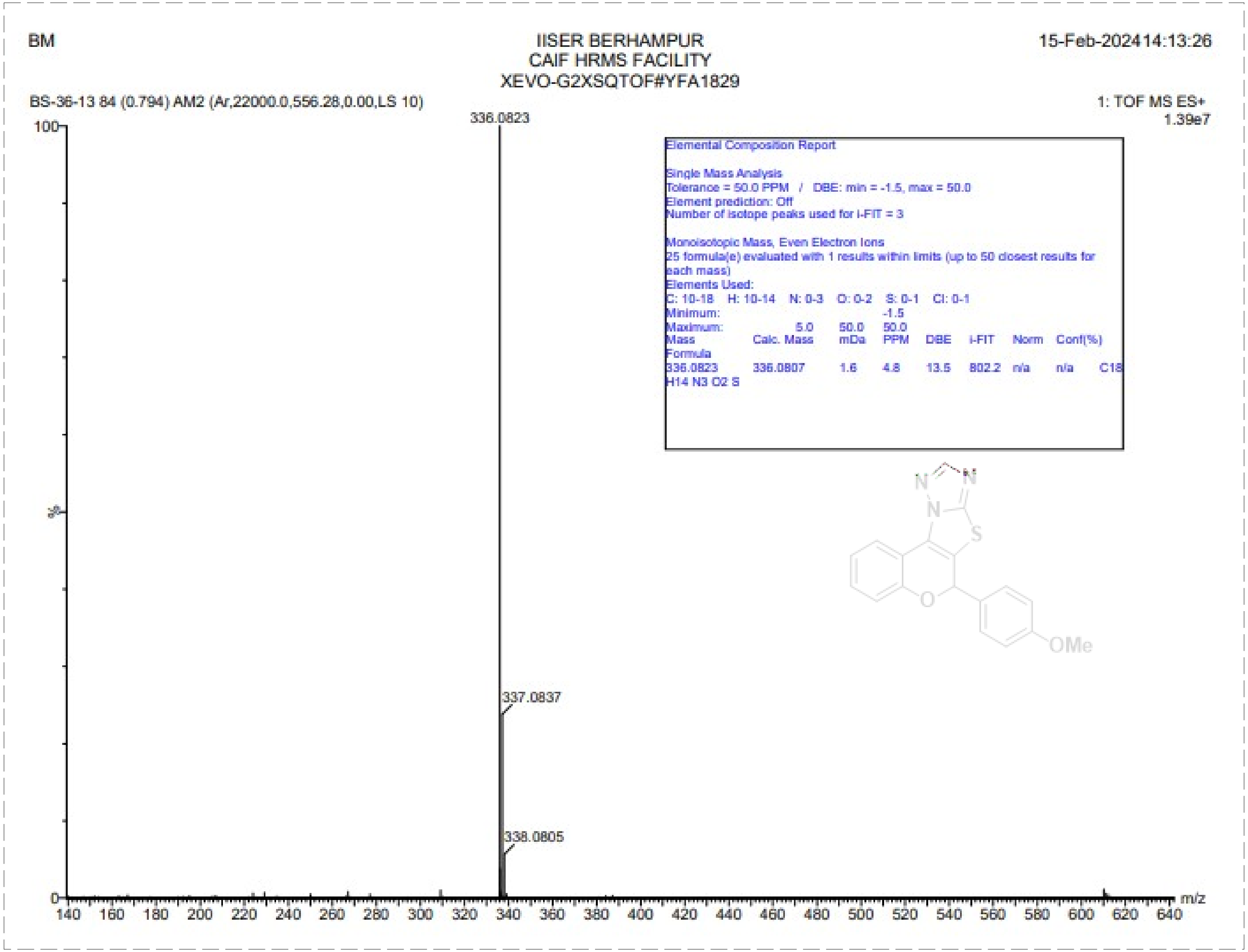

Fig S45. HRMS Spectrum of compound **4o**

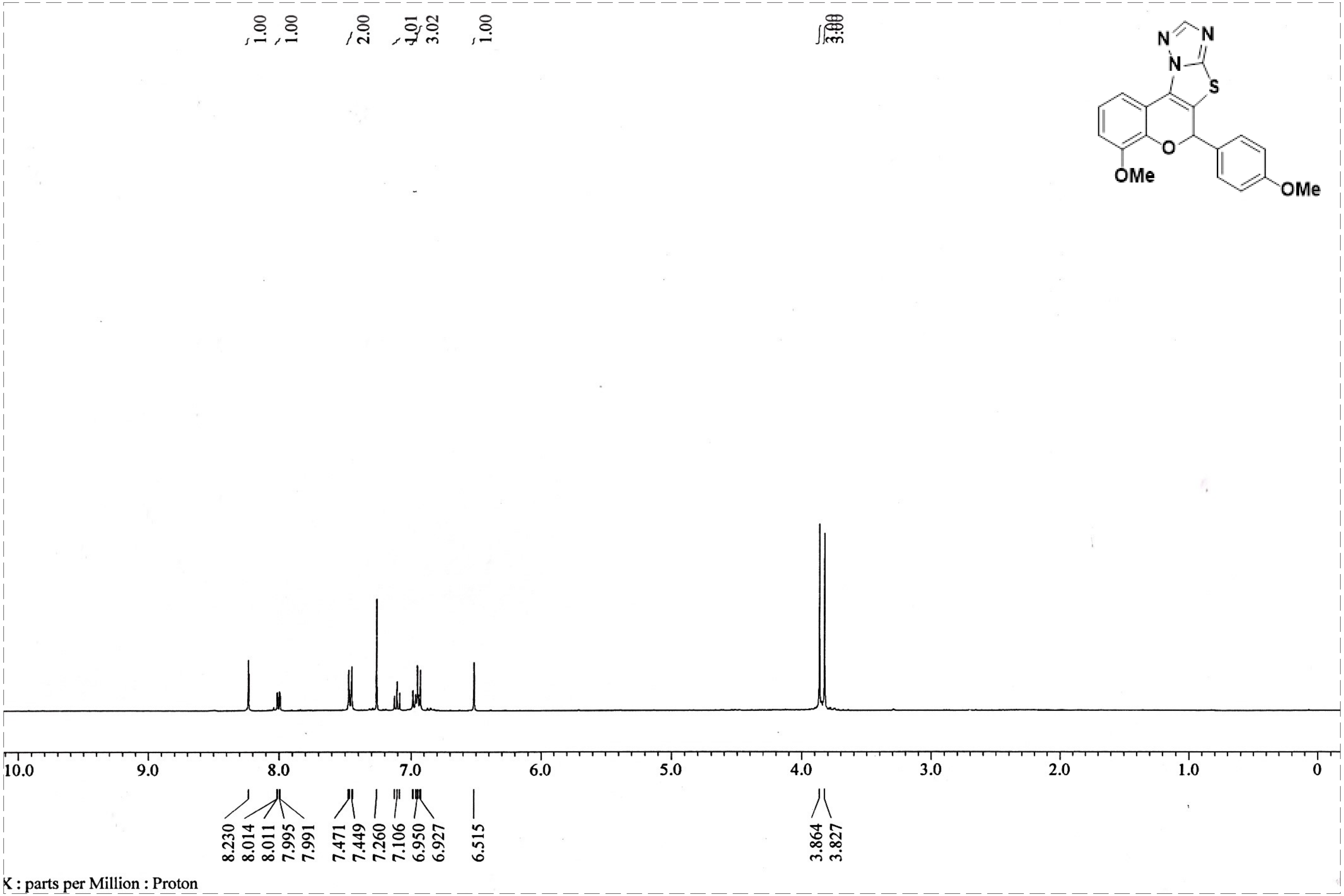

Fig S46. ^{1}H NMR Spectrum of compound **4p**

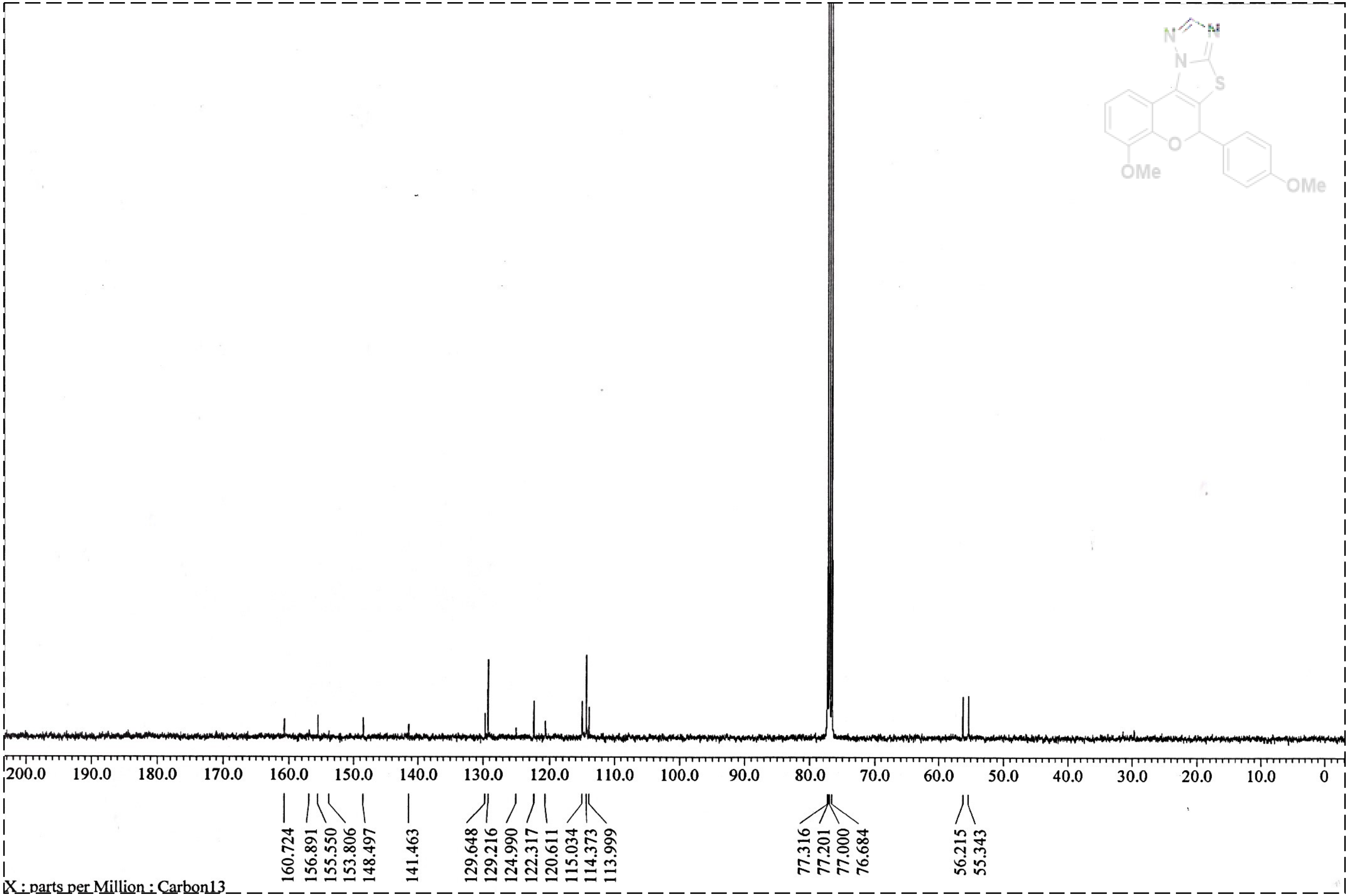

Fig S47. ^{13}C NMR Spectrum of compound **4p**

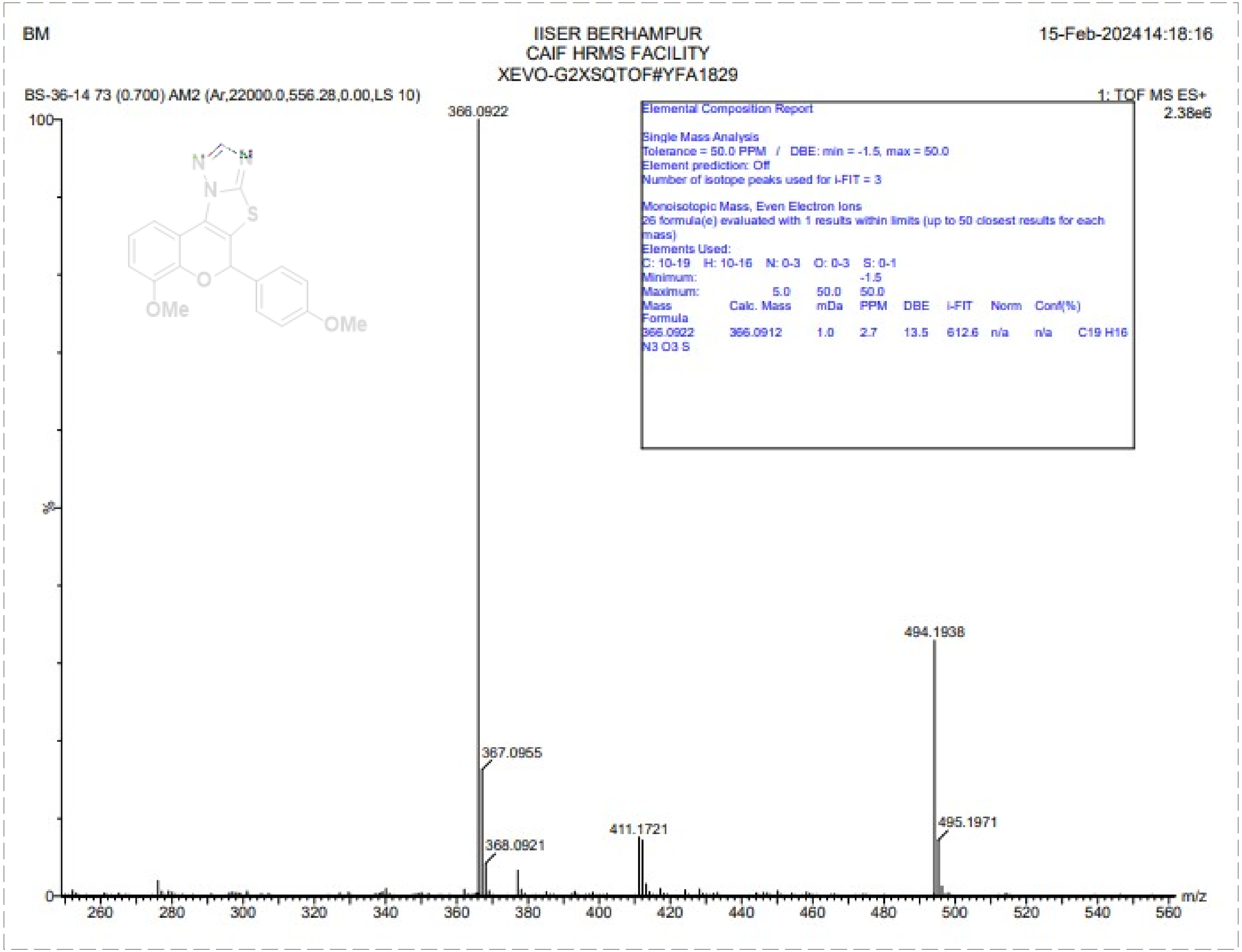

Fig S48. HRMS Spectrum of compound **4p**

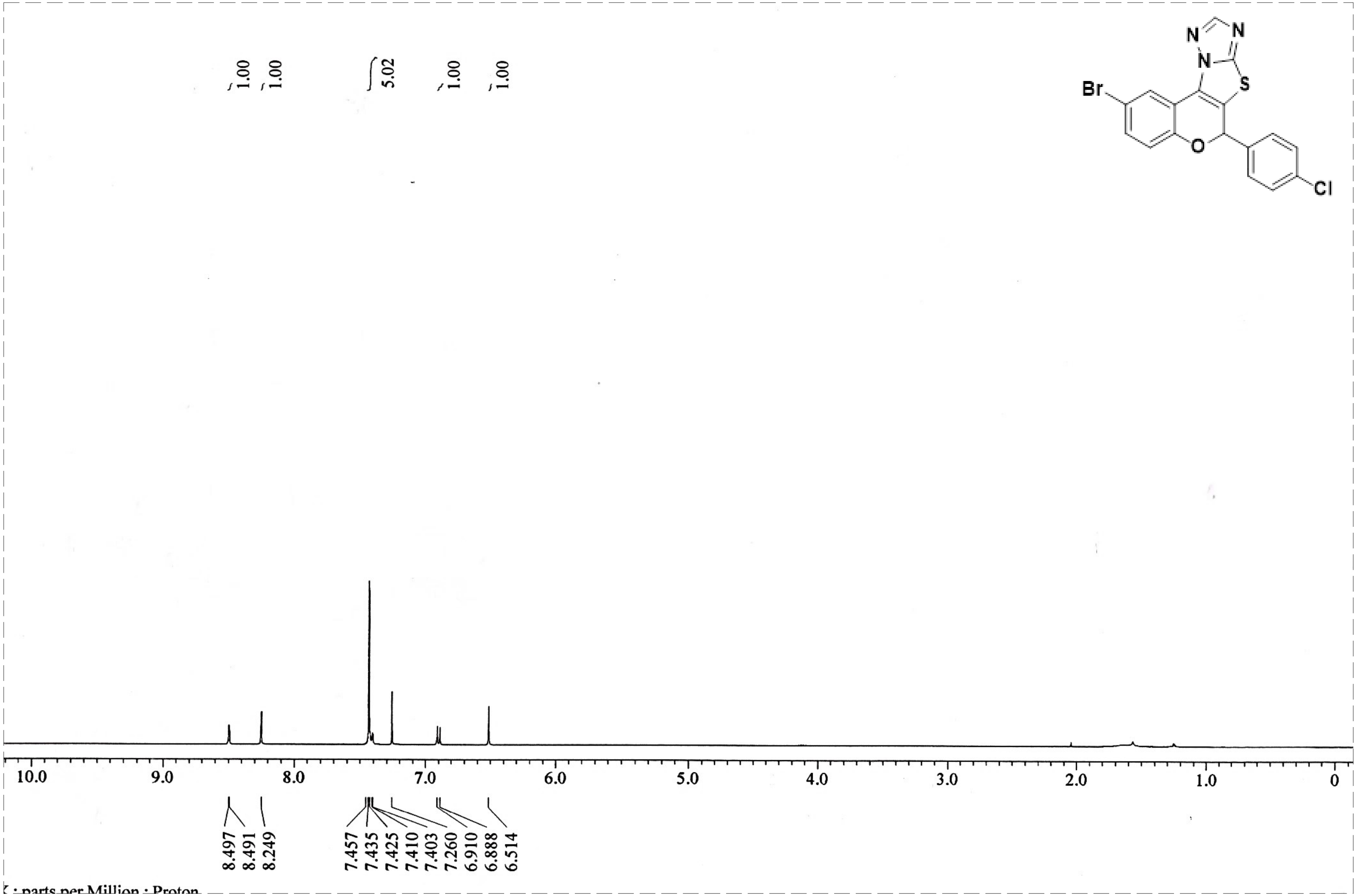

Fig S49. ^{1}H NMR Spectrum of compound **4q**

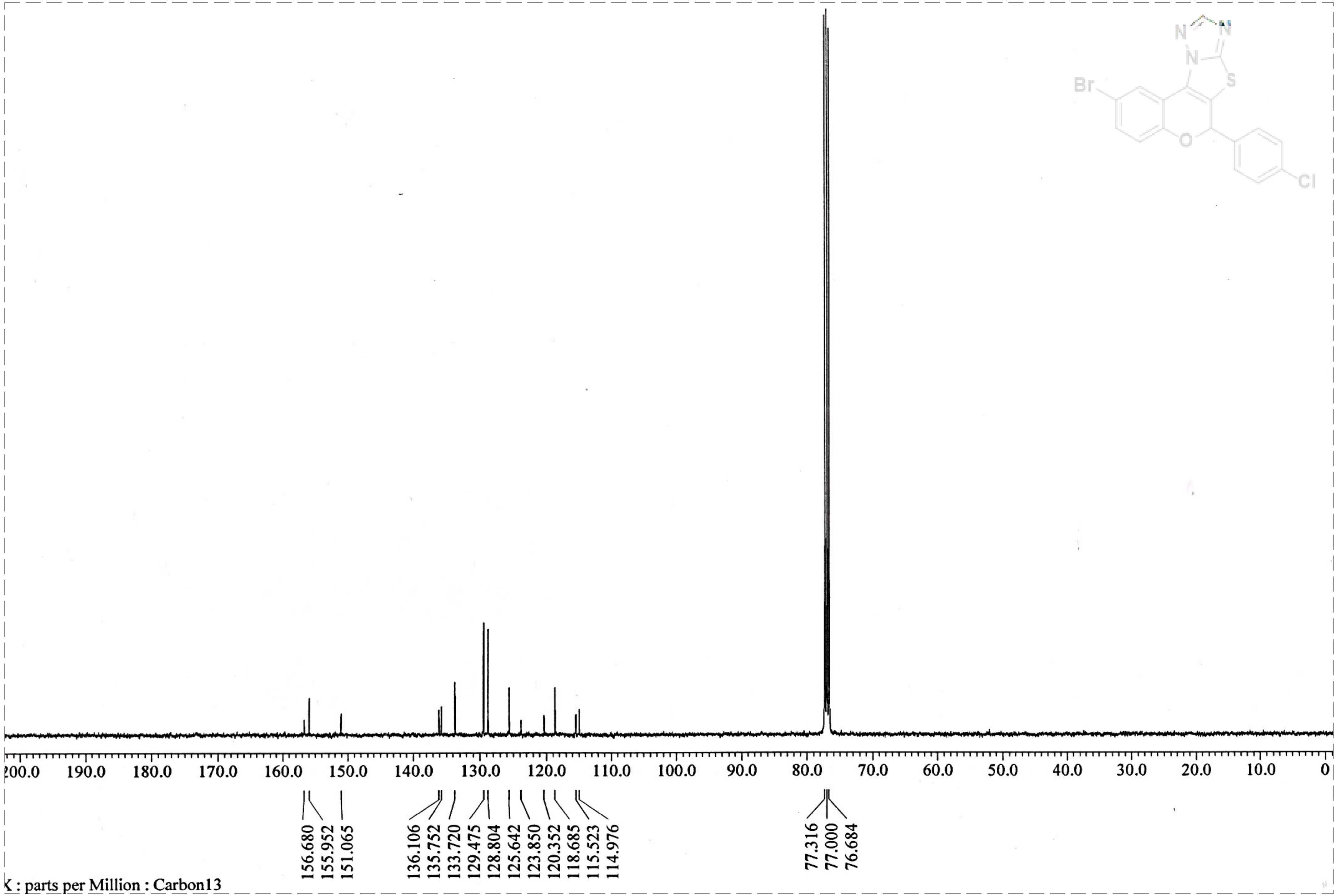

Fig S50. ^{13}C NMR Spectrum of compound **4q**

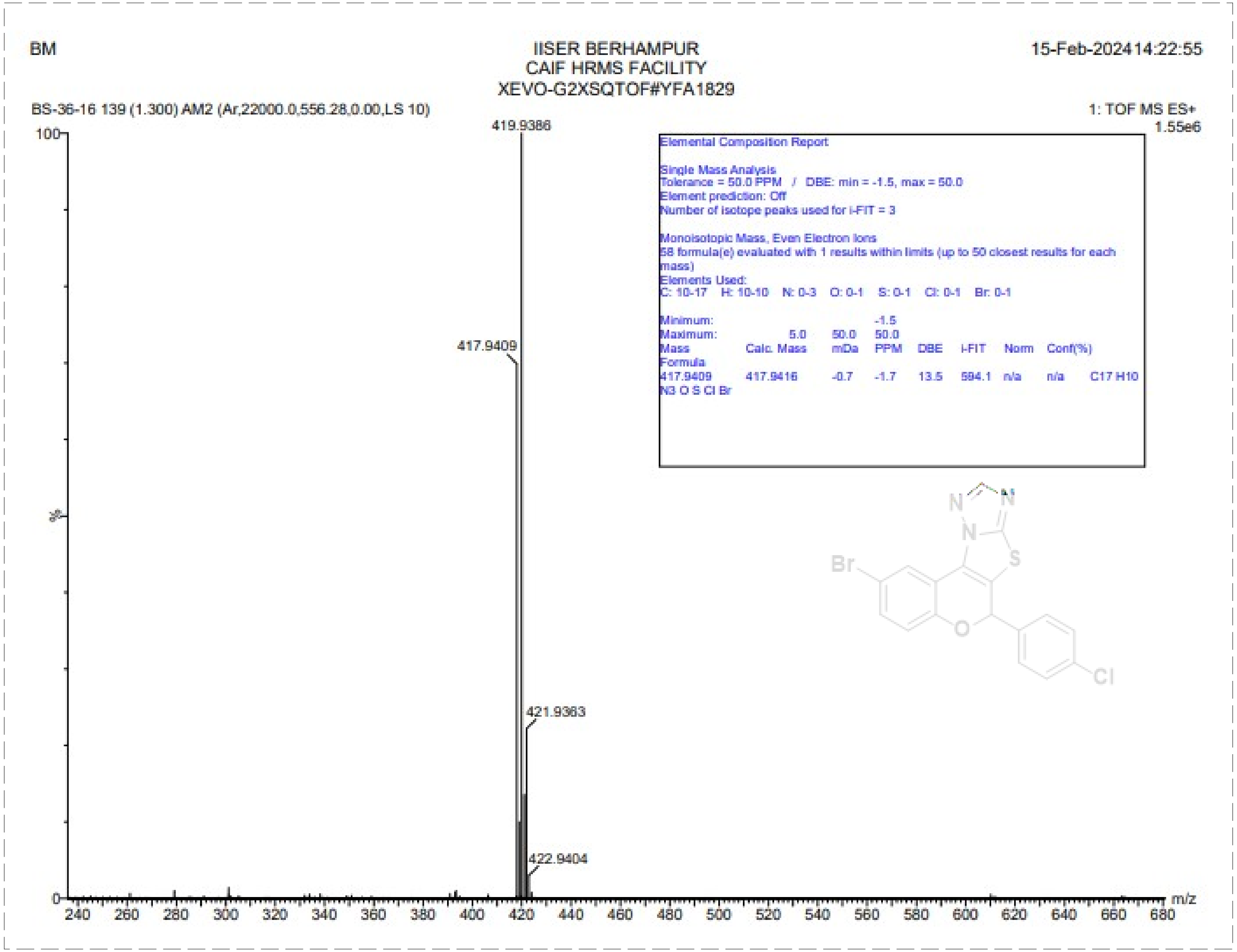

Fig S51. HRMS Spectrum of compound **4q**

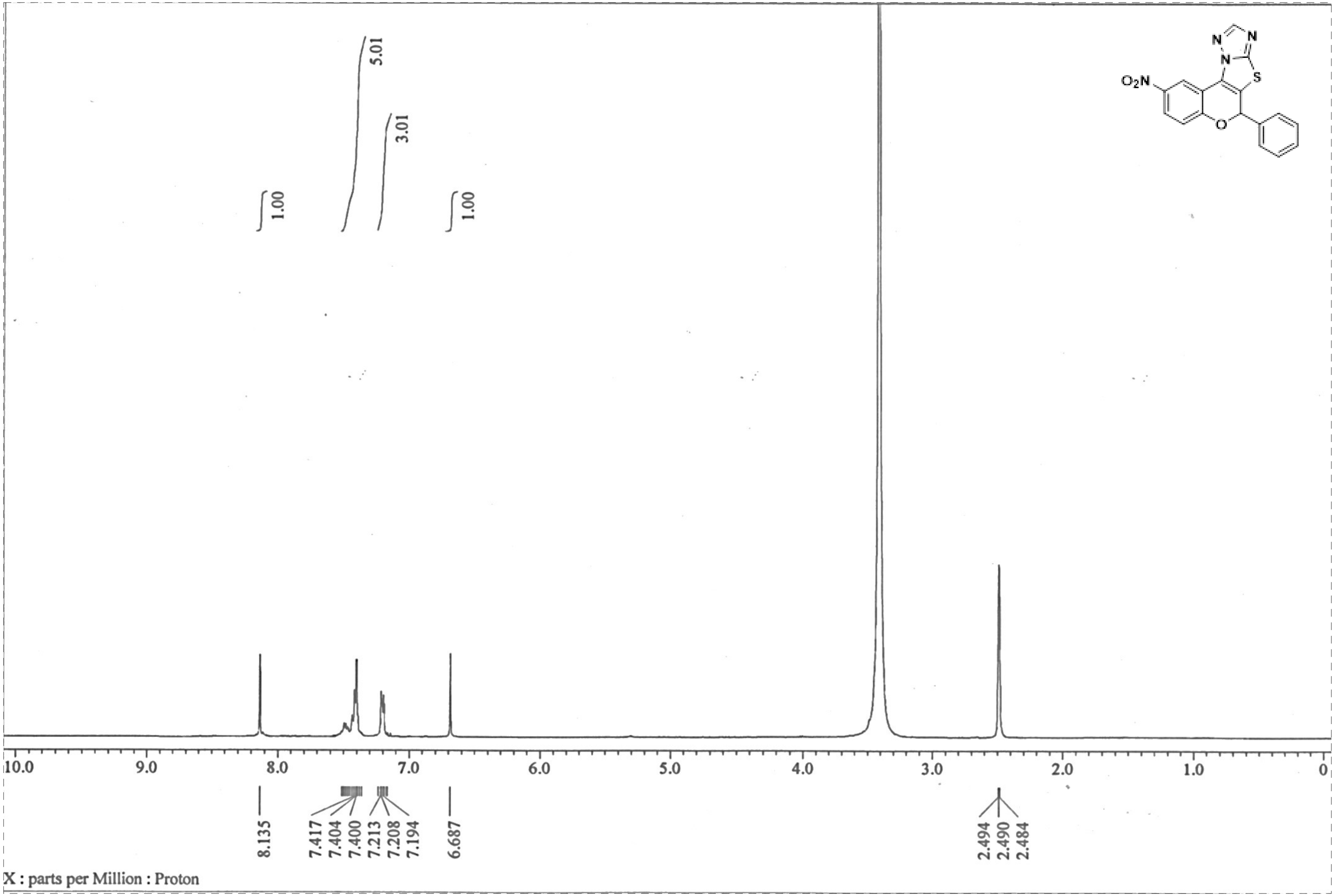

Fig S52. ^{1}H NMR Spectrum of compound **4r**

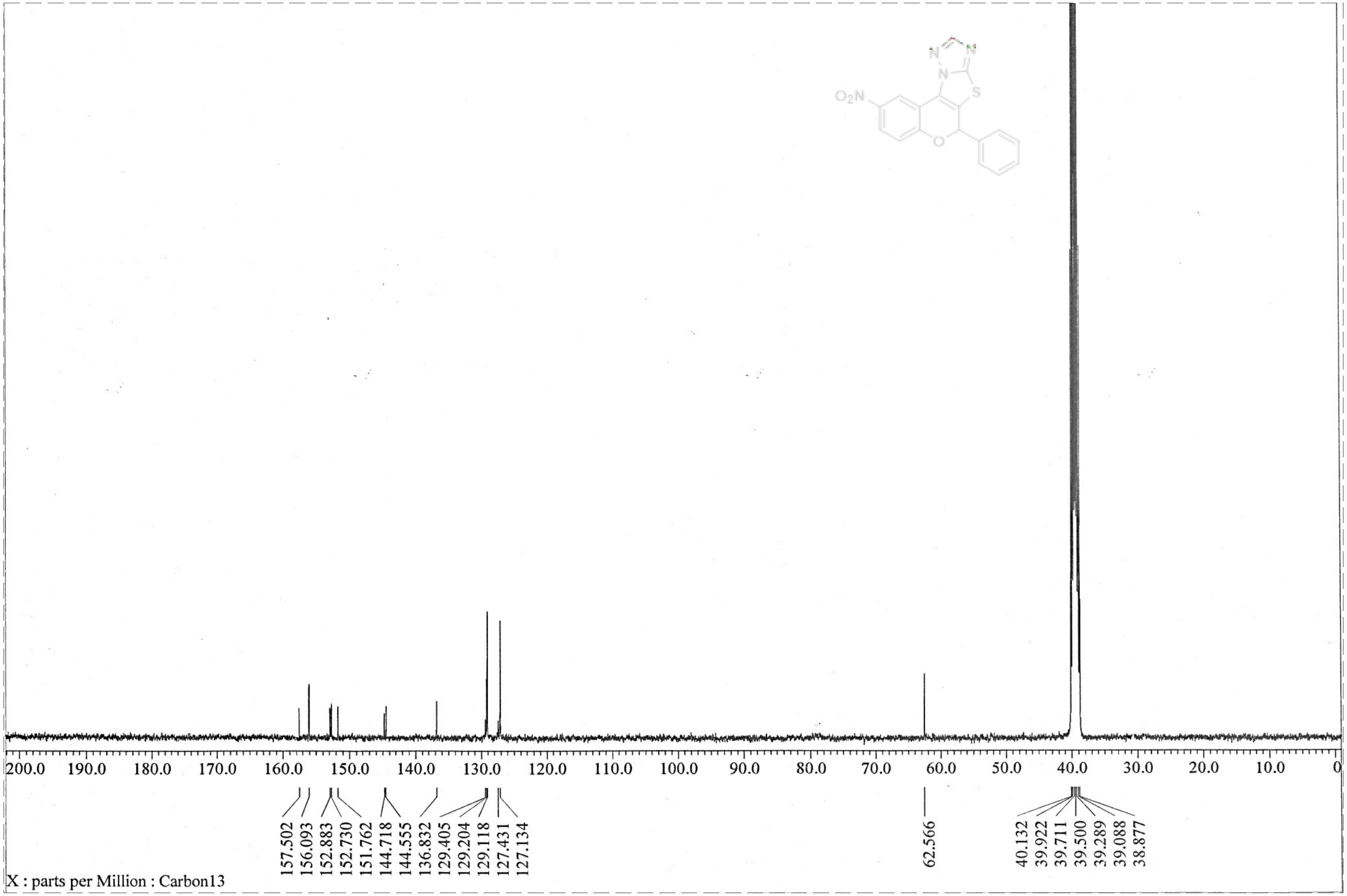

Fig S53. ^{13}C NMR Spectrum of compound **4r**

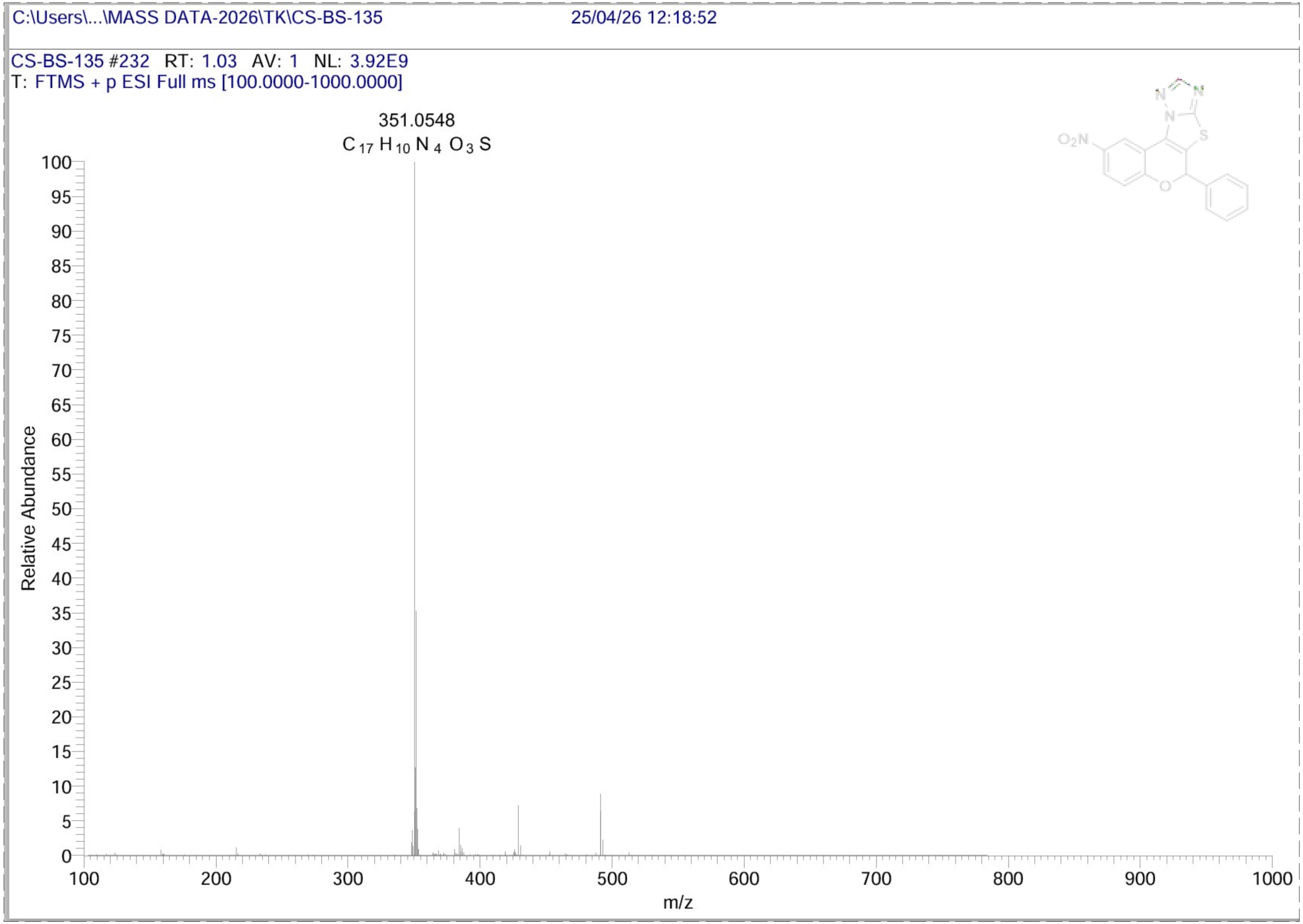

Fig S54. HRMS Spectrum of compound **4r**

4. Biological activity evaluations

4.1. *In vitro anticancer activity*

4.1.1. Reagents, materials, and instrumentation

Dulbecco′s Modified Eagle Medium (DMEM) (Gibco BRL, UK), Fetal Bovine Serum (FBS) (Gibco BRL, UK), 10X phosphate buffer saline (Gibco BRL, UK), trypsin 0.25% EDTA 0.003% (Gibco BRL, UK) and penicillin/streptomycin solution 10 mg/mL (Sigma-Aldrich), DMSO (HiMedia), (S,R)-Noscapine (Aldrich), 4% Paraformaldehyde solution (HiMedia), Hoechst 3334 (trihydrochloride, trihydrate) (Invitrogen), Crystal violet (HiMedia), 3-(4,5-Dimethyl-2-thiazolyl)-2,5-diphenyl-2*H*-tetrazolium bromide (Hi-AR™) were used in this study for cell culture and treatment. All other chemicals were also of analytical grade and exhibited the highest purity level. Two human breast cancer cell lines (MDA-MB-231 and MCF-7), one lung cancer cell line (A549) and a human embryonic kidney cell line (HEK-293) were obtained from NCCS, Pune, India. These cell lines were maintained and cultured in a humidified incubator at 37 °C with 5% CO_2. The cytotoxicity measurements were performed using a microplate reader (iMark™). The cellular morphology was examined using an inverted microscope (Nikon ECLIPSE T_S2R). Laboratory experiments and all related manipulations have been carried out under strict aseptic condition using a class A2 Biosafety Cabinet.[6]

4.1.2. Cell viability assay

The cell proliferation activity was determined using the MTT assay (3-(4,5-dimethylthiazol-2-yl)-2,5-diphenyltetrazolium bromide). In brief, 5×10^3 cells/well were seeded into 96-well plates with different concentrations of the drug (5, 10, 20, 50, 75, and 100 µM), followed by a 72h of incubation period. After a specified time period, the cells were treated with an MTT solution (5mg/mL) in light-protected conditions for 3-4h at 37 °C. The absorbance thereafter was determined at 570 nm using a plate reader. The IC_{50} value of the drug was calculated using GraphPad Prism software (version 9.0). All the experiments were performed in three replicates.[6]

4.1.3. Hoechst 3334 and Acridine Orange staining assay

MCF7 cells were cultured on poly-L-lysine-coated coverslips in 6-well plates and treated with the IC_{50} concentrations of two potent compounds **4f, 4h** and **4i** for 24 hours. Subsequently, the cells were fixed using 4% *p*-formaldehyde and then stained with Hoechst 3334 and acridine orange. The morphological changes in control and treated cells were evaluated using a fluorescence microscope. Apoptotic cells were identified based on nuclear condensation, formation of apoptotic bodies, and membrane blebbing.[6]

4.2. *In vitro antimicrobial activity*

The antibacterial assay of all synthesized compounds was evaluated *in vitro* using the agar well diffusion method against the test organisms, namely, Gram-positive *S. aureus* and Gram-negative *E. coli*. Gentamicin served as the standard drug. The compounds were initially dissolved in DMSO, and their zone of inhibition (ZI) was assessed on Mueller-Hinton agar plates. A sterilized Petri plate was first poured with 25 mL of sterilized media, which was allowed to solidify. The microbial suspension was then spread over the agar surface, and a sterile cork borer was used to create 6 mm diameter wells. For ZI measurement, 80 µL of the test compounds (100 µg/mL)) was introduced into each well. The plates were incubated at 37 ± 2°C for 24 hours to measure the inhibition zones. To determine the minimum inhibitory concentration (MIC), the synthesized compounds were further diluted to various concentrations (10, 20, 30, 40, 50, 60, 70, 80, and 100 µg/mL), and bacterial inhibition was assessed using a 96-well plate assay.[7-9]

5. Computational studies

5.1. *In silico molecular docking investigations*

The docking calculation of compounds **4(a-q)** was carried out by using AutoDock Tools version v4.2. Crystal structure of the *E. coli* protein, bacterial DNA gyrase (PDBID: 3G7E), and *S. aureus* protein, bacterial DNA gyrase (PDBID: 3G7B), was retrieved from Protein Data Bank (https://www. rcsb.org/), and 3D structures of the synthesized ligands were prepared by ChemDraw Ultra 12.0. Initially, during the molecular docking procedure, polar H-bonds were added, bounded ligands with water molecules were eliminated and other default parameters were employed. The 2D illustration of the docked complex of ligand-receptor was visualized by PyMOL (www.pymol.org) and BIOVIA Discovery Studio R2 2017. Subsequently, the top docking score was selected for further assessment of antibacterial properties.[10-11]

5.1.1. Molecular docking studies of the synthesized compounds 4(a-e), 4g and 4(j-q) with PDB ID: 1ZXN

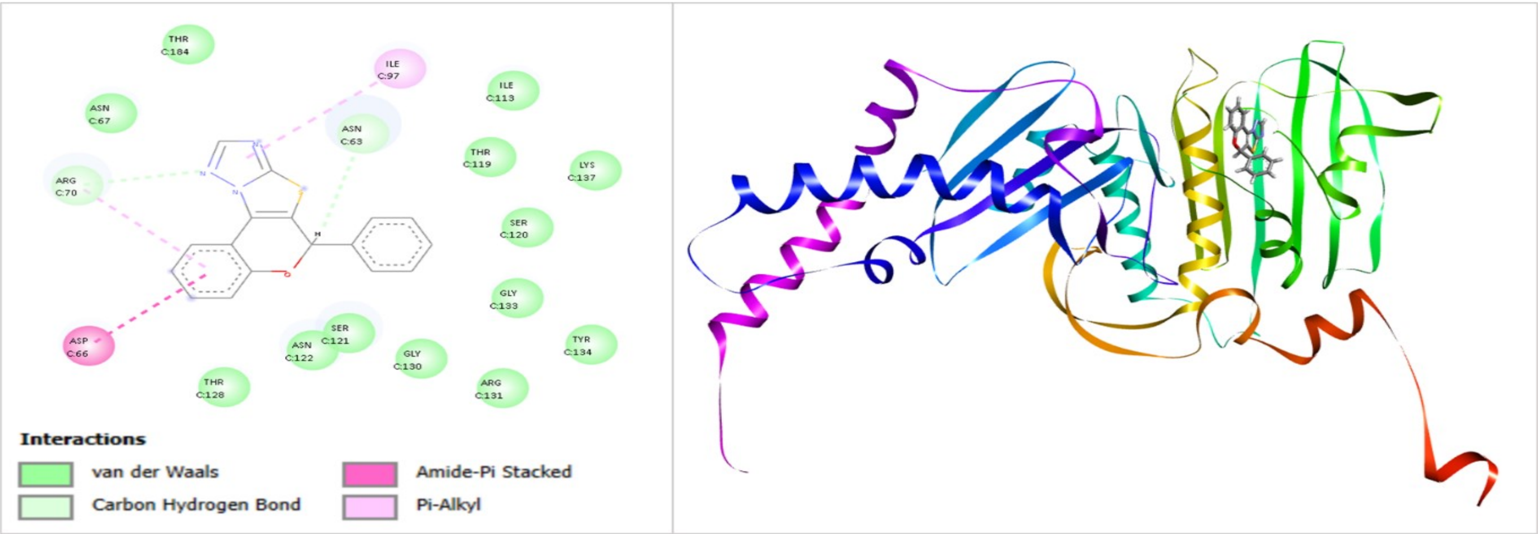

Fig S55. Binding interaction of compound **4a** with (PDBID:1ZXN)

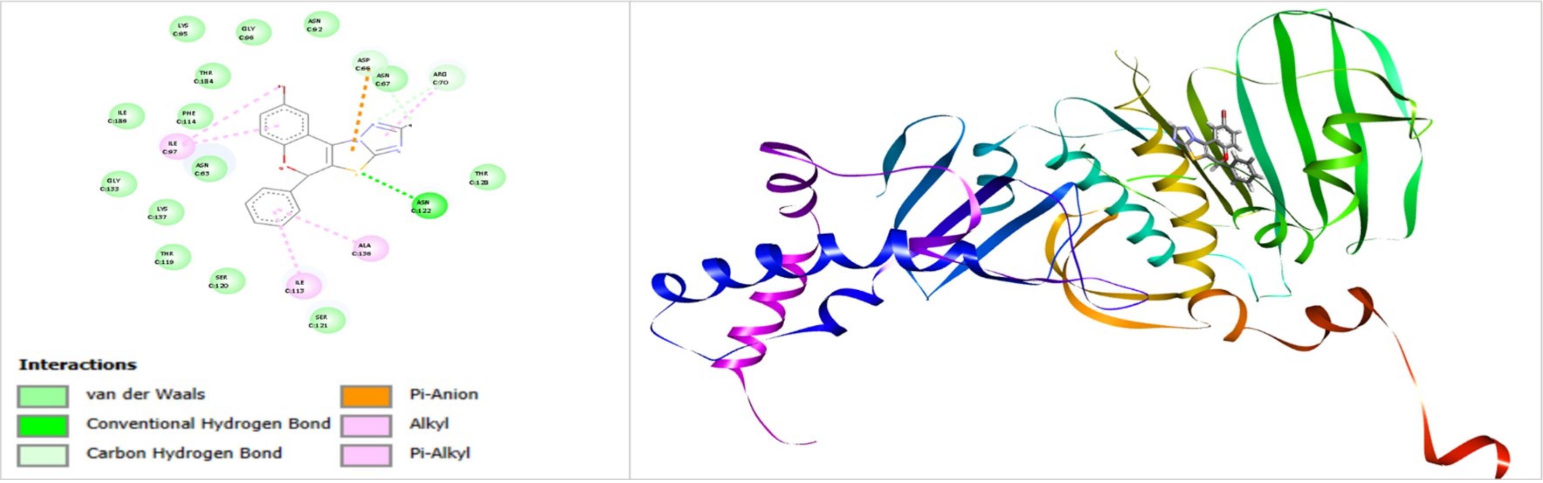

Fig S56. Binding interaction of compound **4b** with (PDBID:1ZXN)

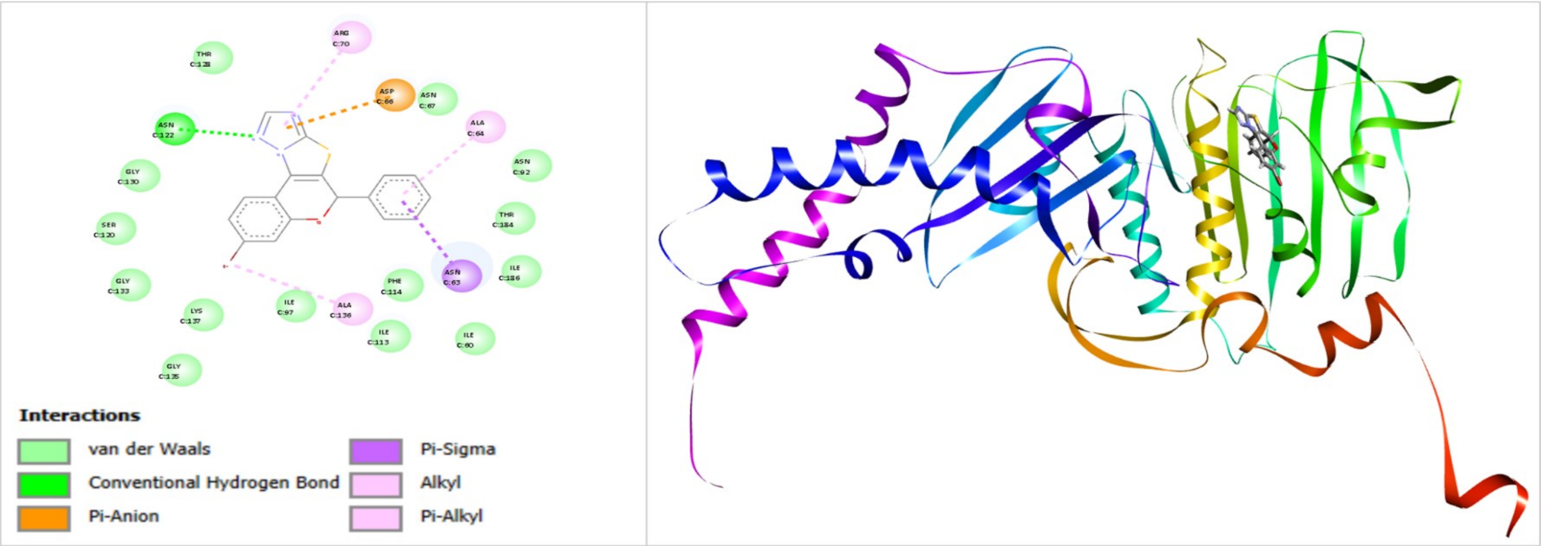

Fig S57. Binding interaction of compound **4c** with (PDBID:1ZXN)

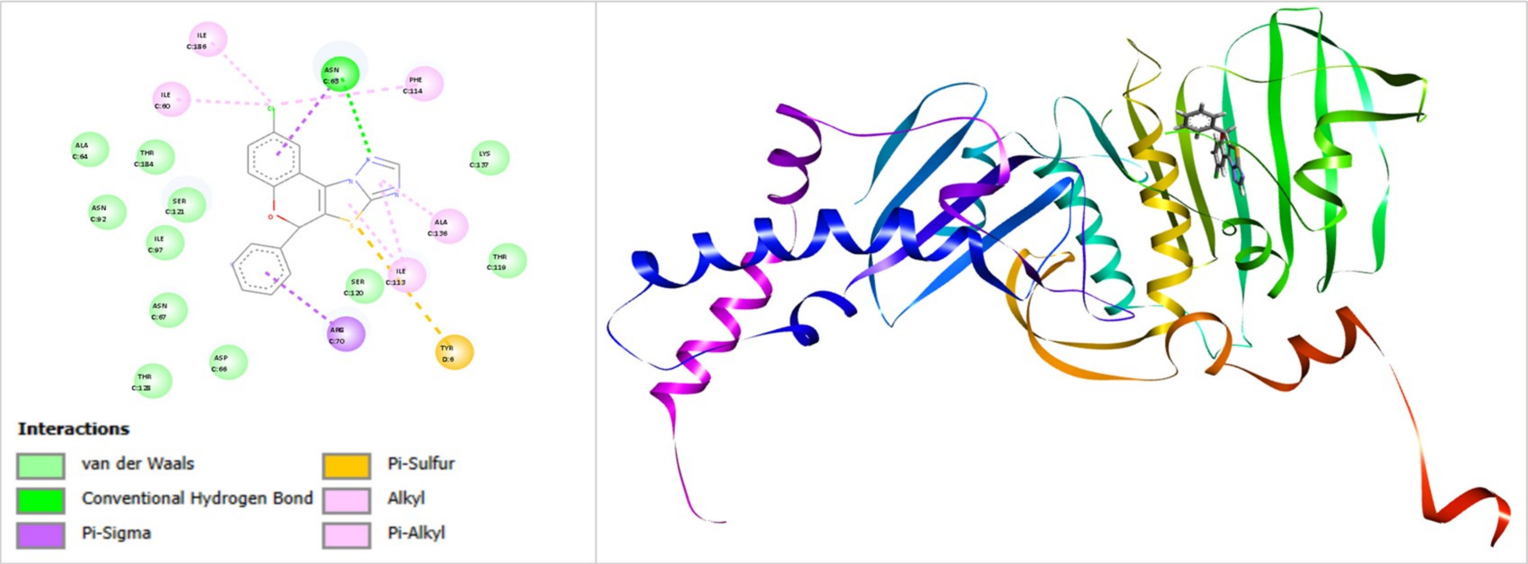

Fig S58. Binding interaction of compound **4d** with (PDBID:1ZXN)

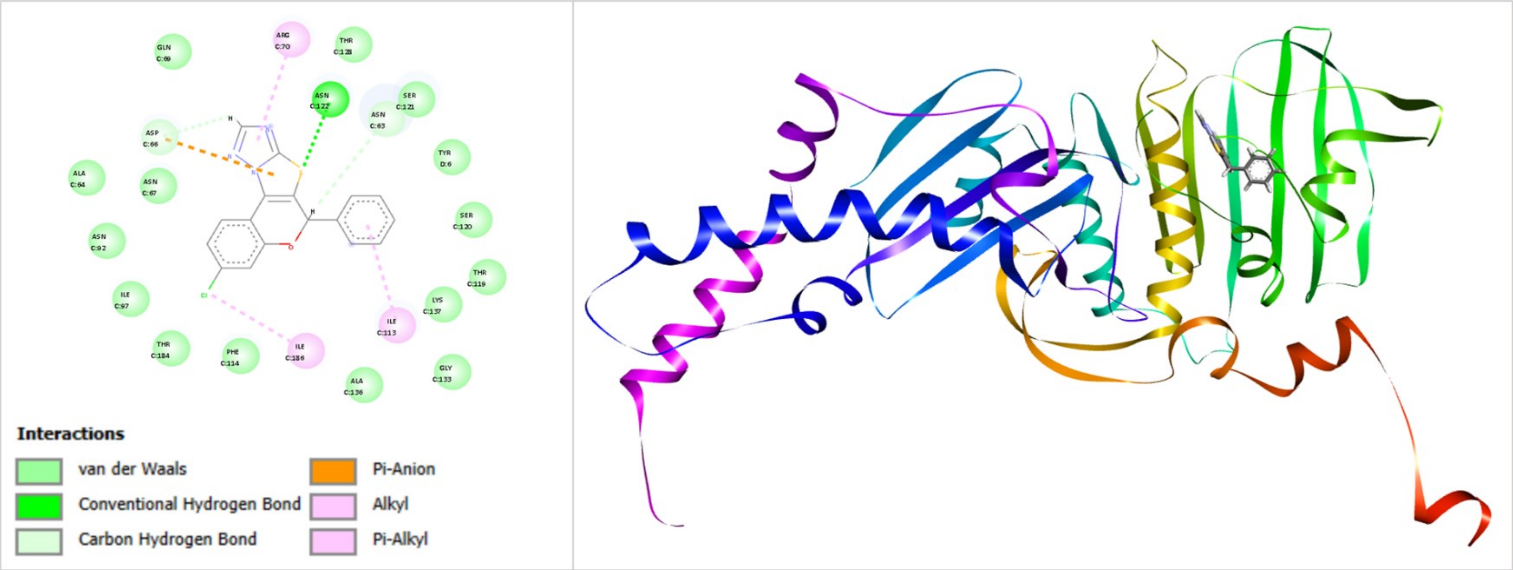

Fig S59. Binding interaction of compound **4e** with (PDBID:1ZXN)

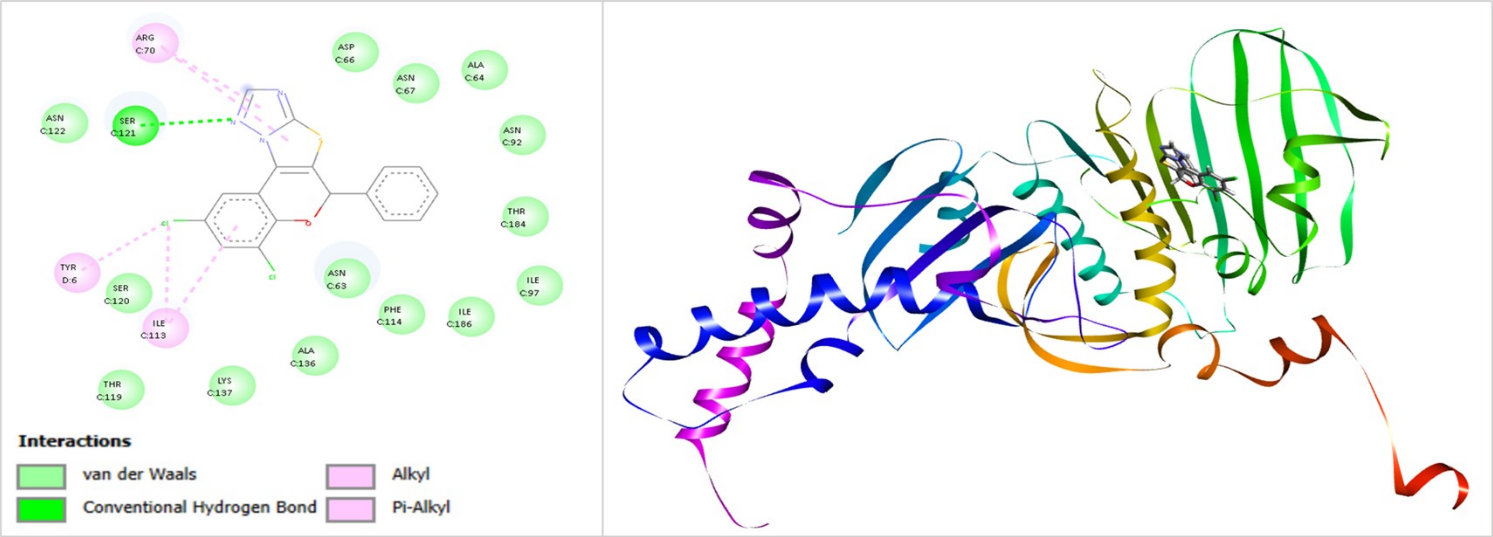

Fig S60. Binding interaction of compound **4g** with (PDBID:1ZXN)

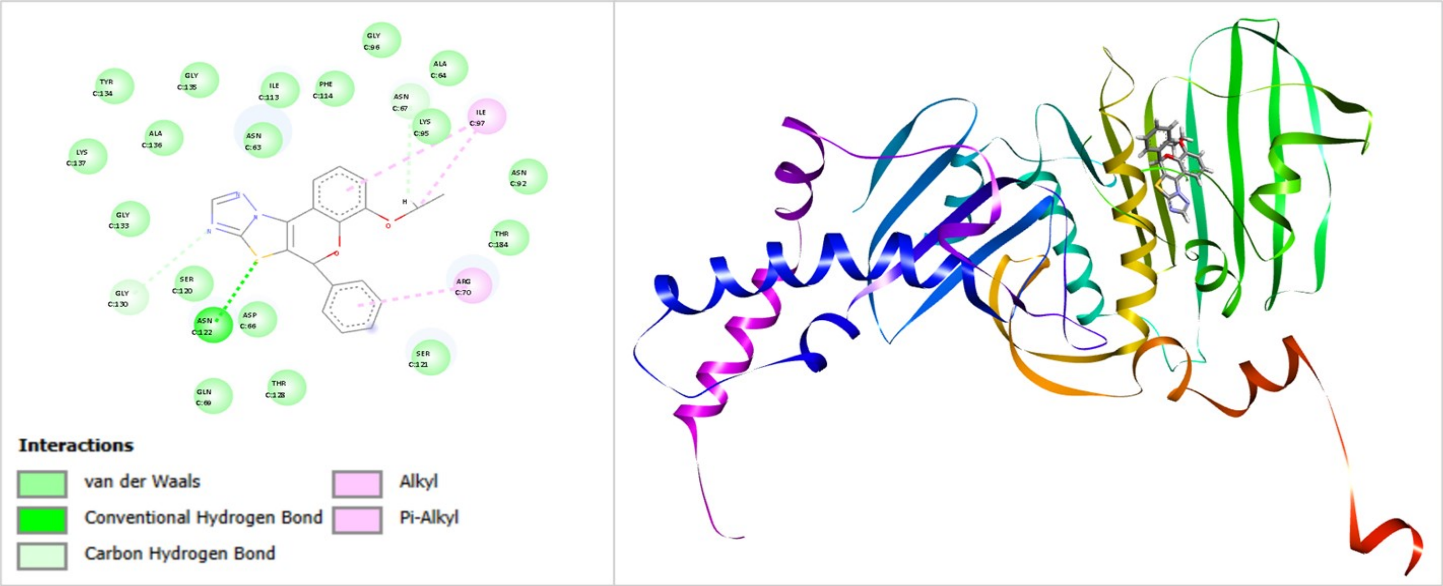

Fig S61. Binding interaction of compound **4j** with (PDBID:1ZXN)

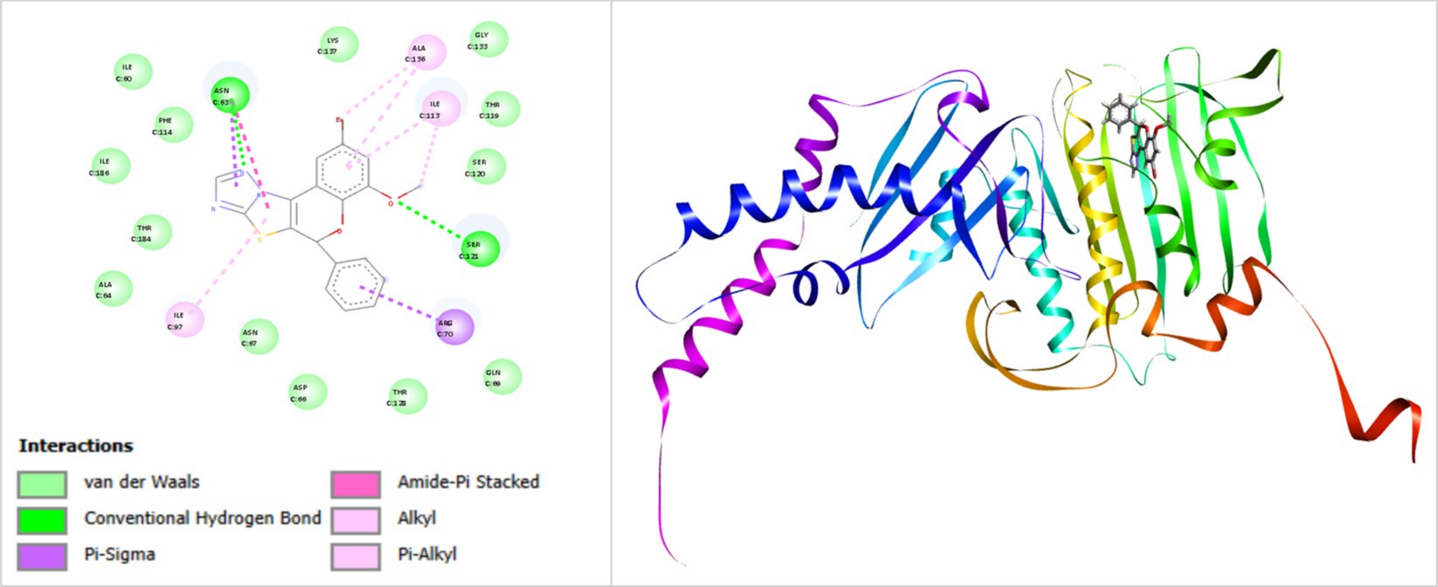

Fig S62. Binding interaction of compound **4k** with (PDBID:1ZXN)

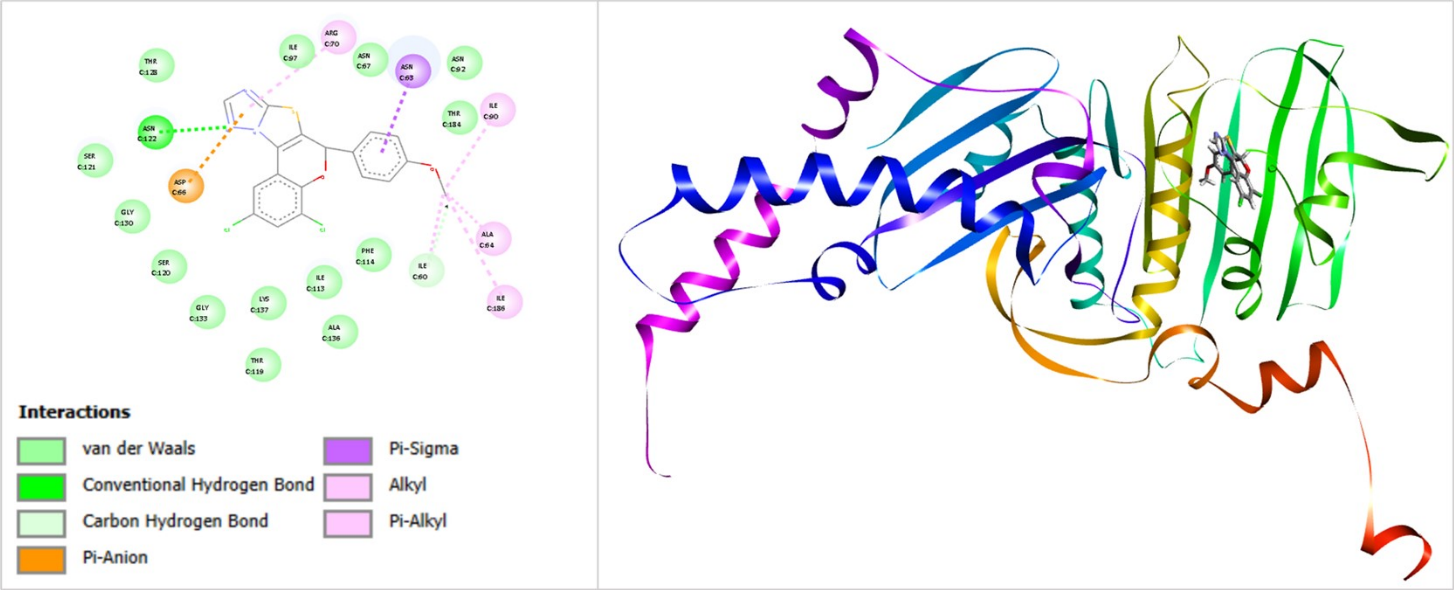

Fig S63. Binding interaction of compound **4l** with (PDBID:1ZXN)

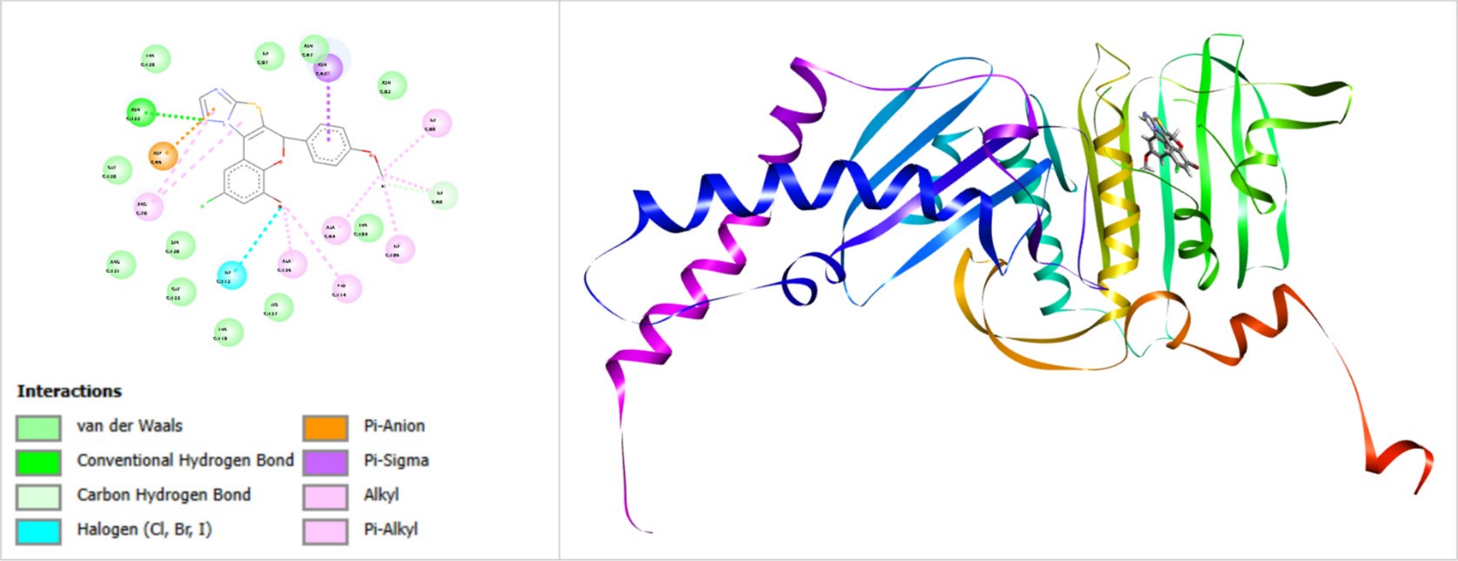

Fig S64. Binding interaction of compound **4m** with (PDBID:1ZXN)

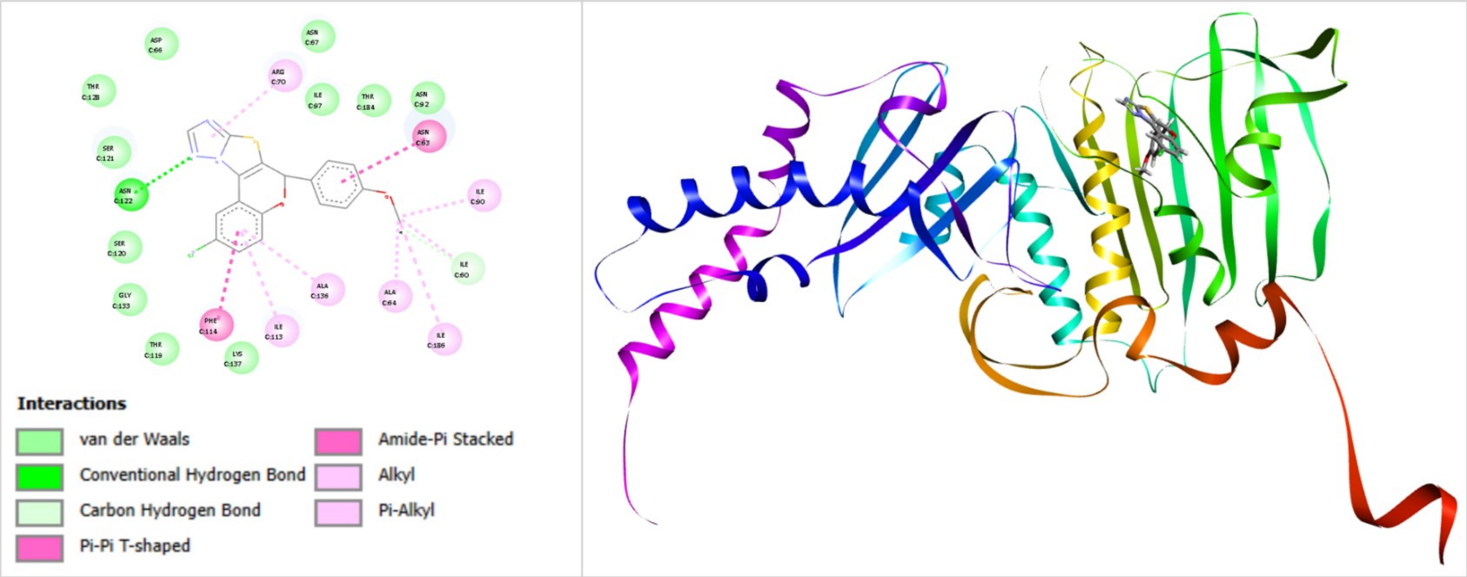

Fig S65. Binding interaction of compound **4n** with (PDBID:1ZXN)

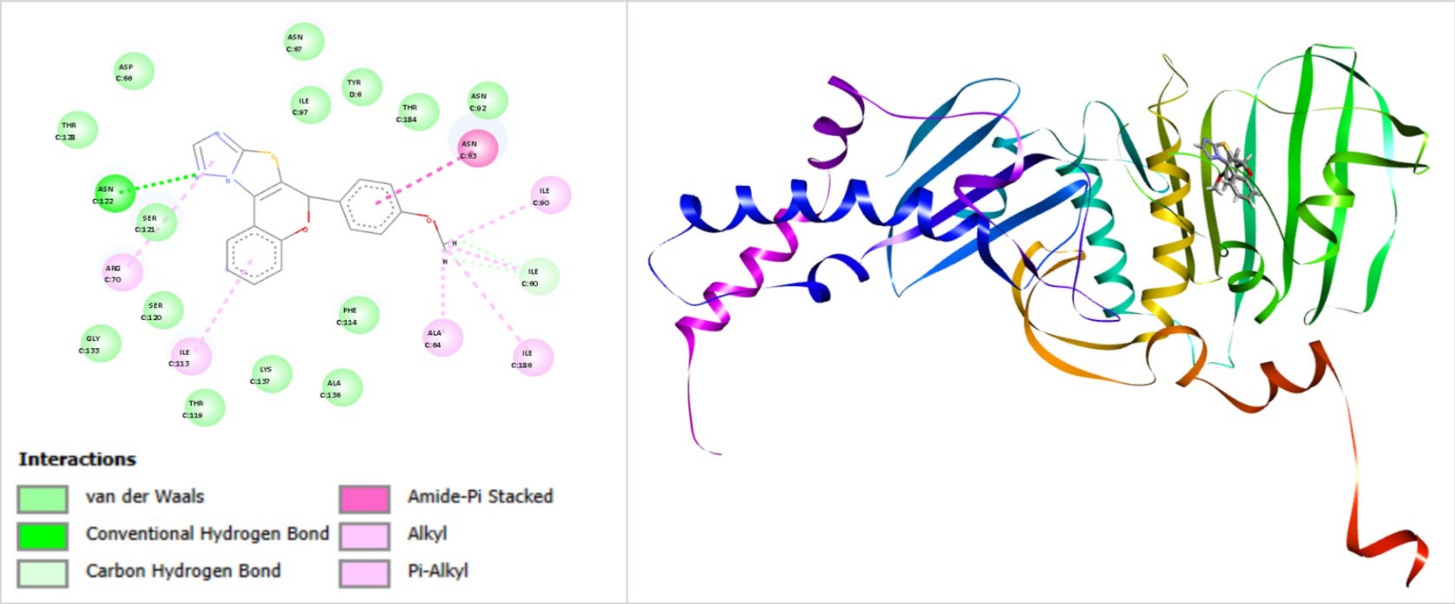

Fig S66. Binding interaction of compound **4o** with (PDBID:1ZXN)

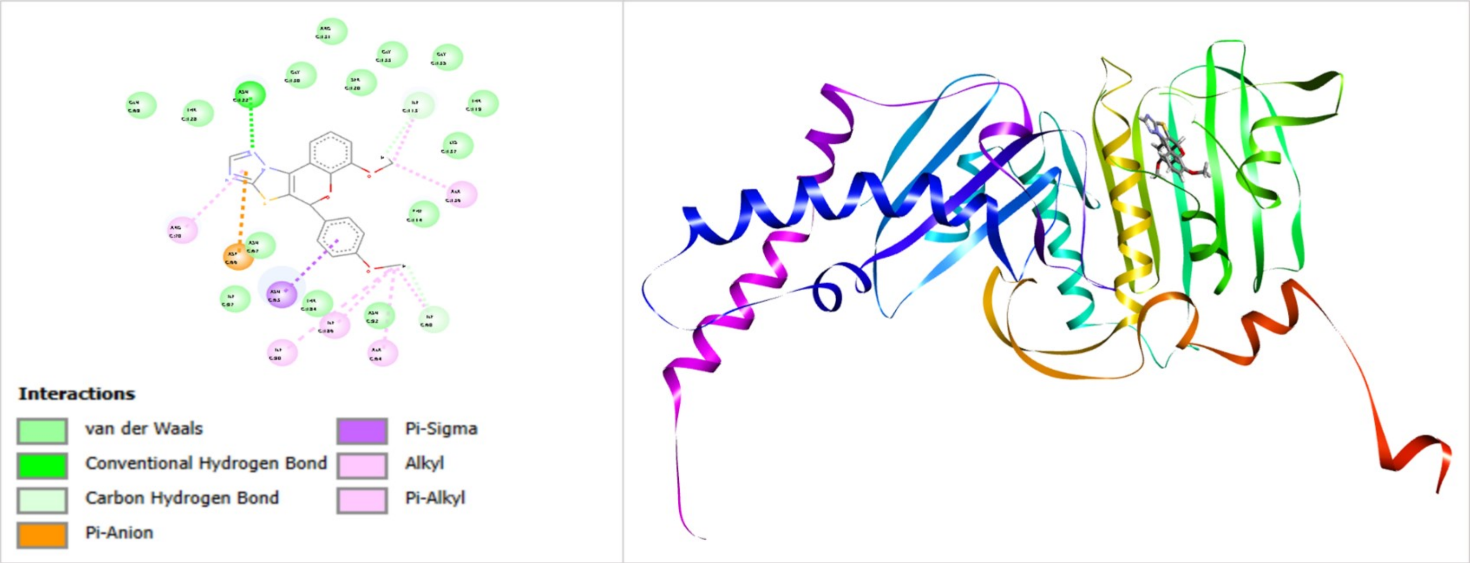

Fig S67. Binding interaction of compound **4p** with (PDBID:1ZXN)

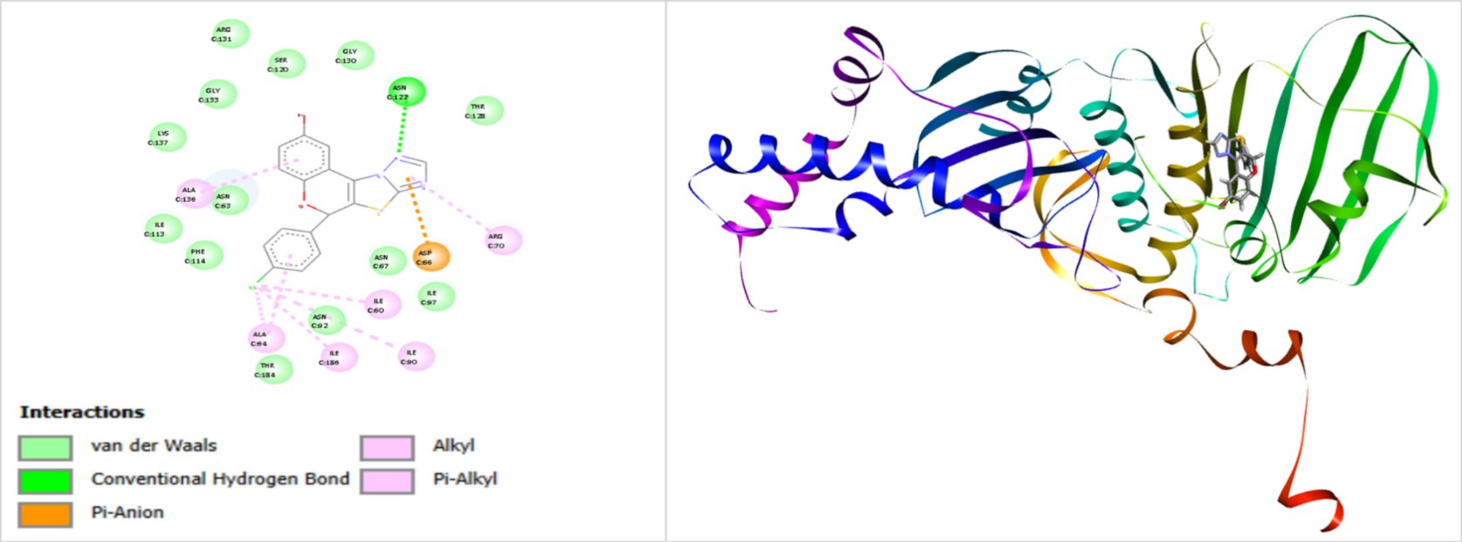

Fig S68. Binding interaction of compound **4q** with (PDBID:1ZXN)

5.1.2. Molecular docking studies of the synthesized compounds 4(a-e), 4g and 4(j-q) with PDB ID: 2W3L

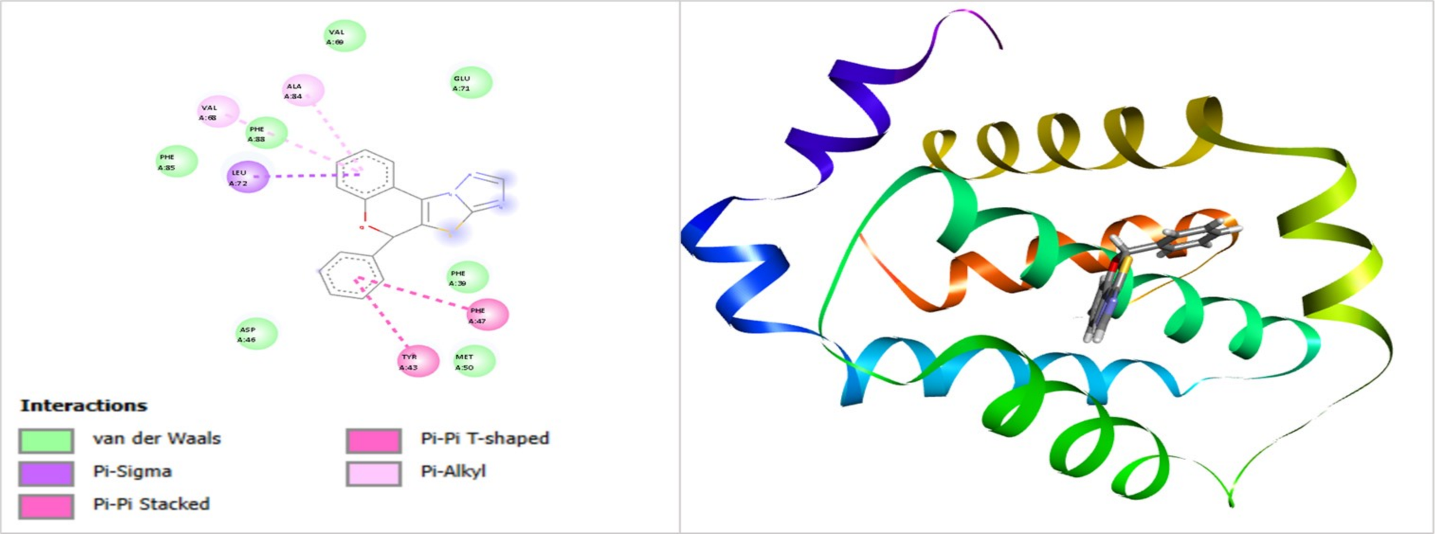

Fig S69. Binding interaction of compound **4a** with (PDBID:2W3L)

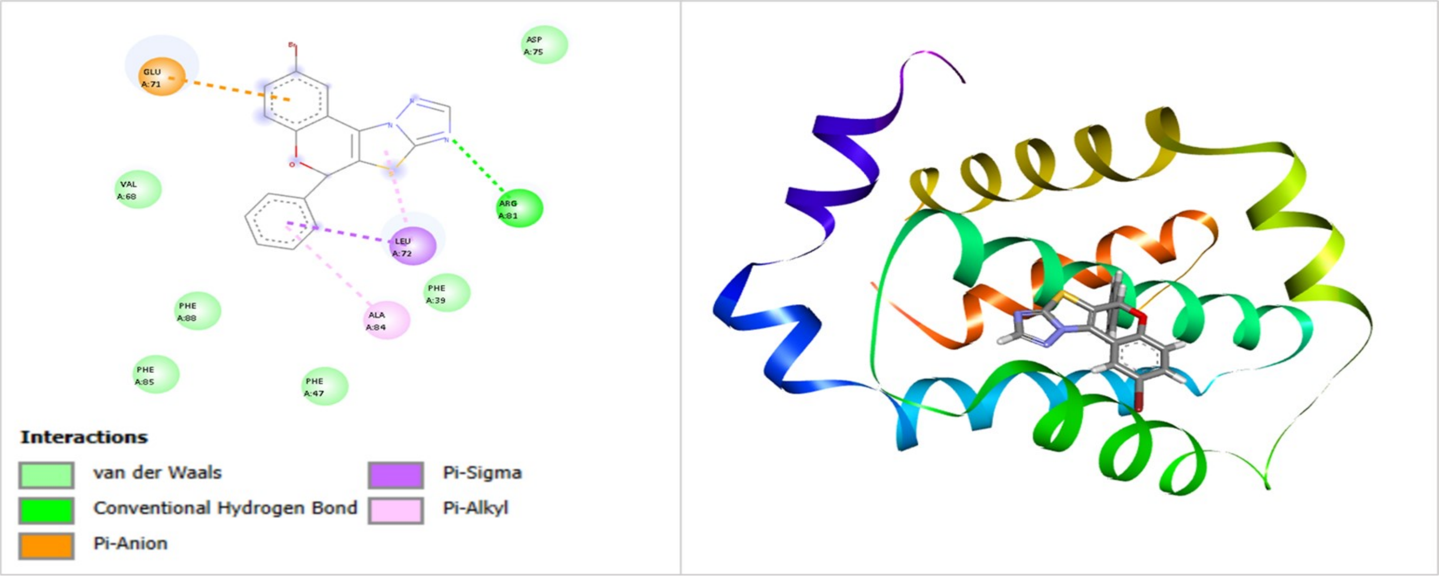

Fig S70. Binding interaction of compound **4b** with (PDBID:2W3L)

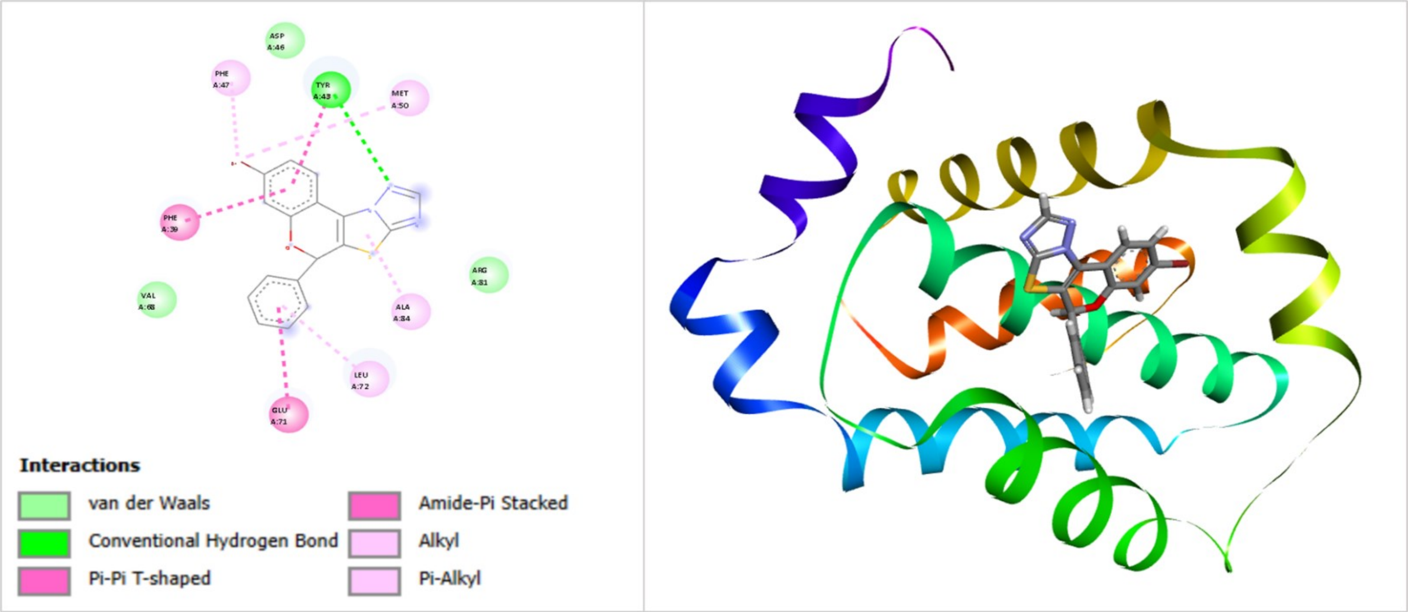

Fig S71. Binding interaction of compound **4c** with (PDBID:2W3L)

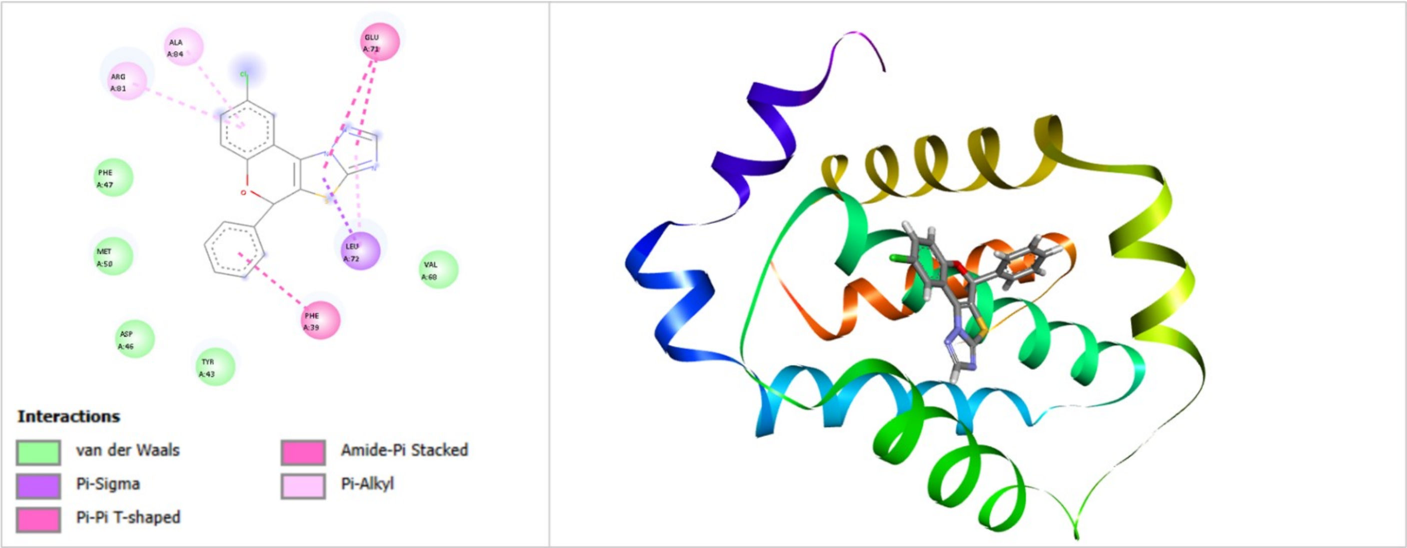

Fig S72. Binding interaction of compound **4d** with (PDBID:2W3L)

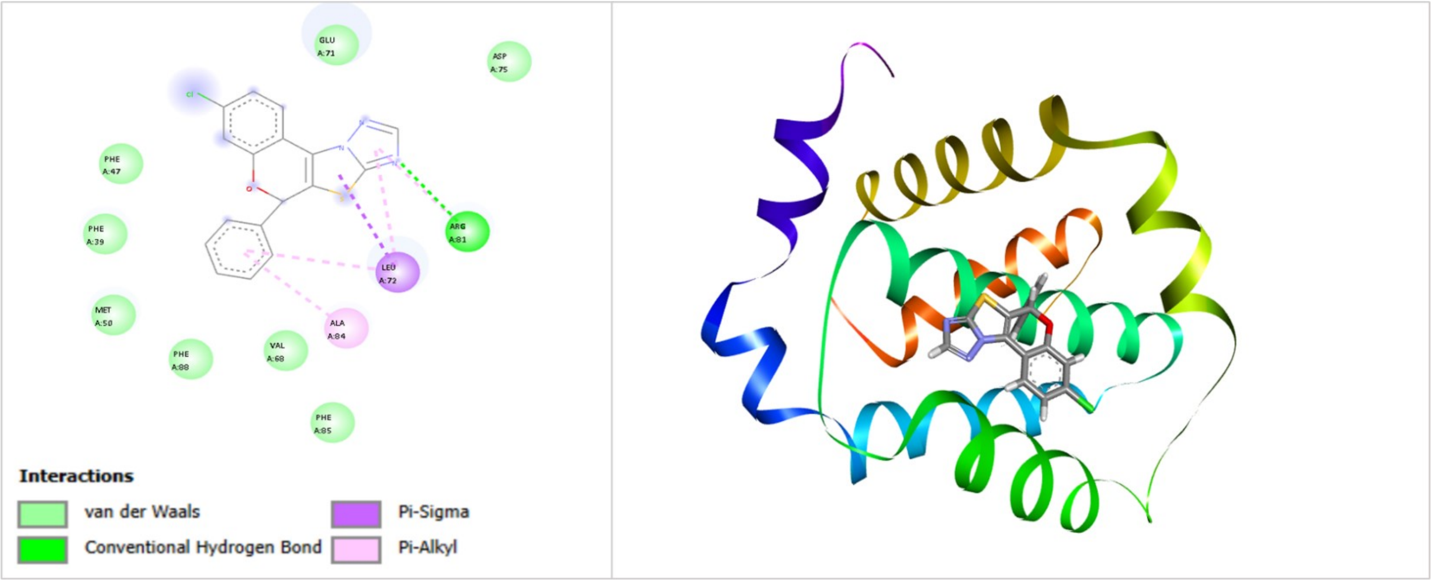

Fig S73. Binding interaction of compound **4e** with (PDBID:2W3L)

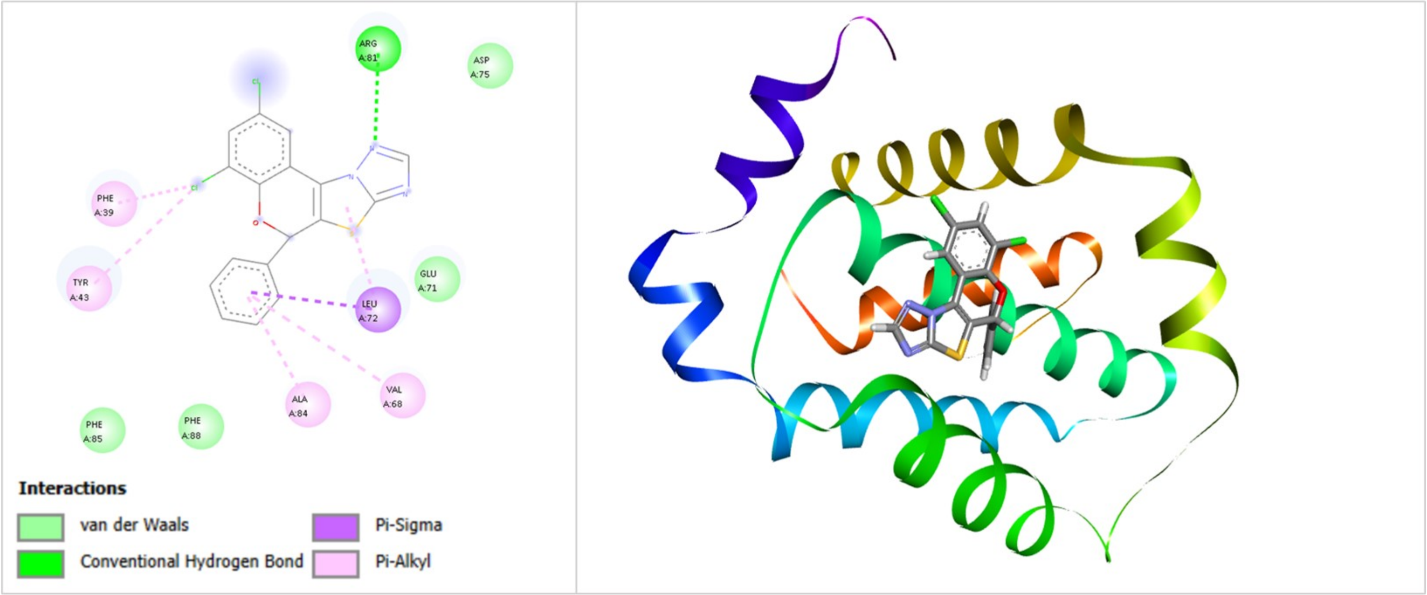

Fig S74. Binding interaction of compound **4g** with (PDBID:2W3L)

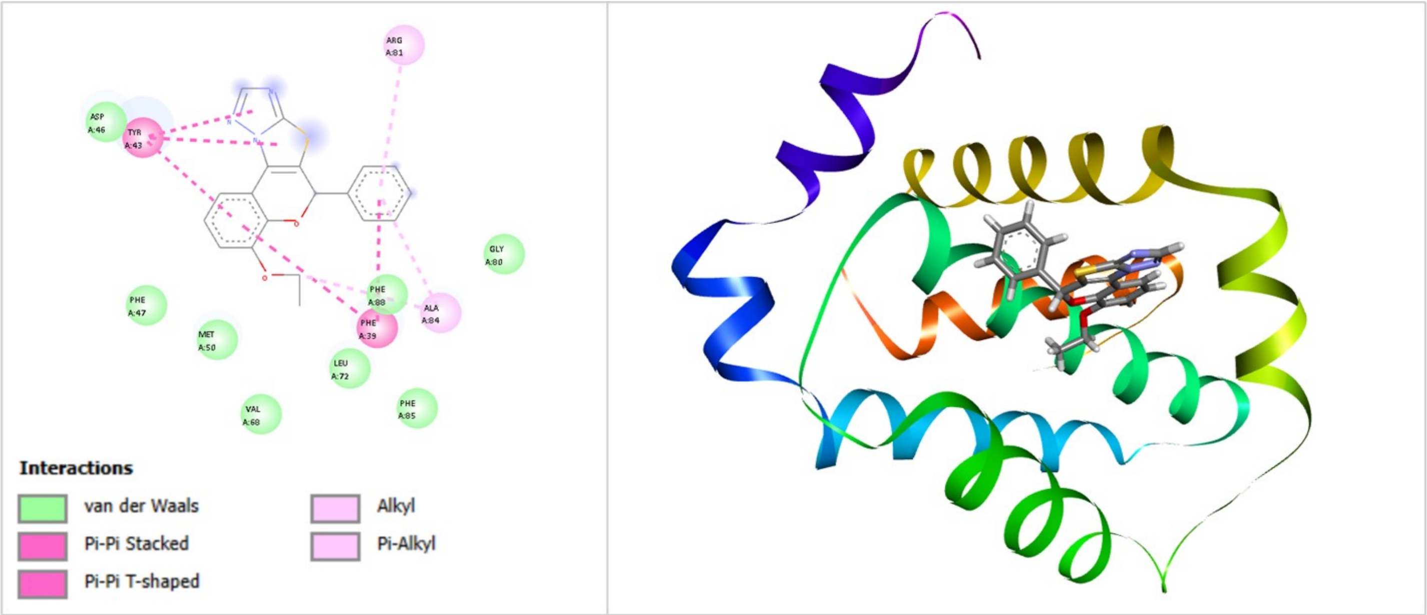

Fig S75. Binding interaction of compound **4j** with (PDBID:2W3L)

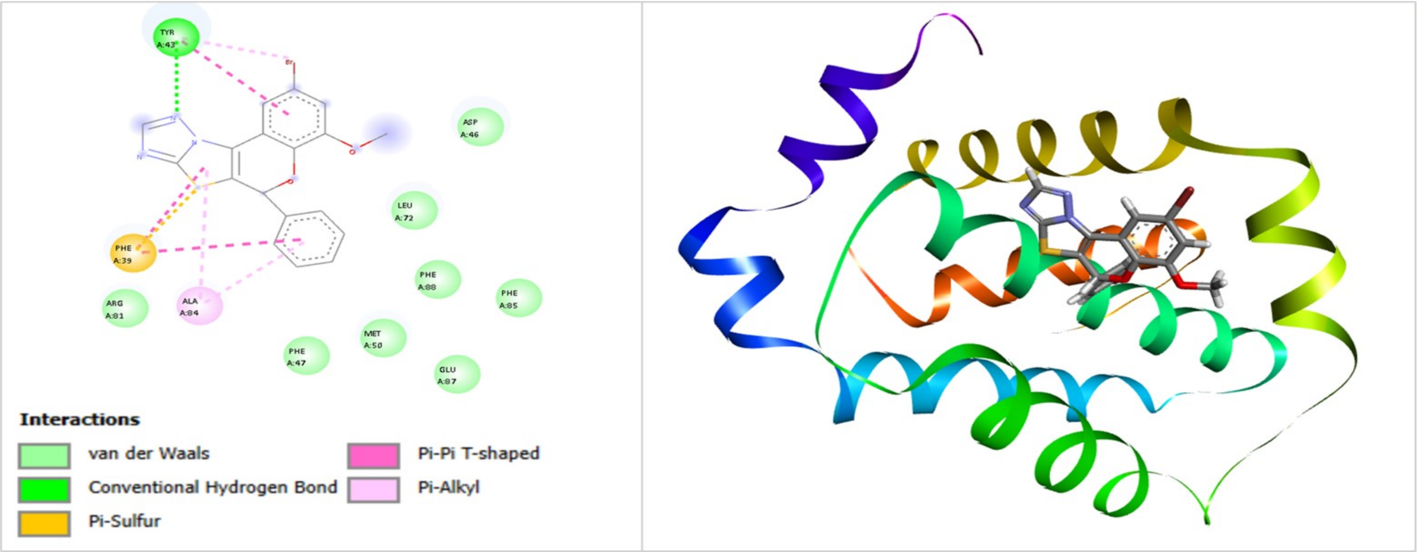

Fig S76. Binding interaction of compound **4k** with (PDBID:2W3L)

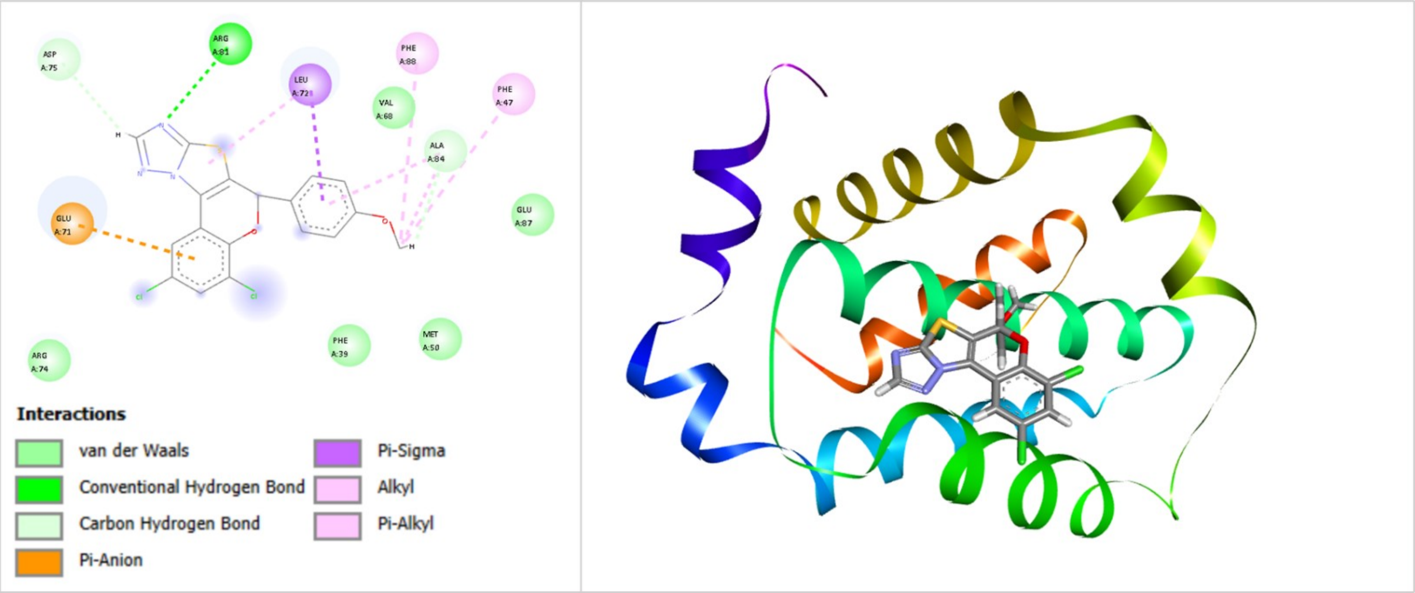

Fig S77. Binding interaction of compound **4l** with (PDBID:2W3L)

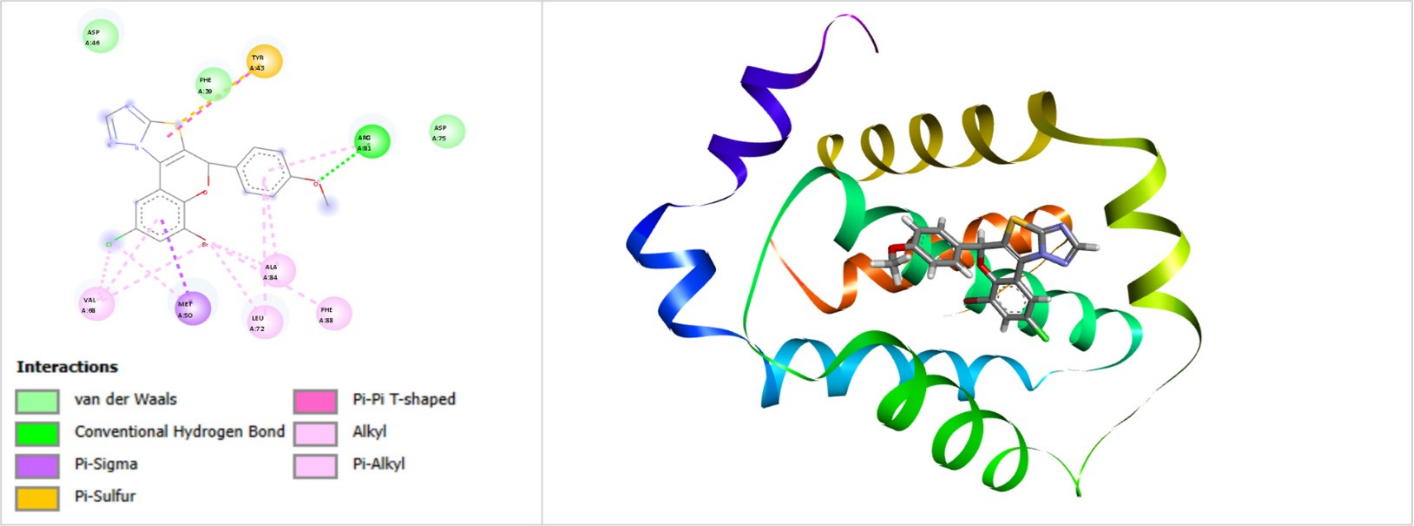

Fig S78. Binding interaction of compound **4m** with (PDBID:2W3L)

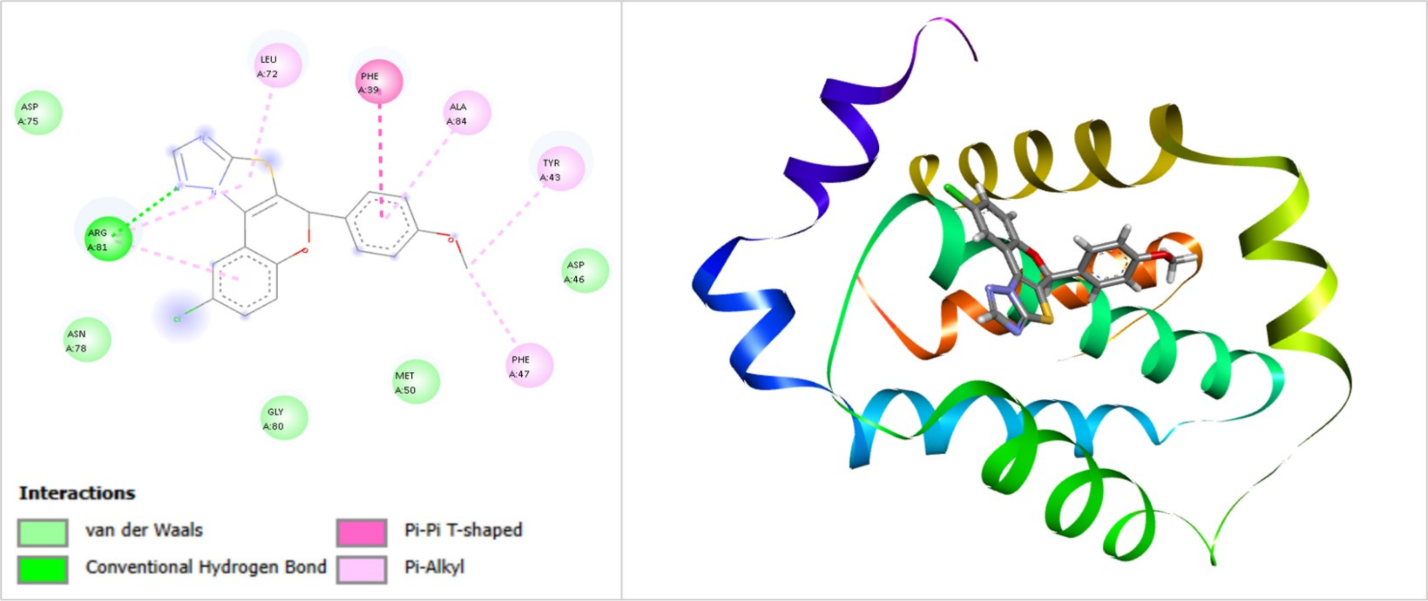

Fig S79. Binding interaction of compound **4n** with (PDBID:2W3L)

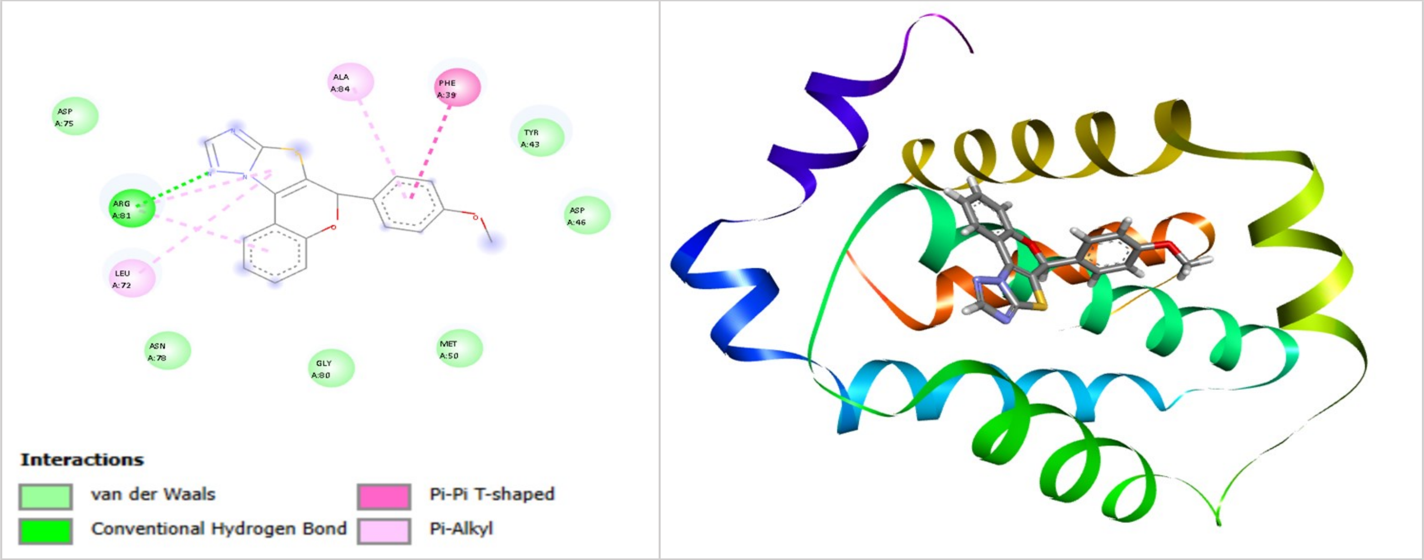

Fig S80. Binding interaction of compound **4o** with (PDBID:2W3L)

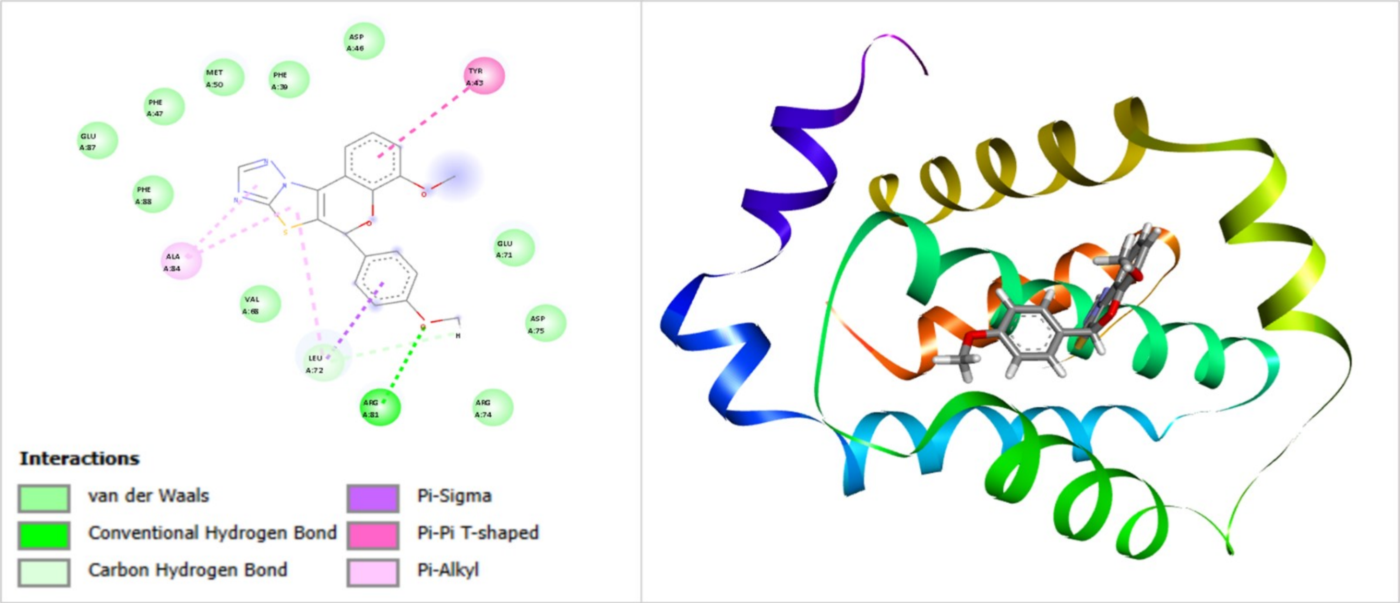

Fig S81. Binding interaction of compound **4p** with (PDBID:2W3L)

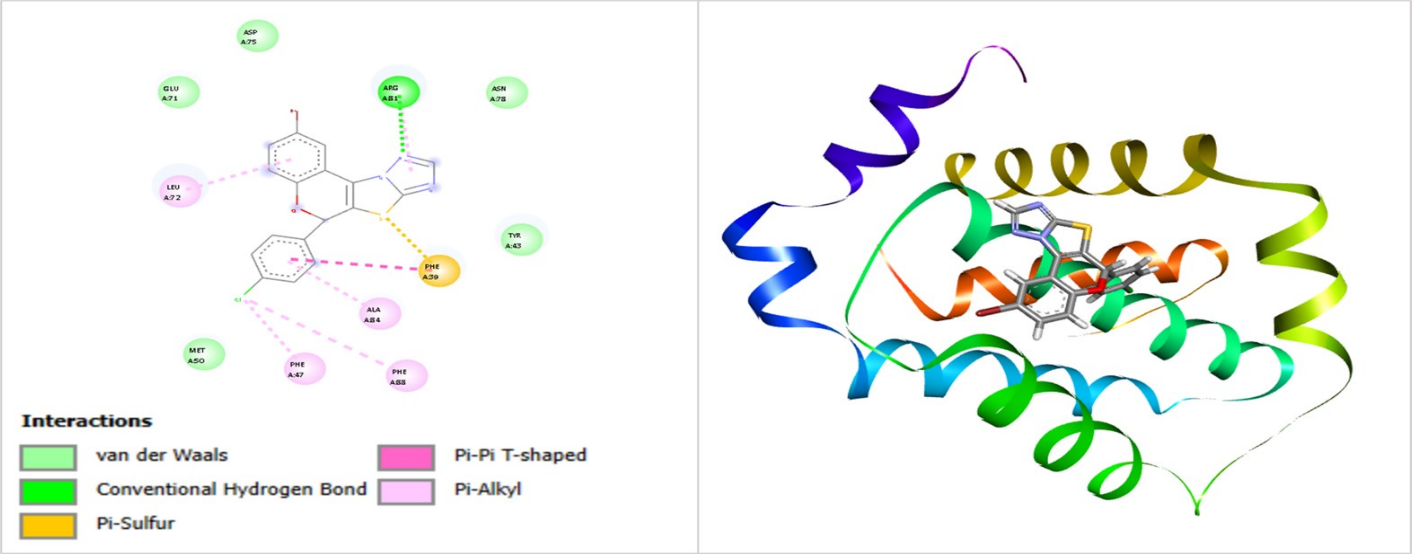

Fig S82. Binding interaction of compound **4q** with (PDBID:2W3L)

5.1.3. Molecular docking studies of the synthesized compounds 4(b-d), 4(f-h) and 4(j-q) with *E. coli* DNA gyrase (PDB ID: 3G7E)

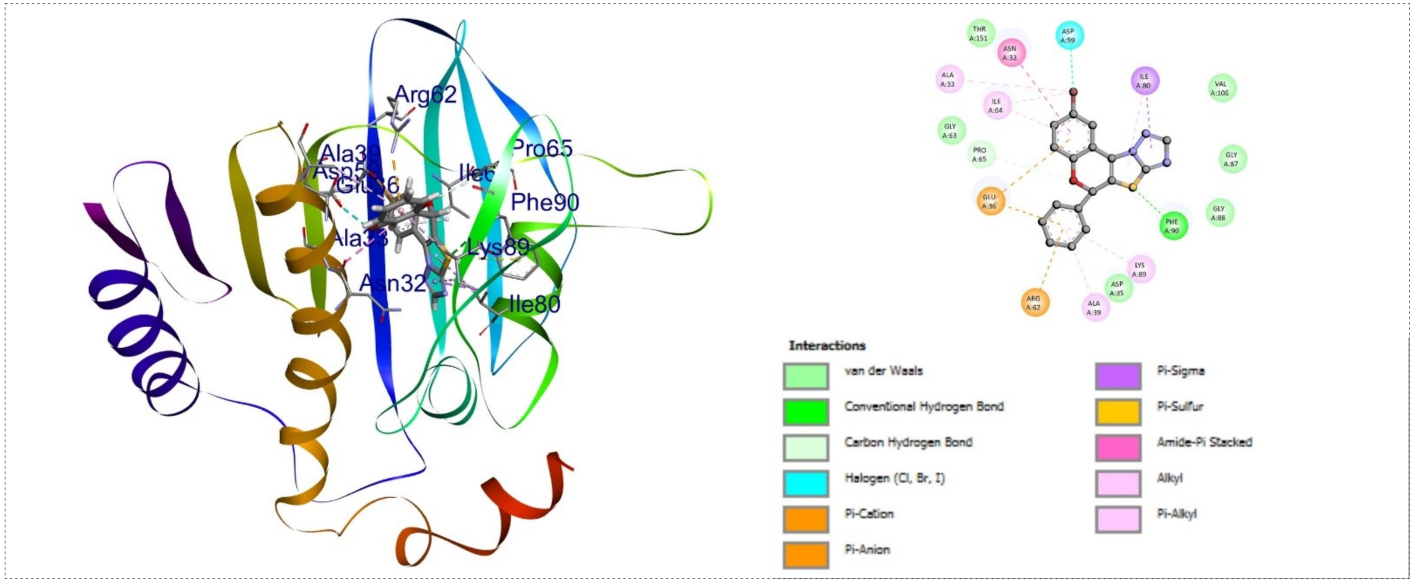

Fig S83. Binding interaction of compound **4b** with *E. coli* DNA gyrase (PDBID:3G7E)

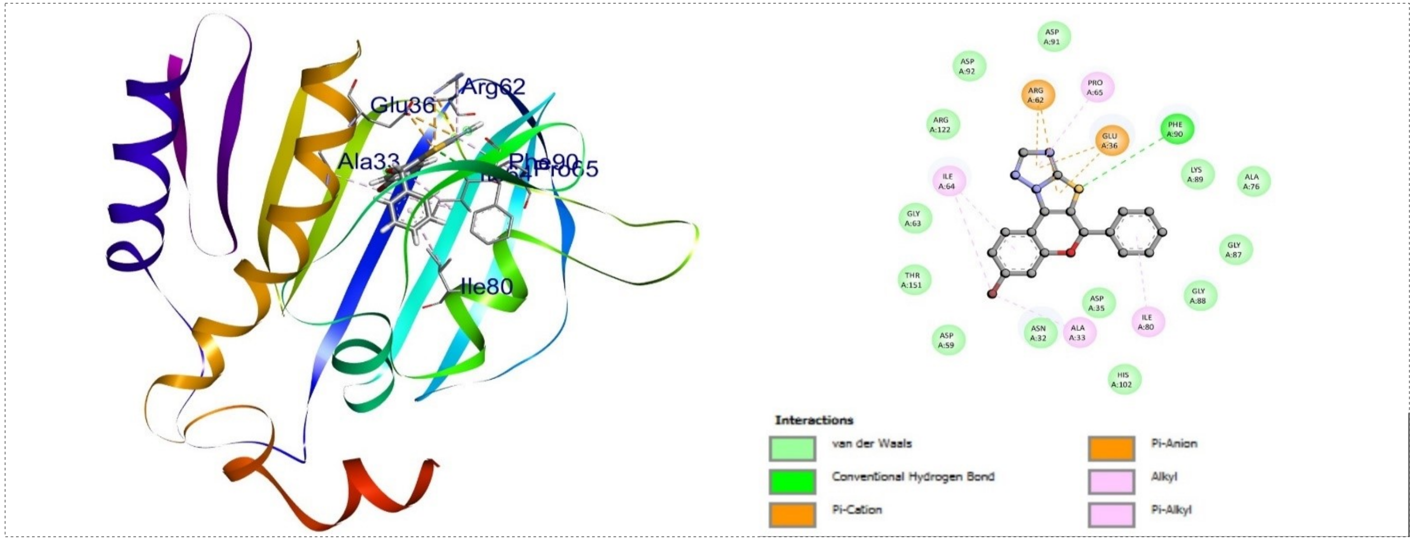

Fig S84. Binding interaction of compound **4c** with *E. coli* DNA gyrase (PDBID:3G7E)

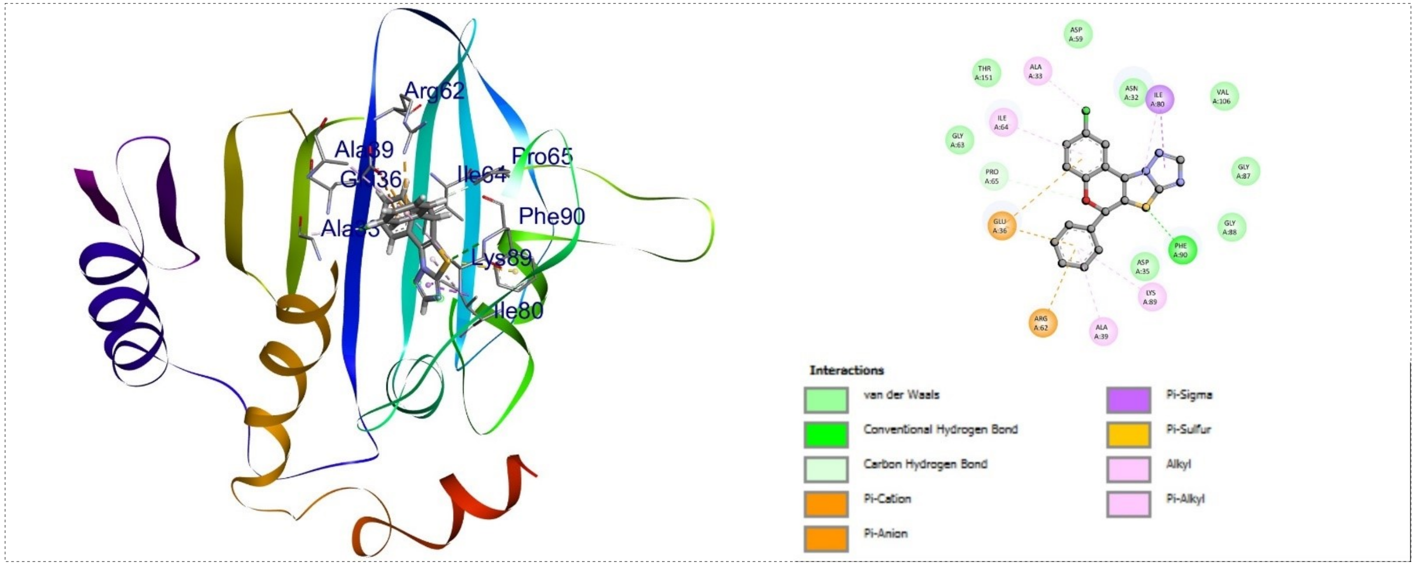

Fig S85. Binding interaction of compound **4d** with *E. coli* DNA gyrase (PDBID:3G7E)

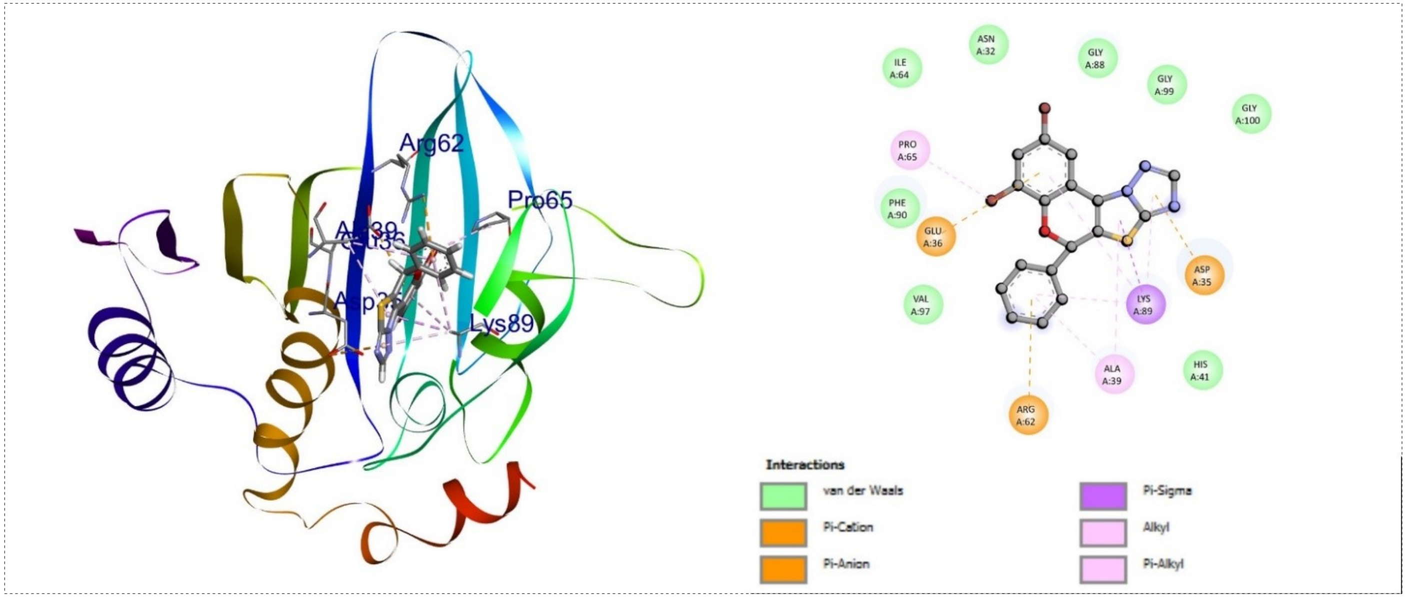

Fig S86. Binding interaction of compound **4f** with *E. coli* DNA gyrase (PDBID:3G7E)

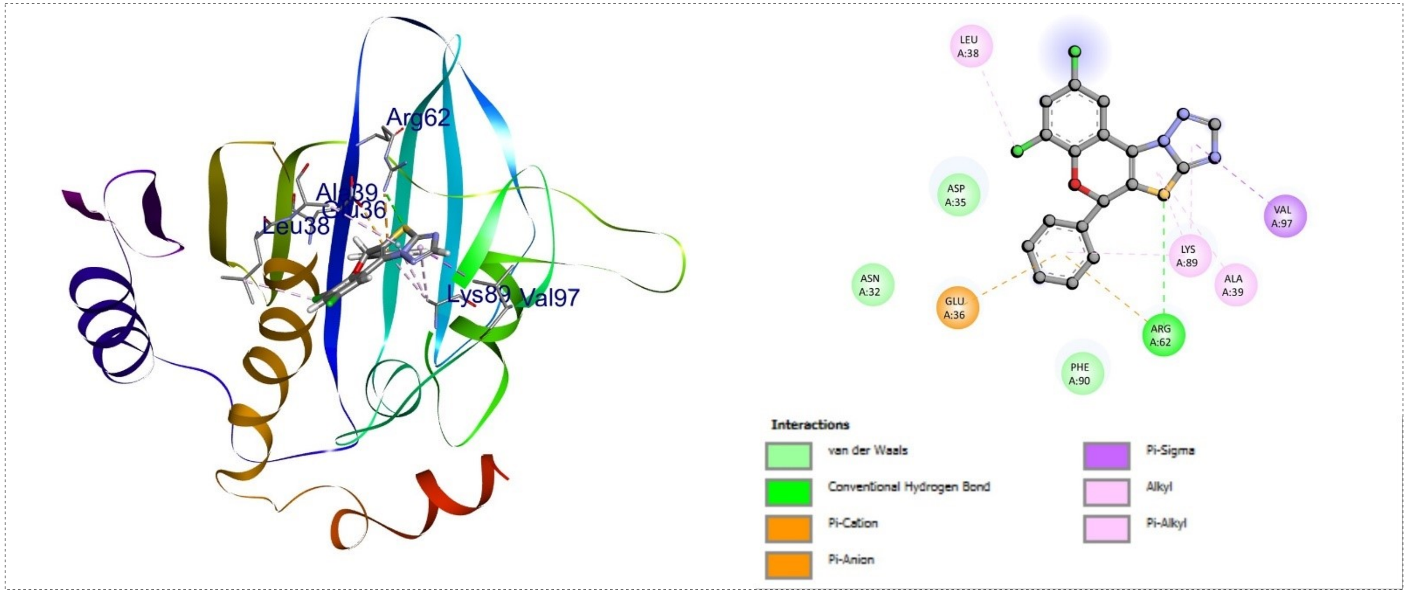

Fig S87. Binding interaction of compound **4g** with *E. coli* DNA gyrase (PDBID:3G7E)

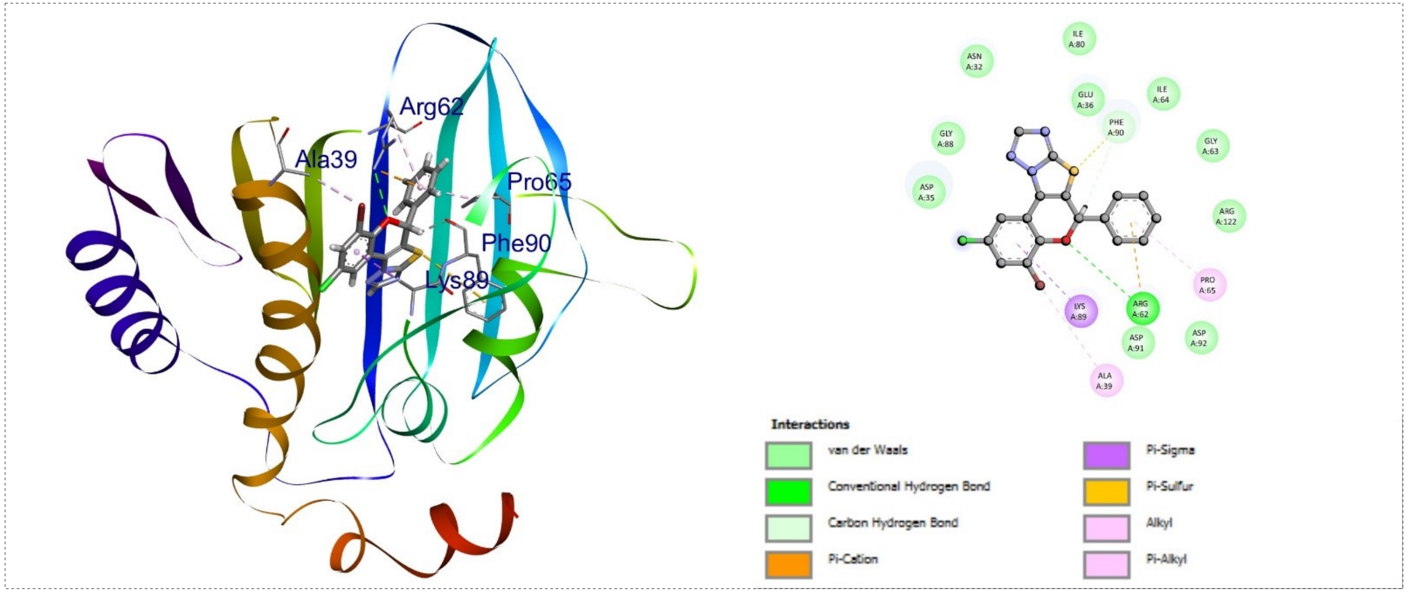

Fig S88. Binding interaction of compound **4h** with *E. coli* DNA gyrase (PDBID:3G7E)

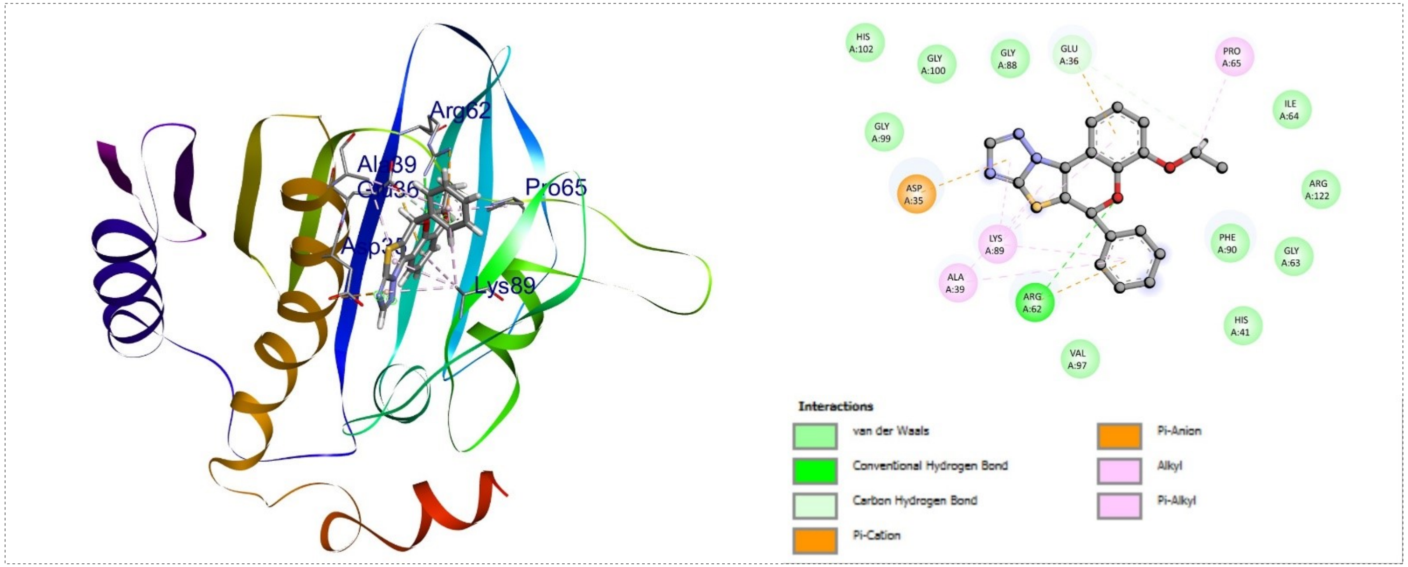

Fig S89. Binding interaction of compound **4j** with *E. coli* DNA gyrase (PDBID:3G7E)

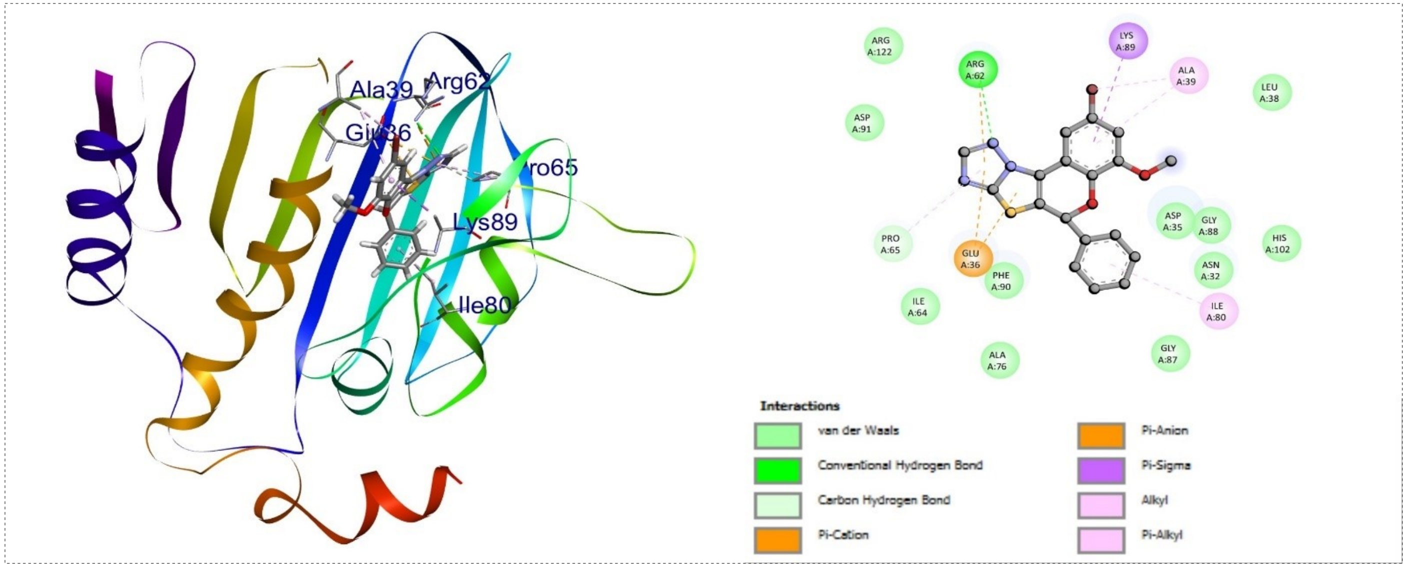

Fig S90. Binding interaction of compound **4k** with *E. coli* DNA gyrase (PDBID:3G7E)

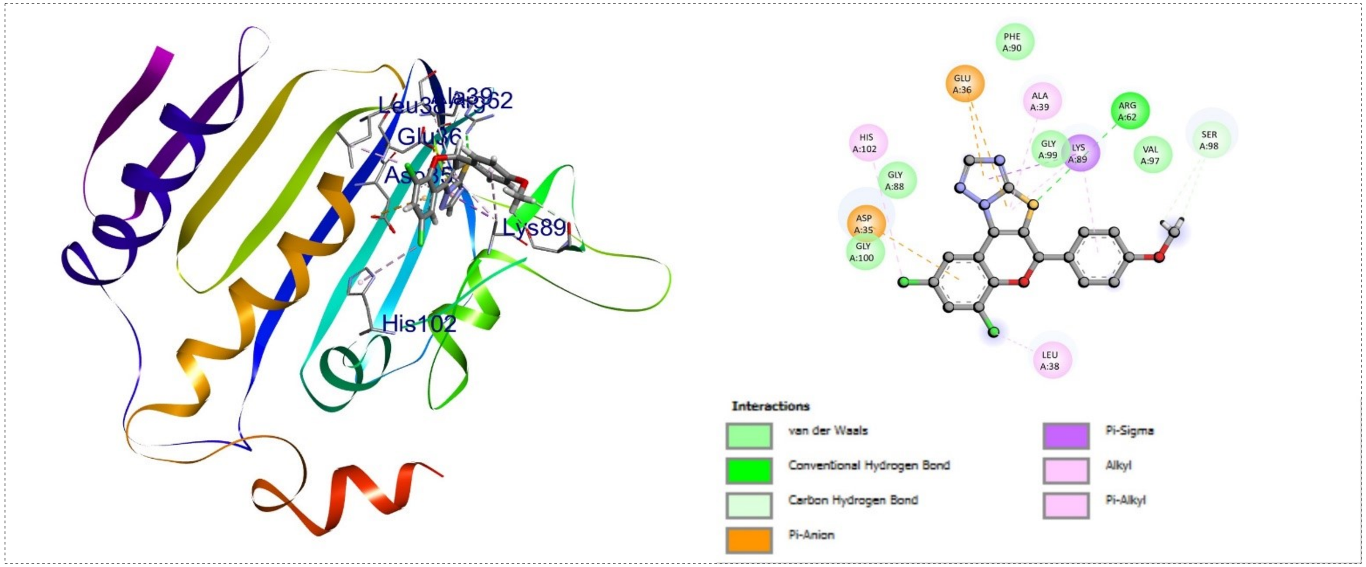

Fig S91. Binding interaction of compound **4l** with *E. coli* DNA gyrase (PDBID:3G7E)

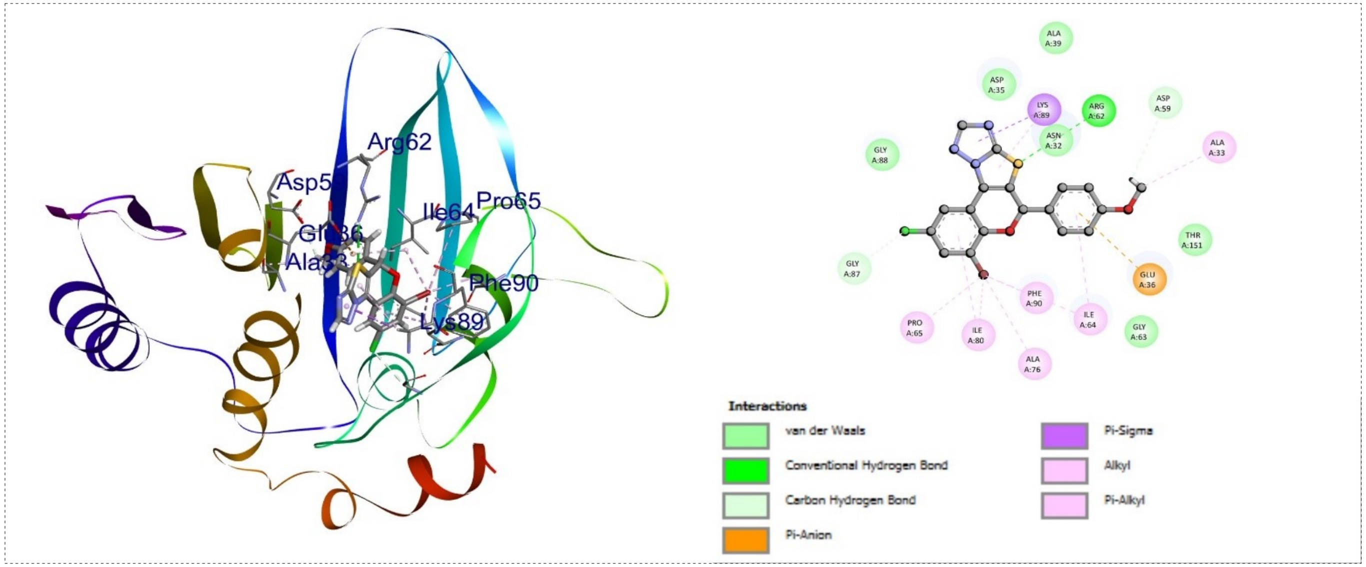

Fig S92. Binding interaction of compound **4m** with *E. coli* DNA gyrase (PDBID:3G7E)

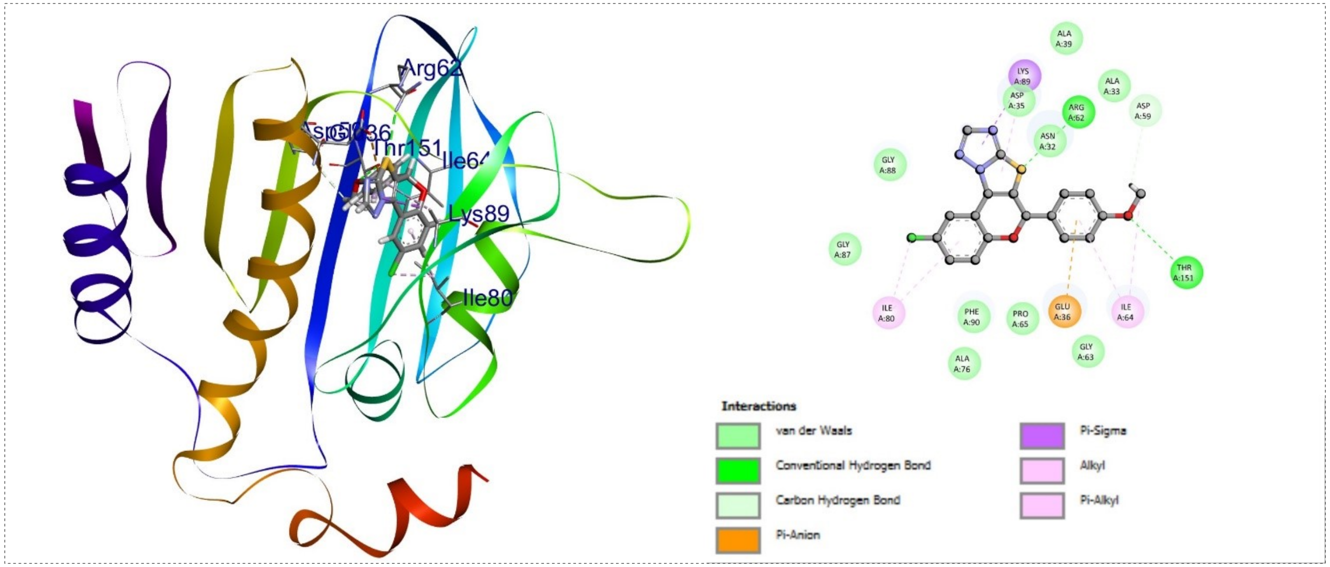

Fig S93. Binding interaction of compound **4n** with *E. coli* DNA gyrase (PDBID:3G7E)

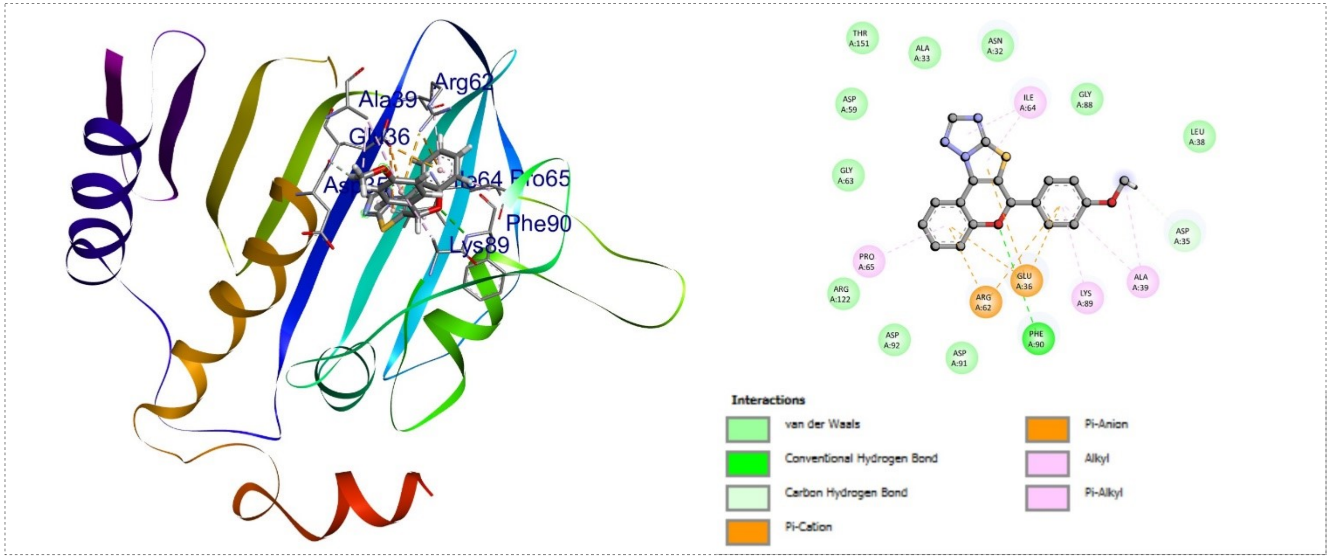

Fig S94. Binding interaction of compound **4o** with *E. coli* DNA gyrase (PDBID:3G7E)

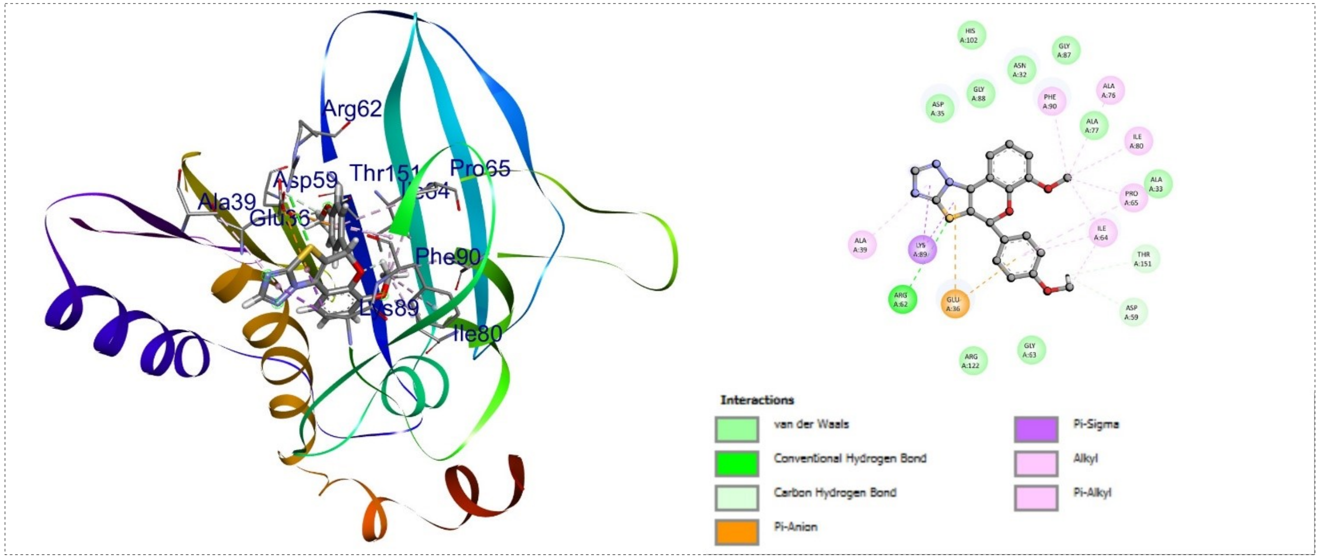

Fig S95. Binding interaction of compound **4p** with *E. coli* DNA gyrase (PDBID:3G7E)

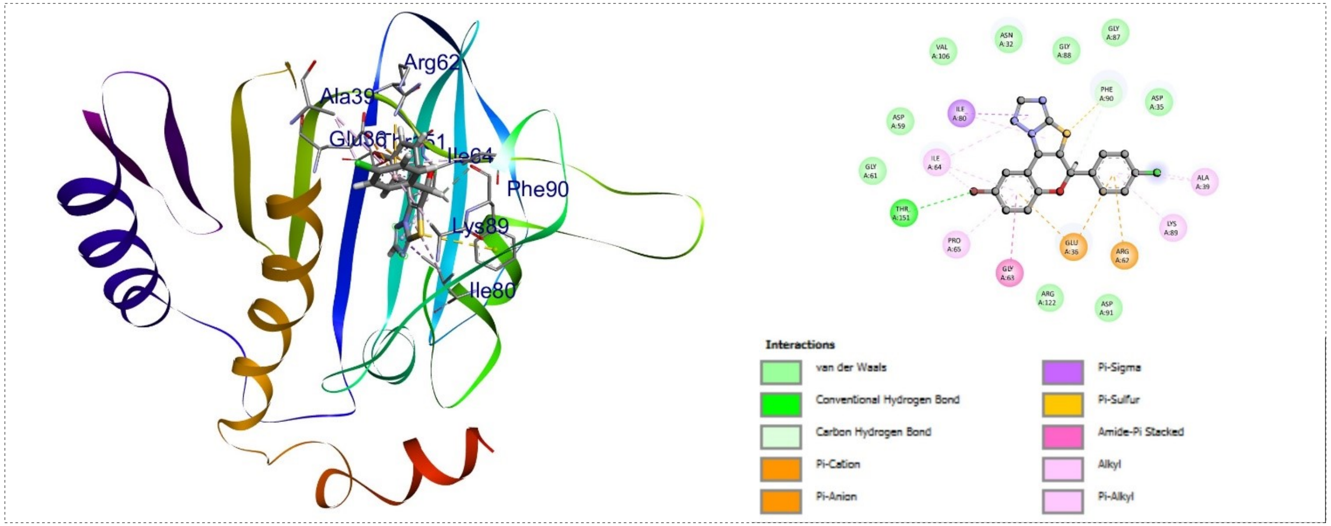

Fig S96. Binding interaction of compound **4q** with *E. coli* DNA gyrase (PDBID:3G7E)

5.1.4. Molecular docking studies of the synthesized compounds 4(b-d), 4(f-h) and 4(j-q) with *S. aureus* DNA gyrase (PDB ID: 3G7B)

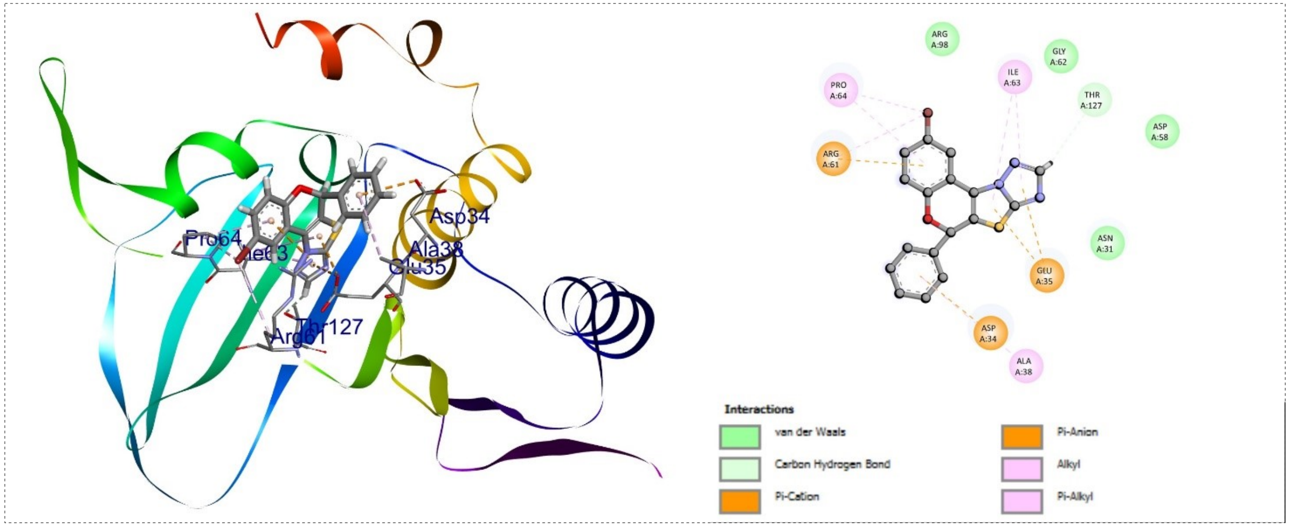

Fig S97. Binding interaction of compound **4b** with *S. aureus* DNA gyrase (PDBID:3G7B)

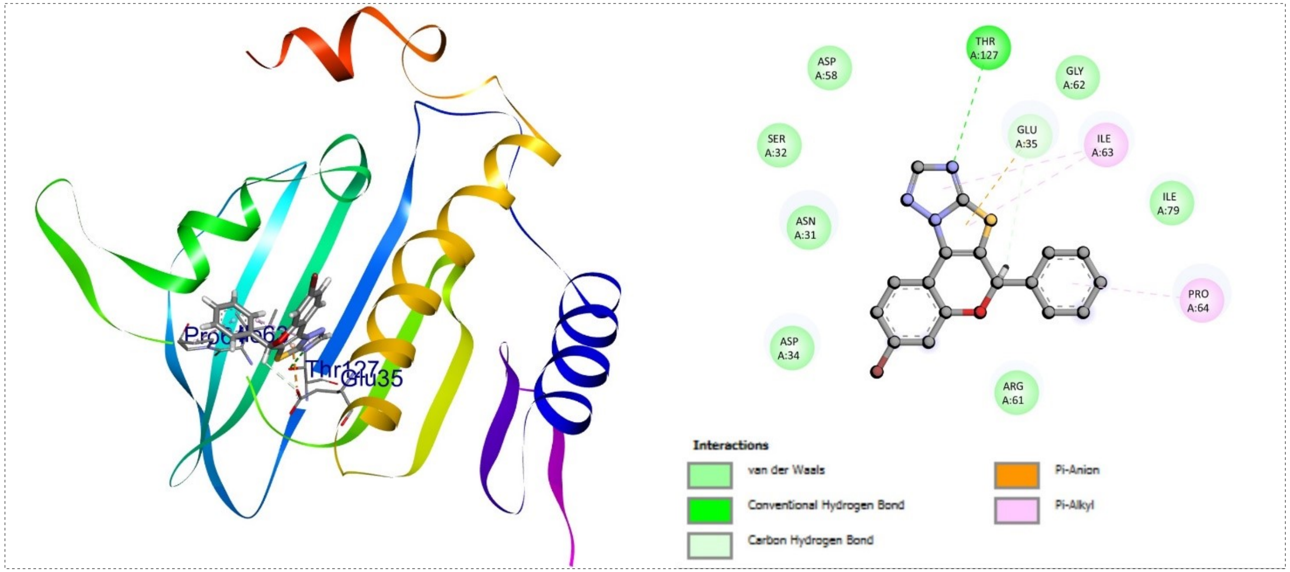

Fig S98. Binding interaction of compound **4c** with *S. aureus* DNA gyrase (PDBID:3G7B)

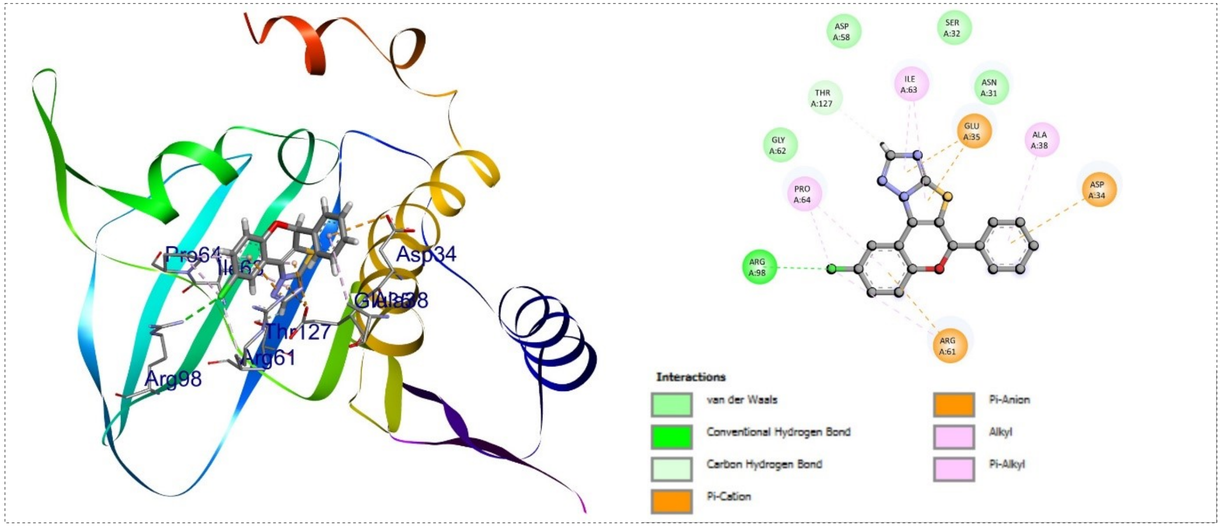

Fig S99. Binding interaction of compound **4d** with *S. aureus* DNA gyrase (PDBID:3G7B)

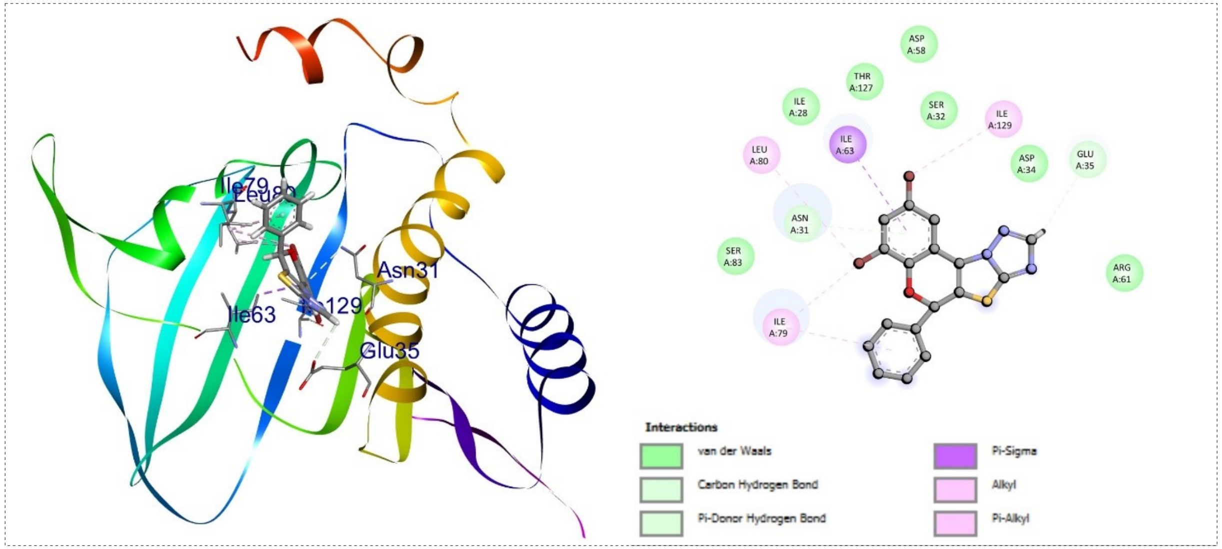

Fig S100. Binding interaction of compound **4f** with *S. aureus* DNA gyrase (PDBID:3G7B)

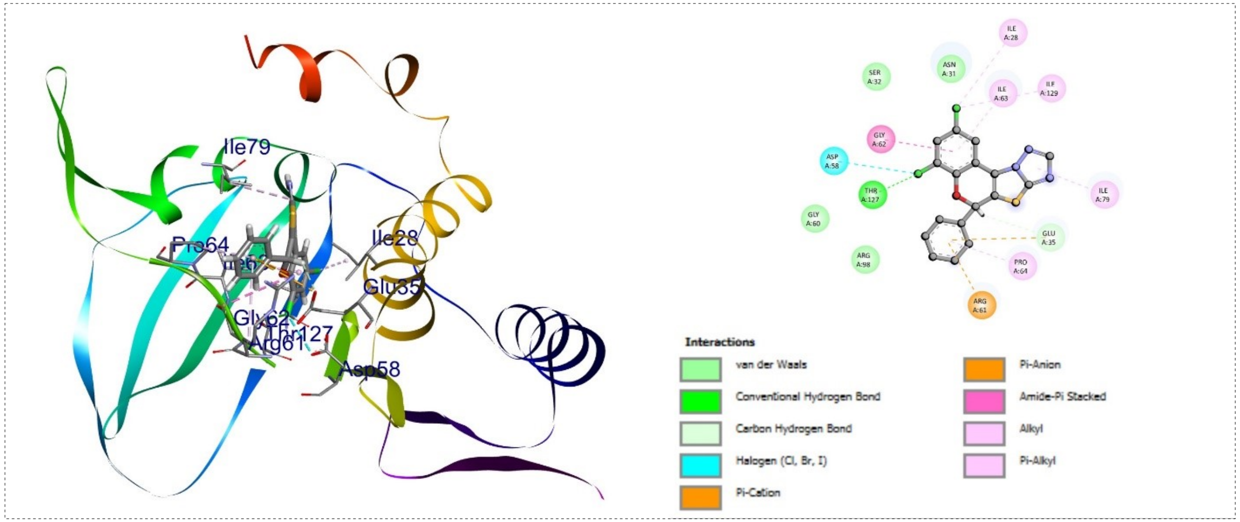

Fig S101. Binding interaction of compound **4g** with *S. aureus* DNA gyrase (PDBID:3G7B)

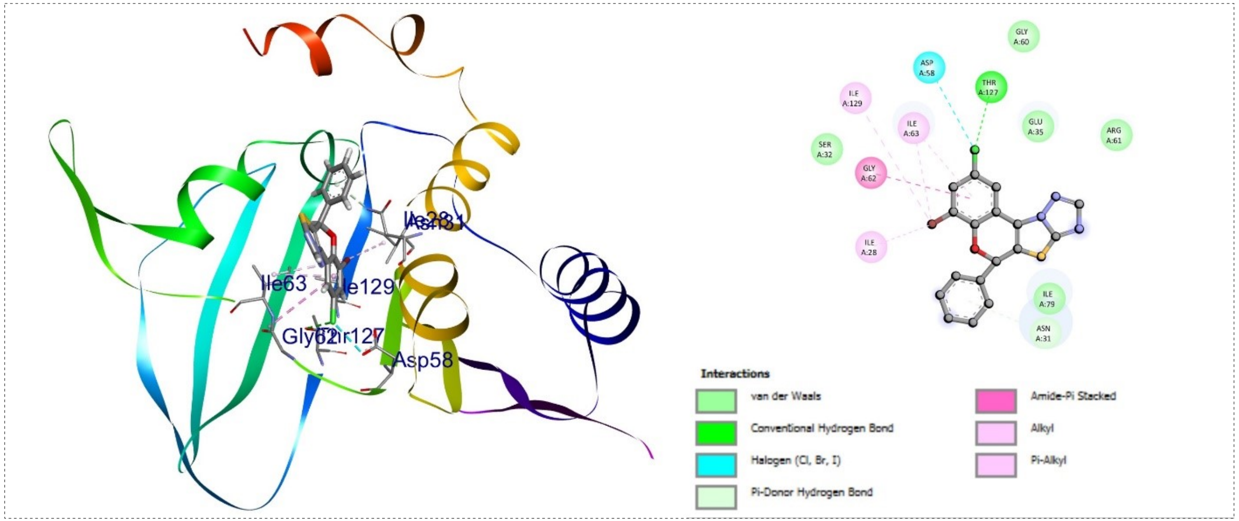

Fig S102. Binding interaction of compound **4h** with *S. aureus* DNA gyrase (PDBID:3G7B)

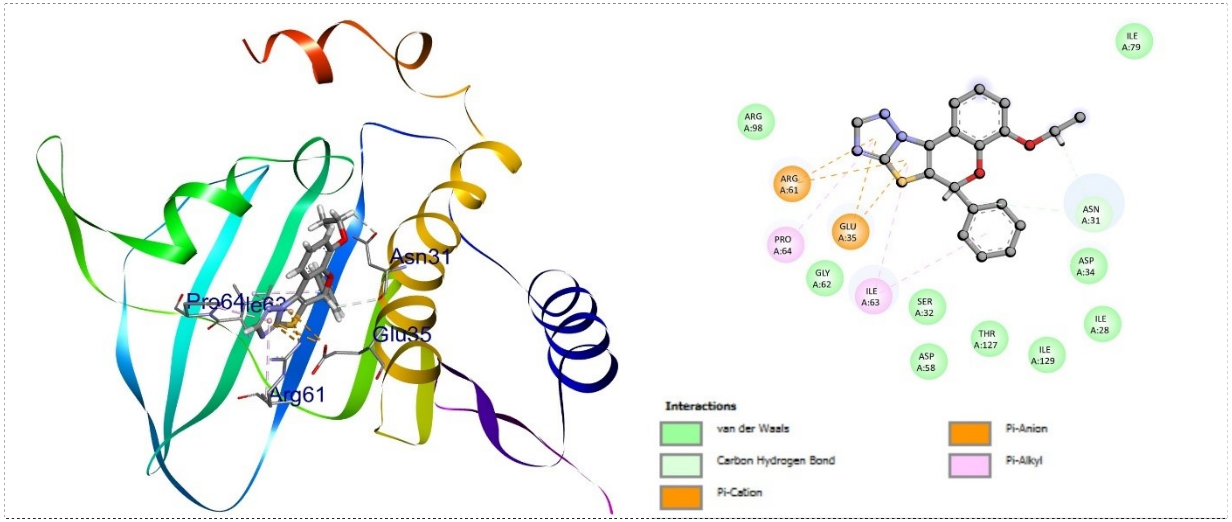

Fig S103. Binding interaction of compound **4j** with *S. aureus* DNA gyrase (PDBID:3G7B)

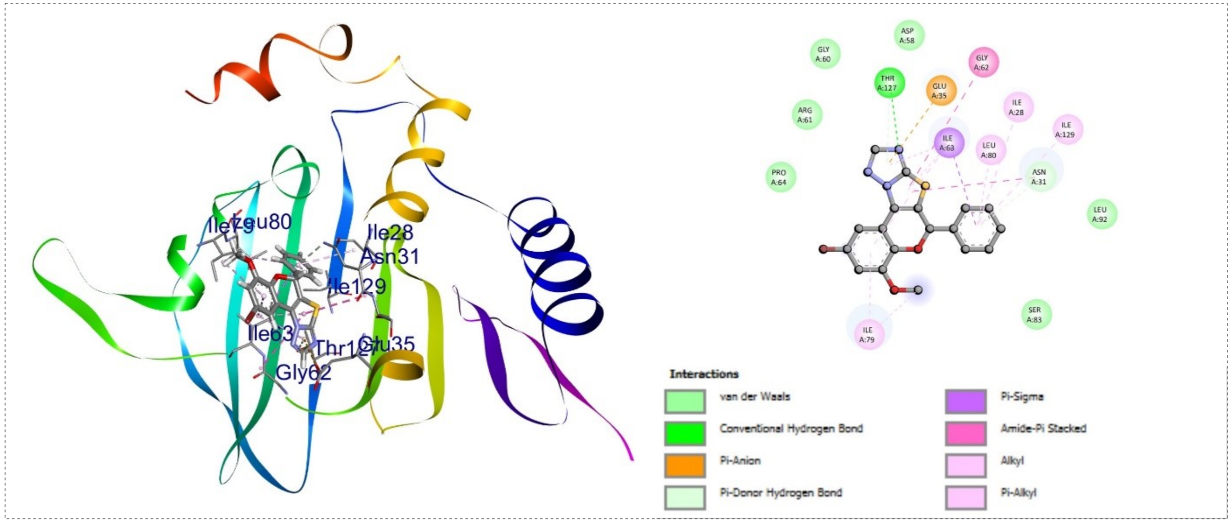

Fig S104. Binding interaction of compound **4k** with *S. aureus* DNA gyrase (PDBID:3G7B)

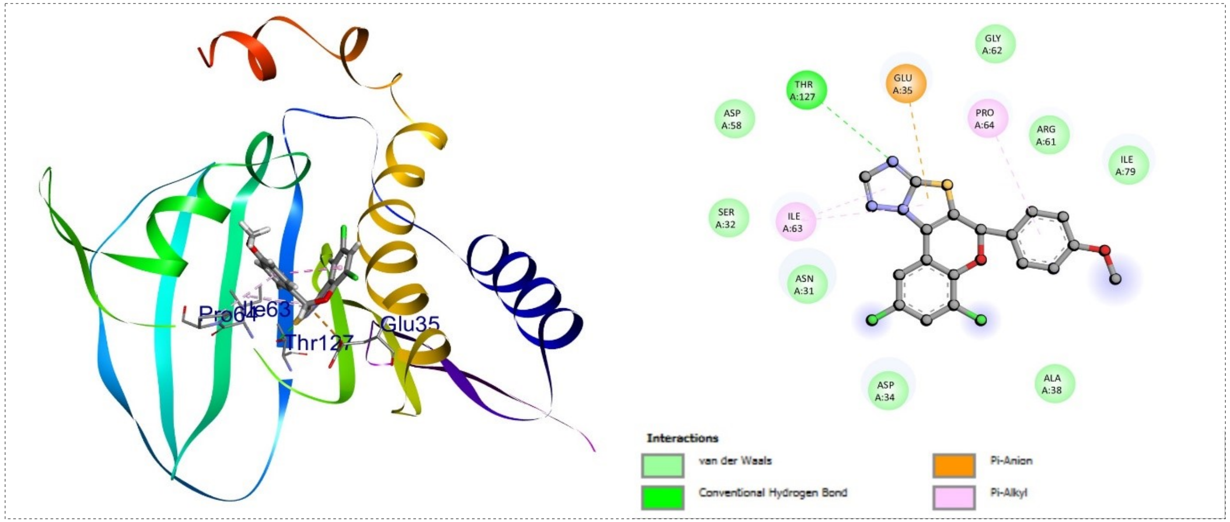

Fig S105. Binding interaction of compound **4l** with *S. aureus* DNA gyrase (PDBID:3G7B)

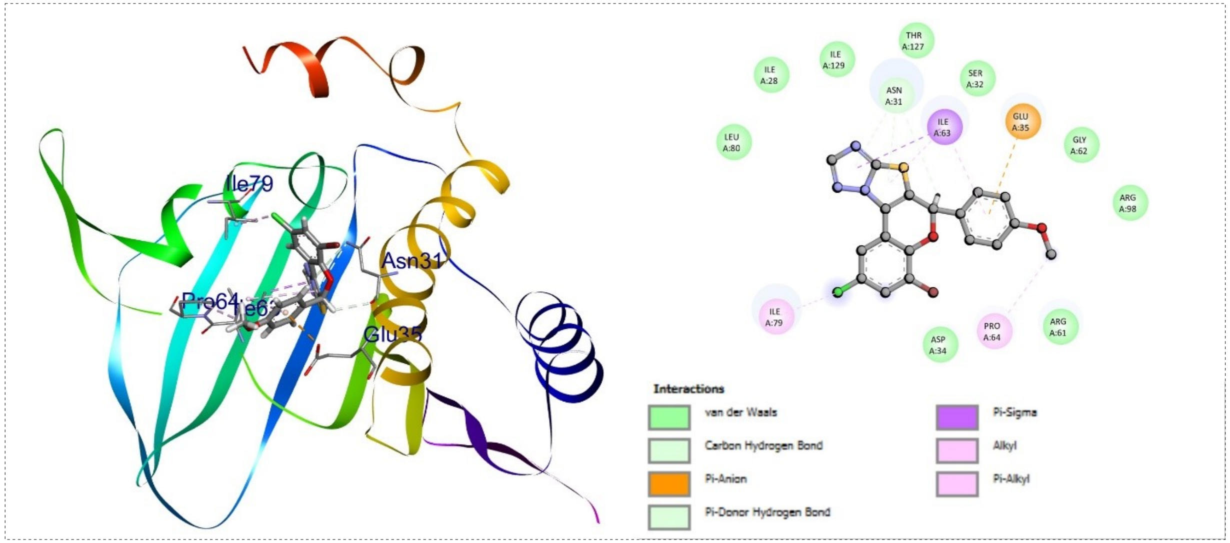

Fig S106. Binding interaction of compound **4m** with *S. aureus* DNA gyrase (PDBID:3G7B)

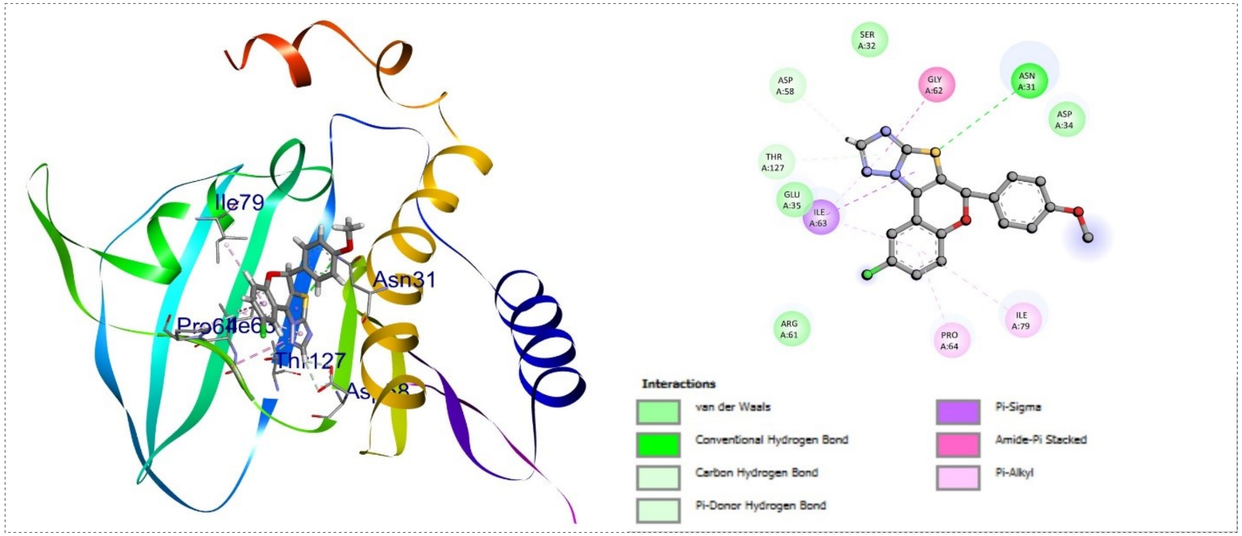

Fig S107. Binding interaction of compound **4n** with *S. aureus* DNA gyrase (PDBID:3G7B)

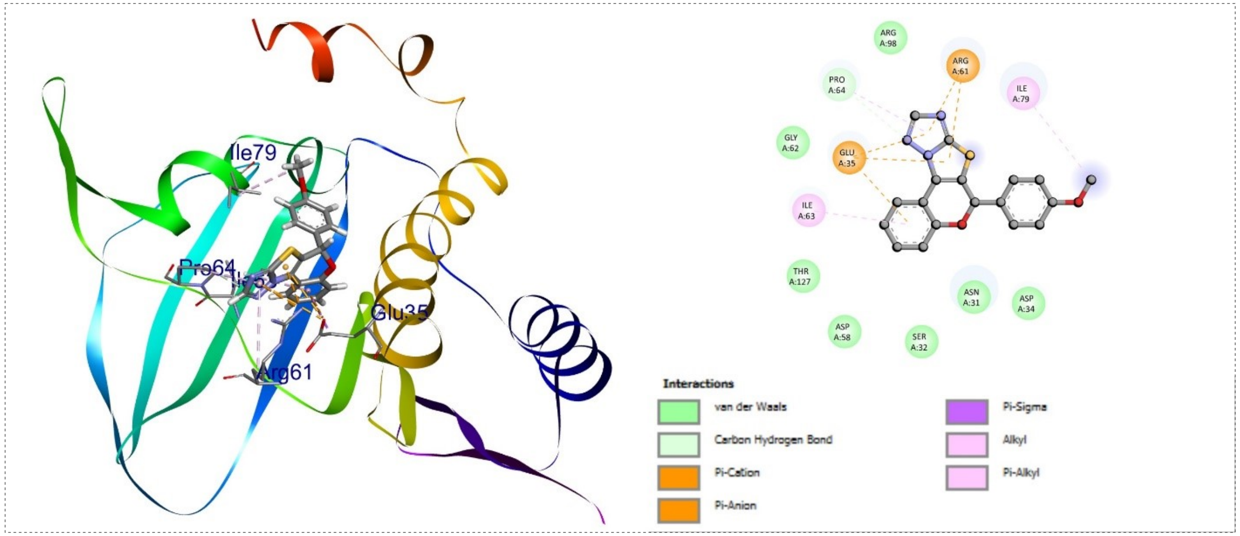

Fig S108. Binding interaction of compound **4o** with *S. aureus* DNA gyrase (PDBID:3G7B)

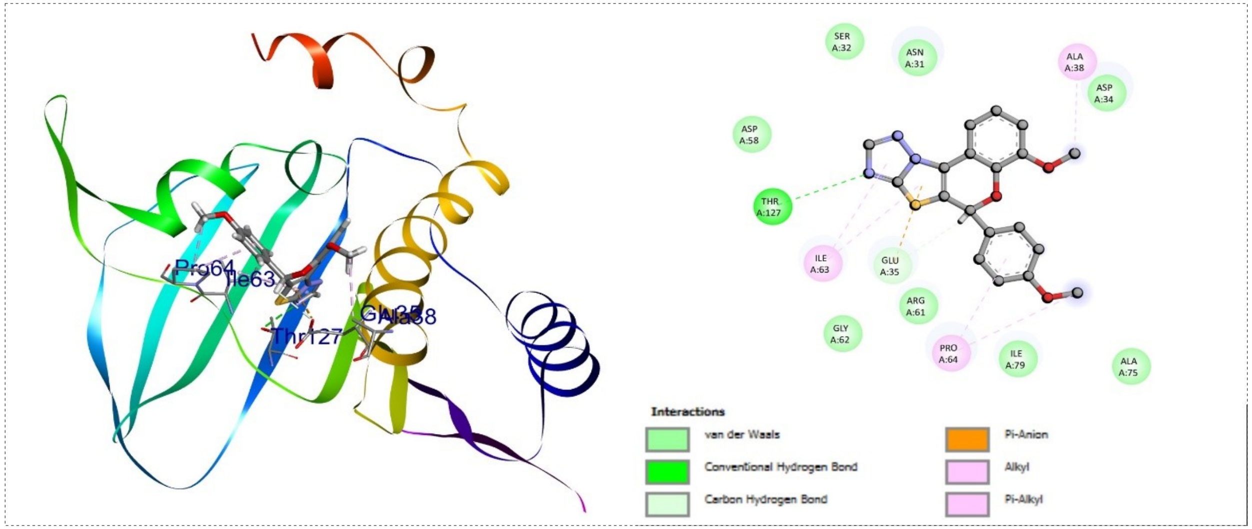

Fig S109. Binding interaction of compound **4p** with *S. aureus* DNA gyrase (PDBID:3G7B)

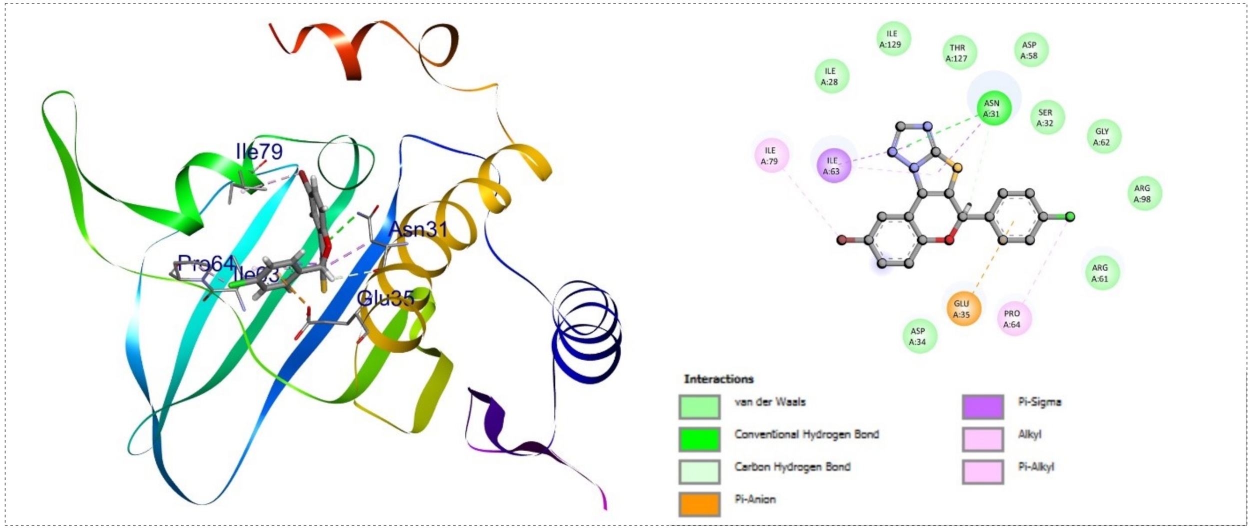

Fig S110. Binding interaction of compound **4q** with *S. aureus* DNA gyrase (PDBID:3G7B)

5.2. DFT study Calculations

Density functional theory (DFT) with Becke-3-Lee Yang Parr (B3LYP) level at standard 6-311G++(d,p) represent one of the most effective techniques for investigation of the reactivity and stability of thiazolo-triazole compounds.[12-17] Gaussian 09W and GaussView 6.0 programmes were used to perform the DFT calculations and visual representations for the synthesized compounds.[18-21] The geometry optimization and molecular orbital energies (E_{LUMO}, E_{HOMO}, and ΔE_g) were computed. Global reactivity descriptors like ionization potential (I), electron affinity (A), electronegativity (χ), chemical potential (μ), global hardness (η), global softness (S), global electrophilicity index (ω), global nucleophilicity index (ε), and extra electronic charge (ΔN) have been computed by mathematical equations (Eqs. 1-9).[22-24] Further, the molecular electrostatic potential (MEP) was determined at the B3LYP/6-311G(d,p) level basic set.

Energy Gap (ΔE_g) = E_{LUMO} - E_{HOMO} (1)
Ionization Potential (I) = - E_{HOMO} (2)
Electron Affinity (A) = - E_{LUMO} (3)
Electronegativity (χ) = - (E_{LUMO} + E_{HOMO})/2 (4)
Chemical potential (μ) = (E_{LUMO} + E_{HOMO})/2 (5)
Global Hardness (η) = (E_{LUMO} - E_{HOMO})/2 (6)
Global Softness (S) = $1/2\eta$ (7)
Global Electrophilicity index (ω) = $\mu^2/2\eta$ (8)
Global Nucleophilicity index (ε) = $1/\omega$ (9)
Extra electronic charge (ΔN) = - μ/η (10)

Fig. S111. Optimized structures, HOMO and LUMO of the synthesized 2*H*-chromene-based-thiazolo-triazole derivatives 4(b-d), 4(f-h), 4(k-n), and 4q

**Compounds**	**Optimized structure**	**LUMO**	**HOMO**

4b

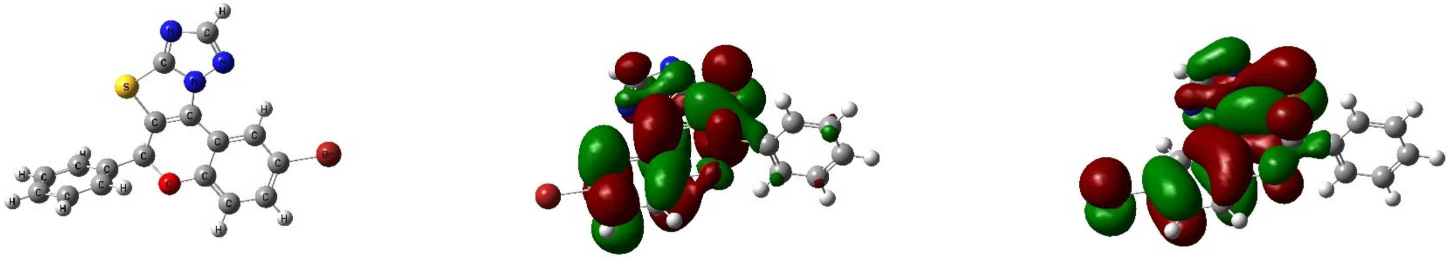

4c

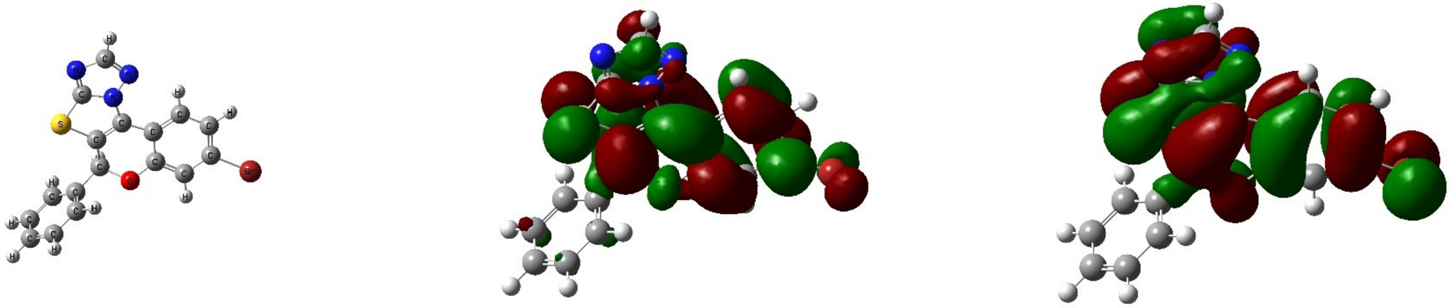

4d

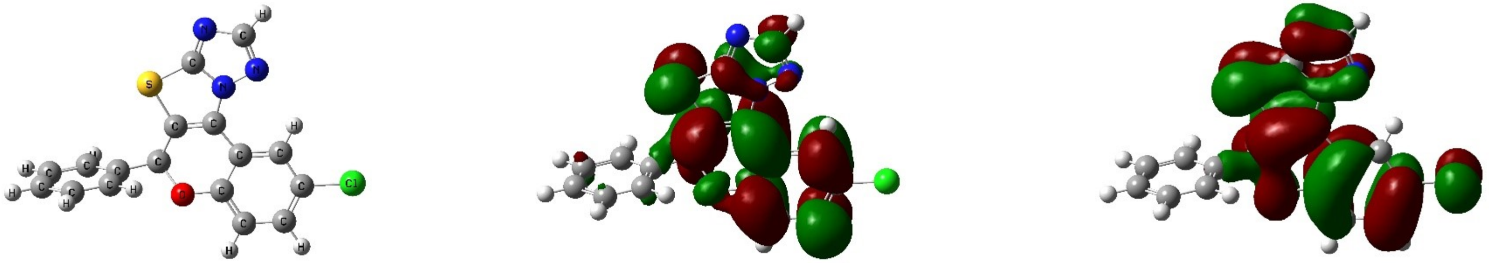

4f

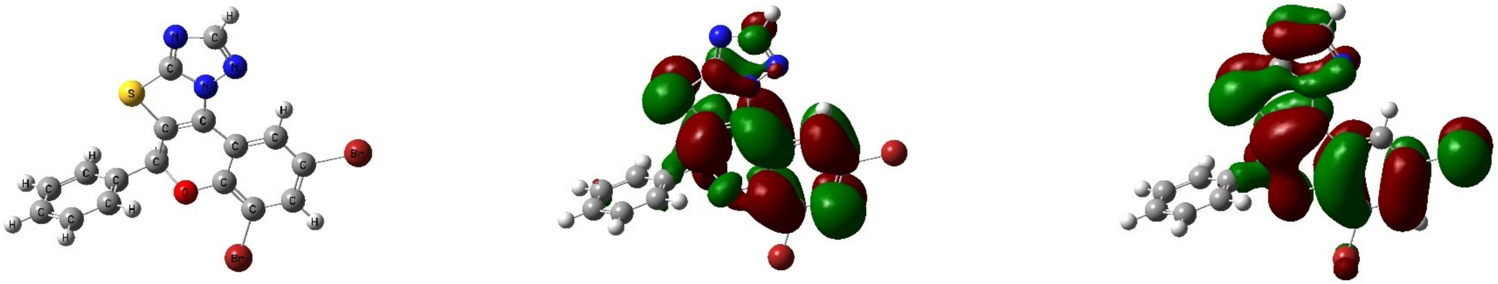

4g

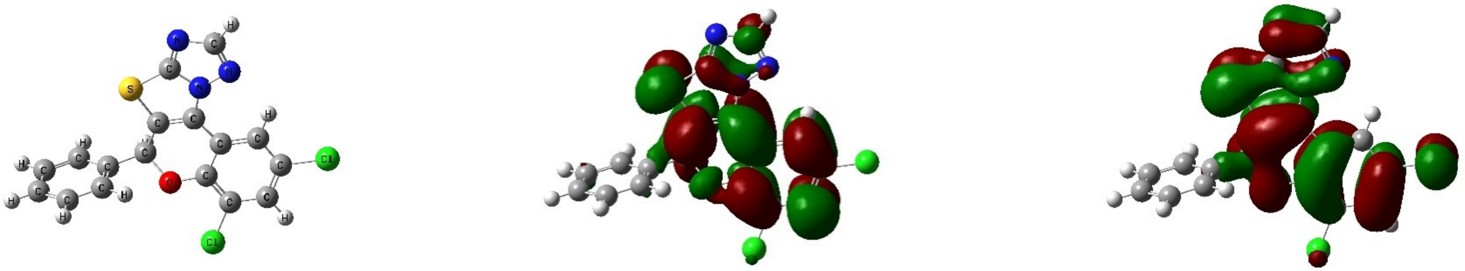

4h

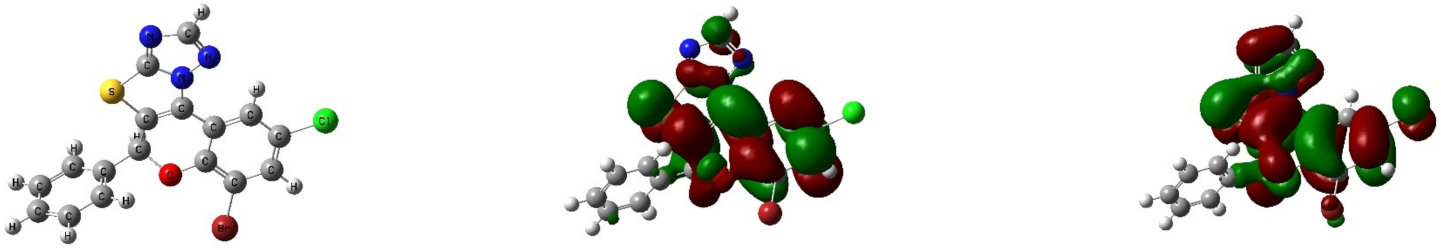

4k

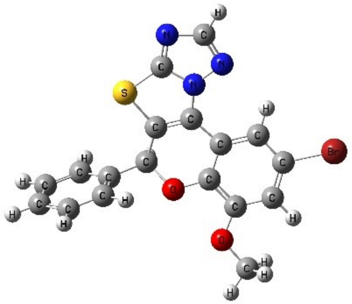

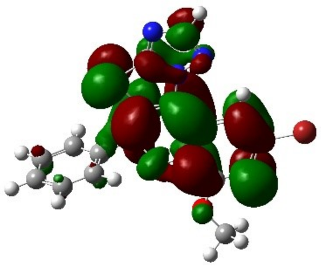

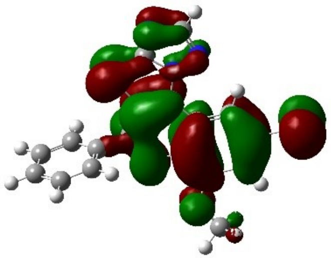

4l

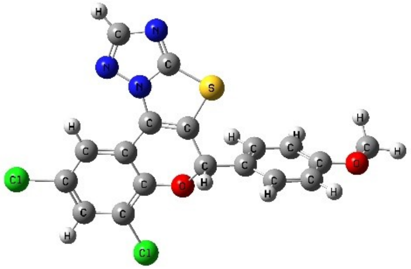

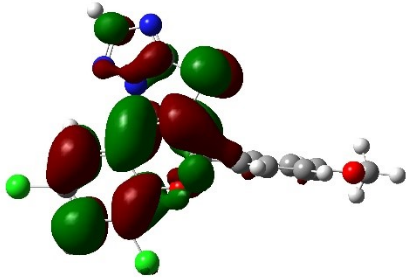

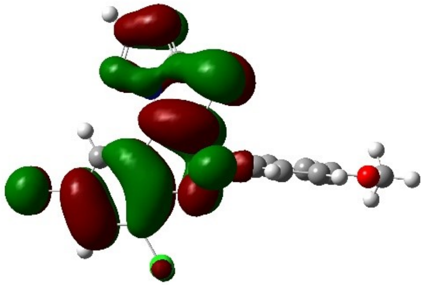

4m

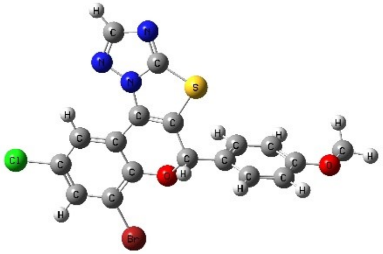

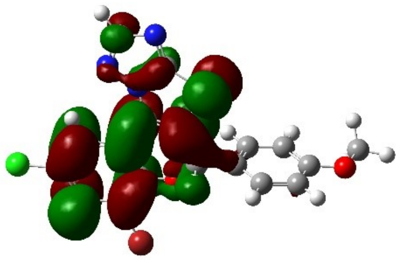

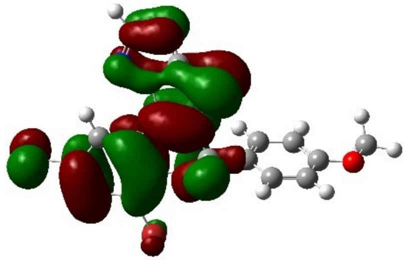

4n

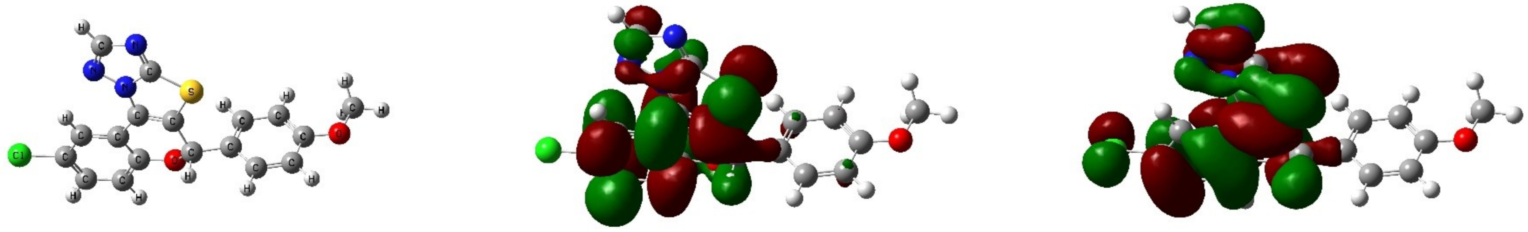

4q

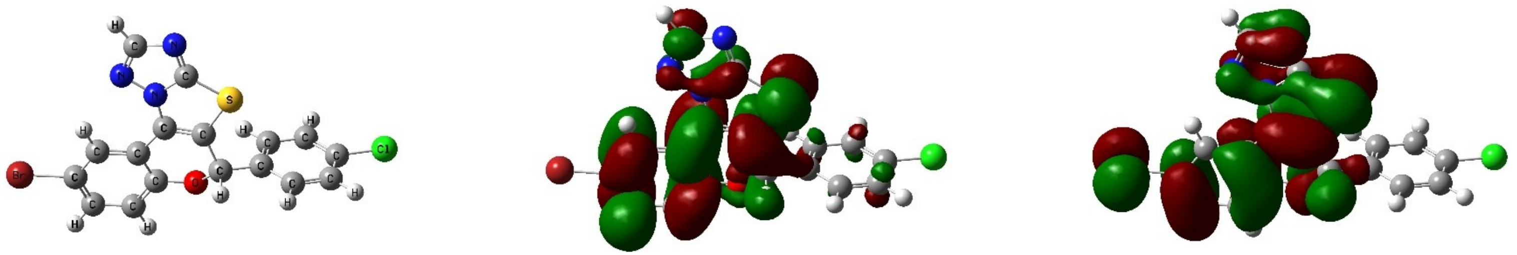

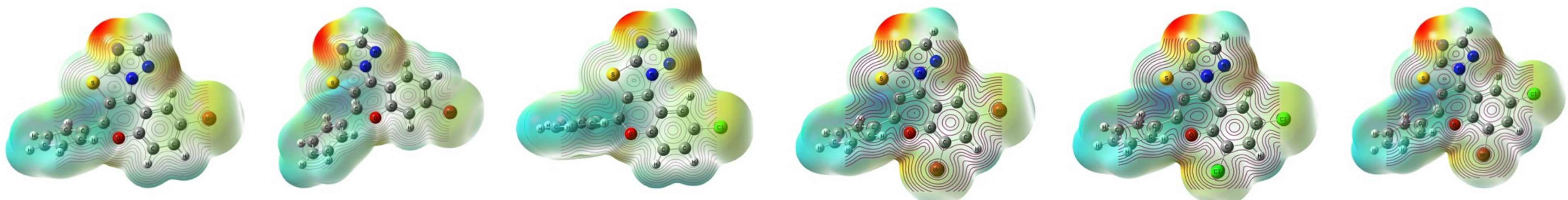

4b 4c 4d 4f 4g 4h

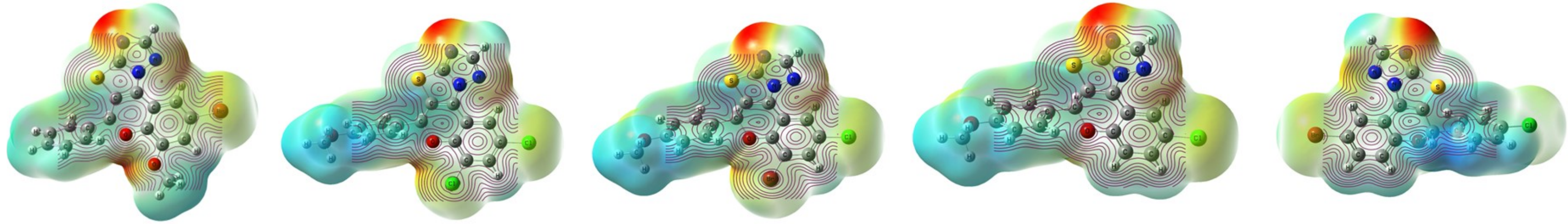

4k 4l 4m 4n 4q

Fig. S112. MEP plots of synthesized 2*H*-chromene-based-thiazolo-triazole derivatives 4(b-d), 4(f-h), 4(k-n), and 4q evaluated using Basis set under B3LYP/6-311G++(d,p)

5.3. *In silico Drug-Likeness and ADMET predictions*

All the potent compounds 4a, 4e, 4f, 4h, 4i and standard drugs Gentamicin, Doxorubicin were evaluated for their in silico pharmacokinetic properties (Druglikeness and ADMET) using two publicly available computational platforms SwissADME (http://www.swissadme.ch/),[25] Pro Tox 3.0 (https://tox.charite.de/protox3/),[26] and pkCSM (http://biosig.unimelb.edu.au/pkcsm/prediction).[27]

5.4. *Toxicity prediction of potent compounds 4a, 4e, 4f, 4h and 4i with standard Gentamicin and Doxorubicin*

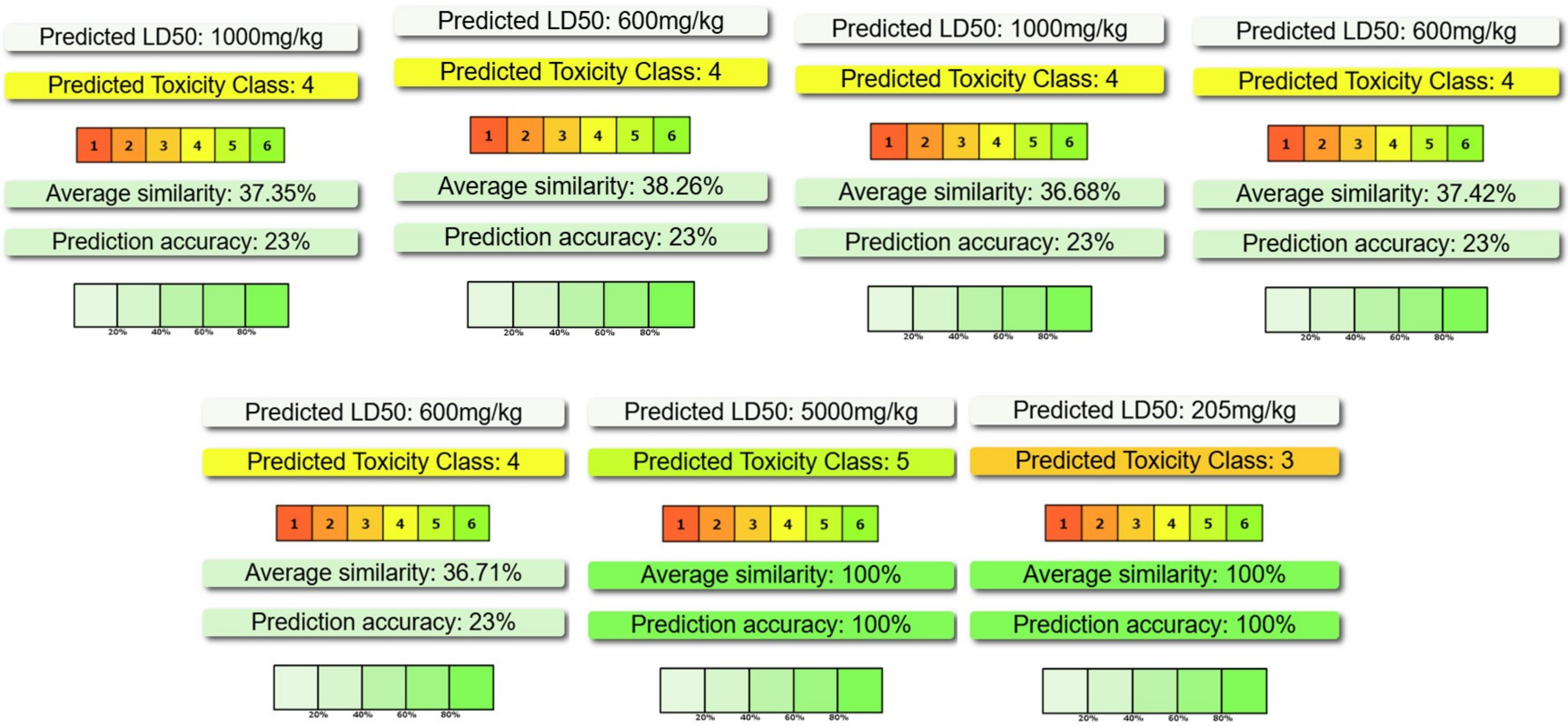

Figure S113. Predicted classes of toxicity of top three potent compounds **4a**, **4e, 4f, 4i,** and **4h** standard drug **Gentamicin** and **Doxorubicin.**

6. References

1. V. Kumar and S. Kumar, *J. Mol. Struct.*, 2024, **1310**, 138285.
2. G. M. Sheldrick, *Acta Crystallogr., Sect. A*, 2008, **64**, 112–122.
3. O. V. Dolomanov, L. J. Bourhis, R. J. Gildea, J. A. K. Howard and H. Puschmann, *J. Appl. Crystallogr.*, 2009, **42**, 339–341.
4. S. S. Hamdani, B. A. Khan, M. N. Ahmed, S. Hameed, K. Akhter, K. Ayub and T. Mahmood, *J. Mol. Struct.*, 2020, **1200**, 127085.
5. G. M. Sheldrick, *Acta Crystallogr., Sect. C*, 2015, **71**, 3–8.
6. B. S. Panda, B. Samanta, E. Naik, S. Nayak, P. Pragyandipta, S. Mohapatra and P. K. Naik, *J. Mol. Struct.*, 2025, **1347**, 143205.
7. C. R. Sahoo, S. Maharana, C. P. Mandhata, A. K. Bishoyi, S. K. Paidesetty and R. N. Padhy, *Saudi J. Biol. Sci.*, 2020, **27**, 1580–1586.
8. I. Wiegand, K. Hilpert and R. E. W. Hancock, *Nat. Protoc.*, 2008, **3**, 163–175.
9. R. N. Mishra, M. A. Ahemad, J. Panda, S. Nayak, S. Mohapatra and C. R. Sahoo, *RSC Adv.*, 2025, **15**, 2930.
10. S. P. Parida, S. Mohapatra, S. Nayak, S. Mohapatra, J. Panda and C. R. Sahoo, *ChemistrySelect*, 2024, **9**, e202402223.
11. B. S. Panda, B. Samanta, S. S. S. S. S. Ambadipudi, S. Nayak, V. L. Nayak, S. Ramakrishna, S. Mohapatra, P. M. Behera and L. Samanta, *ChemistrySelect*, 2024, **9**, e202400115.
12. L. Zhang, L. Qiu, X. Xie, J. Ye and A. Hu, *J. Mol. Struct.*, 2024, **1310**, 138047.
13. S. M. Eldaly, H. M. Hassaneen and N. H. Metwally, *J. Mol. Struct.*, 2025, **1327**, 141204.
14. P. Rajesab, B. S. Mathada, V. Niranjan, L. Sinha, A. S. Setlur, A. Maurya, K. Chandrashekar and O. Prasad, *J. Mol. Struct.*, 2025, **143166**.
15. K. Rajagopal, S. Dhandayutham, M. Nandhagopal, M. Narayanasamy, M. I. Elzagheid, L. Rhyman and P. Ramasami, *J. Mol. Struct.*, 2022, **1255**, 132374.
16. A. Al Sheikh Ali, D. Khan, A. Naqvi, F. F. Al-Blewi, N. Rezki, M. R. Aouad and M. Hagar, *ACS Omega*, 2020, **6**, 301–316.
17. H. S. El-Hema, S. M. Soliman, W. El-Dougdoug, M. H. Ahmed, A. Abdelmajeid, E. S. Nossier, M. F. Hussein, A. A. Alrayes, M. Hassan, N. A. Ahmed and A. A. H. Abdel-Rahman, *ACS Omega*, 2025, **10**, 9703–9717.
18. C. Peng, P. Y. Ayala, H. B. Schlegel and M. J. Frisch, *J. Comput. Chem.*, 1996, **17**, 49–56.
19. M. J. Frisch, J. A. Pople and J. S. Binkley, *J. Chem. Phys.*, 1984, **80**, 3265–3269.

20. J. G. Stuart and J. W. Jebaraj, *Indian J. Chem.*, 2023, **62**, 1061–1080.
21. B. S. Panda, M. A. Ahemad, S. Mohapatra, E. Naik, S. Nayak, P. K. Naik, D. Bhattacharya, C. R. Sahoo and M. K. Sahoo, *J. Mol. Struct.*, 2024, **1318**, 139323.
22. A. S. Badran, A. Ahmed, A. I. Nabeel and M. A. Ibrahim, *J. Mol. Struct.*, 2024, **1298**, 137030.
23. J. Luo, Z. Q. Xue, W. M. Liu, J. L. Wu and Z. Q. Yang, *J. Phys. Chem. A*, 2006, **110**, 12005–12009.
24. P. Ramesh, M. L. Caroline, S. Muthu, B. Narayana, M. Raja and A. B. Geoffrey, *Spectrochim. Acta, Part A*, 2019, **222**, 117190.
25. A. Daina, O. Michielin and V. Zoete, *Sci. Rep.*, 2017, **7**, 42717.
26. P. Banerjee, A. O. Eckert, A. K. Schrey and R. Preissner, *Nucleic Acids Res.*, 2018, **46**, W257–W263.
27. D. E. V. Pires, T. L. Blundell and D. B. Ascher, *J. Med. Chem.*, 2015, **58**, 4066–4072.
